# Synthesis, Antiproliferative Activity, and ADME Profiling of Novel Racemic and Optically Pure Aryl-Substituted Purines and Purine Bioisosteres

**DOI:** 10.3390/biom15030351

**Published:** 2025-02-28

**Authors:** Martina Piškor, Astrid Milić, Sanja Koštrun, Maja Majerić Elenkov, Petra Grbčić, Sandra Kraljević Pavelić, Krešimir Pavelić, Silvana Raić-Malić

**Affiliations:** 1 Department of Organic Chemistry, University of Zagreb, Faculty of Chemical Engineering and Technology, Marulićev trg 19, 10000 Zagreb, Croatia; mpiskor@fkit.unizg.hr; 2 Selvita d.o.o., 10000 Zagreb, Croatia; astrid.milic@selvita.com (A.M.);; 3 Division of Organic Chemistry and Biochemistry, Ruder Bošković Institute, Bijenička 54, 10000 Zagreb, Croatia; maja.majeric-elenkov@irb.hr; 4 Juraj Dobrila University of Pula, Faculty of Medicine, 52100 Pula, Croatia; petra.grbcic@unipu.hr (P.G.); pavelic@unipu.hr (K.P.); 5 Faculty of Health Studies, University of Rijeka, Viktora Cara Emina 5, 51000 Rijeka, Croatia; sandra.kraljevic.pavelic@fzsri.uniri.hr

**Keywords:** purine, purine bioisosteres, antiproliferative activity, ADME profiling

## Abstract

The aim of this study was to synthesize new racemic and optically pure aryl-substituted purine bioisosteres using ultrasound-assisted Cu(I)-catalyzed Huisgen 1,3-dipolar cycloaddition. Regioselective synthesis of α-azido alcohols was applied to afford heterocycles with a 2-hydroxyeth-1-yl linker. Catalytic asymmetric synthesis using halohydrin dehalogenase in the ring-opening of epoxides gave enantioenriched azido alcohols, which subsequently afforded *R*- and *S*-enantiomers of purine and pyrrolo[2,3-*d*]pyrimidines with a 1-hydroxyeth-2-yl linker. The newly synthesized compounds were evaluated in vitro for their antiproliferative activity against four malignant tumor cell lines. The influence of regioisomerism and the stereochemistry of the hydroxyethyl group, as well as a *N*-heterocyclic scaffold linked to the aryl moiety on cytostatic activity was evaluated. Of all the compounds tested, purine **40a** and pyrrolo[2,3-*d*]pyrimidine **45a** derivatives with *p*-trifluoromethyl-substituted aryl connected to 1,2,3-triazole via a 2-hydroxyeth-1-yl spacer showed promising submicromolar antiproliferative activity. In addition, compound **45a** exhibited selectivity towards the tumor cell line, with a selectivity index (SI) of 40, moderate clearance, and good membrane permeability.

## 1. Introduction

Due to the increasing incidence of cancer and the problems of multiple side effects and resistance to classical chemotherapeutic agents, the search for new antitumor agents is becoming more urgent [1]. It was estimated that in 2020, more than 19.3 million new cancer cases were diagnosed and approximately 10.0 million deaths were recorded from cancer worldwide [2]. The growing proportion of chiral drug molecules on the market takes advantage of the chiral switching of already marketed racemates and develops de novo enantiomerically pure compounds [3,4,5,6]. As a consequence, enantioselective synthesis has gained considerable attention in medicinal chemistry because the different enantiomers or diastereomers of a molecule often exhibit different biological activities [7,8,9]. These differences between enantiomers may arise not only from drug interactions at the receptors, but also from their absorption, distribution, metabolism, and excretion [10]. The synthesis of enantiomerically pure compounds is one of the most demanding challenges in organic chemistry. Among successful applications of transition metal catalysis, organocatalysis, and biocatalysis, enzymes have emerged as environmentally friendly catalysts for high regioselective, chemoselective, and stereoselective transformations [11,12]. Halohydrin dehalogenases (HHDHs) are biotechnologically interesting enzymes that catalyze a wide range of bond formations, such as carbon–carbon, carbon–nitrogen, carbon–oxygen, carbon–chlorine, and carbon–bromine. HHDHs have been used for the synthesis of highly optically active epoxides and 1,2-azido alcohols [13]. Nucleobase-derived compounds have appeared as important pharmacophores interacting with the synthesis and functions of nucleic acids and enzymes [14,15,16,17,18,19]. These *N*-heterocycles have gained importance in recent years in combating different types of cancer [20,21]. Some purine-based drugs are well known antimetabolites that interfere with DNA replication and cell division by causing cell death when incorporated into DNA or RNA [22,23]. Purine-based compounds have emerged as potent kinase inhibitors that play a pivotal role in the proliferation, migration, and survival of human tumor cells [16,24,25,26]. First-generation purine and second-generation deazapurine inhibitors of heat shock protein 90 (HSP90) have also been developed [27]. Recently, some purine analogs have been identified as inhibitors targeting the microtubule-severing enzyme katanin, which plays an essential role in various carcinomas [28]. Others have found application as antagonists of the Smoothened (SMO) receptor, which is the most druggable target in the Hedgehog signaling pathway for anticancer agents [29].

**Figure 1:**
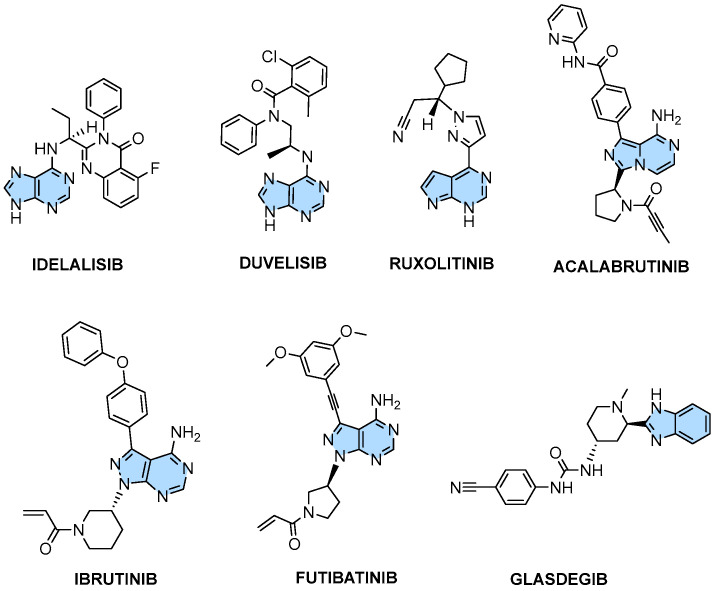
Chiral purine and purine isostere derivatives as antitumor drugs.

Among the increasing number of small-molecule targeted drugs that have been developed for the treatment of malignancies, some chiral purine and purine isostere derivatives are associated with antitumor activities given their ability to interfere with carcinogenesis processes, and have been approved by the FDA for the treatment of various types of cancer (Figure 1) [30,31,32,33,34].

Targeted therapies of idelalisib and duvelisib include the inhibition of phosphatidylinositol 3-kinase (PI3K), while acalabrutinib and ibrutinib emerged as Bruton’s tyrosine kinase (BTK) inhibitors [35] and ruxolitinib as Janus kinase (JAK) inhibitors [36]. Futibatinib is a highly selective irreversible fibroblast growth factor receptor 1 (FGFR) inhibitor in patients with advanced solid tumors [37] and glasdegib acts as a SMO inhibitor [38].

**Figure 2:**
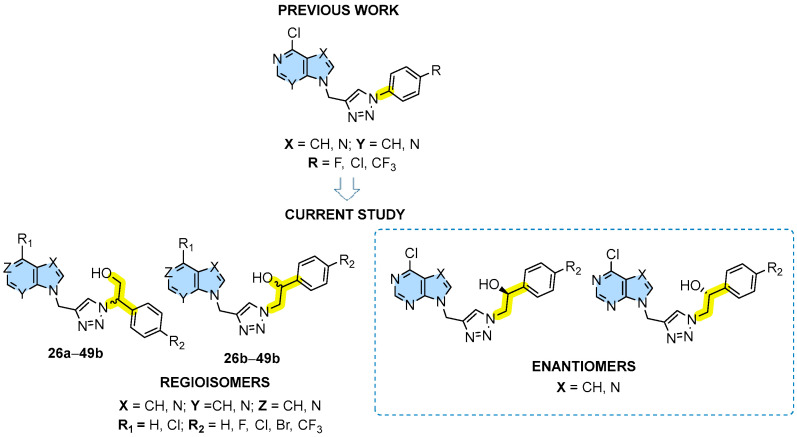
Design of novel racemic α- and β-regioisomers and optically pure aryl-substituted purine bioisosteres.

In continuation of our previous study on the antiproliferative activity of purine and pseudopurine derivatives [39,40,41,42,43], herein we propose the synthesis of selected racemic *N*-heterocyclic analogs with hydroxyethyl linker containing primary and secondary hydroxyl groups, instead of directly connected aromatic and 1,2,3-triazole scaffolds (Figure 2) [43]. The introduction of a spacer between the heterocyclic moieties enables additional flexibility in structural preorganization, while the introduction of the primary and secondary hydroxyl groups affects both the biological and physicochemical properties of the prepared molecules [44]. The physicochemical properties can be significantly altered by the introduction of a hydroxyl group, mainly by increasing the polarity and thus the solubility in aqueous media.

Since aliphatic alcohols have pKa values above 14, they do not alter the charge of a ligand, but have the potential for a moderate increase in hydrophilicity without impairing membrane permeation. In addition to the regioisomeric effect, the impact of the chirality of enantiomerically enriched *N*-aryl-substituted 6-chloropurine and 4-chloropyrrolo[2,3-*d*]pyrimidine derivatives on antiproliferative activity was evaluated.

## 2. Materials and Methods

### 2.1. General

Solvents and chemicals, including starting purines and purine bioisosters **1**–**5**, as well as para-substituted 2-phenyloxiranes **11**–**15** and phenacyl bromide **21**–**25**, were purchased from Sigma-Aldrich (St. Louis, MO, USA), Alfa Aesar (Haverhill, MA, USA), and Fisher Scientific International (Pittsburgh, PA, USA). The progress of all reactions was monitored by thin layer chromatography on pre-coated Merck (Darmstadt, Germany) silica gel plates 60F254. The synthesized compounds were purified via column chromatography using Fluka (Buchs, Switzerland) silica gel (0.063−0.2 mm) and the appropriate solvent. All NMR spectra were recorded using a Bruker 300, 400, and 600 MHz NMR spectrometer (Bruker Biospin, Rheinstetten, Germany) in deuterated DMSO solutions, with TMS as the internal standard. The melting points of all newly synthesized compounds were determined using a Koffler (Reichert, Vienna) hot stage microscope. The ^1^H (δ/ppm) and ^13^C NMR (δ/ppm) spectra of compounds **26a,b**–**49a,b** can be found in the Supporting Information (Figures S1–S48). The purity of all newly synthesized compounds was determined by elemental analysis and the elemental analysis results are within 0.5% of the theoretical values. The UV/Vis absorption spectra of compounds 4**0a**, **45a**, and **49b** were measured at a concentration of 1 × 10⁻⁴ mol dm⁻³ in phosphate buffer and DMSO (99:1, *v*/*v*) at pH 7.4 using a Varian Cary 50 spectrophotometer in double-beam mode at 25 °C.

### 2.2. Experimental Procedures for the Synthesis of Compounds

Moreover, 1-(Prop-2-yn-1-yl)-1*H*-indole **6 [45]**, 1-(prop-2-yn-1-yl)-1*H*-benzimidazole **7 [43]**, 6-chloro-9-(prop-2-yn-1-yl)-9*H*-purine **8** [43,46], 4-chloro-7-(prop-2-yn-1-yl)-7*H*-pyrrolo[2,3-*d*]pyrimidine **9 [47]**, 4-chloro-1-(prop-2-yn-1-yl)-1*H*-imidazo[4,5-*c*]pyridine **10 [43]**, 2-azido-2-phenylethan-1-ol **16a [48]**, 2-azido-2-(4-fluorophenyl)ethan-1-ol **17a [49]**, 2-azido-2-(4-chlorophenyl)ethan-1-ol **18a [50]**, 2-azido-2-(4-bromophenyl)ethan-1-ol **19a [50]**, 2-azido-2-(4-(trifluoromethyl)phenyl)ethan-1-ol **20a [49]**, 2-azido-1-phenylethan-1-ol **16b [51]**, 2-azido-1-(4-fluorophenyl)ethan-1-ol **17b [51]**, 2-azido-1-(4-chlorophenyl)ethan-1-ol **18b [52]**, 2-azido-1-(4-bromophenyl)ethan-1-ol **19b** [51], and 2-azido-1-(4-(trifluoromethyl)phenyl)ethan-1-ol **20b [49]** were synthesized according to a known procedure. Enantioenriched (ee = 89−99%) ß-azido alcohols (*R*), (*S*)-**16b**-(*R*), (*S*)-**18b** and (*R*), and (S)-**20b** were also synthesized according to a well-known procedure given in the literature [49].

#### 2.2.1. General Procedure for the Synthesis of Racemic Aryl-Substituted Purine Bioisosteres with Primary Hydroxyl (**26a**−**49a**) and Secondary Hydroxyl (**26b**−**49b**) Groups

The corresponding *N*-propargylated heterocyclic base **6**−**10** (1 eq.) was dissolved in methanol, and the corresponding racemic 1,2-azido alcohol with primary hydroxyl group **16a,b**−**20a,b** (1.2 eq.) and Cu(OAc)_2_ (0.05 eq.) were added. The reaction mixture was irradiated under ultrasound for 2 h in a laboratory ultrasonic cleaning bath. The solvent was removed under reduced pressure, and the residue was purified using column chromatography.

**1-((1-(2-Hydroxy-1-phenylethyl)-1*H*-1,2,3-triazol-4-yl)methyl)-1*H*-indole (26a)**

Compound **26a** was prepared using the procedure mentioned above from compound **6** (50 mg, 0.32 mmol) and 2-azido-2-phenylethanol **16a** (62 mg, 0.39 mmol). After purification via column chromatography (CH_2_Cl_2_/CH_3_OH = 30:1), compound **26a** was isolated as a white powder (43 mg, 42%, m.p. = 167–169 °C). ^1^H NMR (400 MHz, DMSO) *δ*/ppm: 8.23 (s, 1H, H-triazole), 7.58 (d, *J* = 7.8 Hz, 1H, H-7), 7.53 (d, *J* = 7.8 Hz, 1H, H-4), 7.43 (d, *J* = 3.1 Hz, 1H, H-2), 7.36–7.29 (m, 5H, H-Ph), 7.14–7.09 (m, 1H, H-6), 7.05–6.98 (m, 1H, H-5), 6.44 (d, *J* = 3.1 Hz, 1H, H-3), 5.82–5.65 (m, 1H, CHCH_2_), 5.46 (s, 2H, CH_2_), 5.26 (t, *J* = 5.3 Hz, 1H, OH), 4.27–4.20 (m, 1H, CH_A_-OH), 3.98–3.93 (m, 1H, CH_B_-OH). ^13^C NMR (151 MHz, DMSO) *δ*/ppm: 143.78, 137.83, 136.03, 129.08, 129.06, 128.68, 128.67, 127.61, 123.54, 121.55, 120.87, 119.58, 110.56, 101.41, 66.54, 63.55, 41.30. Anal. calcd. For C_19_H_18_N_4_O (Mr = 318.38): C, 71.68; H, 5.70; N, 17.60; found: C 71.61, H 5.66, N 17.57.

**1-((1-(2-Hydroxy-1-(4-fluorophenyl)ethyl)-1*H*-1,2,3-triazol-4-yl)methyl)-1*H*-indole (27a)**

Compound **27a** was prepared using the procedure mentioned above from compound **6** (50 mg, 0.32 mmol) and 2-azido-2-(4-fluorophenyl)ethanol **17a** (69 mg, 0.38 mmol). After purification via column chromatography (CH_2_Cl_2_/CH_3_OH = 50:1), compound **27a** was isolated as a yellow oil (101 mg, 94%). ^1^H NMR (600 MHz, DMSO) *δ*/ppm: 8.20 (s, 1H, H-triazole), 7.57 (d, *J* = 7.8 Hz, 1H, H-7), 7.53 (d, *J* = 7.8 Hz, 1H, H-4), 7.42 (d, *J* = 3.1 Hz, 1H, H-2), 7.39–7.36 (m, 2H, H-Ph), 7.18–7.15 (m, 2H, H-Ph), 7.12 (t, *J* = 7.2 Hz, 1H, H-6), 7.01 (t, *J* = 7.2 Hz, 1H, H-5), 6.43 (d, *J* = 3.1 Hz, 1H, H-3), 5.78–5.76 (m, 1H, CHCH_2_), 5.45 (s, 2H, CH_2_), 5.26 (t, *J* = 5.0 Hz, 1H, OH), 4.22–4.18 (m, 1H, CH_A_-OH), 3.96–3.92 (m, 1H, CH_B_-OH).^13^C NMR (151 MHz, DMSO) *δ*/ppm: 162.48 (d, *J_CF_* = 244.6 Hz), 143.97, 136.19, 134.22 (d, *J_CF_* = 3.0 Hz), 130.08 (d, *J_CF_* = 8.4 Hz), 129.20, 128.82, 123.69, 121.72, 121.02, 119.74, 116.04 (d, *J_CF_* = 21.5 Hz), 110.69, 101.59, 65.79, 63.66, 41.43. Anal. calcd. For C_19_H_17_FN_4_O (Mr = 336.37): C, 67.84; H, 5.09; N, 16.66; found: C, 67.91; H, 5.02; N, 16.60.

**1-((1-(2-Hydroxy-1-(4-chlorophenyl)ethyl)-1*H*-1,2,3-triazol-4-yl)methyl)-1*H*-indole (28a)**

Compound **28a** was prepared using the procedure mentioned above from compound **6** (50 mg, 0.32 mmol) and 2-azido-2-(4-chlorophenyl)ethanol **18a** (76 mg, 0.38 mmol). After purification via column chromatography (CH_2_Cl_2_/CH_3_OH = 30:1), compound **28a** was isolated as a colorless oil (58 mg, 51%). ^1^H NMR (400 MHz, DMSO) *δ*/ppm: 8.21 (s, 1H, H-triazole), 7.58 (d, *J* = 8.3 Hz, 1H, H-7), 7.53 (d, *J* = 7.9 Hz, 1H, H-4), 7.43 (d, *J* = 3.4 Hz, 2H, H-Ph), 7.39–7.41 (m, 1H, H-2), 7.34 (d, *J* = 8.6 Hz, 2H, H-Ph), 7.13 (t, *J* = 7.4 Hz, 1H, H-6), 7.02 (t, *J* = 7.4 Hz, 1H, H-5), 6.44 (d, *J* = 3.1 Hz, 1H, H-3), 5.80–5.76 (m, 1H, CHCH_2_), 5.46 (s, 2H, CH_2_), 5.29 (t, *J* = 5.3 Hz, 1H, OH), 4.23–4.17 (m, 1H, CH_A_-OH), 3.98–3.92 (m, 1H, CH_B_-OH).^13^C NMR (101 MHz, DMSO) *δ*/ppm: 143.86, 136.80, 136.03, 133.40, 129.67, 129.06, 128.67, 123.65, 121.57, 120.87, 119.59, 110.55, 101.44, 65.60, 63.38, 41.27. Anal. calcd. For C_19_H_17_ClN_4_O (Mr = 352.82): C, 64.68; H, 4.86; N, 15.88; found: C, 64.61; H, 4.91; N, 15.81.

**1-((1-(2-Hydroxy-1-(4-bromophenyl)ethyl)-1*H*-1,2,3-triazol-4-yl)methyl)-1*H*-indole (29a)**

Compound **29a** was prepared using the procedure mentioned above from compound **6** (50 mg, 0.32 mmol) and 2-azido-2-(4-bromophenyl)ethanol **19a** (93 mg, 0.38 mmol). After purification via column chromatography (CH_2_Cl_2_/CH_3_OH = 50:1), compound **29a** was isolated as a yellow oil (31 mg, 24%). ^1^H NMR (600 MHz, DMSO) ^1^H NMR (300 MHz, DMSO) *δ*/ppm: 8.14 (s, 1H, H-triazole), 7.54–7.44 (m, 4H, H-7, H-4, H-Ph), 7.36 (d, *J* = 3.1 Hz, 1H, H-2), 7.21 (d, *J* = 8.4 Hz, 2H, H-Ph), 7.10–7.02 (t, *J* = 7.0 Hz, 1H, H-6), 6.95 (t, *J* = 7.0 Hz, 1H, H-5), 6.37 (dd, *J* = 3.1, 0.7 Hz, 1H, H-3), 5.70 (t, *J* = 5.8 Hz, 1H, CHCH_2_), 5.39 (s, 2H, CH_2_), 5.23 (t, *J* = 5.3 Hz, 1H, OH), 4.18–4.08 (m, 1H, CH_A_-OH), 3.92–3.85 (m, 1H, CH_B_-OH).^13^C NMR (101 MHz, DMSO) *δ*/ppm: 143.86, 137.21, 136.04, 132.00, 129.99, 129.07, 128.67, 123.66, 121.99, 121.58, 120.88, 119.60, 110.55, 101.52, 65.54, 63.34, 41.28. Anal. calcd. For C_19_H_17_BrN_4_O (Mr = 397.28): C, 57.44; H, 4.31; N, 14.10; found: C, 57.38; H, 4.37; N, 14.17.

**1-((1-(2-Hydroxy-1-(4-(trifluoromethyl)phenyl)ethyl)-1*H*-1,2,3-triazol-4-yl)methyl)-1*H*-indole** **(30a)**

Compound **30a** was prepared using the procedure mentioned above from compound **6** (50 mg, 0.32 mmol) and 2-azido-2-(4-(trifluoromethyl)phenyl)ethanol **20a** (88 mg, 0.38 mmol). After purification via column chromatography (CH_2_Cl_2_/CH_3_OH = 50:1), compound **30a** was isolated as a yellow oil (93 mg, 76%). ^1^H NMR (600 MHz, DMSO) *δ*/ppm: 8.24 (s, 1H, H-triazole), 7.71 (d, *J* = 8.2 Hz, 2H, H-Ph, H-7), 7.58 (dd, *J* = 8.2, 0.8 Hz, 1H, H-4), 7.54–7.51 (m, 3H, H-Ph), 7.43 (d, *J* = 3.2 Hz, 1H, H-2), 7.12 (ddd, *J* = 8.2, 7.0, 1.1 Hz, 1H, H-6), 7.01 (ddd, *J* = 8.0, 7.0, 0.9 Hz, 1H, H-5), 6.44 (dd, *J* = 3.2, 0.8 Hz, 1H, H-3), 5.90 (dd, *J* = 8.3, 5.0 Hz, 1H, CHCH_2_), 5.47 (s, 2H, CH_2_), 5.34 (t, *J* = 5.3 Hz, 1H, OH), 4.25–4.21 (m, 1H, CH_A_-OH), 4.03–3.99 (m, 1H, CH_B_-OH). ^13^C NMR (151 MHz, DMSO) *δ*/ppm: 143.44, 141.87, 135.54, 128.82, 128.57, 128.40, 128.17, 128.14, 125.86 (q, *J* = 273.8 Hz), 125.49 (q, *J* = 3.6 Hz), 123.36, 121.07, 120.37, 119.08, 110.04, 100.95, 65.19, 62.81. Anal. calcd. For C_20_H_17_F_3_N_4_O (Mr = 386.38) C, 62.17; H, 4.44; N, 14.50; found: C, 62.23; H, 4.54; N, 14.59.

**1-((1-(2-Hydroxy-1-phenylethyl)-1*H*-1,2,3-triazol-4-yl)methyl)-1*H*-benzimidazole (31a)**

Compound **31a** was prepared using the procedure mentioned above from compound **7** (50 mg, 0.32 mmol) and 2-azido-2-phenylethanol **16a** (62 mg, 0.38 mmol). After purification via column chromatography (CH_2_Cl_2_/CH_3_OH = 30:1), compound **31a** was isolated as a white powder (77 mg, 75%, m.p = 214–215 °C). ^1^H NMR (400 MHz, DMSO) *δ*/ppm: 8.35 (s, 1H, H-2), 8.32 (s, 1H, H-triazole), 7.67–7.64 (m, 1H, H-4/H-7), 7.64–7.62 (m, 1H, H-4/H-7), 7.34–7.29 (m, 5H, H-Ph), 7.25–7.17 (m, 2H, H-5, H-6), 5.79–5.76 (m, 1H, CHCH_2_), 5.57 (s, 2H, CH_2_), 5.28 (t, *J* = 5.4 Hz, 1H, OH), 4.28–4.21 (m, 1H, CH_A_-OH), 3.99–3.94 (m, 1H, CH_B_-OH). ^13^C NMR (101 MHz, DMSO) *δ*/ppm: 144.40, 143.93, 142.77, 137.74, 134.05, 129.09, 128.65, 127.60, 123.88, 122.79, 122.06, 119.90, 111.18, 66.62, 63.53, a signal assigned to the CH_2_ group is obscured by the DMSO signal. Anal. calcd. For C_18_H_17_N_5_O (Mr = 319.37) C, 67.70; H, 5.37; N, 21.93; found: C, 67.79; H, 5.33; N, 21.84.

**1-((1-(2-Hydroxy-1-(4-fluorophenyl)ethyl)-1*H*-1,2,3-triazol-4-yl)methyl)-1*H*-benzimidazole (32a)**

Compound **32a** was prepared using the procedure mentioned above from compound **7** (50 mg, 0.32 mmol) and 2-azido-2-(4-fluorophenyl)ethanol **17a** (69 mg, 0.38 mmol). After purification via column chromatography (CH_2_Cl_2_/CH_3_OH = 50:1), compound **32a** was isolated as a white powder (20 mg, 19%, m.p. = 190–192 °C). ^1^H NMR (600 MHz, DMSO) *δ*/ppm: 8.32 (s, 1H, H-2/H-triazole), 8.31 (s, 1H, H-2/H-triazole), 7.65–7.62 (m, 2H, H-4, H-7), 7.40–7.37 (m, 2H, H-Ph), 7.23 (d, *J* = 8.2 Hz, 1H, H-5/H-6), 7.18 (dd, *J* = 14.1, 5.2 Hz, 3H, H-5/H-6, H-Ph), 5.79 (dd, *J* = 8.7, 4.9 Hz, 1H, CHCH_2_), 5.56 (s, 2H, CH_2_), 5.28 (t, *J* = 5.3 Hz, 1H, OH), 4.24–4.19 (m, 1H, CH_A_-OH), 3.97–3.93 (m, 1H, CH_B_-OH). ^13^C NMR (151 MHz, DMSO) *δ*/ppm: 161.86 (d, *J* = 244.5 Hz), 144.93, 143.95, 143.46, 142.33, 133.59, 133.51 (d, *J* = 3.2 Hz), 129.46 (d, *J* = 8.5 Hz), 123.41, 122.34, 121.59, 119.43, 115.44 (d, *J* = 21.7 Hz), 110.70, 65.24, 62.99. Anal. calcd. For C_18_H_16_FN_5_O (Mr = 337.36) C, 64.09; H, 4.78; N, 20.76; found: C, 64.12; H, 4.82; N, 20.80.

**1-((1-(2-Hydroxy-1-(4-chlorophenyl)ethyl)-1*H*-1,2,3-triazol-4-yl)methyl)-1*H*-benzimidazole (33a)**

Compound **33a** was prepared using the procedure mentioned above from compound **7** (50 mg, 0.32 mmol) and 2-azido-2-(4-chlorophenyl)ethanol **18a** (75 mg, 0.38 mmol). After purification via column chromatography (CH_2_Cl_2_/CH_3_OH = 30:1), compound **33a** was isolated as a white powder (28 mg, 25%, m.p. = 174–176 °C). ^1^H NMR (400 MHz, DMSO) *δ*/ppm: 8.33 (s, 1H, H-triazole/H-2), 8.32 (s, 1H, H-triazole/H-2), 7.65 (d, *J* = 4.0 Hz, 1H, H-4/H-7), 7.63 (d, *J* = 4.1 Hz, 1H, H-4/H-7), 7.43–7.40 (m, 2H, H-Ph), 7.36–7.33 (m, 2H, H-Ph), 7.27–7.22 (m, 1H, H-5/H-6), 7.22–7.17 (m, 1H, H-5/H-6), 5.80–5.78 (m, 1H, CHCH_2_), 5.57 (s, 2H, CH_2_), 5.31 (t, *J* = 5.3 Hz, 1H, OH), 4.24–4.18 (m, 1H, CH_A_-OH), 3.99–3.93 (m, 1H, CH_B_-OH). ^13^C NMR (101 MHz, DMSO) *δ* 144.21, 143.95, 142.83, 142.70, 136.70, 133.43, 129.67, 129.07, 124.00, 122.82, 122.07, 119.92, 111.18, 65.67, 63.36, a signal assigned to the CH_2_ group is obscured by the DMSO signal. Anal. calcd. For C_18_H_16_ClN_5_O (Mr = 353.81) C, 61.11; H, 4.56; N, 19.79; found: C, 61.03; H, 4.63; N, 19.87.

**1-((1-(2-Hydroxy-1-(4-bromophenyl)ethyl)-1*H*-1,2,3-triazol-4-yl)methyl)-1*H*-benzimidazole (34a)**

Compound **34a** was prepared using the procedure mentioned above from compound **7** (50 mg, 0.32 mmol) and 2-azido-2-(4-bromophenyl)ethanol **19a** (93 mg, 0.38 mmol). After purification via column chromatography (CH_2_Cl_2_/CH_3_OH = 50:1), compound **34a** was isolated as a white powder (80 mg, 63%, m.p. = 203–205 °C). ^1^H NMR (300 MHz, DMSO) *δ*/ppm: 8.26 (s, 1H, H-triazole/H-2), 8.25 (s, 1H, H-triazole/H-2), 7.60–7.55 (m, 2H, H-4, H-7), 7.48 (d, *J* = 8.4 Hz, 2H, H-Ph), 7.23–7.11 (m, 4H, H-Ph, H-5, H-6), 5.72 (dd, *J* = 8.6, 5.1 Hz, 1H, CHCH_2_), 5.51 (s, 2H, CH_2_), 5.25 (t, *J* = 5.3 Hz, 1H, OH), 4.14 (ddd, *J* = 11.3, 8.4, 5.6 Hz, 1H, CH_A_-OH), 3.90 (dt, *J* = 11.0, 4.7 Hz, 1H, CH_B_-OH). ^13^C NMR (151 MHz, DMSO) *δ*/ppm: 144.68, 144.21, 143.11, 137.39, 134.35, 132.27, 130.25, 124.28, 123.06, 122.34, 122.29, 120.18, 111.44, 66.00, 63.58, a signal assigned to the CH_2_ group is obscured by the DMSO signal. Anal. calcd. For C_18_H_16_BrN_5_O (Mr = 398.26) C, 54.29; H, 4.05; N, 17.59; found: C, 54.34; H, 4.01; N, 17.66.

**1-((1-(2-Hydroxy-1-(4-(trifluoromethyl)phenyl)ethyl)-1*H*-1,2,3-triazol-4-yl)methyl)-1*H*-benzimidazole (35a)**

Compound **35a** was prepared using the procedure mentioned above from compound **7** (50 mg, 0.32 mmol) and 2-azido-2-(4-(trifluoromethyl)phenyl)ethanol **20a** (88 mg, 0.38 mmol). After purification via column chromatography (CH_2_Cl_2_/CH_3_OH = 50:1), compound **35a** was isolated as a white powder (45 mg, 36%, m.p. = 71–73 °C). ^1^H NMR (600 MHz, DMSO) *δ*/ppm: 8.36 (s, 1H, H-triazole), 8.33 (s, 1H, H-2), 7.72 (d, *J* = 8.2 Hz, 2H, H-Ph), 7.64 (d, *J* = 8.0 Hz, 2H, H-4, H-7), 7.52 (d, *J* = 8.2 Hz, 2H, H-Ph), 7.24 (t, *J* = 7.5 Hz, 1H, H-5/H-6), 7.19 (dd, *J* = 10.9, 4.1 Hz, 1H, H-5/H-6), 5.91 (dd, *J* = 8.2, 5.0 Hz, 1H, CHCH_2_), 5.58 (s, 2H, CH_2_), 5.36 (t, *J* = 5.3 Hz, 1H, OH), 4.26–4.22 (m, 1H, CH_A_-OH), 3.93–3.86 (m, *J* = 11.5, 5.0 Hz, 1H, CH_B_-OH). ^13^C NMR (151 MHz, DMSO) *δ*/ppm: 142.39, 141.77, 128.83, 128.51 (q, *J_CF3_* = 34.2 Hz), 128.41, 128.26, 128.13, 125.49 (q, *J_CF3_* = 3.7 Hz), 125.47 (q, *J_CF3_* = 277.8 Hz), 122.32, 123.10 (q, *J_CF3_* = 267.1 Hz), 121.57, 119.40, 110.68, 65.25, 62.77, a signal assigned to the CH_2_ group is obscured by the DMSO signal. Anal. calcd. For C_19_H_16_F_3_N_5_O (Mr = 387.37) C, 58.91; H, 4.16; N, 18.08; found: C, 58.88; H, 4.09; N, 18.11.

**6-Chloro-9-((1-(2-hydroxy-1-phenylethyl)-1*H*-1,2,3-triazol-4-yl)methyl)-9*H*-purine** **(36a)**

Compound **36a** was prepared using the abovementioned procedure from compound **8** (50 mg, 0.26 mmol) and 2-azido-2-phenylethanol **16a** (51 mg, 0.31 mmol). After purification via column chromatography (CH_2_Cl_2_/CH_3_OH = 50:1), compound **36a** was isolated as a white powder (70 mg, 76%, m.p. = 151−153 °C). ^1^H NMR (600 MHz, DMSO) *δ*/ppm: 8.80 (s, 1H, H-2), 8.79 (s, 1H, H-8), 8.35 (s, 1H, H-triazole), 7.34–7.31 (m, 5H, H-Ph), 5.78–5.73 (m, 1H, CHCH_2_), 5.63 (s, 2H, CH_2_), 5.26 (t, *J* = 5.4 Hz, 1H, OH), 4.26–4.21 (m, 1H, CH_A_-OH), 3.98–3.94 (m, 1H, CH_B_-OH). ^13^C NMR (151 MHz, DMSO) *δ*/ppm: 151.70, 151.64, 149.05, 141.65, 141.50, 137.14, 130.72, 128.59, 128.22, 127.12, 123.32, 66.24, 63.02, a signal assigned to the CH_2_ group is obscured by the DMSO signal. Anal. calcd. For C_16_H_14_ClN_7_O (Mr = 355.79) C, 54.01; H, 3.97; N, 27.56; found: C, 54.11; H, 3.89; N, 27.49.

**6-Chloro-9-((1-(2-hydroxy-1-(4-fluorophenyl)ethyl)-1*H*-1,2,3-triazol-4-yl)methyl)-9*H*-purine (37a)**

Compound **37a** was prepared using the procedure mentioned above from compound **8** (50 mg, 0.26 mmol) and 2-azido-2-(4-fluorophenyl)ethanol **17a** (56 mg, 0.31 mmol). After purification via column chromatography (CH_2_Cl_2_/CH_3_OH = 50:1), compound **37a** was isolated as a white powder (77 mg, 79%, m.p. = 90–91 °C). ^1^H NMR (400 MHz, DMSO) *δ*/ppm: 8.80 (s, 2H, H-2, H-8), 8.34 (s, 1H, H-triazole), 7.42–7.38 (m, 2H, H-Ph), 7.21–7.17 (m, 2H, H-Ph), 5.78–5.81 (m, 1H, CHCH_2_), 5.64 (s, 2H, CH_2_), 5.28 (t, *J* = 5.4 Hz, 1H, OH), 4.25–4.18 (m, 1H, CH_A_-OH), 3.98–3.92 (m, 1H, CH_B_-OH). ^13^C NMR (101 MHz, DMSO) *δ*/ppm: 161.47 (d, *J_CF_* = 242.9 Hz), 151.67, 149.08, 147.35, 141.28, 138.04, 130.74, 127.98 (d, *J_CF_* = 8.1 Hz), 127.43, 124.46, 114.82 (d, *J* = 21.3 Hz), 70.55, 56.47, a signal assigned to the CH_2_ group is obscured by the DMSO signal. Anal. calcd. For C_16_H_13_ClFN_7_O (Mr = 373.78) C, 51.41; H, 3.51; N, 26.23; found: C, 54.38; H, 3.48; N, 26.18.

**6-Chloro-9-((1-(2-hydroxy-1-(4-chlorophenyl)ethyl)-1*H*-1,2,3-triazol-4-yl)methyl)-9*H*-purine (38a)**

Compound **38a** was prepared using the procedure mentioned above from compound **8** (50 mg, 0.26 mmol) and 2-azido-2-(4-chlorophenyl)ethanol **18a** (61 mg, 0.31 mmol). After purification via column chromatography (CH_2_Cl_2_/CH_3_OH = 50:1), compound **38a** was isolated as a white powder (52 mg, 51%, m.p. = 98−100 °C). ^1^H NMR (400 MHz, DMSO) *δ*/ppm: 8.80 (s, 2H, H-2, H-8), 8.34 (s, 1H, H-triazole), 7.44–7.41 (m, 2H, H-Ph), 7.36–7.34 (m, 2H, H-Ph), 5.82–5.78 (m, 1H, CHCH_2_), 5.64 (s, 2H, CH_2_), 5.30 (t, *J* = 5.3 Hz, 1H, OH), 4.24–4.17 (m, 1H, CH_A_-OH), 3.99–3.93 (m, 1H, CH_B_-OH). ^13^C NMR (101 MHz, DMSO) *δ*/ppm: 152.22, 152.17, 149.57, 147.95, 142.06, 136.62, 133.46, 131.23, 129.68, 129.09, 123.98, 65.80, 63.36, a signal assigned to the CH_2_ group is obscured by the DMSO signal. Anal. calcd. For C_16_H_13_Cl_2_N_7_O (Mr = 390.23) C, 49.25; H, 3.36; N, 25.13; found: C, 49.19; H, 3.42; N, 25.08.

**6-Chloro-9-((1-(2-hydroxy-1-(4-bromophenyl)ethyl)-1*H*-1,2,3-triazol-4-yl)methyl)-9*H*-purine (39a)**

Compound **39a** was prepared using the procedure mentioned above from compound **8** (50 mg, 0.26 mmol) and 2-azido-2-(4-bromophenyl)ethanol **19a** (75 mg, 0.31 mmol). After purification via column chromatography (CH_2_Cl_2_/CH_3_OH = 50:1), compound **39a** was isolated as a white powder (32 mg, 28%, m.p. = 98−101 °C). ^1^H NMR (400 MHz, DMSO) *δ*/ppm: 8.80 (s, 2H, H-2, H-8), 8.34 (s, 1H, H-triazole), 7.56 (d, *J* = 8.5 Hz, 2H, H-Ph), 7.29 (d, *J* = 8.5 Hz, 2H, H-Ph), 5.78 (m, 1H, CHCH_2_), 5.64 (s, 2H, CH_2_), 5.30 (t, *J* = 5.4 Hz, 1H, OH), 4.24–4.16 (m, 1H, CH_A_-OH), 3.99–3.93 (m, 1H, CH_B_-OH). ^13^C NMR (101 MHz, DMSO) *δ*/ppm: 152.21, 152.17, 149.57, 148.03, 142.05, 137.03, 132.01, 131.22, 129.99, 123.98, 122.04, 65.85, 63.30, a signal assigned to the CH_2_ group is obscured by the DMSO signal. Anal. calcd. For C_16_H_13_BrClN_7_O (Mr = 434.68) C, 44.21; H, 3.01; N, 22.56; found: C, 44.16; H, 3.07; N, 22.62.

**6-Chloro-9-((1-(2-hydroxy-1-(4-(trifluoromethyl)phenyl)ethyl)-1*H*-1,2,3-triazol-4-yl)methyl)-9*H*-purine (40a)**

Compound **40a** was prepared using the procedure mentioned above from compound **8** (50 mg, 0.26 mmol) and 2-azido-2-(4-(trifluoromethyl)phenyl)ethanol **20a** (72 mg, 0.31 mmol). After purification via column chromatography (CH_2_Cl_2_/CH_3_OH = 50:1), compound **30a** was isolated as a colorless oil (40 mg, 36%). ^1^H NMR (600 MHz, DMSO) *δ*/ppm: 8.79 (s, 2H, H-2, H-8), 8.37 (s, 1H, H-triazole), 7.73 (d, *J* = 8.1 Hz, 2H, H-Ph), 7.53 (d, *J* = 8.1 Hz, 2H, H-Ph), 5.91 (dd, *J* = 8.1, 5.0 Hz, 1H, CHCH_2_), 5.64 (d, *J* = 6.3 Hz, 2H, CH_2_), 5.35 (t, *J* = 5.3 Hz, 1H, OH), 4.25–4.21 (m, 1H, CH_A_-OH), 4.03–4.00 (m, 1H, CH_B_-OH). ^13^C NMR (151 MHz, DMSO) *δ*/ppm: 151.71, 151.66, 149.06, 141.68, 141.61, 130.72, 128.75 (q, *J* = 32.1 Hz), 128.14, 126.70, 125.50 (q, *J* = 3.2 Hz), 124.00 (q, *J* = 272.4 Hz), 123.70, 65.37, 62.77, a signal assigned to the CH_2_ group is obscured by the DMSO signal. Anal. calcd. For C_17_H_13_ClF_3_N_7_O (Mr = 423.78) C, 48.18; H, 3.09; N, 23.14; found: C, 48.26; H, 3.15; N, 23.17.

**4-Chloro-7-((1-(2-hydroxy-1-phenylethyl)-1*H*-1,2,3-triazol-4-yl)methyl)-7*H*-pyrrolo[2,3-*d*]pyrimidine (41a)**

Compound **41a** was prepared using the procedure mentioned above from compound **9** (50 mg, 0.26 mmol) and 2-azido-2-phenylethanol **16a** (51 mg, 0.31 mmol). After purification via column chromatography (CH_2_Cl_2_/CH_3_OH = 50:1), compound **41a** was isolated as a white powder (75 mg, 81%, m.p. = 139–141 °C). ^1^H NMR (600 MHz, DMSO) *δ*/ppm: 8.66 (s, 1H, H-2), 8.28 (s, 1H, H-triazole), 7.81 (d, *J* = 3.6 Hz, 1H, H-6), 7.31–7.35 (m, 5H, H-Ph), 6.68 (d, *J* = 3.6 Hz, 1H, d, H-5), 5.75 (m, 1H, CHCH_2_), 5.59 (s, 2H, CH_2_), 5.26 (t, *J* = 5.2 Hz, 1H, OH), 4.26–4.22 (m, 1H, CH_A_-OH), 3.97–3.92 (m, 1H, CH_B_-OH) ^13^C NMR (151 MHz, DMSO) *δ*/ppm: 151.19, 150.90, 150.90, 142.83, 137.72, 131.80, 129.13, 128.76, 127.66, 123.72, 117.25, 99.39, 66.72, 63.54, a signal assigned to the CH_2_ group is obscured by the DMSO signal. Anal. calcd. For C_17_H_15_ClN_6_O (Mr = 354.80) C, 57.55; H, 4.26; N, 23.69; found C, 57.61; H, 4.19; N, 23.76.

**4-Chloro-7-((1-(2-hydroxy-1-(4-fluorophenyl)ethyl)-1*H*-1,2,3-triazol-4-yl)methyl)-7*H*-pyrrolo[2,3-*d*]pyrimidine (42a)**

Compound **42a** was prepared using the procedure mentioned above from compound **9** (50 mg, 0.26 mmol) and 2-azido-2-(4-fluorophenyl)ethanol **17a** (56 mg, 0.31 mmol). After purification via column chromatography (CH_2_Cl_2_/CH_3_OH = 50:1), compound **42a** was isolated as a white powder (73 mg, 75%, m.p. = 90–91 °C). ^1^H NMR (600 MHz, DMSO) *δ*/ppm: 8.67 (s, 1H, H-2), 8.26 (s, 1H, H-triazole), 7.81 (d, *J* = 3.6 Hz, 1H, H-6), 7.39 (m, 2H, H-Ph), 7.17 (d, *J* = 8.9 Hz, 2H, H-Ph), 6.68 (d, *J* = 3.6 Hz, 1H, H-5), 5.76–5.78 (m, 1H, CHCH_2_), 5.58 (s, 2H, CH_2_), 5.27 (t, *J* = 5.2 Hz, 1H, OH), 4.26–4.21 (m, 1H, CH_A_-OH), 3.96–3.92 (m, 1H, CH_B_-OH). ^13^C NMR (151 MHz, DMSO) *δ*/ppm: 162.06 (d, *J_CF_* = 244.6 Hz), 150.87, 150.62, 150.59, 142.55, 133.69 (d, *J_CF_* = 2.9 Hz), 131.49, 129.67 (d, *J_CF_* = 8.4 Hz), 123.41, 116.94, 115.63 (d, *J_CF_* = 21.4 Hz), 99.07, 65.49, 63.18, a signal assigned to the CH_2_ group is obscured by the DMSO signal. Anal. calcd. For C_17_H_14_ClFN_6_O (Mr = 372.79) C, 54.77; H, 3.79; N, 22.54; found C, 54.72; H, 3.83; N, 22.61.

**4-Chloro-7-((1-(2-hydroxy-1-(4-chlorophenyl)ethyl)-1*H*-1,2,3-triazol-4-yl)methyl)-7*H*-pyrrolo[2,3-*d*]pyrimidine (43a)**

Compound **43a** was prepared using the procedure mentioned above from compound **9** (50 mg, 0.26 mmol) and 2-azido-2-(4-chlorophenyl)ethanol **18a** (62 mg, 0.31 mmol). After purification via column chromatography (CH_2_Cl_2_/CH_3_OH = 50:1), compound **43a** was isolated as a white powder (83 mg, 82%, m.p. = 149–150 °C). ^1^H NMR (400 MHz, DMSO) *δ*/ppm: 8.65 (s, 1H, H-2), 8.25 (s, 1H, H-triazole), 7.80 (d, *J* = 3.6 Hz, 1H, H-6), 7.39 (s, 2H, H-Ph), 7.34 (d, 2H, H-Ph), 6.67 (d, *J* = 3.6 Hz, 1H, H-5), 5.79–5.75 (m, 1H, CHCH_2_), 5.58 (s, 2H, CH_2_), 5.28 (t, *J* = 5.2 Hz, 1H, OH), 4.23–4.16 (m, 1H, CH_A_-OH), 3.97–3.93 (m, 1H, CH_B_-OH). ^13^C NMR (101 MHz, DMSO) *δ*/ppm: 151.16, 150.91, 150.87, 142.88, 136.68, 133.45, 131.78, 129.69, 129.09, 123.82, 117.23, 99.37, 65.74, 63.35, a signal assigned to the CH_2_ group is obscured by the DMSO signal. Anal. calcd. For C_17_H_14_Cl_2_N_6_O (Mr = 389.24) C, 52.46; H, 3.63; N, 21.59; found C, 52.51; H, 3.71; N, 21.67.

**4-Chloro-7-((1-(2-hydroxy-1-(4-bromophenyl)ethyl)-1*H*-1,2,3-triazol-4-yl)methyl)-7*H*-pyrrolo[2,3-*d*]pyrimidine (44a)**

Compound **44a** was prepared using the procedure mentioned above from compound **9** (50 mg, 0.26 mmol) and 2-azido-2-(4-bromophenyl)ethanol **19a** (75 mg, 0.31 mmol). After purification via column chromatography (CH_2_Cl_2_/CH_3_OH = 50:1), compound **44a** was isolated as a white powder (39 mg, 35%, m.p. = 211–213 °C). ^1^H NMR (400 MHz, DMSO) *δ*/ppm: 8.66 (s, 1H, H-2), 8.26 (s, 1H, H-triazole), 7.81 (d, *J* = 3.6 Hz, 1H, H-6), 7.55 (d, *J* = 8.5 Hz, 2H, H-Ph), 7.28 (d, *J* = 8.5 Hz, 2H, H-Ph), 6.68 (d, *J* = 3.6 Hz, 1H, H-5), 5.78–5.74 (m, 1H, CHCH_2_), 5.58 (s, 2H, CH_2_), 5.29 (t, *J* = 5.3 Hz, 1H, OH), 4.23–4.17 (m, 1H, CH_A_-OH), 3.97–3.92 (m, 1H, CH_B_-OH). ^13^C NMR (151 MHz, DMSO) *δ*/ppm: 150.67, 150.44, 150.39, 142.40, 136.61, 131.54, 131.32, 129.52, 123.34, 121.55, 116.74, 98.88, 65.30, 62.80, a signal assigned to the CH_2_ group is obscured by the DMSO signal. Anal. calcd. For C_17_H_14_BrClN_6_O (Mr = 433.69) C, 47.08; H, 3.25; N, 19.38; found C, 47.13; H, 3.29; N, 19.44.

**4-Chloro-7-((1-(2-hydroxy-1** **-(4-(trifluoromethyl)phenyl)** **ethyl)-1*H*-1,2,3-triazol-4-yl)methyl)-7*H*-pyrrolo[2,3-*d*]pyrimidine (45a)**

Compound **45a** was prepared using the procedure mentioned above from compound **9** (50 mg, 0.26 mmol) and 2-azido-2-(4-(trifluoromethyl)phenyl)ethanol **20a** (72 mg, 0.31 mmol). After purification via column chromatography (CH_2_Cl_2_/CH_3_OH = 50:1), compound **45a** was isolated as a colorless oil (38 mg, 35%). ^1^H NMR (400 MHz, DMSO) *δ*/ppm: 8.66 (s, 1H, H-2), 8.26 (s, 1H, H-triazole), 7.81 (d, *J* = 3.6 Hz, 1H, H-6), 7.40 (d, *J* = 8.6 Hz, 2H, H-Ph), 7.35 (d, *J* = 8.6 Hz, 2H, H-Ph), 6.68 (d, *J* = 3.6 Hz, 1H, H-5), 5.78 (dd, *J* = 8.7, 5.0 Hz, 1H, CHCH_2_), 5.59 (s, 2H, CH_2_), 5.29 (t, *J* = 5.6 Hz, 1H, OH), 4.24–4.19 (m, CH_A_-OH, 1H), 3.98–3.92 (m, 1H, CH_B_-OH). ^13^C NMR (151 MHz, DMSO) *δ*/ppm: 151.14, 150.88, 150.81, 147.02, 142.72, 131.67, 128.55 (q, *J*_CF3_ = 31.5 Hz), 127.29, 125.41 (q, *J*_CF3_ = 3.6 Hz), 124.77, 124.69 (q, *J*_CF3_ = 272.2 Hz), 117.22, 99.26, 71.10, 56.61, a signal assigned to the CH_2_ group is obscured by the DMSO signal. Anal. calcd. For C_18_H_14_ClF_3_N_6_O (Mr = 422.80) C, 51.14; H, 3.34; N, 19.88; found C, 51.09; H, 3.39; N, 19.81.

**4-Chloro-1-((1-(2-hydroxy-1-phenylethyl)-1*H*-1,2,3-triazol-4-yl)methyl)-1*H*-imidazo[4,5-*c*]pyridine (46a)**

Compound **46a** was prepared using the procedure mentioned above from compound **10** (50 mg, 0.26 mmol) and 2-azido-2-phenylethanol **16a** (51 mg, 0.31 mmol). After purification via column chromatography (CH_2_Cl_2_/CH_3_OH = 50:1), compound **46a** was isolated as a white powder (59 mg, 64%, m.p. = 92–94 °C). ^1^H NMR (600 MHz, DMSO) *δ*/ppm: 8.58 (s, 1H, H-2), 8.37 (s, 1H, H-triazole), 8.15 (d, *J* = 5.6 Hz, 1H, H-6), 7.74 (d, *J* = 5.6 Hz, 1H, H-7), 7.35–7.29 (m, 5H, H-Ph), 5.80–5.74 (m, 1H, CHCH_2_), 5.65 (s, 2H, CH_2_), 5.27 (t, *J* = 5.7 Hz, 1H, OH), 4.25–4.21 (m, 1H, CH_A_-OH), 3.98–3.94 (m, 1H, CH_B_-OH). ^13^C NMR (75 MHz, DMSO) *δ*/ppm: 143.59, 142.33, 141.66, 140.74, 137.75, 129.47, 129.15, 127.81, 126.69, 124.30, 107.74, 63.75, a signal assigned to the CH_2_ group is obscured by the DMSO signal, and signals of two quaternary C atoms were not observed. Anal. calcd. For C_17_H_15_ClN_6_O (Mr = 354.80) C, 57.55; H, 4.26; N, 23.69; found C, 57.61; H, 4.32; N, 23.61.

**4-Chloro-1-((1-(2-hydroxy-1-(4-fluorophenyl)ethyl)-1*H*-1,2,3-triazol-4-yl)methyl)-1*H*-imidazo[4,5-*c*]pyridine (47a)**

Compound **47a** was prepared using the procedure mentioned above from compound **10** (50 mg, 0.26 mmol) and 2-azido-2-(4-fluorophenyl)ethanol **17a** (56 mg, 0.31 mmol). After purification via column chromatography (CH_2_Cl_2_/CH_3_OH = 50:1), compound **47a** was isolated as a white powder (60 mg, 62%, m.p. = 100–101 °C). ^1^H NMR (400 MHz, DMSO) *δ*/ppm: 8.68 (s, 1H, H-2), 8.31 (s, 1H, H-triazole), 8.15 (d, *J* = 5.5 Hz, 1H, H-6), 7.73 (d, *J* = 5.5 Hz, 1H, H-7), 7.41–7.37 (m, 2H, H-Ph), 7.21–7.16 (m, 2H, H-Ph), 5.85 (s, 2H, CH_2_), 5.81–5.76 (m, 1H, CHCH_2_), 5.28 (t, *J* = 5.3 Hz, 1H, OH), 4.29–4.26 (m, 1H, CH_A_-OH), 3.98–3.92 (m, 1H, CH_B_-OH). ^13^C NMR (75 MHz, DMSO) *δ*/ppm: 162.34 (d, *J_CF_* = 244.8 Hz), 151.42, 145.10, 143.32, 141.16, 134.00 (d, *J_CF_* = 2.9 Hz), 129.89 (d, *J*_CF_ = 8.3 Hz), 123.38, 116.04, 115.75 (d, *J_CF_* = 21.5 Hz), 65.80, 63.47, 41.75, a signal assigned to the CH_2_ group is obscured by the DMSO signal. Anal. calcd. For C_17_H_14_ClFN_6_O (Mr = 372.79) C, 54.77; H, 3.79; N, 22.54; found C, 54.71; H, 3.72; N, 22.49.

**4-Chloro-1-((1-(2-hydroxy-1-(4-chlorophenyl)ethyl)-1*H*-1,2,3-triazol-4-yl)methyl)-1*H*-imidazo[4,5-*c*]pyridine (48a)**

Compound **48a** was prepared using the procedure mentioned above from compound **10** (50 mg, 0.26 mmol) and 2-azido-2-(4-chlorophenyl)ethanol **18a** (62 mg, 0.31 mmol). After purification via column chromatography (CH_2_Cl_2_/CH_3_OH = 50:1), compound **48a** was isolated as a white powder (47 mg, 46%, m.p. = 108–110 °C). ^1^H NMR (400 MHz, DMSO) *δ*/ppm: 8.58 (s, 1H, H-2), 8.36 (s, 1H, H-triazole), 8.16 (d, *J* = 5.6 Hz, 1H, H-6), 7.75 (d, *J* = 5.6 Hz, 1H, H-7), 7.42 (d, *J* = 8.5 Hz, 2H, H-Ph), 7.34 (d, *J* = 8.6 Hz, 2H, H-Ph), 5.81 (dd, *J* = 8.5, 4.9 Hz, 1H, CHCH_2_), 5.66 (s, 2H, CH_2_), 5.31 (t, *J* = 5.3 Hz, 1H, OH), 4.24–4.17 (m, 1H CH_A_-OH), 3.99–3.93 (m, 1H, CH_B_-OH). ^13^C NMR (101 MHz, DMSO) *δ*/ppm: 146.71, 142.15, 141.42, 141.32, 140.50, 137.55, 136.61, 133.46, 129.67, 129.09, 124.11, 107.44, 65.74, 63.34. Anal. calcd. For C_17_H_14_Cl_2_N_6_O (Mr = 389.24) C, 52.46; H, 3.63; N, 21.59; found C, 52.41; H, 3.59; N, 21.52.

**4-Chloro-1-((1-(2-hydroxy-1-(4-bromophenyl)ethyl)-1*H*-1,2,3-triazol-4-yl)methyl)-1*H*-imidazo[4,5-*c*]pyridine (49a)**

Compound **49a** was prepared using the procedure mentioned above from compound **10** (50 mg, 0.26 mmol) and 2-azido-2-(4-bromophenyl)ethanol **19a** (75 mg, 0.31 mmol). After purification via column chromatography (CH_2_Cl_2_/CH_3_OH = 50:1) compound **49a** was isolated as a white powder (41 mg, 36%, m.p. = 99–102 °C). ^1^H NMR (400 MHz, DMSO) *δ*/ppm: 8.58 (s, 1H, H-2), 8.36 (s, 1H, H-triazole), 8.16 (d, *J* = 5.6 Hz, 1H, H-6), 7.75 (d, *J* = 5.6 Hz, 1H, H-7), 7.42 (d, *J* = 8.5 Hz, 2H, H-5), 7.34 (d, *J* = 8.6 Hz, 2H, H-Ph), 5.81 (d, *J* = 8.5 Hz, 1H, H-Ph), 5.81–5.78 (m, CHCH_2_), 5.66 (s, 2H, CH_2_), 5.31 (t, *J* = 5.3 Hz, 1H, OH), 4.24–4.17 (m, 1H, CH_A_-OH), 3.99–3.94 (m, 1H, CH_B_-OH). ^13^C NMR (101 MHz, DMSO) *δ*/ppm: 146.73, 142.16, 141.44, 141.34, 140.51, 137.56, 137.04, 132.04, 130.00, 124.15, 122.07, 107.46, 65.36, 63.30, a signal assigned to the CH_2_ group is obscured by the DMSO signal. Anal. calcd. For C_17_H_14_BrClN_6_O (Mr = 433.69) C, 47.08; H, 3.25; N, 19.38; found C, 47.11; H, 3.31; N, 19.31.

**1-((1-(2-Hydroxy-2-phenylethyl)-1*H*-1,2,3-triazol-4-yl)methyl)-1*H*-indole** **(26b)**

Compound **26b** was prepared using the abovementioned procedure from compound **6** (50 mg, 0.32 mmol) and 2-azido-1-phenylethanol **16b** (62 mg, 0.38 mmol). After purification via column chromatography (CH_2_Cl_2_/CH_3_OH = 30:1), compound **26b** was isolated as a white powder (65 mg, 64%, m.p. = 119–120 °C). ^1^H NMR (400 MHz, DMSO) *δ*/ppm: 7.93 (s, 1H, H-triazole), 7.59–7.52 (m, 2H, H-4, H-7), 7.41 (d, *J* = 3.2 Hz, 1H, H-2), 7.33–7.24 (m, 5H, H-Ph), 7.16–7.14 (m, 1H, H-6), 7.05–7.01 (m, 1H, H-5), 6.44 (d, *J* = 3.1, 1H, H-3), 5.76 (d, *J* = 4.7 Hz, 1H, OH), 5.45 (s, 2H, CH_2_), 4.95–4.91 (m, 1H, CHCH_2_), 4.48–4.38 (m, 2H, CHCH_2_). ^13^C NMR (101 MHz, DMSO) *δ*/ppm: 143.64, 142.46, 136.02, 129.05, 128.70, 128.61, 127.98, 126.46, 124.42, 121.54, 120.83, 119.56, 110.59, 101.34, 71.79, 56.97, 41.28. Anal. calcd. For C_19_H_18_N_4_O (Mr = 318.38) C, 71.68; H, 5.70; N, 17.60; found C, 71.72; H, 5.68; N, 17.68.

**1-((1-(2-Hydroxy-2-(4-fluorophenyl)ethyl)-1*H*-1,2,3-triazol-4-yl)methyl)-1*H*-indole (27b)**

Compound **27b** was prepared using the procedure mentioned above from compound **6** (50 mg, 0.32 mmol) and 2-azido-1-(4-fluorophenyl)ethanol **17b** (69 mg, 0.38 mmol). After purification via column chromatography (CH_2_Cl_2_/CH_3_OH = 30:1), compound **27b** was isolated as a white powder (53 mg, 49%, m.p. = 165–167 °C). ^1^H NMR (600 MHz, DMSO) *δ*/ppm: 7.90 (s, 1H, H-triazole), 7.56–7.54 (m, 2H, H-4, H-7), 7.41 (d, *J* = 3.2 Hz, 1H, H-2), 7.32–7.30 (m, 2H, H-Ph), 7.16–7.10 (m, 1H, H-6), 7.10–7.06 (m, 2H, H-Ph), 7.05–7.00 (m, 1H, H-5), 6.46–6.43 (m, 1H, H-3), 5.81 (d, *J* = 4.7 Hz, 1H, OH), 5.44 (s, 2H, CH_2_), 4.96–4.93 (m, 1H, CHCH_2_), 4.50–4.39 (m, 2H, CHCH_2_). ^13^C NMR (75 MHz, CDCl_3_) *δ*/ppm: 157.91 (d, *J_CF_* = 246.13 Hz), 143.20, 138.34 (d, *J_CF_* = 2.9 Hz), 135.52, 128.57, 128.23, 127.96 (d, *J_CF_* = 8.3 Hz), 123.90, 121.04, 120.35, 119.08 (d, *J_CF_* = 21.3 Hz), 114.69, 110.10, 100.85, 70.59, 56.36, 40.80. Anal. calcd. For C_19_H_17_FN_4_O (Mr = 336.37) C, 67.84; H, 5.09; N, 16.66; found C, 67.79; H, 5.13; N, 16.70.

**1-((1-(2-Hydroxy-2-(4-chlorophenyl)ethyl)-1*H*-1,2,3-triazol-4-yl)methyl)-1*H*-indol (28b)**

Compound **28b** was prepared using the procedure mentioned above from compound **6** (50 mg, 0.32 mmol) and 2-azido-1-(4-chlorophenyl)ethanol **18b** (76 mg, 0.38 mmol). After purification via column chromatography (CH_2_Cl_2_/CH_3_OH = 30:1), compound **28b** was isolated as a white powder (47 mg, 42%, m.p. = 154–156 °C). ^1^H NMR (300 MHz, DMSO) *δ*/ppm: 7.90 (s, 1H, H-triazole), 7.56 (d, *J* = 3.5 Hz, 1H, H-7), 7.55 (d, *J* = 3.2 Hz, 1H, H-4), 7.40 (d, *J* = 3.1 Hz, 1H, H-2), 7.26–7.32 (m, 4H, H-Ph), 7.14 (dd, *J* = 11.2, 4.2 Hz, 1H, H-6), 7.03 (dd, *J* = 11.0, 4.1 Hz, 1H, H-5), 6.44 (d, *J* = 2.6 Hz, 1H, H-3), 5.85 (d, *J* = 4.7 Hz, 1H, OH), 5.44 (s, 2H, CH_2_), 4.96–4.92 (m, 1H, CHCH_2_), 4.45–4.42 (m, 2H, CHCH_2_). ^13^C NMR (151 MHz, DMSO) *δ*/ppm: 143.70, 141.37, 136.00, 132.45, 129.05, 128.72, 128.53, 128.34, 124.41, 121.53, 120.84, 119.56, 110.58, 101.34, 71.03, 56.69, 41.29. Anal. calcd. For C_19_H_17_ClN_4_O (Mr = 352.82) C, 64.68; H, 4.86; N, 15.88; found C, 64.62; H, 4.71; N, 15.80.

**1-((1-(2-Hydroxy-2-(4-bromophenyl)ethyl)-1*H*-1,2,3-triazol-4-yl)methyl)-1*H*-indol (29b)**

Compound **29b** was prepared using the procedure mentioned above from compound **6** (76 mg, 0.32 mmol) and 2-azido-1-(4-bromophenyl)ethanol **19b** (93 mg, 0.38 mmol). After purification via column chromatography (CH_2_Cl_2_/CH_3_OH = 30:1), compound **29b** was isolated as a white powder (62 mg, 49%, m.p. = 65–67 °C). ^1^H NMR (600 MHz, DMSO) *δ*/ppm: 7.91 (s, 1H, H-triazole), 7.54–7.56 (m, 2H, H-4, H-7), 7.45–7.43 (m, 2H, H-Ph), 7.40 (d, *J* = 3.1 Hz, 1H, H-2), 7.22 (d, *J* = 8.4 Hz, 2H, H-Ph), 7.15–7.12 (m, 1H, H-6), 7.04–7.01 (m, 1H, H-5), 6.44 (d, *J* = 3.1, 1H, H-3), 5.86 (d, *J* = 4.7 Hz, 1H, OH), 5.44 (s, 2H, CHCH_2_), 4.95–4.92 (m, 1H, CHCH_2_), 4.47–4.41 (m, 2H, CH_2_). ^13^C NMR (151 MHz, DMSO) *δ*/ppm: 143.72, 141.80, 136.01, 131.46, 129.07, 128.73, 128.71, 124.43, 121.54, 121.02, 120.85, 119.58, 110.60, 101.35, 71.08, 56.64, 41.30. Anal. calcd. For C_19_H_17_BrN_4_O (Mr = 397.28) C, 57.44; H, 4.31; N, 14.10; found C, 57.51; H, 4.27; N, 14.18.

**1-((1-(2-Hydroxy-2-(4-(trifluoromethyl)phenyl)ethyl)-1*H*-1,2,3-triazol-4-yl)methyl)-1*H*-** **indol (30b)**

Compound **30b** was prepared using the procedure mentioned above from compound **6** (76 mg, 0.32 mmol) and 2-azido-1-(4-(trifluoromethyl)phenyl)ethanol **19b** (88 mg, 0.38 mmol). After purification via column chromatography (CH_2_Cl_2_/CH_3_OH = 30:1), compound **30b** was isolated as a white powder (53 mg, 43%, m.p. = 73–75 °C). ^1^H NMR (600 MHz, DMSO) *δ*/ppm: 8.09 (s, 1H, H-triazoli), 7.62 (m, 3H, H-7, H-4, H-2), 7.61 (d, *J* = 8.2 Hz, 2H, H-Ph), 7.50 (d, *J* = 8.3 Hz, 2H, H-Ph), 7.24 (ddd, *J* = 24.5, 13.0, 7.6 Hz, 3H, H-6, H-5, H-3), 6.01 (d, *J* = 4.7 Hz, 1H, OH), 5.56 (s, 2H, CH_2_), 5.10–5.04 (m, 1H, CHCH_2_), 4.56–4.43 (m, 2H, CHCH_2_). ^13^C NMR (151 MHz, DMSO) *δ*/ppm: 147.04, 145.46, 142.70, 128.53 (q, *J* = 31.5 Hz), 127.27, 126.39 (q, *J* = 271.4 Hz), 125.44 (q, *J* = 3.7 Hz), 124.88, 123.81, 123.81, 123.24, 123.24, 122.77, 122.03, 119.91, 111.23, 71.08, 56.62. Anal. calcd. For C_20_H_17_F_3_N_4_O (Mr = 386.38) C, 62.17; H, 4.44; N, 14.50; found C, 62.21; H, 4.40; N, 14.56.

**1-((1-(2-Hydroxy-2-phenylethyl)-1*H*-1,2,3-triazol-4-yl)methyl)-1*H*-benzimidazole** **(31b)**

Compound **31b** was prepared using the procedure mentioned above from compound **7** (50 mg, 0.32 mmol) and 2-azido-1-phenylethanol **16b** (62 mg, 0.38 mmol). After purification via column chromatography (CH_2_Cl_2_/CH_3_OH = 30:1), compound **31b** was isolated as a white powder (87 mg, 85%, m.p. = 178–179 °C). ^1^H NMR (400 MHz, DMSO) *δ*/ppm: 8.30 (s, 1H, H-2), 8.06 (s, 1H, H-triazole), 7.64 (m, 2H, H-4, H-7), 7.32–7.19 (m, 7H, H-Ph, H-5, H-6, H-3), 5.78 (d, *J* = 4.7 Hz, 1H, OH), 5.56 (s, 2H, CH_2_), 4.97–4.92 (m, 1H, CHCH_2_), 4.50–4.43 (m, 2H, CHCH_2_). ^13^C NMR (101 MHz, DMSO) *δ*/ppm: 144.36, 143.97, 142.57, 142.37, 134.05, 128.74, 127.99, 126.45, 124.84, 122.79, 122.19, 119.87, 111.23, 71.62, 57.03, a signal assigned to the CH_2_ group is obscured by the DMSO signal. Anal. calcd. For C_18_H_17_N_5_O (Mr = 319.37) C, 67.70; H, 5.37; N, 21.93; found C, 67.67; H, 5.41; N, 21.81.

**1-((1-(2-Hydroxy-2-(4-fluorphenyl)ethyl)-1*H*-1,2,3-triazol-4-yl)methyl)-1*H*-benzimidazole** **(32b)**

Compound **32b** was prepared using the procedure mentioned above from compound **7** (50 mg, 0.32 mmol) and 2-azido-1-(4-fluorophenyl)ethanol **17b** (69 mg, 0.38 mmol). After purification via column chromatography (CH_2_Cl_2_/CH_3_OH = 30:1), compound **32b** was isolated as a white powder (51 mg, 47%, m.p. = 173–174 °C). ^1^H NMR (600 MHz, DMSO) *δ*/ppm: 8.30 (s, 1H, H-2), 8.03 (s, 1H, H-triazole), 7.66–7.64 (m, 1H, H-4), 7.62–7.60 (d, *J* = 7.9 Hz, 1H, H-7), 7.32–7.29 (m, 2H, H-Ph), 7.26–7.23 (m, 1H, H-5), 7.21–7.19 (m, 1H, H-6), 7.07–7.04 (m, 2H, H-Ph), 5.83 (d, J = 4.7 Hz, 1H, OH), 5.54 (s, 2H, CH_2_), 4.94–4.97 (m, 1H, CHCH_2_), 4.47–4.42 (m, 2H, CHCH_2_). ^13^C NMR (151 MHz, DMSO) *δ*/ppm: 161.61 (d, *J_CF_* = 242.8 Hz), 144.02, 143.62, 142.26, 138.18 (d, *J_CF_* = 2.3 Hz), 133.66, 128.09 (*J_CF_* = 7.8 Hz), 124.46, 122.43, 121.70, 119.52, 114.96, 110.87, 70.69, 56.56, a signal assigned to the CH_2_ group is obscured by the DMSO signal. Anal. calcd. For C_18_H_16_FN_5_O (Mr = 337.36) C, 64.09; H, 4.78; N, 20.76; found C, 64.14; H, 4.82; N, 20.69.

**1-((1-(2-Hydroxy-2-(4-chlorophenyl)ethyl)-1*H*-1,2,3-triazol-4-yl)methyl)-1*H*-benzimidazole** **(33b)**

Compound **33b** was prepared using the procedure mentioned above from compound **7** (50 mg, 0.32 mmol) and 2-azido-1-(4-chlorophenyl)ethanol **18b** (75 mg, 0.38 mmol). After purification via column chromatography (CH_2_Cl_2_/CH_3_OH = 30:1), compound **33b** was isolated as a white powder (37 mg, 33%, m.p. = 218–220 °C). ^1^H NMR (400 MHz, DMSO) *δ*/ppm: 8.32 (s, 1H, H-2), 8.05 (s, 1H, H-triazole), 7.66 (d, *J* = 7.9 Hz, 1H, H-4), 7.62 (d, *J* = 7.9 Hz, 1H, H-7), 7.29 (s, 4H, H-Ph), 7.23 (m, 1H, H-5), 7.21 (m, 1H, H-6), 5.89 (d, *J* = 4.4 Hz, 1H, OH), 5.55 (s, 2H, CH_2_), 4.97 (m, 1H, CHCH_2_), 4.47 (t, *J* = 6.0 Hz, 2H, CHCH_2_). ^13^C NMR (151 MHz, DMSO) *δ*/ppm: 144.55, 144.35, 142.92, 141.62, 134.18, 132.75, 128.82, 128.62, 125.12, 123.06, 122.33, 120.19, 111.52, 71.27, 57.04, a signal assigned to the CH_2_ group is obscured by the DMSO signal. Anal. calcd. For C_18_H_16_ClN_5_O (Mr = 353.81) C, 61.11; H, 4.56; N, 19.79; found C, 61.06; H, 4.61; N, 19.70.

**1-((1-(2-Hydroxy-2-(4-bromophenyl)ethyl)-1*H*-1,2,3-triazol-4-yl)methyl)-1*H*-benzimidazole** **(34b)**

Compound **34b** was prepared using the procedure mentioned above from compound **7** (50 mg, 0.32 mmol) and 2-azido-1-(4-bromophenyl)ethanol **19b** (93 mg, 0.38 mmol). After purification via column chromatography (CH_2_Cl_2_/CH_3_OH = 30:1), compound **34b** was isolated as a white powder (25 mg, 20%, m.p. = 224–226 °C). ^1^H NMR (400 MHz, DMSO) *δ*/ppm: 8.31 (s, 1H, H-2), 8.06 (s, 1H, H-triazole), 7.66 (d, *J* = 7.7 Hz, 1H, H-4), 7.62 (d, *J* = 7.8 Hz, 1H, H-7), 7.44 (d, *J* = 8.4 Hz, 4H, H-Ph), 7.26–7.19 (m, 4H, H-Ph, H-5, H-6), 5.89 (d, *J* = 4.7 Hz, 1H, OH), 5.56 (s, 2H, CH_2_), 4.95 (dt, *J* = 9.1, 4.6 Hz, 1H, CHCH_2_), 4.52–4.41 (m, 2H, CHCH_2_). ^13^C NMR (151 MHz, DMSO) *δ*/ppm: 144.54, 144.15, 142.79, 141.89, 134.17, 131.59, 128.84, 124.98, 122.92, 122.19, 121.17, 120.04, 111.36, 71.18, 56.84, a signal assigned to the CH_2_ group is obscured by the DMSO signal. Anal. calcd. For C_18_H_16_BrN_5_O (Mr = 398.26) C, 54.29; H, 4.05; N, 17.59; found C, 54.21; H, 4.12; N, 17.64.

**1-((1-(2-Hydroxy-2-(4-(trifluoromethyl)phenyl)ethyl)-1*H*-1,2,3-triazol-4-yl)methyl)-1*H*-benzimidazole** **(35b)**

Compound **35b** was prepared using the procedure mentioned above from compound **7** (50 mg, 0.32 mmol) and 2-azido-1-(4-(trifluoromethyl)phenyl)ethanol **20b** (88 mg, 0.38 mmol). After purification via column chromatography (CH_2_Cl_2_/CH_3_OH = 30:1), compound **35b** was isolated as a white powder (25 mg, 20%, m.p. = 208–210 °C). ^1^H NMR (600 MHz, DMSO) *δ*/ppm: 8.30 (s, 1H, H-2), 8.05 (s, 1H, H-triazole), 7.65 (d, *J* = 7.8 Hz, 1H, H-4), 7.60 (m, 1H, H-7), 7.44–7.42 (m, 2H, H-Ph), 7.21 (ddd, *J* = 9.0, 3.8, 2.0 Hz, 3H, H-Ph, H-5, H-6), 5.88 (d, *J* = 4.7 Hz, 1H, OH), 5.55 (s, 2H, CH_2_), 4.94 (dt, *J* = 7.4, 4.5 Hz, 1H, CHCH_2_), 4.46 (qd, *J* = 13.9, 6.0 Hz, 2H, CHCH_2_). ^13^C NMR (151 MHz, DMSO) *δ*/ppm: 142.90, 142.28, 129.23 (q, *J* = 31.8 Hz), 128.64, 127.27, 126.01, 124.51 (q, *J* = 271.8 Hz), 124.89, 124.23, 122.81, 122.06, 121.81, 119.96, 111.25, 65.76, 63.27, a signal assigned to the CH_2_ group is obscured by the DMSO signal. Anal. calcd. For C_19_H_16_F_3_N_5_O (Mr = 387.37) C, 58.91; H, 4.16; N, 18.08; found C, 58.86; H, 4.09; N, 18.13.

**6-Chloro** **-9** **-((1-(2-hydroxy-2-phenylethyl)-1*H*-1,2,3-triazol-4-yl)methyl)-9*H*-purine (36b)**

Compound **36b** was prepared using the abovementioned procedure from compound **8** (50 mg, 0.26 mmol) and 2-azido-1-phenylethanol **16b** (51 mg, 0.31 mmol). After purification via column chromatography (CH_2_Cl_2_/CH_3_OH = 30:1), compound **36b** was isolated as a white powder (55 mg, 60%, m.p = 129–131 °C). ^1^H NMR (400 MHz, DMSO) *δ*/ppm: 8.81 (s, 1H, H-2), 8.77 (s, 1H, H-8), 8.07 (s, 1H, H-triazole), 7.33–7.23 (m, 5H, H-Ph), 5.77 (d, *J* = 4.7 Hz, 1H, OH), 5.62 (s, 2H, CH_2_), 4.95 (dt, *J* = 8.6, 4.5 Hz, 1H, CHCH_2_), 4.46 (qd, *J* = 13.7, 6.2 Hz, 2H, CHCH_2_). ^13^C NMR (101 MHz, DMSO) *δ*/ppm: 152.18, 152.13, 149.55, 147.83, 142.34, 141.74, 131.24, 128.57, 127.97, 126.45, 124.97, 71.75, 57.07, a signal assigned to the CH_2_ group is obscured by the DMSO signal. Anal. calcd. For C_16_H_14_ClN_7_O (Mr = 355.79) C, 54.01; H, 3.97; N, 27.56; found C, 54.09; H, 3.91; N, 27.49.

**6-Chloro-9-((1-(2-hydroxy-2-(4-fluorophenyl)ethyl)-1*H*-1,2,3-triazol-4-yl)methyl)-9*H*-purine (37b)**

Compound **37b** was prepared using the procedure mentioned above from compound **8** (50 mg, 0.26 mmol) and 2-azido-1-(4-fluorophenyl)ethanol **17b** (56 mg, 0.31 mmol). After purification via column chromatography (CH_2_Cl_2_/CH_3_OH = 30:1), compound **37b** was isolated as a white powder (28 mg, 29%, m.p. = 131–134 °C). ^1^H NMR (400 MHz, DMSO) *δ* 8.81 (s, 1H, H-2), 8.77 (s, 1H, H-8), 8.06 (s, 1H, H-triazole), 7.34 (d, *J* = 2.9 Hz, 2H, H-Ph), 7.09 (m, 2H, H-Ph), 5.82 (d, *J* = 4.7 Hz, 1H, OH), 5.61 (s, 2H, CH_2_), 4.98–4.94 (m, 1H, CHCH_2_), 4.46 (t, *J* = 6.6 Hz, 2H, CHCH_2_). ^13^C NMR (101 MHz, DMSO) *δ* 161.97 (d, *J_CF_* = 242.9 Hz), 152.17, 149.58, 147.85, 141.78, 138.54, 131.24, 128.48 (d, *J_CF_* = 8.1 Hz), 127.93, 124.96, 115.32 (d, *J_CF_* = 21.3 Hz), 71.05, 56.97, a signal assigned to the CH_2_ group is obscured by the DMSO signal. Anal. calcd. For C_16_H_13_FClN_7_O (Mr = 373.78) C, 51.41; H, 3.51; N, 26.23; found C, 51.49; H, 3.47; N, 26.19.

**6-Chloro-9-((1-(2-hydroxy-2-(4-chlorophenyl)ethyl)-1*H*-1,2,3-triazol-4-yl)methyl)-9*H*-purine (38b)**

Compound **38b** was prepared using the procedure mentioned above from compound **8** (50 mg, 0.26 mmol) and 2-azido-1-(4-chlorophenyl)ethanol **18b** (61 mg, 0.31 mmol). After purification via column chromatography (CH_2_Cl_2_/CH_3_OH = 30:1), compound **38b** was isolated as a white powder (81 mg, 80%, m.p. = 89–92 °C). ^1^H NMR (300 MHz, DMSO) *δ*/ppm: 8.81 (s, 1H, H-2), 8.77 (s, 1H, H-8), 8.07 (s, 1H, H-triazole), 7.32 (m, 4H, H-Ph), 5.87 (d, *J* = 4.7 Hz, 1H, OH), 5.61 (s, 2H, CH_2_), 4.96 (dt, *J* = 9.2, 4.6 Hz, 1H, CHCH_2_), 4.54–4.37 (m, 2H, CHCH_2_). ^13^C NMR (151 MHz, DMSO) *δ*/ppm: 151.55, 151.51, 148.96, 147.24, 141.18, 140.69, 131.86, 130.61, 127.92, 127.75, 124.36, 70.37, 56.19, a signal assigned to the CH_2_ group is obscured by the DMSO signal. Anal. calcd. For C_16_H_13_Cl_2_N_7_O (Mr = 390.23) C, 49.25; H, 3.36; N, 25.13; found C, 49.19; H, 3.41; N, 25.09.

**6-Chloro-9-((1-(2-hydroxy-2-(4-bromophenyl)ethyl)-1*H*-1,2,3-triazol-4-yl)methyl)-9*H*-purine (39b)**

Compound **39b** was prepared using the procedure mentioned above from compound **8** (50 mg, 0.26 mmol) and 2-azido-1-(4-bromophenyl)ethanol **19b** (75 mg, 0.31 mmol). After purification via column chromatography (CH_2_Cl_2_/CH_3_OH = 30:1), compound **39b** was isolated as a white powder (66 mg, 58%, m.p. = 86–89 °C). ^1^H NMR (600 MHz, DMSO) *δ*/ppm: 8.79 (s, 1H, H-2), 8.74 (s, 1H, H-8), 8.05 (s, 1H, H-triazole), 7.45–7.43 (m, 2H, H-Ph), 7.24 (d, *J* = 1.6 Hz, 2H, H-Ph), 5.82 (d, *J* = 4.8 Hz, 1H, OH), 5.60 (s, 2H, CH_2_), 4.96–4.93 (m, 1H, CHCH_2_), 4.46 (dd, *J* = 15.9, 6.0 Hz, 2H, CHCH_2_). ^13^C NMR (151 MHz, DMSO) *δ*/ppm: 151.69, 151.65, 149.09, 147.38, 141.32, 141.25, 130.97, 130.75, 128.25, 124.49, 120.55, 70.55, 56.27, a signal assigned to the CH_2_ group is obscured by the DMSO signal. Anal. calcd. For C_16_H_13_BrClN_7_O (Mr = 434.68) C, 44.21; H, 3.01 N, 22.56; found C, 44.27; H, 3.08; N, 22.50.

**6-Chloro-9-((1-(2-hydroxy-2-(4-(trifluoromethyl)phenyl)ethyl)-1*H*-1,2,3-triazol-4-yl)methyl)-9*H*-purine (40b)**

Compound **40b** was prepared using the procedure mentioned above from compound **8** (50 mg, 0.26 mmol) and 2-azido-1-(4-(trifluoromethyl)phenyl)ethanol **20b** (72 mg, 0.31 mmol). After purification via column chromatography (CH_2_Cl_2_/CH_3_OH = 30:1), compound **40b** was isolated as a white powder (68 mg, 60%, m.p. = 84–86 °C). ^1^H NMR (300 MHz, DMSO) *δ*/ppm: 8.81 (s, 1H, H-2), 8.76 (d, *J* = 6.0 Hz, 1H, H-8), 8.11 (s, 1H, H-triazole), 7.64 (d, *J* = 8.3 Hz, 2H, H-Ph), 7.53 (d, *J* = 8.1 Hz, 2H, H-Ph), 5.98 (d, *J* = 4.7 Hz, 1H, OH), 5.62 (s, 2H, CH_2_), 5.13–4.93 (m, 1H, CHCH_2_), 4.51 (qd, *J* = 13.8, 6.0 Hz, 2H, CHCH_2_). ^13^C NMR (151 MHz, DMSO) *δ*/ppm: 151.65, 151.63, 149.07, 147.35, 146.50, 141.34, 130.72, 128.06 (q, *J_CF3_* = 31.7 Hz), 126.89 (q, *J_CF3_* = 271.8 Hz), 124.93 (q, *J_CF3_* = 3.6 Hz), 124.50, 121.45, 70.59, 56.18, a signal assigned to the CH_2_ group is obscured by the DMSO signal. Anal. calcd. For C_17_H_13_ClF_3_N_7_O (Mr = 434.68) C, 48.18; H, 3.09; N, 23.14; found C, 48.23; H, 3.02; N, 23.11.

**4-Chloro-7-((1-(2-hydroxy-2-phenylethyl)-1*H*-1,2,3-triazol-4-yl)methyl)-7*H*-pyrrolo[2,3-*d*]pyrimidine (41b)**

Compound **41b** was prepared using the procedure mentioned above from compound **9** (50 mg, 0.26 mmol) and 2-azido-2-phenylethanol **16b** (51 mg, 0.31 mmol). After purification via column chromatography (CH_2_Cl_2_/CH_3_OH = 50:1), compound **41b** was isolated as a white powder (57 mg, 62%, m.p. = 114–117 °C). ^1^H NMR (400 MHz, DMSO) *δ*/ppm: 8.68 (s, 1H, H-2), 7.97 (s, 1H, H-triazole), 7.78 (d, *J* = 3.6 Hz, 1H, H-6), 7.29 (dd, *J* = 12.4, 4.5 Hz, 5H, H-Ph), 6.69 (d, *J* = 3.6 Hz, 1H, H-5), 5.76 (d, *J* = 4.7 Hz, 1H, OH), 5.57 (s, 2H, CH_2_), 4.94 (dt, *J* = 8.7, 4.6 Hz, 1H, CHCH_2_), 4.50–4.40 (m, 2H, CHCH_2_). ^13^C NMR (101 MHz, DMSO) *δ*/ppm: 151.13, 150.90, 150.82, 142.61, 142.39, 131.71, 128.58, 127.96, 126.46, 124.74, 117.25, 99.28, 71.64, 57.01, a signal assigned to the CH_2_ group is obscured by the DMSO signal. Anal. calcd. For C_17_H_15_ClN_6_O (Mr = 354.80) C, 57.55; H, 4.26; N, 23.69; found C, 57.49; H, 4.31; N, 23.73.

**4-Chloro-7-((1-(2-hydroxy-2-(4-fluorophenyl)ethyl)-1*H*-1,2,3-triazol-4-yl)methyl)-7*H*-pyrrolo[2,3-*d*]pyrimidine (42b)**

Compound **42b** was prepared using the procedure mentioned above from compound **9** (50 mg, 0.26 mmol) and 2-azido-1-(4-fluorophenyl)ethanol **17b** (56 mg, 0.31 mmol). After purification via column chromatography (CH_2_Cl_2_/CH_3_OH = 50:1), compound **42b** was isolated as a white powder (49 mg, 51%, m.p. = 154–156 °C). ^1^H NMR (400 MHz, DMSO) *δ*/ppm: 8.68 (s, 1H, H-2), 7.95 (s, 1H, H-triazole), 7.77 (d, *J* = 3.6 Hz, 1H, H-6), 7.33 (dd, *J* = 8.5, 5.7 Hz, 2H, H-Ph), 7.09 (t, *J* = 8.9 Hz, 2H, H-Ph), 6.69 (d, *J* = 3.6 Hz, 1H, H-5), 5.81 (d, *J* = 4.7 Hz, 1H, OH), 5.57 (s, 2H, CH_2_), 4.96 (dt, *J* = 9.4, 4.8 Hz, 1H, CHCH_2_), 4.44 (dd, *J* = 12.1, 4.9 Hz, 2H, CHCH_2_). ^13^C NMR (151 MHz, DMSO) *δ*/ppm: 161.45 (d, *J_CF_* = 243.0 Hz), 150.63, 150.38, 150.30, 142.13, 138.04 (d, *J* = 2.7 Hz), 131.18, 127.94 (d, *J_CF_* = 8.2 Hz), 124.21, 116.72, 114.79 (d, *J_CF_* = 21.2 Hz), 98.76, 70.54, 56.38, a signal assigned to the CH_2_ group is obscured by the DMSO signal. Anal. calcd. For C_17_H_14_ClFN_6_O (Mr = 372.79) C, 54.77; H, 3.79; N, 22.54; found C, 54.83; H, 3.74; N, 22.59.

**4-Chloro-7-((1-(2-hydroxy-2-(4-chlorophenyl)ethyl)-1*H*-1,2,3-triazol-4-yl)methyl)-7*H*-pyrrolo[2,3-*d*]pyrimidine (43b)**

Compound **43b** was prepared using the procedure mentioned above from compound **9** (50 mg, 0.26 mmol) and 2-azido-1-(4-chlorophenyl)ethanol **18b** (61 mg, 0.31 mmol). After purification via column chromatography (CH_2_Cl_2_/CH_3_OH = 50:1), compound **43b** was isolated as a white powder (52 mg, 51%, m.p. = 152–154 °C). ^1^H NMR (600 MHz, DMSO) *δ*/ppm: 8.68 (s, 1H, H-2), 7.96 (s, 1H, H-triazole), 7.77 (d, *J* = 3.6 Hz, 1H, H-6), 7.33–7.29 (m, 4H, H-Ph), 6.68 (d, *J* = 3.6 Hz, 1H, H-5), 5.86 (d, *J* = 4.7 Hz, 1H, OH), 5.56 (s, 2H, CH_2_), 4.98–4.94 (m, 1H, CHCH_2_), 4.49–4.41 (m, 2H, CHCH_2_). ^13^C NMR (151 MHz, DMSO) *δ*/ppm: 151.16, 150.91, 150.83, 142.69, 141.36, 132.48, 131.71, 128.47, 128.29, 124.75, 117.24, 99.29, 71.01, 56.75, a signal assigned to the CH_2_ group is obscured by the DMSO signal. Anal. calcd. For C_17_H_14_Cl_2_N_6_O (Mr = 389.24) C, 52.46; H, 3.63; N, 21.59; found C, 52.40; H, 3.71; N, 21.65.

**4-Chloro-7-((1-(2-hydroxy-2-(4-bromophenyl)ethyl)-1*H*-1,2,3-triazol-4-yl)methyl)-7*H*-pyrrolo[2,3-*d*]pyrimidine (44b)**

Compound **44b** was prepared using the procedure mentioned above from compound **9** (50 mg, 0.26 mmol) and 2-azido-1-(4-bromophenyl)ethanol **19b** (75 mg, 0.31 mmol). After purification via column chromatography (CH_2_Cl_2_/CH_3_OH = 50:1), compound **44b** was isolated as a white powder (50 mg, 44%, m.p. = 161–164 °C). ^1^H NMR (300 MHz, DMSO) *δ*/ppm: 8.68 (s, 1H, H-2), 7.97 (s, 1H, H-triazole), 7.77 (d, *J* = 3.6 Hz, 1H, H-6), 7.46 (d, *J* = 8.4 Hz, 2H, H-Ph), 7.25 (d, *J* = 8.4 Hz, 2H, H-Ph), 6.69 (d, *J* = 3.6 Hz, 1H, H-5), 5.86 (d, *J* = 4.7 Hz, 1H, OH), 5.57 (s, 2H, CH_2_), 4.94 (dd, *J* = 8.4, 3.7 Hz, 1H, CHCH_2_), 4.45 (dd, *J* = 5.9, 4.2 Hz, 2H, CHCH_2_). ^13^C NMR (151 MHz, DMSO) *δ*/ppm: 151.16, 150.90, 150.82, 142.67, 141.76, 131.69, 131.45, 128.72, 124.75, 121.02, 117.24, 99.28, 71.05, 56.69, a signal assigned to the CH_2_ group is obscured by the DMSO signal. Anal. calcd. For C_17_H_14_BrClN_6_O (Mr = 433.69) C, 47.08; H, 3.25; N, 19.38; found C, 47.15; H, 3.17; N, 19.43.

**4-Chloro-7-((1-(2-hydroxy-2-(4-(trifluoromethyl)phenyl)ethyl)-1*H*-1,2,3-triazol-4-yl)methyl)-7*H*-pyrrolo[2,3-*d*]pyrimidine (45b)**

Compound **45b** was prepared using the procedure mentioned above from compound **9** (50 mg, 0.26 mmol) and 2-azido-1-(4-(trifluoromethyl)phenyl)ethanol **20b** (72 mg, 0.31 mmol). After purification via column chromatography (CH_2_Cl_2_/CH_3_OH = 50:1), compound **45b** was isolated as a white powder (23 mg, 21%, m.p. = 74–75 °C). ^1^H NMR (600 MHz, DMSO) *δ*/ppm: 8.68 (s, 1H, H-2), 8.00 (s, 1H, H-triazole), 7.77 (d, *J* = 3.6 Hz, 1H, H-6), 7.63 (d, *J* = 8.2 Hz, 2H, H-Ph), 7.52 (d, *J* = 8.1 Hz, 2H, H-Ph), 6.67 (d, *J* = 3.6 Hz, 1H, H-5), 5.98 (d, *J* = 4.8 Hz, 1H, OH), 5.57 (s, 2H, CH_2_), 5.08–5.06 (m, 1H, CHCH_2_), 4.55–4.45 (m, 2H, CHCH_2_). ^13^C NMR (151 MHz, DMSO) *δ*/ppm: 151.14, 150.88, 150.81, 147.03, 142.72, 131.67, 128.55 (q, *J_CF3_* = 31.5 Hz), 125.50, 125.42 (q, *J_CF3_* = 3.5 Hz), 124.77, 124.69 (d, *J_CF3_* = 272.2 Hz), 117.22, 99.26, 71.10, 56.61, a signal assigned to the CH_2_ group is obscured by the DMSO signal. Anal. calcd. For C_18_H_14_F_3_N_6_O (Mr = 422.80) C, 51.14; H, 3.34; N, 19.88; found C, 51.09; H, 3.39; N, 19.81.

**4-Chloro-1-((1-(2-hydroxy-2-phenylethyl)-1*H*-1,2,3-triazol-4-yl)methyl)-1*H*-imidazo[4,5-*c*]pyridine (46b)**

Compound **46b** was prepared using the procedure mentioned above from compound **10** (50 mg, 0.26 mmol) and 2-azido-1-phenylethanol **16b** (51 mg, 0.31mmol). After purification via column chromatography (CH_2_Cl_2_/CH_3_OH = 50:1), compound **46b** was isolated as a white powder (71 mg, 77%, m.p. = 92–94 °C). ^1^H NMR (400 MHz, DMSO) *δ*/ppm: 8.57 (s, 1H, H-2), 8.17 (d, *J* = 5.6 Hz, 1H, H-6), 8.10 (s, 1H, H-triazole), 7.73 (d, *J* = 5.6 Hz, 1H, H-7), 7.28 (m, 5H, H-Ph), 5.81 (d, *J* = 4.6 Hz, 1H, OH), 5.65 (s, 2H, CH_2_), 4.99–4.93 (m, 1H, CHCH_2_), 4.54–4.42 (m, 2H, CHCH_2_,). ^13^C NMR (101 MHz, DMSO) *δ*/ppm: 146.64, 142.31, 141.82, 141.37, 141.30, 140.41, 137.59, 128.58, 128.00, 126.44, 125.06, 107.47, 71.73, 57.06, a signal assigned to the CH_2_ group is obscured by the DMSO signal. Anal. calcd. For C_17_H_15_ClN_6_O (Mr = 354.80) C, 57.55; H, 4.26; N, 23.69; found C, 57.41; H, 4.39; N, 23.73.

**4-Chloro-1-((1-(2-hydroxy-2-(4-fluorophenyl)ethyl)-1*H*-1,2,3-triazol-4-yl)methyl)-1*H*-imidazo[4,5-*c*]pyridine (47b)**

Compound **47b** was prepared using the procedure mentioned above from compound **10** (50 mg, 0.26 mmol) and 2-azido-1-(4-fluorophenyl)ethanol **17b** (56 mg, 0.31 mmol). After purification via column chromatography (CH_2_Cl_2_/CH_3_OH = 50:1), compound **47b** was isolated as a white powder (49 mg, 51%, m.p. = 98–100 °C). ^1^H NMR (300 MHz, DMSO) *δ*/ppm: 8.69 (s, 1H, H-2), 8.16 (d, *J* = 4.5 Hz, 1H, H-6), 7.98 (s, 1H, H-triazole), 7.74 (d, *J* = 4.5 Hz, 1H, H-7), 7.31 (dd, *J* = 8.6, 5.6 Hz, 2H, H-Ph), 7.06 (t, *J* = 8.9 Hz, 2H, H-Ph), 5.85–5.79 (m, 3H, OH, CH_2_), 4.96 (dd, *J* = 11.7, 4.8 Hz, 1H, CHCH_2_), 4.49–4.43 (m, 2H, CHCH_2_). ^13^C NMR (75 MHz, DMSO) *δ*/ppm: 160.33, 154.70 (d, *J_CF_* = 234.4 Hz), 152.26, 143.04, 141.16, 138.51 (d, *J_CF_* = 2.9 Hz), 136.68, 133.61, 128.43 (d, *J_CF_* = 8.2 Hz), 124.29, 115.27 (d, *J_CF_* = 21.3 Hz), 105.46, 71.02, 56.90, 41.86. Anal. calcd. For C_17_H_14_ClFN_6_O (Mr = 372.79) C, 54.77; H, 3.79; N, 22.54; found C, 54.81; H, 3.74; N, 22.48.

**4-Chloro-1-((1-(2-hydroxy-2-(4-chlorophenyl)ethyl)-1*H*-1,2,3-triazol-4-yl)methyl)-1*H*-imidazo[4,5-*c*]pyridine (48b)**

Compound **48b** was prepared using the procedure mentioned above from compound **10** (50 mg, 0.26 mmol) and 2-azido-1-(4-chlorophenyl)ethanol **18b** (61 mg, 0.31mmol). After purification via column chromatography (CH_2_Cl_2_/CH_3_OH = 50:1), compound **48b** was isolated as a white powder (39 mg, 39%, m.p. = 95–98 °C). ^1^H NMR (400 MHz, DMSO) *δ*/ppm: 8.57 (s, 1H, H-2), 8.17 (d, *J* = 5.6 Hz, 1H, H-6), 8.09 (s, 1H, H-triazole), 7.72 (d, *J* = 5.6 Hz, 1H, H-7), 7.30 (s, 4H, H-Ph), 5.89 (d, *J* = 4.6 Hz, 1H, OH), 5.64 (s, 2H, CH_2_), 4.97 (dt, *J* = 7.4, 4.6 Hz, 1H, CHCH_2_), 4.52–4.42 (m, 2H, CHCH_2_). ^13^C NMR (151 MHz, DMSO) *δ*/ppm: 146.66, 141.89, 141.42, 141.29, 140.40, 137.59, 132.48, 128.53, 128.35, 125.05, 107.46, 70.96, 56.79, a signal assigned to the CH_2_ group is obscured by the DMSO signal, and the signal of the one quaternary C atom was not observed. Anal. calcd. For C_17_H_14_Cl_2_N_6_O (Mr = 389.24) C, 52.46; H, 3.63; N, 21.59; found C, 52.51; H, 3.70; N, 21.51.

**4-Chloro-1-((1-(2-hydroxy-2-(4-bromophenyl)ethyl)-1*H*-1,2,3-triazol-4-yl)methyl)-1*H*-imidazo[4,5-*c*]pyridine (49b)**

Compound **49b** was prepared using the procedure mentioned above from compound **10** (50 mg, 0.26 mmol) and 2-azido-1-(4-bromophenyl)ethanol **19b** (75 mg, 0.31 mmol). After purification via column chromatography (CH_2_Cl_2_/CH_3_OH = 50:1), compound **49b** was isolated as a white powder (45 mg, 40%, m.p. = 107–109 °C). ^1^H NMR (300 MHz, DMSO) *δ*/ppm: 8.56 (s, 1H, H-2), 8.16 (d, *J* = 5.6 Hz, 1H, H-6), 8.09 (s, 1H, H-triazole), 7.71 (d, *J* = 5.6 Hz, 1H, H-7), 7.30 (s, 4H, H-Ph), 5.89 (d, *J* = 4.6 Hz, 1H, H-Ph), 5.63 (s, 2H, CH_2_), 4.96 (dt, *J* = 7.2, 4.7 Hz, 1H, CHCH_2_), 4.53–4.40 (m, 2H, CHCH_2_). ^13^C NMR (75 MHz, DMSO) *δ*/ppm: 146.66, 141.89, 141.42, 141.28, 140.40, 137.59, 132.48, 128.53, 128.35, 125.05, 107.46, 70.96, 56.79, a signal assigned to the CH_2_ group is obscured by the DMSO signal, and the signal of the one quaternary C atom was not observed. Anal. calcd. For C_17_H_14_BrClN_6_O (Mr = 433.69) C, 47.08; H, 3.25; N, 19.38; found C, 47.00; H, 3.16; N, 19.31.

#### 2.2.2. General Procedure for the Synthesis of Optically Enriched (*R*)- and (*S*)-*N*-Aryl-Substituted Derivatives of 6-Chloropurine and Pyrrolo[2,3-*d*]Pyrimidine with a Secondary Hydroxyl Group

The corresponding *N*-propargylated heterocyclic base **8**, **9** (1 eq.) was dissolved in methanol, and the corresponding optically enriched azido alcohols with a secondary hydroxyl group (*R*), (*S*)-**16b**-(*R*), (*S*)-**18b** and (*R*), (*S*)-**20b** (1.2 eq.), and Cu(OAc)_2_ (0.05 eq.) were added. The reaction mixture was irradiated under ultrasound for 2 h in a laboratory ultrasonic cleaning bath. The solvent was removed under reduced pressure, and the residue was purified via column chromatography.

**(*R*)-6-Chloro** **-9** **-((1-(2-hydroxy-2-phenylethyl)-1*H*-1,2,3-triazol-4-yl)methyl)-9*H*-purine ((*R*)-36b)**

Compound (*R*)-**36b** was prepared using the procedure mentioned above from compound **3** (45 mg, 0.23 mmol) and (*R*)-2-azido-1-phenylethanol (*R*)-**16b** (46 mg, 0.28 mmol, ee = 95%). After purification via column chromatography (CH_2_Cl_2_/CH_3_OH = 30:1) compound (*R*)-**36b** was isolated as a white powder (30 mg, 37%, m.p. = 129–131 °C, ee = 95%, [α]_D_^24^ − 30.0 (c = 0.0010)). ^1^H NMR (400 MHz, DMSO) *δ*/ppm: 8.81 (s, 1H, H-2), 8.77 (s, 1H, H-8), 8.07 (s, 1H, H-triazole), 7.33–7.23 (m, 5H, H-Ph), 5.77 (d, *J* = 4.7 Hz, 1H, OH), 5.62 (s, 2H, CH_2_), 4.95 (dt, *J* = 8.6, 4.5 Hz, 1H, CHCH_2_), 4.46 (qd, *J* = 13.7, 6.2 Hz, 2H, CHCH_2_). ^13^C NMR (101 MHz, DMSO) *δ*/ppm: 152.18, 152.13, 149.55, 147.83, 142.34, 141.74, 131.24, 128.57, 127.97, 126.45, 124.97, 71.75, 57.07, a signal assigned to the CH_2_ group is obscured by the DMSO signal. Anal. calcd. For C_16_H_14_ClN_7_O (Mr = 355.79) C, 54.01; H, 3.97; N, 27.56; found C, 54.11; H, 3.89; N, 27.64.

**(*S*)-6-Chloro** **-9** **-((1-(2-hydroxy-2-phenylethyl)-1*H*-1,2,3-triazol-4-yl)methyl)-9*H*-purine ((*S*)-36b)**

Compound (*S*)-**36b** was prepared using the procedure mentioned above from compound **3** (45 mg, 0.23 mmol) and (*S*)-2-azido-1-phenylethanol (*S*)-**16b** (45 mg, 0.28 mmol, ee = 99%). After purification via column chromatography (CH_2_Cl_2_/CH_3_OH = 30:1), compound (*S*)-**36b** was isolated as a white powder (48 mg, 59%, m.p. = 129–131 °C, ee = 99%, [α]_D_^24^ + 30.0 (c = 0.0010)). ^1^H NMR (400 MHz, DMSO) *δ*/ppm: 8.81 (s, 1H, H-2), 8.77 (s, 1H, H-8), 8.07 (s, 1H, H-triazole), 7.33–7.23 (m, 5H, H-Ph), 5.77 (d, *J* = 4.7 Hz, 1H, OH), 5.62 (s, 2H, CH_2_), 4.95 (dt, *J* = 8.6, 4.5 Hz, 1H, CHCH_2_), 4.46 (qd, *J* = 13.7, 6.2 Hz, 2H, CHCH_2_). ^13^C NMR (101 MHz, DMSO) *δ*/ppm: 152.18, 152.13, 149.55, 147.83, 142.34, 141.74, 131.24, 128.57, 127.97, 126.45, 124.97, 71.75, 57.07, a signal assigned to the CH_2_ group is obscured by the DMSO signal. Anal. calcd. For C_16_H_14_ClN_7_O (Mr = 355.79) C, 54.01; H, 3.97; N, 27.56; found C, 54.07; H, 4.03; N, 27.50.

**(*R*)-6-Chloro-9-((1-(2-hydroxy-2-(4-fluorophenyl)ethyl)-1*H*-1,2,3-triazol-4-yl)methyl)-9*H*-purine ((*R*)-37b)**

Compound (*R*)-**37b** was prepared using the procedure mentioned above from compound **3** (30 mg, 0.16 mmol) and 2-azido-1-(4-fluorophenyl)ethanol (*R*)-**17b** (34 mg, 0.19 mmol, ee = 99%). After purification via column chromatography (CH_2_Cl_2_/CH_3_OH = 30:1), compound (*R*)-**37b** was isolated as a white powder (26 mg, 27%, m.p. = 131–134 °C, ee = 99%, [α]_D_^24^ −20.0 (c = 0.0010)). ^1^H NMR (400 MHz, DMSO) *δ*/ppm: 8.81 (s, 1H, H-2), 8.77 (s, 1H, H-8), 8.06 (s, 1H, H-triazole), 7.34 (d, *J* = 2.9 Hz, 2H, H-Ph), 7.09 (m, 2H, H-Ph), 5.82 (d, *J* = 4.7 Hz, 1H, OH), 5.61 (s, 2H, CH_2_), 4.98–4.94 (m, 1H, CHCH_2_), 4.46 (t, *J* = 6.6 Hz, 2H, CHCH_2_). ^13^C NMR (101 MHz, DMSO) *δ*/ppm: 161.97 (d, *J_CF_* = 242.9 Hz), 152.17, 149.58, 147.85, 141.78, 138.54, 131.24, 128.48 (d, *J_CF_* = 8.1 Hz), 127.93, 124.96, 115.32 (d, *J_CF_* = 21.3 Hz), 71.05, 56.97, a signal assigned to the CH_2_ group is obscured by the DMSO signal. Anal. calcd. For C_16_H_13_FClN_7_O (Mr = 373.78) C, 51.41; H, 3.51; N, 26.23; found C, 51.46; H, 3.58; N, 26.19.

**(*S*)-6-Chloro-9-((1-(2-hydroxy-2-(4-fluorophenyl)ethyl)-1*H*-1,2,3-triazol-4-yl)methyl)-9*H*-purine ((*S*)-37b)**

Compound (*S*)-**37b** was prepared using the procedure mentioned above from compound **3** (30 mg, 0.16 mmol) and 2-azido-1-(4-fluorophenyl)ethanol (*S*)-**17b** (34 mg, 0.19 mmol, ee = 97%). After purification via column chromatography (CH_2_Cl_2_/CH_3_OH = 30:1), compound (*S*)-**37b** was isolated as a white powder (20 mg, 33%, m.p. = 131–134 °C, ee = 97%, [α]_D_^24^ + 18.2 (c = 0.0011)). ^1^H NMR (400 MHz, DMSO) *δ*/ppm: 8.81 (s, 1H, H-2), 8.77 (s, 1H, H-8), 8.06 (s, 1H, H-triazole), 7.34 (d, *J* = 2.9 Hz, 2H, H-Ph), 7.09 (m, 2H, H-Ph), 5.82 (d, *J* = 4.7 Hz, 1H, OH), 5.61 (s, 2H, CH_2_), 4.98–4.94 (m, 1H, CHCH_2_), 4.46 (t, *J* = 6.6 Hz, 2H, CHCH_2_). ^13^C NMR (101 MHz, DMSO) *δ*/ppm: 161.97 (d, *J_CF_* = 242.9 Hz), 152.17, 149.58, 147.85, 141.78, 138.54, 131.24, 128.48 (d, *J_CF_* = 8.1 Hz), 127.93, 124.96, 115.32 (d, *J_CF_* = 21.3 Hz), 71.05, 56.97, a signal assigned to the CH_2_ group is obscured by the DMSO signal. Anal. calcd. For C_16_H_13_FClN_7_O (Mr = 373.78) C, 51.41; H, 3.51; N, 26.23; found C, 51.38; H, 3.55; N, 26.18.

**(*R*)-6-Chloro-9-((1-(2-hydroxy-2-(4-chlorophenyl)ethyl)-1*H*-1,2,3-triazol-4-yl)methyl)-9*H*-purine) ((*R*)-38b)**

Compound (*R*)-**38b** was prepared using the procedure mentioned above from compound **5** (50 mg, 0.26 mmol) and 2-azido-1-(4-chlorophenyl)ethanol (*R*)-**18b** (61 mg, 0.31 mmol, ee *=* 89%). After purification via column chromatography (CH_2_Cl_2_/CH_3_OH = 50:1), compound (*R*)-**38b** was isolated as a white powder (43 mg, 44%, m.p. = 89–92 °C, ee *=* 89%, [α]_D_^24^ − 36.4 (c =0.0011)). ^1^H NMR (300 MHz, DMSO) *δ*/ppm: 8.81 (s, 1H, H-2), 8.77 (s, 1H, H-8), 8.07 (s, 1H, H-triazole), 7.32 (m, 4H, H-Ph), 5.87 (d, *J* = 4.7 Hz, 1H, OH), 5.61 (s, 2H, CH_2_), 4.96 (dt, *J* = 9.2, 4.6 Hz, 1H, CHCH_2_), 4.54–4.37 (m, 2H, CHCH_2_). ^13^C NMR (151 MHz, DMSO) *δ*/ppm: 151.55, 151.51, 148.96, 147.24, 141.18, 140.69, 131.86, 130.61, 127.92, 127.75, 124.36, 70.37, 56.19, a signal assigned to the CH_2_ group is obscured by the DMSO signal. Anal. calcd. For C_16_H_13_Cl_2_N_7_O (Mr = 390.23) C, 49.25; H, 3.36; N, 25.13; found C, 49.33; H, 3.31; N, 25.19.

**(*S*)-6-Chloro-9-((1-(2-hydroxy-2-(4-chlorophenyl)ethyl)-1*H*-1,2,3-triazol-4-yl)methyl)-9*H*-purine) ((*S*)-38b)**

Compound (*S*)-**38b** was prepared using the procedure mentioned above from compound **5** (58 mg, 0.30 mmol) and 2-azido-1-(4-chlorophenyl)ethanol (*S*)-**18b** (72 mg, 0.36 mmol, ee = 95%). After purification via column chromatography (CH_2_Cl_2_/CH_3_OH = 50:1), compound (*S*)-**38b** was isolated as a white powder (85 mg, 73%, m.p. = 89–92 °C, ee = 95%, [α]_D_^24^ + 33.3 (c = 0.0012)). ^1^H NMR (300 MHz, DMSO) *δ*/ppm: 8.81 (s, 1H, H-2), 8.77 (s, 1H, H-8), 8.07 (s, 1H, H-triazole), 7.32 (m, 4H, H-Ph), 5.87 (d, *J* = 4.7 Hz, 1H, OH), 5.61 (s, 2H, CH_2_), 4.96 (dt, *J* = 9.2, 4.6 Hz, 1H, CHCH_2_), 4.54–4.37 (m, 2H, CHCH_2_). ^13^C NMR (151 MHz, DMSO) δ 151.55, 151.51, 148.96, 147.24, 141.18, 140.69, 131.86, 130.61, 127.92, 127.75, 124.36, 70.37, 56.19, a signal assigned to the CH_2_ group is obscured by the DMSO signal. Anal. calcd. For C_16_H_13_C_l2_N_7_O (Mr = 390.23) C, 49.25; H, 3.36; N, 25.13; found C, 49.32; H, 3.289; N, 25.08.

**(*R*)-6-Chloro-9-((1-(2-hydroxy-2-(4-(trifluoromethyl)phenyl)ethyl)-1*H*-1,2,3-triazol-4-yl)methyl)-9*H*-purine ((*R*)-40b)**

Compound (*R*)-**40b** was prepared using the procedure mentioned above from compound **5** (50 mg, 0.26 mmol) and (*R*)-2-azido-1-(4-(trifluoromethyl)phenyl)ethanol (*R*)-**40b** (72 mg, 0.31 mmol, ee *=* 97%). After purification via column chromatography (CH_2_Cl_2_/CH_3_OH = 50:1), compound (*R*)-**40b** was isolated as a white powder (85 mg, 75%, m.p. = 84–86 °C, ee *=* 97%, [α]_D_^24^ − 45.5 (c = 0.0011). ^1^H NMR (300 MHz, DMSO) *δ*/ppm: 8.81 (s, 1H, H-2), 8.76 (d, *J* = 6.0 Hz, 1H, H-8), 8.11 (s, 1H, H-triazole), 7.64 (d, *J* = 8.3 Hz, 2H, H-Ph), 7.53 (d, *J* = 8.1 Hz, 2H, H-Ph), 5.98 (d, *J* = 4.7 Hz, 1H, OH), 5.62 (s, 2H, CH_2_), 5.13–4.93 (m, 1H, CHCH_2_), 4.51 (qd, *J* = 13.8, 6.0 Hz, 2H, CHCH_2_). ^13^C NMR (151 MHz, DMSO) *δ*/ppm: 151.65, 151.63, 149.07, 147.35, 146.50, 141.34, 130.72, 128.06 (q, *J_CF3_* = 31.7 Hz), 126.89 (q, *J_CF3_* = 271.8 Hz), 124.93 (q, *J_CF3_* = 3.6 Hz), 124.50, 121.45, 70.59, 56.18, a signal assigned to the CH_2_ group is obscured by the DMSO signal. Anal. calcd. For C_17_H_13_ClF_3_N_7_O (Mr = 434.68) C, 48.18; H, 3.09; N, 23.14; found C, 48.23; H, 3.14; N, 23.20.

**(*S*)-6-Chloro-9-((1-(2-hydroxy-2-(4-(trifluoromethyl)phenyl)ethyl)-1*H*-1,2,3-triazol-4-yl)methyl)-9*H*-purine ((*S*)-40b)**

Compound (*S*)-**40b** was prepared using the procedure mentioned above from compound **5** (50 mg, 0.26 mmol) and (*R*)-2-azido-1-(4-(trifluoromethyl)phenyl)ethanol (*S*)-**40b** (72 mg, 0.31 mmol, ee *=* 97%). After purification via column chromatography (CH_2_Cl_2_/CH_3_OH = 50:1), compound (*S*)-**40b** was isolated as a white powder (91 mg, 81%, m.p. = 84–86 °C, ee *=* 97%, [α]_D_^24^ + 50 (c =0.0010)). ^1^H NMR (300 MHz, DMSO) *δ*/ppm: 8.81 (s, 1H, H-2), 8.76 (d, *J* = 6.0 Hz, 1H, H-8), 8.11 (s, 1H, H-triazole), 7.64 (d, *J* = 8.3 Hz, 2H, H-Ph), 7.53 (d, *J* = 8.1 Hz, 2H, H-Ph), 5.98 (d, *J* = 4.7 Hz, 1H, OH), 5.62 (s, 2H, CH_2_), 5.13–4.93 (m, 1H, CHCH_2_), 4.51 (qd, *J* = 13.8, 6.0 Hz, 2H, CHCH_2_). ^13^C NMR (151 MHz, DMSO) *δ*/ppm: 151.65, 151.63, 149.07, 147.35, 146.50, 141.34, 130.72, 128.06 (q, *J_CF3_* = 31.7 Hz), 126.89 (q, *J_CF3_* = 271.8 Hz), 124.93 (q, *J_CF3_* = 3.6 Hz), 124.50, 121.45, 70.59, 56.18, a signal assigned to the CH_2_ group is obscured by the DMSO signal. Anal. calcd. For C_17_H_13_ClF_3_N_7_O (Mr = 434.68) C, 48.18; H, 3.09; N, 23.14; found C, 48.11; H, 3.13; N, 23.09.

**(*R*)-4-Chloro-7-((1-(2-hydroxy-2-phenylethyl)-1*H*-1,2,3-triazol-4-yl)methyl)-7*H*-pyrrolo[2,3-*d*]pyrimidine ((*R*)-41b)**

Compound (*R*)-**41b** was prepared using the procedure mentioned above from compound **9** (25 mg, 0.13 mmol) and 2-azido-2-phenylethanol (*R*)-**16b** (21 mg, 0.16 mmol, ee = 95%). After purification via column chromatography (CH_2_Cl_2_/CH_3_OH = 50:1), compound (*R*)-**41b** was isolated as a white powder (16 mg, 35%, m.p. = 114–117, ee = 95%, [α]_D_^24^ − 30.0 (c = 0.0010)). ^1^H NMR (400 MHz, DMSO) *δ*/ppm: 8.68 (s, 1H, H-2), 7.97 (s, 1H, H-triazole), 7.78 (d, *J* = 3.6 Hz, 1H, H-6), 7.29 (dd, *J* = 12.4, 4.5 Hz, 5H, H-Ph), 6.69 (d, *J* = 3.6 Hz, 1H, H-5), 5.76 (d, *J* = 4.7 Hz, 1H, OH), 5.57 (s, 2H, CH_2_), 4.94 (dt, *J* = 8.7, 4.6 Hz, 1H, CHCH_2_), 4.50–4.40 (m, 2H, CHCH_2_). ^13^C NMR (101 MHz, DMSO) *δ*/ppm: 151.13, 150.90, 150.82, 142.61, 142.39, 131.71, 128.58, 127.96, 126.46, 124.74, 117.25, 99.28, 71.64, 57.01. Anal. calcd. For C_17_H_15_ClN_6_O (Mr = 354.80) C, 57.55; H, 4.26; N, 23.69; found C, 57.61; H, 4.19; N, 23.61.

**(*S*)-4-Chloro-7-((1-(2-hydroxy-2-phenylethyl)-1*H*-1,2,3-triazol-4-yl)methyl)-7*H*-pyrrolo[2,3-*d*]pyrimidine ((*S*)-41b)**

Compound (*S*)-**41b** was prepared using the procedure mentioned above from compound **9** (54 mg, 0.28 mmol) and 2-azido-2-phenylethanol (*S*)-**16b** (55 mg, 0.34 mmol, ee = 99%). After purification via column chromatography (CH_2_Cl_2_/CH_3_OH = 50:1), compound (*S*)-**41b** was isolated as white powder (88 mg, 76%, m.p. = 114–117, ee = 99%, [α]_D_^24^ + 30.0 (c = 0.0010)). ^1^H NMR (400 MHz, DMSO) *δ*/ppm: 8.68 (s, 1H, H-2), 7.97 (s, 1H, H-triazole), 7.78 (d, *J* = 3.6 Hz, 1H, H-6), 7.29 (dd, *J* = 12.4, 4.5 Hz, 5H, H-Ph), 6.69 (d, *J* = 3.6 Hz, 1H, H-5), 5.76 (d, *J* = 4.7 Hz, 1H, OH), 5.57 (s, 2H, CH_2_), 4.94 (dt, *J* = 8.7, 4.6 Hz, 1H, CHCH_2_), 4.50–4.40 (m, 2H, CHCH_2_). ^13^C NMR (101 MHz, DMSO) *δ*/ppm: 151.13, 150.90, 150.82, 142.61, 142.39, 131.71, 128.58, 127.96, 126.46, 124.74, 117.25, 99.28, 71.64, 57.01. Anal. calcd. For C_17_H_15_ClN_6_O (Mr = 354.80) C, 57.55; H, 4.26; N, 23.69; found C, 57.48; H, 4.19; N, 23.62.

**(*R*)-4-Chloro-7-((1-(2-hydroxy-2-(4-fluorophenyl)ethyl)-1*H*-1,2,3-triazol-4-yl)methyl)-7*H*-pyrrolo[2,3-*d*]pyrimidine ((*R*)-42b)**

Compound (*R*)-**42b** was prepared using the procedure mentioned above from compound **9** (43 mg, 0.22 mmol) and 2-azido-1-(4-fluorophenyl)ethanol (*R*)-**17b** (50 mg, 0.27 mmol, ee = 99%). After purification via column chromatography (CH_2_Cl_2_/CH_3_OH = 50:1), compound (*R*)-**42b** was isolated as a white powder (44 mg, 54%, m.p. = 154–156 °C, ee = 99%, [α]_D_^24^ − 18.2 (c = 0.0011)). ^1^H NMR (400 MHz, DMSO) *δ*/ppm: 8.68 (s, 1H, H-2), 7.95 (s, 1H, H-triazole), 7.77 (d, *J* = 3.6 Hz, 1H, H-6), 7.33 (dd, *J* = 8.5, 5.7 Hz, 2H, H-Ph), 7.09 (t, *J* = 8.9 Hz, 2H, H-Ph), 6.69 (d, *J* = 3.6 Hz, 1H, H-5), 5.81 (d, *J* = 4.7 Hz, 1H, OH), 5.57 (s, 2H, CH_2_), 4.96 (dt, *J* = 9.4, 4.8 Hz, 1H, CHCH_2_), 4.44 (dd, *J* = 12.1, 4.9 Hz, 2H, CHCH_2_). ^13^C NMR (151 MHz, DMSO) *δ* 161.45 (d, *J_CF_* = 243.0 Hz), 150.63, 150.38, 150.30, 142.13, 138.04 (d, *J* = 2.7 Hz), 131.18, 127.94 (d, *J_CF_* = 8.2 Hz), 124.21, 116.72, 114.79 (d, *J_CF_* = 21.2 Hz), 98.76, 70.54, 56.38, a signal assigned to the CH_2_ group is obscured by the DMSO signal. Anal. calcd. For C_17_H_14_ClFN_6_O (Mr = 372.79) C, 54.77; H, 3.79; N, 22.54; found C, 54.71; H, 3.73; N, 22.49.

**(*S*)-4-Chloro-7-((1-(2-hydroxy-2-(4-fluorophenyl)ethyl)-1*H*-1,2,3-triazol-4-yl)methyl)-7*H*-pyrrolo[2,3-*d*]pyrimidine ((*S*)-42b)**

Compound (*S*)-**42b** was prepared using the procedure mentioned above from compound **9** (30 mg, 0.15 mmol) and 2-azido-1-(4-fluorophenyl)ethanol (*S*)-**17b** (36 mg, 0.19 mmol, ee = 97%). After purification via column chromatography (CH_2_Cl_2_/CH_3_OH = 50:1), compound (*S*)-**42b** was isolated as a white powder (30 mg, 54%, m.p. = 154–156 °C, ee = 97%, [α]_D_^24^ + 16.7 (c = 0.0012)). ^1^H NMR (400 MHz, DMSO) *δ*/ppm: 8.68 (s, 1H, H-2), 7.95 (s, 1H, H-triazole), 7.77 (d, *J* = 3.6 Hz, 1H, H-6), 7.33 (dd, *J* = 8.5, 5.7 Hz, 2H, H-Ph), 7.09 (t, *J* = 8.9 Hz, 2H, H-Ph), 6.69 (d, *J* = 3.6 Hz, 1H, H-5), 5.81 (d, *J* = 4.7 Hz, 1H, OH), 5.57 (s, 2H, CH_2_), 4.96 (dt, *J* = 9.4, 4.8 Hz, 1H, CHCH_2_), 4.44 (dd, *J* = 12.1, 4.9 Hz, 2H, CHCH_2_). ^13^C NMR (151 MHz, DMSO) *δ*/ppm: 161.45 (d, *J_CF_* = 243.0 Hz), 150.63, 150.38, 150.30, 142.13, 138.04 (d, *J* = 2.7 Hz), 131.18, 127.94 (d, *J_CF_* = 8.2 Hz), 124.21, 116.72, 114.79 (d, *J_CF_* = 21.2 Hz), 98.76, 70.54, 56.38, a signal assigned to the CH_2_ group is obscured by the DMSO signal. Anal. calcd. For C_17_H_14_ClFN_6_O (Mr = 372.79) C, 54.77; H, 3.79; N, 22.54; found C, 54.82; H, 3.83; N, 22.49.

**(*R*)-4-Chloro-7-((1-(2-hydroxy-2-(4-chlorophenyl)ethyl)-1*H*-1,2,3-triazol-4-yl)methyl)-7*H*-pyrrolo[2,3-*d*]pyrimidine ((*R*)-43b)**

Compound (*R*)-**43b** was prepared using the procedure mentioned above from compound **9** (52 mg, 0.27 mmol) and 2-azido-1-(4-chlorophenyl)ethanol (*R*)-**18b** (63 mg, 0.32 mmol, ee = 89%). After purification via column chromatography (CH_2_Cl_2_/CH_3_OH = 50:1), compound (*R*)-**43b** was isolated as a white powder (74 mg, 71%, m.p. = 152–154 °C, ee = 89%, [α]_D_^24^ − 40.0 (c = 0.0010)). ^1^H NMR (600 MHz, DMSO) *δ*/ppm: 8.68 (s, 1H, H-2), 7.96 (s, 1H, H-triazole), 7.77 (d, *J* = 3.6 Hz, 1H, H-6), 7.33–7.29 (m, 4H, H-Ph), 6.68 (d, *J* = 3.6 Hz, 1H, H-5), 5.86 (d, *J* = 4.7 Hz, 1H, OH), 5.56 (s, 2H, CH_2_), 4.98–4.94 (m, 1H, CHCH_2_), 4.49–4.41 (m, 2H, CHCH_2_). ^13^C NMR (151 MHz, DMSO) *δ*/ppm: 151.16, 150.91, 150.83, 142.69, 141.36, 132.48, 131.71, 128.47, 128.29, 124.75, 117.24, 99.29, 71.01, 56.75, a signal assigned to the CH_2_ group is obscured by the DMSO signal. Anal. calcd. For C_17_H_14_Cl_2_N_6_O (Mr = 389.24) C, 52.46; H, 3.63; N, 21.59; found C, 52.40; H, 3.69; N, 21.63.

**(*S*)-4-Chloro-7-((1-(2-hydroxy-2-(4-chlorophenyl)ethyl)-1*H*-1,2,3-triazol-4-yl)methyl)-7*H*-pyrrolo[2,3-*d*]pyrimidine ((*S*)-43b)**

Compound (*S*)-**43b** was prepared using the procedure mentioned above from compound **9** (52 mg, 0.27 mmol) and 2-azido-1-(4-chlorophenyl)ethanol (*S*)-**18b** (63 mg, 0.32 mmol, ee = 95%). After purification via column chromatography (CH_2_Cl_2_/CH_3_OH = 50:1), compound (*S*)-**43b** was isolated as a white powder (94 mg, 89%, m.p. = 152–154 °C, ee = 95%, [α]_D_^24^ + 40.0 (c = 0.0010)). ^1^H NMR (600 MHz, DMSO) *δ*/ppm: 8.68 (s, 1H, H-2), 7.96 (s, 1H, H-triazole), 7.77 (d, *J* = 3.6 Hz, 1H, H-6), 7.33–7.29 (m, 4H, H-Ph), 6.68 (d, *J* = 3.6 Hz, 1H, H-5), 5.86 (d, *J* = 4.7 Hz, 1H, OH), 5.56 (s, 2H, CH_2_), 4.98–4.94 (m, 1H, CHCH_2_), 4.49–4.41 (m, 2H, CHCH_2_). ^13^C NMR (151 MHz, DMSO) *δ*/ppm: 151.16, 150.91, 150.83, 142.69, 141.36, 132.48, 131.71, 128.47, 128.29, 124.75, 117.24, 99.29, 71.01, 56.75, a signal assigned to the CH_2_ group is obscured by the DMSO signal. Anal. calcd. For C_17_H_14_Cl_2_N_6_O (Mr = 389.24) C, 52.46; H, 3.63; N, 21.59; found C, 52.51; H, 3.58; N, 21.51.

**(*R*)-4-Chloro-7-((1-(2-hydroxy-2-(4-(trifluoromethyl)phenyl)ethyl)-1*H*-1,2,3-triazol-4-yl)methyl)-7*H*-pyrrolo[2,3-*d*]pyrimidin ((*R*)-45b)**

Compound (*R*)-**45b** was prepared using the procedure mentioned above from compound **9** (76 mg, 0.40 mmol) and (*R*)-2-azido-2-(4-(trifluoromethyl)phenyl)ethanol (*R*)-**20a** (110 mg, 0.47 mmol, ee = 97%). After purification via column chromatography (CH_2_Cl_2_/CH_3_OH = 50:1), compound (*R*)-**45b** was isolated as a white powder (101 mg, 60%, m.p. = 74–75 °C, ee = 97%, [α]_D_^24^ − 44.4 (c = 0.0009)). ^1^H NMR (600 MHz, DMSO) *δ*/ppm: 8.68 (s, 1H, H-2), 8.00 (s, 1H, H-triazole), 7.77 (d, *J* = 3.6 Hz, 1H, H-6), 7.63 (d, *J* = 8.2 Hz, 2H, H-Ph), 7.52 (d, *J* = 8.1 Hz, 2H, H-Ph), 6.67 (d, *J* = 3.6 Hz, 1H, H-5), 5.98 (d, *J* = 4.8 Hz, 1H, OH), 5.57 (s, 2H, CH_2_), 5.08–5.06 (m, 1H, CHCH_2_), 4.55–4.45 (m, 2H, CHCH_2_). ^13^C NMR (151 MHz, DMSO) *δ*/ppm: 151.14, 150.88, 150.81, 147.03, 142.72, 131.67, 128.55 (q, *J_CF3_* = 31.5 Hz), 125.50, 125.42 (q, *J_CF3_* = 3.5 Hz), 124.77, 124.69 (d, *J_CF3_* = 272.2 Hz), 117.22, 99.26, 71.10, 56.61, a signal assigned to the CH_2_ group is obscured by the DMSO signal. Anal. calcd. For C_18_H_14_F_3_N_6_O (Mr = 422.80) C, 51.14; H, 3.34; N, 19.88; found C, 51.21; H, 3.28; N, 19.95.

**(*S*)-4-Chloro-7-((1-(2-hydroxy-2-(4-(trifluoromethyl)phenyl)ethyl)-1*H*-1,2,3-triazol-4-yl)methyl)-7*H*-pyrrolo[2,3-*d*]pyrimidin ((*S*)-45b)**

Compound (*S*)-**45b** was prepared using the procedure mentioned above from compound **9** (76 mg, 0.40 mmol) and (*S*)-2-azido-2-(4-(trifluoromethyl)phenyl)ethanol (*S*)-**20a** (110 mg, 0.47 mmol, ee = 97%). After purification via column chromatography (CH_2_Cl_2_/CH_3_OH = 50:1), compound (*S*)-**45b** was isolated as a white powder (112 mg, 66%, m.p. = 74–75 °C, ee = 97%, [α]_D_^24^ + 50.0 (c = 0.0010)). ^1^H NMR (600 MHz, DMSO) *δ*/ppm: 8.68 (s, 1H, H-2), 8.00 (s, 1H, H-triazole), 7.77 (d, *J* = 3.6 Hz, 1H, H-6), 7.63 (d, *J* = 8.2 Hz, 2H, H-Ph), 7.52 (d, *J* = 8.1 Hz, 2H, H-Ph), 6.67 (d, *J* = 3.6 Hz, 1H, H-5), 5.98 (d, *J* = 4.8 Hz, 1H, OH), 5.57 (s, 2H, CH_2_), 5.08–5.06 (m, 1H, CHCH_2_), 4.55–4.45 (m, 2H, CHCH_2_). ^13^C NMR (151 MHz, DMSO) *δ*/ppm: 151.14, 150.88, 150.81, 147.03, 142.72, 131.67, 128.55 (q, *J_CF3_* = 31.5 Hz), 125.50, 125.42 (q, *J_CF3_* = 3.5 Hz), 124.77, 124.69 (d, *J_CF3_* = 272.2 Hz), 117.22, 99.26, 71.10, 56.61, a signal assigned to the CH_2_ group is obscured by the DMSO signal. Anal. calcd. For C_18_H_14_F_3_N_6_O (Mr = 422.80) C, 51.14; H, 3.34; N, 19.88; found C, 51.21; H, 3.29; N, 19.82.

### 2.3. Examination of Antiproliferative Activity In Vitro

#### Cell Culturing and Proliferation Assays

The human ATCC tumor cell lines breast adenocarcinoma (MCF7), colorectal carcinoma (HT-29), pancreatic ductal adenocarcinoma (CFPAC-1), hepatocellular carcinoma (HepG2), and normal skin fibroblasts (HFF) derived from ATCC (American Type Culture Collection) were cultured in Dulbecco’s Modified Eagle Medium (DMEM) or Minimum Essential Medium (MEM) supplemented with 10% fetal bovine serum (FBS), 2 mM L-glutamine, 100 U/mL penicillin, and 100 µg/mL streptomycin (Lonza, Basel, Switzerland) in a humidified atmosphere with 5% CO_2_ at 37 °C. For the experiment, carcinoma cell lines and normal human cell lines were seeded in 96-well microtiter plates at a density of 3000 cells per well and 5000 cells per well, respectively, depending on the doubling time of the specific cell line. The test substances were then added at fivefold, tenfold dilutions (0.01 to 100 µM), freshly prepared in the growth medium on the day of testing, and incubated for a further 72 h. After 72 h of incubation, the growth rate of the cells was determined using the MTT assay according to the manufacturer’s guidelines. The percentage of growth was calculated by transforming the experimentally determined absorbance values using the formulas suggested by the National Institutes of Health (NIH). The IC_50_ values were calculated from the dose–response curves using linear regression analysis. The experiment was performed in tetraplicates in two individual experiments.

### 2.4. In Vitro ADME Profiling

#### 2.4.1. MDCKII-MDR1 Permeability Assay

MDCKII-hMDR1 cells were obtained from Solvo Biotechnology, Hungary. DMEM, fetal bovine serum, Glutamax-100, Antibiotic/Antimycotic, DMSO, Dulbecco’s phosphate-buffered saline, and MEM Non-essential amino acids were purchased from Sigma (St. Louis, MO, USA). Bi-directional permeability and P-glycoprotein substrate assessments were carried out in Madin-Darby canine epithelial cells with the over-expressed human MDR1 gene (MDCKII-MDR1), coding for P-glycoprotein. Experimental procedures, as well as cell culture conditions, were the same as previously described [53]. Briefly, compounds (10 µM, 1% DMSO *v*/*v*) in duplicate were incubated at 37 °C for 60 min with cell monolayer on 24-well Millicell inserts (Millipore, Burlington, MA, USA) with and without the P-glycoprotein inhibitor Elacridar (2 µM, International Laboratory, San Francisco, CA, USA). The inhibition of P-glycoprotein was verified by amprenavir (Moravek Biochemicals Inc, Brea, CA, USA) and monolayer integrity using Lucifer yellow (Sigma, St. Louis, MO, USA). LC-MS/MS measured compound concentrations and Lucifer yellow was measured on an Infinite F500 (Tecan, Männedorf, CH, Switzerland) using excitation of 485 nm and emission of 530 nm.

#### 2.4.2. Metabolic Stability

Mouse liver microsomes were obtained from Corning Life Sciences (Corning, NY, USA). DMSO, nicotinamide adenine dinucleotide phosphate (NADP), glucose-6-phosphate, glucose-6-phosphate dehydrogenase, magnesium chloride, propranolol, caffeine, diclofenac, and phosphate-buffered saline (PBS) were purchased from Sigma (St. Louis, MO, USA). Acetonitrile (ACN) and methanol (MeOH) were obtained from Merck (Darmstadt, Germany). Testosterone was purchased from Steraloids (Newport, RI, USA). Metabolic stability was assessed in mouse liver microsomes. Compounds (final concentration of 1 µM, 0.03% DMSO *v*/*v*) were incubated in duplicate in phosphate buffer (50 mM, pH 7.4) at 37 °C together with mouse liver microsomes in the absence and presence of the NADPH cofactor (0.5 mM nicotinamide adenine dinucleotide phosphate, 5 mM glucose-6-phosphate, 1.5 U/mL glucose-6-phosphate dehydrogenase, and 0.5 mM magnesium chloride). Incubation and sampling were performed on a Freedom EVO 200 (Tecan, Männedorf, CH, Switzerland) at 0.3, 10, 20, 30, 45, and 60 min. The reaction was quenched using 3 volumes of a mixture of ACN/MeOH (2:1) containing internal standard (diclofenac), centrifuged, and the supernatants were analyzed using LC-MS/MS. The metabolic activity of the microsomes was verified via simultaneous analysis of several controls including testosterone, propranolol, and caffeine. The in vitro half-life (t_1/2_) was calculated using GraphPad Prism (v9.0 software) non-linear regression of % of parent compound remaining versus time. In vitro clearance, expressed as µL/min/mg, was estimated from the in vitro half-life (t_1/2_) and normalized for the protein amount in the incubation mixture, assuming 52.5 mg of protein per gram of liver and using constant values for mouse liver weight/body weight [87.5 g/kg] and mouse liver blood flow (LBF) [131 mL/min/kg].

#### 2.4.3. LC-MS/MS Analysis

All samples were quantified using tandem mass spectrometry coupled to liquid chromatography. Samples were analyzed on a Sciex API4000 Triple Quadrupole Mass Spectrometer (Sciex, Division of MDS Inc., Toronto, Canada) coupled to a Shimadzu Nexera X2 UHPLC frontend (Kyoto, Japan). Samples were injected onto a UHPLC column (HALO2 C18, 2.1 × 20 mm, 2 µm or Luna Omega 1.6 µm Polar C18 100A, 30 × 2.1 mm) and eluted with a gradient at 50 °C. The mobile phase was composed of acetonitrile/water mixture (9/1, with 0.1% formic acid) and 0.1% formic acid in deionized water. The flow rate was 0.7 mL/min under gradient conditions, leading to a total run time of 1.5–2 min. A positive ion mode with turbo spray, an ion source temperature of 550 °C, and a dwell time of 150 ms were utilized for mass spectrometric detection. Quantitation was performed using multiple reaction monitoring (MRM) at the specific transitions for each compound.

#### 2.4.4. In Silico Profiling

The in silico ADME properties as well as the structural parameters were calculated using ACD Percepta software (ACD/Percepta, v2021.1.3) [54]. Data analysis and visualization were performed using DataWarrior (DataWarrior v06.01.01, 2024). Cresset suite software (Flare™, v7.0) was used to perform field based alignment and the conformational search [55].

## 3. Results

### 3.1. Chemistry

**Scheme 1:**
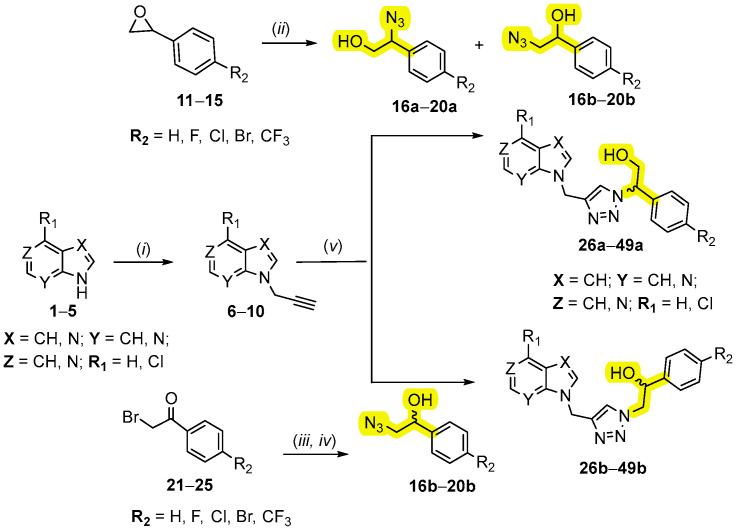
Cu(I)-catalyzed synthesis of novel racemic aryl-substituted derivatives of purines and purine isosteres. Reagents and conditions: (i) propargyl bromide, NaH, DMF, r.t., overnight; (ii) NaN_3_, NH_4_Cl, MeOH/H_2_O (8:1), 75 °C, overnight; (iii) NaN_3_, DMF, r.t, 0.5 h; (iv) NaBH_4,_ MeOH, r.t., 1 h; and (v) Cu(OAc)_2_, MeOH, 2 h, under ultrasound irradiation.

The targeted racemic **26a**–**49a**, **26b**–**49b**, and enantiomeric-enriched *N*-heterocycles (*R*)-**36b**-(*R*)-**38b**, (*R*)-**40b**, (*R*)-**41b**-(*R*)-**43b**, (*R*)-**45b**, and (*S*)-**36b**-(*S*)-**38b**, (*S*)-**40b**, (*S*)-**41b**-(*S*)-**43b**, and (*S*)-**45b** were synthesized via the copper(I)-catalyzed Huisgen 1,3-dipolar cycloaddition of terminal alkynyl derivatives of purine and purine isosteres **6**–**10** and 1,2-azido alcohols **16a**–**20a** and **16b**–**20b** using an environmentally benign synthetic protocol [56] with ultrasonic irradiation (Scheme 1).

The key intermediates *N*-propargylated indole (**6**) [45], benzimidazole (**7**) [43], 6-chloro-9*H*-purine (**8**) [43], 4-chloro-7*H*-pyrrolo[2,3-*d*]pyrimidine (**9**) [47], and 4-chloro-1*H*-imidazo[4,5-*c*]pyridine (**10**) [43] were obtained via *N*-alkylation of the corresponding heterocyclic base **1–5**. *N*-Alkylation of 6-chloropurine (**3**) and 4-chloro-1*H*-imidazo[4,5-*c*]pyridine (**5**) afforded *N*-9 regioisomers **8** and **10**, which was confirmed by 1D and 2D NMR techniques [43]. Racemic 1,2-azido alcohols with primary hydroxyl groups **16a–20a** were synthesized via a regioselective ring*-*opening reaction of various *para*-substituted *2-*phenyloxiranes 11**–**15 with sodium azide under basic conditions. The regioselectivity of the 2-phenyloxirane derivatives can be explained by the electronic effects of the aromatic substituent that direct the azide nucleophile towards the benzylic α-position due to the stabilization of the positive charge via resonance with the aromatic moiety [57]. However, the strong electron-withdrawing trifluoromethyl substituent at the phenyl ring in **15** significantly decreases the regioselectivity of **20a**/**20b** from 15.6 (α/*β*) to 1.5 (α/*β*) (Table 1). Consequently, *α*-azido alcohols **16a–20a** with the primary hydroxyl group were isolated as major products in very good yields (51**–**88%) [48,49,50], while *β*-azido alcohols **16b–20b** with the secondary hydroxyl group were obtained in yields of 6**–**34% (Table 1).

**Scheme 2:**
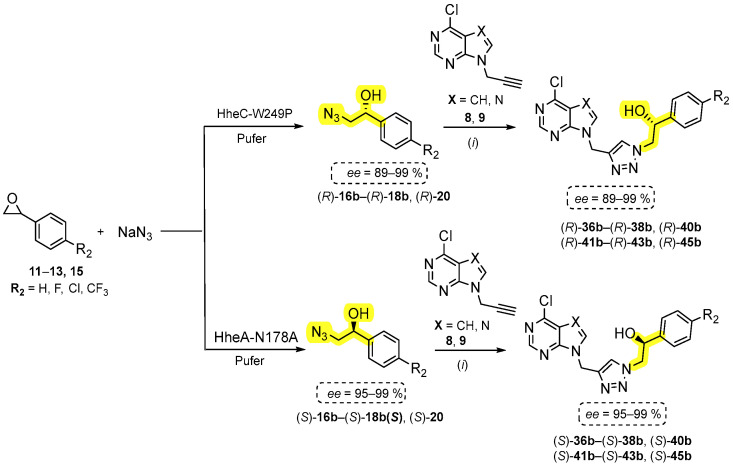
Cu(I)-catalyzed synthesis of novel optically enriched (*R*)- and (*S*)-aryl-substituted derivatives of 6-chloropurine and 4-chloropyrrolo[2,3-*d*]pyrimidine. Reagents and conditions: (i) MeOH, Cu(OAc)_2_, 2 h, under ultrasound irradiation.

Therefore, a new synthetic pathway for *β*-azido alcohols was used in which *p-*substituted phenacyl bromides were treated with sodium azide, followed by fast reduction to afford corresponding *β*-azido alcohols **16b**–**20b [49,51,52]**. Enantioenriched (ee = 89**–**99%) *β*-azido alcohols **16b–18b** and **20b**, as *R*- and *S*-enantiomers, were obtained via biocatalytic azidolysis of various *para*-substituted 2-phenyloxiranes with sodium azide as a nucleophile and halohydrin dehalogenase, which were found to be enantioselective (Scheme 2) [58].

The application of halohydrin dehalogenases and biocatalysis in general, particularly in the synthesis of active pharmaceutical ingredients, has evolved into an environmentally friendly method [59]. For the synthesis of enantiopure (*R*)-1,2-azido alcohols, the HheC-W249P variant was used, while for (*S*)-1,2-azido alcohols, HheA-N178A was applied [60]. Absolute configurations of 1,2-azido alcohols were assigned based on the previously reported data [60,61]. Novel enantioenriched (*R*)- and (*S*)-*N*-aryl-substituted derivatives of 6-chloropurine ((*R*)-**36b**-(*R*)-**38b**, (*R*)-**40b**, (*S*)-**36b**-(*S*)-**38b,** and (*S*)-**40b**) and pyrrolo[2,3-*d*]pyrimidine ((*R*)-**41b**-(*R*)-**43b**, (*R*)-**45b**, (*S*)-**41b**-(*S*)-**43b,** and (*S*)-**45b**) were subsequently synthesized using the copper(I)-catalyzed 1,3-dipolar cycloaddition of propargylated *N*-heterocycles **8** or **9** and optically pure *ß*-substituted azido alcohols **16b–18b** and **20b** under ultrasound irradiation (Scheme 2).

### 3.2. Biological Profiling

#### 3.2.1. Evaluation of Antiproliferative Activity

Newly synthesized compounds were evaluated for their cytotoxic activity in vitro against four human cancer cell lines, including human breast adenocarcinoma (MCF-7), ductal pancreatic adenocarcinoma (CFPAC-1), colorectal carcinoma (HT-29), and hepatocellular carcinoma (HepG2), as well as on non-cancerous skin fibroblasts (HFF-1), to check for non-specific toxicity. Furthermore, 5-Fluorouracil (5-FU) was used as a reference drug. The results are expressed as 50% inhibitory concentration (IC_50_) values and are listed in Table 2, Table 3, Table 4 and Table 5. It can be observed that purine (**36a**–**40a** and **36b**–**40b**) (Table 3) and pyrrolo[2,3-*d]*pyrimidine (**41a**–**45a** and **41b**–**45b**) (Table 4) derivatives exhibited better antiproliferative activity than indole (**26a**–**30a** and **26b**–**30b**) (Table 2), benzimidazole (**31a**–**35a** and **31b**–**35b**) (Table 2), and imidazo[4,5-*c*]pyridine (**46a**–**49a** and **46b**–**49b**) (Table 5) derivatives.

From indoles and benzimidazoles, the best growth inhibition was observed in CFPAC-1 cells, while from 6-chloropurines and 4-chloro-1*H*-imidazo[4,5-*c*]pyridines, in HepG2 cells. Among 4-chloropyrrolo[2,3-*d*]pyrimidines, the most pronounced inhibition was found in MCF-7 and CFPAC-1 cells.

In addition, *N*-heterocyclic derivatives with the halogen-substituted aromatic unit had better inhibitory activity than their unsubstituted analogs.

From indoles, purines, and pyrrolo[2,3-*d*]pyrimidines, activities decreased in the following order: CF_3_ > Br > Cl > F. For instance, indole **30a**, purine **40a**, and pyrrolo[2,3-*d*]pyrimidine **45a** with 4-(trifluoromethyl)phenyl moiety showed the highest inhibitory effect (**30a**: IC_50_ = 9 µM on CFPAC-1; **40a**: IC_50_ = 0.69 µM on HT-29, IC_50_ = 0.64 µM on HepG2; and **45a**: IC_50_ = 0.5 µM on MCF-7 cell line). These compounds also contain the primary hydroxyl group in a 2-hydroxyeth-1-yl spacer.

They exhibited toxicity to normal skin fibroblasts (HFF-1) with selectivity indexes (SI) ranging from 4 to 40. The best selectivity compared to a non-tumor cell line was observed for pyrrolo[2,3-*d*]pyrimidine **45a**, with a SI of 40. Pyrrolo[2,3-*d*]pyrimidine derivatives (**43a** and **45a**) showed the best inhibitory activity on the CFPAC-1 cell line, with IC_50_ values ranging from 1 to 8 µM. Non-substituted aryl indoles (**26a** and **26b**), benzimidazoles (**31a** and **31b**), and imidazo[4,5-*c*]pyridines (**46a** and **46b**) were deprived of any activity.

Comparing the influence of the hydroxyethyl linker and enantiomers of 6-chloropurines and 4-chloropyrrolo[2,3-*d*]pyrimidines on activity, we may conclude that the linker with the primary hydroxyl group in regioisomers of the **26a**–**45a** series had a generally higher growth-inhibiting effect on the tested cell lines than the linker with the secondary hydroxyl group in the **26b**–**45b** series, and that the *S*-enantiomers were more active than the corresponding *R*-enantiomers. Although the difference in antitumor activity was not pronounced, the *S*-enantiomers were twofold less cytotoxic to normal fibroblasts (HFF-1) than the corresponding *R*-enantiomers.

**Figure 3:**
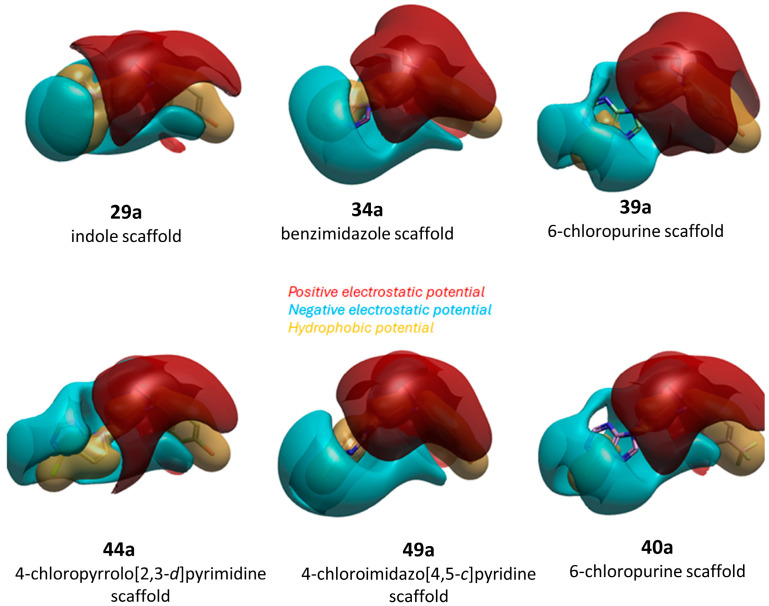
Electrostatic potential calculated from molecular fields for *p*-bromophenyl-substituted derivatives from explored subseries aligned on the minimum energy conformation of the *p*-(trifluoromethyl)phenyl-substituted 6-chloropurine **40a**.

To better understand the observed SAR, the *p*-bromophenyl-substituted derivatives with the primary hydroxyl group—**29a**, **34a**, **39a**, **44a,** and **49a**—were aligned on the minimum energy conformation of compound **40a**, and the molecular fields calculated by the Cresset software [55] were compared (Figure 3). All compounds were well aligned on the minimum energy conformation of compound **40a**, indicating that the observed potency differences came from differences in the distribution of the electrostatic potential. The combination of the lower lipophilicity and larger negative electrostatic potential of the 6-chloropurine scaffold compared to the other heterocycles resulted in its higher potency. In addition, the increased lipophilic potential of the trifluoromethyl-phenyl substitution also contributed to the better potency profile when compared to the bromo analogs, and resulted in the most active compound **40a**.

**Figure 4:**
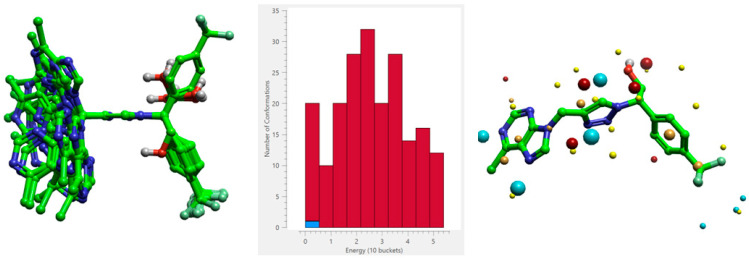
Conformational analysis of compound **40a**: left—conformational distribution, middle—energy histogram of generated conformations, and right—minimum energy conformation.

Due to a larger number of rotational bonds, conformational space is large, with over 100 conformations within 3 kcal/mol, as demonstrated in Figure 4 for the most active compound **40a** on HT-29 and HepG2 cells. The most stable is trans-conformation, while the least stable conformations adopt a cis-geometry of left- and right-hand side aromatic moieties with respect to the central triazole.

#### 3.2.2. Physico-Chemical and ADME Properties

The calculated structural (logP, TPSA, HBD, and HBA) and PhysChem parameters (solubility, %PPB, and probability of CYP3A4 inhibition), as well as the measured permeability and metabolic stability (clearance values, CL) are shown in Table 6. All compounds are in the good lipophilicity range and fit within the Lipinski rule of 5. The predicted solubility varies from very soluble to insoluble, as a balance between lipophilicity and polarity, additionally influenced by the number of heteroatoms in the bicyclic moiety, the primary or secondary alcohol, and the substituent on the right-hand side phenyl. The predicted plasma protein binding is in the range of 91.5–99.5, and although not necessarily very accurate in absolute value predictions, gives quite a good understanding of the relative free fraction within the studied series. Most significant cytochrome P450 (CYP3A4) inhibition is flagged for pyrrolo[2,3-*d*]pyrimidine derivatives **44a,b** and **45a,b**, while compounds **39a,b**, **40a,b,c**, and **49a,b** are predicted as possible CYP3A4 inhibitors (Table 6). This should be further de-risked for future, more advanced compounds.

Purine and imidazo[4,5-*c*]pyridine analogs showed high metabolic stability due to decreased potential for aromatic hydroxylation, while indole and pyrrolo[2,3-*d*]pyrimidine derivatives exhibited low metabolic stability. Compounds with secondary alcohols were metabolically less stable than compounds with primary alcohols, indicating a higher contribution of *N*-dealkylation in comparison with *O*-dealkylation. Indole and pyrrolo[2,3-*d*]pyrimidine derivatives had good permeability, while benzimidazole, 6-chloropurine, and imidazo[4,5-*c*]pyridine analogs had low permeability, due to decreased lipophilicity.

Selected 6-chloropurine (**40a**), 4-chloropyrrolo[2,3-*d*]pyrimidine (**45a**), and 4-chloroimidazo[4,5-*c*]pyridine (**49b**) with significant antiproliferative activity were examined for hydrolytic stability in an aqueous buffer solution. No significant changes were observed in their UV/Vis spectra over 24 h, indicating that these compounds remain hydrolytically stable under the tested conditions (Supporting Information S49).

**Figure 5:**
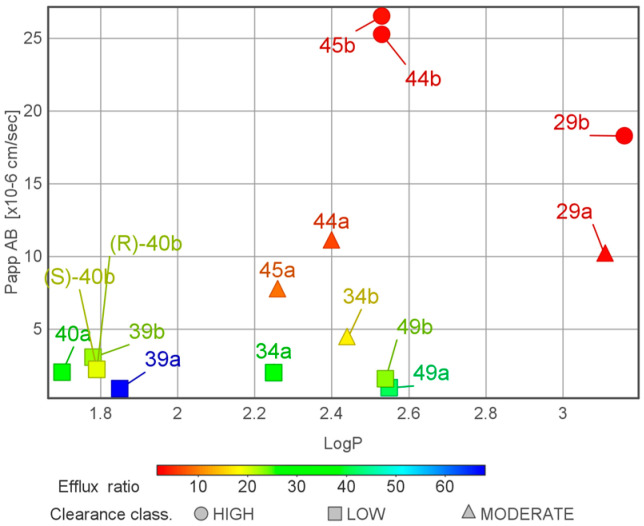
Graphical presentation of measured ADME parameters.

Permeability is also influenced by a high efflux ratio for the majority of compounds. Compounds **29b**, **44b**, and **45b** had the highest permeability and low efflux, but also had high metabolic clearance, while compounds **39a**, **39b**, **40a**, and **40b** with low clearance demonstrated low permeability and higher efflux values, as shown in Figure 5.

## 4. Discussion

Enantiomers of 6-chloropurine and 4-chloropyrrolo[2,3-*d*]pyrimidine with hydroxyethyl linker were obtained via the biocatalytic ring-opening of epoxides with halohydrin dehalogenase to afford optically pure *ß*-substituted azido alcohols that subsequently, with *N*-propargylated 6-chloropurine and 4-chloropyrrolo[2,3-*d*]pyrimidine, gave the corresponding (*R*)- and (*S*)-*N*-aryl-substituted derivatives (*R*)-**36b**–(*R*)-**38b**, (*R*)-**40b** (*R*)-**41b**–(*R*)-**43b**, and (*R*)-**45b** and (*S*)-**36b**–(*S*)-**38b**, (*S*)-**40b**, (*S*)-**41b**–(*S*)-**43b**, and (*S*)-**45b**, respectively. Ultrasound-assisted synthesis and the catalytic asymmetric route using halohydrin dehalogenase contribute to a more sustainable synthetic approach to obtaining the target compounds.

**Figure 6:**
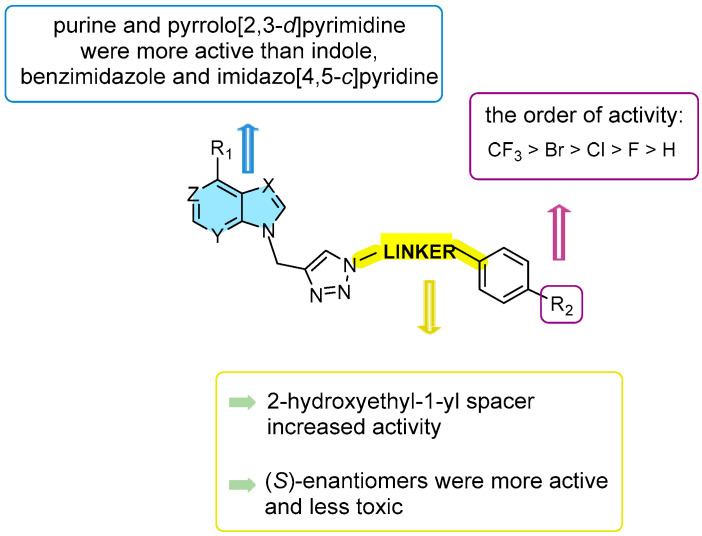
Insight into structure–antiproliferative activity relationship of the racemic and enantioenriched aryl-substituted *N*-heterocycles.

Comparing the influence of the hydroxyethyl linker between 1,2,3-triazole and the aromatic moieties on antiproliferative activity, we may conclude that the linker with the primary hydroxyl group in the regioisomers of the **26a**–**45a** series had a generally higher growth-inhibiting effect on the tested cell lines than the linker with the secondary hydroxyl group in the **26b**–**45b** series (Figure 6).

The only exceptions are imidazo[4,5-*c*]pyridine derivatives, among which compounds **47b–49b**, with a 2-hydroxyeth-2-yl spacer, showed better activity than their analogs **47a–49a**, with a 2-hydroxyeth-1-yl spacer. Overall, regioisomers **26a–45a,** with the primary hydroxyl group, also exhibited a somewhat lower toxicity towards normal fibroblasts (HHF-1) than the regioisomers of the **26b–45b** series. As mentioned before, from a series of regioisomers with a 2-hydroxyeth-2-yl spacer, benzimidazole **34b** and imidazo[4,5-*c*]pyridine **49b** derivatives with 4-bromophenyl moiety displayed the highest inhibitory effect. A comparison of the antiproliferative activity of enantiomeric-enriched 6-chloropurines and 4-chloropyrrolo[2,3-*d*]pyrimidines showed that *S*-enantiomers were more active than the corresponding *R*-enantiomers.

Based on the previous antiproliferative results on purine isosteres with an aryl moiety directly linked to 1,2,3-triazole [43], it can be observed that the introduction of a spacer, especially 2-hydroxyeth-1-yl, between aryl and 1,2,3-triazole, generally improves the activity and reduces the cytotoxic effect on non-cancerous cells. The difference in growth inhibition is more pronounced for indoles, benzimidazoles, and 4-chloropyrrolo[2,3-*d*]pyrimidines than for 6-chloropurines. Similarly to the 6-chloropurine **40a** and the 4-chloropyrrolo[2,3-*d*]pyrimidine **45a** with a 2-hydroxyeth-1-yl spacer, which showed the best antiproliferative activity (**40a**: IC_50_ = 0.69 µM on HT-29**,** IC_50_ = 7.9 µM on HepG2; **45a**: IC_50_ = 0.5 µM on MCF-7; and **40a**: IC_50_ = 1 µM on CFPAC-1), a structural analog with *p*-(trifluoromethyl)phenyl directly connected to 1,2,3-triazole exhibited the best activity (IC_50_ = 7.9 µM on CFPAC-1), albeit high toxicity to normal cells (IC_50_ = 0.75 µM) [43]. Conversely, **40a** and **45b** were found to be less toxic to normal cells than analogs, with aryl directly connected to 1,2,3-trazole, with SIs between 20 and 40.

Evaluation of the ADME properties showed that metabolic stability ranges from high for purine and imidazo[4,5-*c*]pyridine analogs to low for indole and pyrrolo[2,3-*d*]pyrimidine derivatives. Compounds with secondary alcohols are metabolically less stable than compounds with primary alcohols. Indole and pyrrolo[2,3-*d*]pyrimidine derivatives have good permeability, while benzimidazole **34a**, 6-chloropurine **39a**, and imidazo[4,5-*c*]pyridines **49a** and **49b** have low permeability (Papp (AB) ≤ 2 × 10^−6^ cm/s). All compounds are P-glycoprotein (Pgp) substrates, with the exception of compound **29b**.

## 5. Conclusions

A new series of aryl-substituted purine bioisosteres containing a linker with the primary (**26a**–**49a**) and secondary hydroxyl groups (**26b**–**49b**) was synthesized via the ultrasound-assisted Cu(I)-catalyzed Huisgen 1,3-dipolar cycloaddition of terminal alkynyl *N*-heterocyclic derivatives and aryl-substituted 1,2-azido alcohols. The results of antiproliferative profiling showed that among all purine and purine isosteres, purine and 4-chloropyrrolo[2,3-*d*]pyrimidine derivatives with halogen-substituted aromatic residue had better inhibitory activity compared to indole, benzimidazole, and 4-chloroimidazo[4,5-*c*]pyridine derivatives and their unsubstituted analogs. In addition, the introduction of a spacer between aryl and 1,2,3-triazole generally improved the growth-inhibiting activity, showing that the linker with the primary hydroxyl group had a higher cytostatic effect and lower toxicity on normal fibroblasts (HHF-1) than the linker with the secondary hydroxyl group. *S*-enantiomers of 6-chloropurines and 4-chloropyrrolo[2,3-*d*]pyrimidines were more active than the corresponding *R*-enantiomers. We can conclude that purine **40a** and 4-chloropyrrolo[2,3-*d*]pyrimidine **45a**, both with 4-(trifluoromethyl)phenyl moiety and the primary hydroxyl group, showed the most significant inhibitory effect (**40a**: IC_50_ = 0.69 µM on HT-29, IC_50_ = 0.64 µM on HepG2; **45a**: IC_50_ = 0.5 µM on MCF-7 cell line). The best selectivity compared to the non-tumor cell line was observed for 4-chloropyrrolo[2,3-*d*]pyrimidine **45a**. Compound **45a** has moderate clearance and good permeability, which makes it quite interesting in combination with promising bioactivity and the selectivity profile. Further studies are required to elucidate the mechanism of action of **45a** as a promising candidate. However, the active efflux has a negative influence on the cellular concentration, indicating a further need for the multi-parametric optimization of the PhysChem and ADME properties.

## Figures and Tables

**Table 1:** Regioselectivity and conversion of ring-opening reaction of 2-phenyloxirane derivatives via NaN_3_ under basic conditions ^a^.

R_2_	Regioisomers	Conversion (%)	Regioselectivity (*α*/*β*)
H	**16a**/**16b**	96%	15.6
F	**17a**/**17b**	71%	4.5
Cl	**18a**/**18b**	68%	5.2
Br	**19a**/**19b**	87%	2.1
CF_3_	**20a**/**20b**	85%	1.5

^a^ Reaction conditions: NaN_3_ (1.2 eq) and NH_4_Cl (1.0 eq) in MeOH/H_2_O (8:1), 75 °C, overnight.

**Table 2:** The growth inhibition effects of racemic indole (**26a,b**–**30a,b**) and benzimidazole derivatives (**31a,b**–**35a,b**) on MCF-7, CFPAC-1, HT-29, and HepG2, as well as on normal skin fibroblasts (HFF-1).

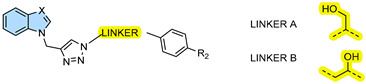									
				IC_50_^a^ (µM)					
Compd	X	Linker	R_2_	MCF-7	CFPAC-1 ^b^	HT-29	HepG2	HFF-1	SI (CFPAC-1) ^c^
**26a**	CH	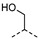	H	>100	>100	>100	>100	>100	-
**26b**	CH	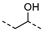	H	>100	>100	>100	>100	>100	-
**27a**	CH	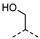	F	51 ± 6.7	68 ± 12.7	47 ± 16.9	49 ± 2.5	42 ± 10.5	0.6
**27b**	CH	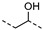	F	>100	>100	>100	>100	>100	-
**28a**	CH	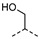	Cl	32 ± 0.0	30 ± 0.6	31 ± 0.9	32 ± 0.2	42 ± 4.6	1.4
**28b**	CH	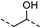	Cl	35 ± 16.2	35 ± 19.8	46 ± 18.4	66 ± 2.7	>100	>2.9
**29a**	CH	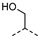	Br	24 ± 1.0	39 ± 2.8	34 ± 0.3	29 ± 0.8	>100	>2.6
**29b**	CH	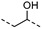	Br	29 ± 14	43 ± 9.1	36 ± 1.4	35 ± 1.8	25 ± 4.1	0.6
**30a**	CH	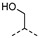	CF_3_	21 ± 1.6	9 ± 3.3	28 ± 1.9	32 ± 3.6	34 ± 1.6	3.7
**30b**	CH	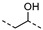	CF_3_	22 ± 3.4	27 ± 0.7	51 ± 15.3	65 ± 2.4	18 ± 0.6	0.7
**31a**	N	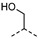	H	>100	>100	>100	>100	>100	-
**31b**	N	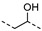	H	91 ± 7.2	>100	>100	>100	>100	-
**32a**	N	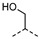	F	98 ± 2.8	>100	>100	74 ± 0.6	>100	-
**32b**	N	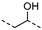	F	>100	>100	>100	>100	>100	-
**33a**	N	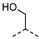	Cl	40 ± 1.8	34 ± 2.3	63 ± 9.8	50 ± 12.8	>100	>2.9
**33b**	N	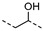	Cl	70 ± 20.5	69 ± 12.1	>100	>100	64 ± 6.17	0.9
**34a**	N	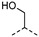	Br	22 ± 5.1	15 ± 8.9	39 ± 4.3	35 ± 14.2	42 ± 8.3	2.8
**34b**	N	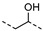	Br	39 ± 10.1	8 ± 3.2	70 ± 14.5	64 ± 19.5	32 ± 14.1	4.0
**35a**	N	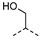	CF_3_	30 ± 0.9	27 ± 0.7	61 ± 0.7	59 ± 4.6	>100	>3.7
**35b**	N	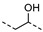	CF_3_	31 ± 13.7	36 ± 1.3	67 ± 2.9	67 ± 3.5	22 ± 18.1	0.6
5-FU				1 ± 0.8	0.14 ± 0.1	0.23 ± 0.0	9 ± 1.3	0.94 ± 0.0	6.7

^a^ IC_50_—compound concentration that inhibited cell growth by 50%. Data represent mean IC_50_ (μM) values ± standard deviation (SD) of three independent experiments. ^b^ Indole analogs of **27**, **28**, and **30** without linker exhibited IC_50_ > 100 μM (CFPAC-1), while benzimidazole analogs of **32**, **33**, and **35** exhibited IC_50_ values of 86.52 μM, 52.55 μM, and 56.43 μM, respectively (CFPAC-1). ^c^ Selectivity index towards ductal pancreatic adenocarcinoma cells (CFPAC-1), SI = [IC_50_ HFF]/[IC_50_ CFPAC-1].

**Table 3:** The growth inhibition effects of racemic and enantiomeric-enriched 6-chloropurine derivatives (**36a,b**–**40a,b**, (*R*),(*S*)-**36b**-(*R*),(*S*)-**38b** and (*R*),(*S*)-**40b**) on MCF-7, CFPAC-1, HT-29, and HepG2, as well as on normal skin fibroblasts (HFF-1).

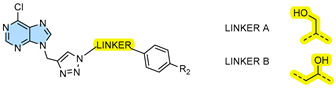									
			IC_50_^a^ (µM)						
Compd	Linker	R_2_	MCF-7	CFPAC-1 ^b^	HT-29	HepG2	HFF-1	SI (HT-29) ^c^	SI (HepG2) ^c^
**36a**	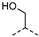	H	53 ± 5.5	60 ± 0.9	54 ± 7.4	55 ± 2.4	>100	>1.9	>1.8
**36b**	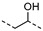	H	69 ± 10.6	65 ± 18.9	61 ± 2.4	41 ± 10.6	80 ± 14.0	1.3	1.9
(*R*)-**36b**	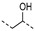	H	61 ± 3.7	66 ± 3.2	86 ± 11.6	57 ± 9.7	>100	>1.2	>1.8
(*S*)-**36b**	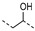	H	33 ± 5.8	32 ± 5.3	48 ± 4.8	21 ± 0.8	>100	>2.1	>4.8
**37a**	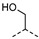	F	38 ± 15.7	50 ± 12.4	41 ± 2.3	37 ± 12.9	64 ± 6.3	1.6	1.7
**37b**	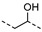	F	49 ± 9.9	56 ± 5.5	46 ± 9.9	54 ± 6.0	59 ± 9.2	1.3	1.1
(*R*)-**37b**	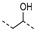	F	>100	>100	>100	64 ± 3.1	35 ± 5.5	0.4	0.6
(*S*)-**37b**	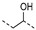	F	73 ± 2.9	65 ± 5.4	24 ± 2.9	45 ± 1.9	70 ± 19.5	2.9	1.6
**38a**	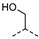	Cl	24 ± 8.8	48 ± 4.9	33 ± 6.06	34 ± 4.2	41 ± 16.3	1.2	1.2
**38b**	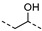	Cl	51 ± 8.9	36 ± 8.2	56 ± 2.8	49 ± 13.3	31 ± 5.3	0.6	0.6
(*R*)-**38b**	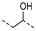	Cl	64 ± 4.8	60 ± 10.3	51 ± 17.8	39 ± 10.5	31 ± 13.4	0.6	0.8
(*S*)-**38b**	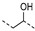	Cl	58 ± 1.9	51 ± 11.7	47 ± 16.8	28 ± 7.5	81 ± 1.8	1.7	2.9
**39a**	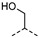	Br	14 ± 4.8	46 ± 3.8	31 ± 2.6	16 ± 5.6	14 ± 4.7	0.5	0.9
**39b**	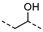	Br	21 ± 14.0	15 ± 1.0	50 ± 0.6	44 ± 8.1	30 ± 2.7	0.6	0.7
**40a**	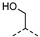	CF_3_	19 ± 4.3	18 ± 3.5	0.69 ± 0.01	0.64 ± 0.1	18 ± 0.6	26.1	28.1
**40b**	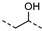	CF_3_	28 ± 6.7	25 ± 6.4	14 ± 8.1	15 ± 9.0	8 ± 4.8	0.6	0.5
(*R*)-**40b**	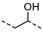	CF_3_	36 ± 23.1	16 ± 2.6	6 ± 0.6	8 ± 0.6	6 ± 4.8	1.0	0.7
(*S*)-**40b**	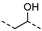	CF_3_	10 ± 0.4	19 ± 2.4	5 ± 0.1	7 ± 1.5	12 ± 3.4	2.4	1.7
5-FU			1 ± 0.8	0.1 ± 0.1	0.2 ± 0.0	9 ± 1.3	0.9 ± 0.0	4.5	0.1

^a^ IC_50_—compound concentration that inhibited cell growth by 50%. Data represent mean IC_50_ (μM) values ± standard deviation (SD) of three independent experiments, ^b^ 6-Chloropurine analogs of **37** and **40** without linker exhibited IC_50_ values of 33.39 μM and 7.90 μM, respectively (CFPAC-1). ^c^ Selectivity index towards colorectal carcinoma (HT-29) SI = [IC_50_ HFF]/[IC_50_ HT-29] and hepatocellular carcinoma (HepG2), SI = [IC_50_ HFF]/[IC_50_ HepG2].

**Table 4:** The growth inhibition effects of racemic and enantiomeric-enriched 4-chloropyrrolo[2,3-*d*]pyrimidine derivatives (**41a,b**–**45a,b**, ((*R*),(***S***)-**41b**-(*R*),(S)-**43b** and (*R*),(*S*)-**45b**)) on MCF-7, CFPAC-1, HT-29, and HepG2, as well as on normal skin fibroblasts (HFF-1).

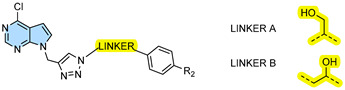									
			IC_50_^a^ (µM)						
Compd	Linker	R_2_	MCF-7	CFPAC-1 ^b^	HT-29	HepG2	HFF-1	SI (MCF-7) ^c^	SI (CFPAC-1) ^c^
**41a**	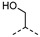	H	39 ± 7.1	33 ± 4.5	33 ± 3.2	38 ± 1.8	62 ± 23.3	1.6	1.9
**41b**	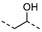	H	44 ± 17.4	49 ± 11.3	50 ± 1.3	50 ± 10.2	44 ± 14.1	1.0	0.9
(*R*)-**41b**	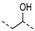	H	46 ± 2.7	58 ± 12.1	46 ± 2.3	52 ± 2.7	23 ± 5.8	0.5	0.4
(*S*)-**41b**	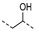	H	45 ± 7.2	49 ± 18.5	41 ± 5.6	41 ± 12.4	58 ± 3.1	1.3	1.2
**42a**	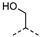	F	39 ± 16.9	36 ± 2.2	39 ± 1.9	47 ± 13.5	51 ± 16.5	1.3	1.4
**42b**	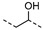	F	45 ± 5.7	34 ± 3.2	49 ± 14.6	38 ± 11.0	51 ± 3.6	1.1	1.5
(*R*)-**42b**	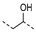	F	60 ± 1.2	20 ± 0.9	27 ± 1.4	32 ± 6.04	24 ± 2.4	0.4	1.2
(*S*)-**42b**	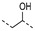	F	41 ± 0.0	13 ± 2.7	33 ± 0.2	49 ± 2.0	73 ± 2.4	1.8	5.6
**43a**	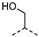	Cl	24 ± 8.8	8 ± 3.2	33 ± 6.1	33 ± 4.2	41 ± 16.3	1.7	5.1
**43b**	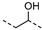	Cl	39 ± 10.1	48 ± 4.9	28 ± 3.8	46 ± 2.2	32 ± 14.1	0.8	0.7
(*R*)-**43b**	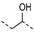	Cl	35 ± 1.9	32 ± 16.1	48 ± 4.8	26 ± 8.2	20 ± 6.5	0.6	0.6
(*S*)-**43b**	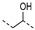	Cl	33 ± 5.8	31 ± 5.3	33 ± 7.8	21 ± 0.8	41 ± 1.1	1.2	1.3
**44a**	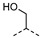	Br	79 ± 17.6	69 ± 2.0	87 ± 9.9	83 ± 28.3	79 ± 17.6	1.0	1.1
**44b**	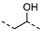	Br	25 ± 6.4	15 ± 0.2	30 ± 0.7	32 ± 3.2	27 ± 11.9	1.1	1.8
**45a**	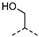	CF_3_	0.5 ± 0.1	1 ± 0.7	26 ± 1.2	28 ± 0.5	20 ± 4.7	40.0	20.0
**45b**	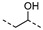	CF_3_	21 ± 1.6	13 ± 2.2	19 ± 7.5	32 ± 16.1	12 ± 3.1	0.6	0.9
(*R*)-**45b**	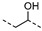	CF_3_	43 ± 4.6	49 ± 8.7	45 ± 4.0	38 ± 7.6	21 ± 3.8	0.5	0.4
(*S*)-**45b**	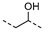	CF_3_	26 ± 8.5	43 ± 10.8	31 ± 2.2	23 ± 2.3	43 ± 3.9	1.7	1.0
5-FU			1 ± 0.8	0.1 ± 0.1	0.2 ± 0.0	9 ± 1.3	0.9 ± 0.0	0.9	9.0

^a^ IC_50_—compound concentration that inhibited cell growth by 50%. Data represent mean IC_50_ (μM) values ± standard deviation (SD) of three independent experiments, ^b^ 4-chloropyrrolo[2,3-*d*]pyrimidine analogs of **42**, **43**, and **45** without linker exhibited IC_50_ values of 48.37 μM, 47.69 μM, and 46.55 μM, respectively (CFPAC-1). ^c^ Selectivity index towards human breast adenocarcinoma cells (MCF-7), SI = [IC_50_ HFF]/[IC_50_ MCF-7] and towards ductal pancreatic adenocarcinoma cells (CFPAC-1), SI = [IC_50_ HFF]/[IC_50_ CFPAC-1].

**Table 5:** The growth inhibition effects of racemic 4-chloro-1*H*-imidazo[4,5-*c*]pyridine derivatives (**46a,b**–**49a,b**) on MCF-7, CFPAC-1, HT-29, and HepG2, as well as on normal skin fibroblasts (HFF-1).

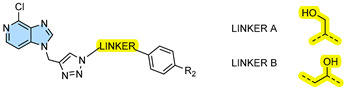								
			IC_50_^a^ (µM)					
Compd	Linker	R_2_	MCF-7	CFPAC-1	HT-29	HepG2	HFF-1	SI (HepG2) ^b^
**46a**	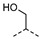	H	>100	>100	>100	>100	>100	-
**46b**	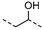	H	>100	>100	>100	>100	>100	-
**47a**	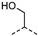	F	63 ± 8.4	51 ± 6.5	>100	>100	75 ± 14.2	0.8
**47b**	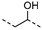	F	21 ± 2.6	18 ± 0.8	65 ± 12.6	55 ± 8.8	18 ± 2.9	0.3
**48a**	** 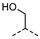 **	Cl	96 ± 1.8	>100	>100	>100	>100	-
**48b**	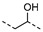	Cl	>100	>100	>100	>100	>100	-
**49a**	** 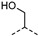 **	Br	77 ± 3.0	77 ± 0.5	77 ± 1.1	66 ± 22.8	90 ± 4.04	1.4
**49b**	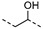	Br	46 ± 0.0	21 ± 0.7	10 ± 3.2	4 ± 0.0	21 ± 5.8	5.3
5-FU			1 ± 0.8	0.1 ± 0.1	0.2 ± 0.0	9 ± 1.3	0.9 ± 0.0	0.1

^a^ IC_50_—compound concentration that inhibited cell growth by 50%. Data represent mean IC_50_ (μM) values ± standard deviation (SD) of three independent experiments. ^b^ Selectivity index towards hepatocellular carcinoma (HepG2), SI = [IC_50_ HFF]/[IC_50_ HepG2].

**Table 6:** Calculated structural and PhysChem parameters, as well as measured permeability and metabolic stability of representative compounds.

Compd	LogP	TPSA ^a^	HBD ^b^	HBA ^c^	Solubility pH = 7.4 Water	%PPB ^d^	CYP3A4 Inhibition ^e^	Clearance Values (CL) ^f^	Clearance Class.	P_app_ (AB) [×10^−6^ cm/s]	P_app_ (BA) [×10^−6^ cm/s]	Efflux Ratio ^g^	Permeability
**29a**	3.11	55.87	1	5	highly insoluble	98.66	0.35	43	MODERATE	10.0	25.5	2.6	HIGH
**29b**	3.16	55.87	1	5	insoluble	97.32	0.41	92	HIGH	18.3	28.6	1.7	HIGH
**34a**	2.25	68.76	1	6	soluble	98.7	0.40	<30	LOW	2.0	65.7	32.8	LOW
**34b**	2.44	68.76	1	6	soluble	95.59	0.42	48	MODERATE	4.3	69.4	17.2	MODERATE
**39a**	1.85	94.54	1	8	very soluble	95.74	0.59	<30	LOW	0.9	58.0	68.1	LOW
**39b**	1.78	94.54	1	8	soluble	91.26	0.74	<30	LOW	3.1	69.6	22.5	MODERATE
**40a**	1.7	94.54	1	8	insoluble	98.4	0.72	<30	LOW	2.1	60.6	29.5	MODERATE
(*R*)-**40b**	1.79	94.54	1	8	soluble	96.98	0.68	<30	LOW	2.2	45.2	21.0	MODERATE
(*S*)-**40b**	1.79	94.54	1	8	soluble	96.98	0.68	<30	LOW	2.2	42.9	19.5	MODERATE
**44a**	2.4	81.65	1	7	insoluble	98.46	0.77	56	MODERATE	10.9	67.4	6.2	HIGH
**44b**	2.53	81.65	1	7	insoluble	97.35	0.81	84	HIGH	25.3	62.7	2.5	HIGH
**45a**	2.26	81.65	1	7	insoluble	99.4	0.76	54	MODERATE	7.6	66.4	8.8	MODERATE
**45b**	2.53	81.65	1	7	insoluble	98.84	0.75	90	HIGH	26.5	90.7	3.4	HIGH
**49a**	2.55	81.65	1	7	very soluble	98.04	0.58	<30	LOW	1.0	41.1	41.4	LOW
**49b**	2.54	81.65	1	7	soluble	96.6	0.66	<30	LOW	1.6	36.9	23.3	LOW

^a^ TPSA—total polar surface area; ^b^ HBD—hydrogen bond donor; ^c^ HBA—hydrogen bond acceptor; ^d^ PPB—plasma protein binding; ^e^ probability of CYP3A4 inhibition less than IC_50_ < 10 µM; ^f^ predicted in vivo hepatic clearance; CL: <30% low, 30–70% moderate, and >70% high**.** ^g^ Efflux ratio: <2 no efflux, >2 active efflux.

## Data Availability

Data that support the findings of this study are available in the Supporting Information of this article.

## Supplementary Materials

The following supporting information can be downloaded at: https://www.mdpi.com/article/10.3390/biom15030351/s1. Figures S1–S49: ^1^H, ^13^C NMR, and UV-Vis spectra of novel compounds.
