# Supplementary material for: Synthesis, Antiproliferative Activity, and ADME Profiling of Novel Racemic and Optically Pure Aryl-Substituted Purines and Purine Bioisosteres
Source: Biomolecules. 2025 Feb 28;15(3):351. doi: 10.3390/biom15030351 (PMC11940194; doi:10.3390/biom15030351)
Supplement: Supplementary file 1 [file biomolecules-15-00351-s001.zip › biomolecules-3456485-supplementary.pdf]

SUPPLEMENTARY INFORMATION

Synthesis, antiproliferative activity and ADME profiling of novel racemic and optically pure aryl-substituted purines and purine bioisosteres

Martina Piškor[1], Astrid Milić[2], Sanja Koštrun[2], Maja Majerić Elenkov[3], Petra Grbčić[4], Sandra Kraljević, Krešimir Pavelić[4], Silvana Raić-Malić[1,*]

[1] Department of Organic Chemistry, University of Zagreb, Faculty of Chemical Engineering and Technology, Marulićev trg 19, 10000 Zagreb, Croatia; mpiskor@fkit.unizg.hr (M.P.); sraic@fkit.unizg.hr (S.R.M.)
[2] Selvita d.o.o., Zagreb 10000, Croatia; Sanja.Kostrun@selvita.com (S.K.), Astrid.Milic@selvita.com (A.M.)
[3] Division of Organic Chemistry and Biochemistry, Ruder Bošković Institute, Bijenička 54, 10000 Zagreb, Croatia; Maja.Majeric-Elenkov@irb.hr (M.M.E.)
[4] Juraj Dobrila University of Pula, Faculty of Medicine, 52100 Pula, Croatia; petra.grbcic@unipu.hr.(P.G.); pavelic@unipu.hr (K.P.)
[5] Faculty of Health Studies, University of Rijeka, Viktora Cara Emina 5, 51000 Rijeka, Croatia; sandra.kraljevic.pavelic@fzsri.uniri.hr (S.K.P)
* Correspondence: sraic@fkit.unizg.hr

Contents

1. NMR spectra of novel compounds 1

2. UV/Vis absorption spectra of selected compounds 49

1. NMR spectra of novel compounds

Fig. S1 a) ^{1}H NMR and b) ^{13}C NMR of compd. **26a**

a)

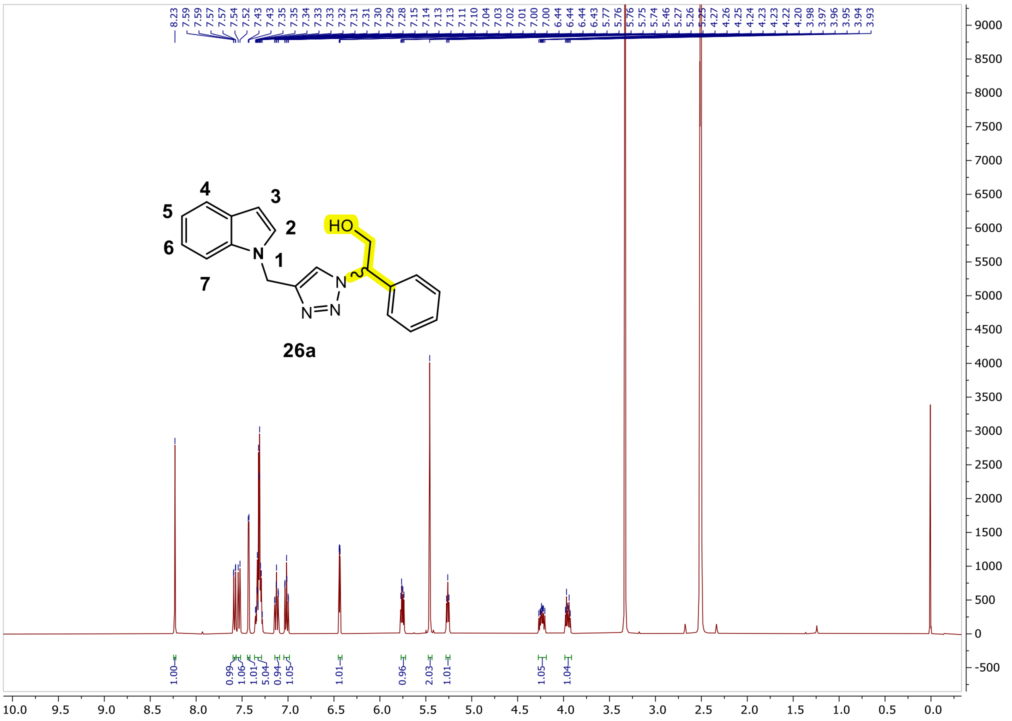

b)

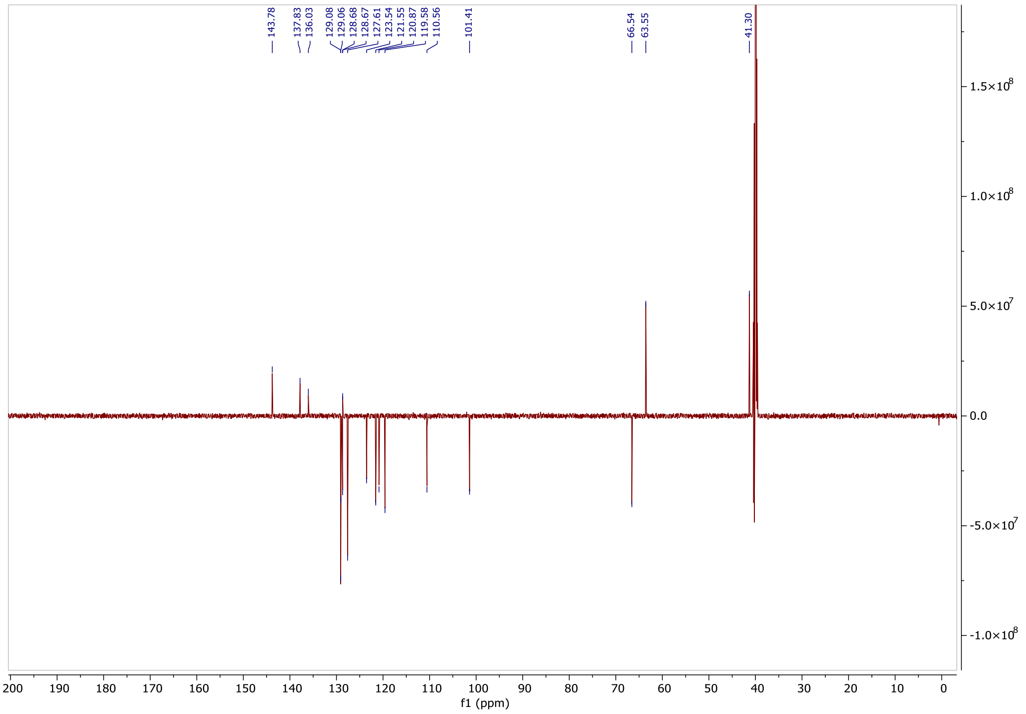

Fig. S2 a) ^{1}H NMR and b) ^{13}C NMR of compd. **27a**

a)

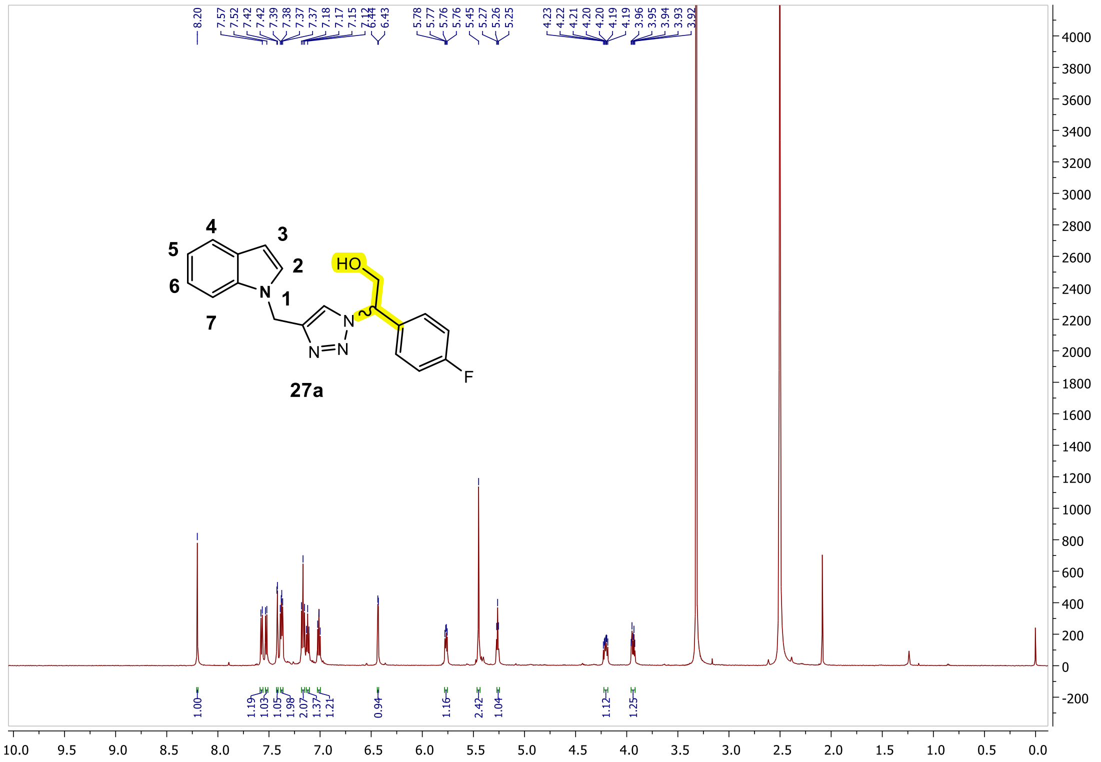

b)

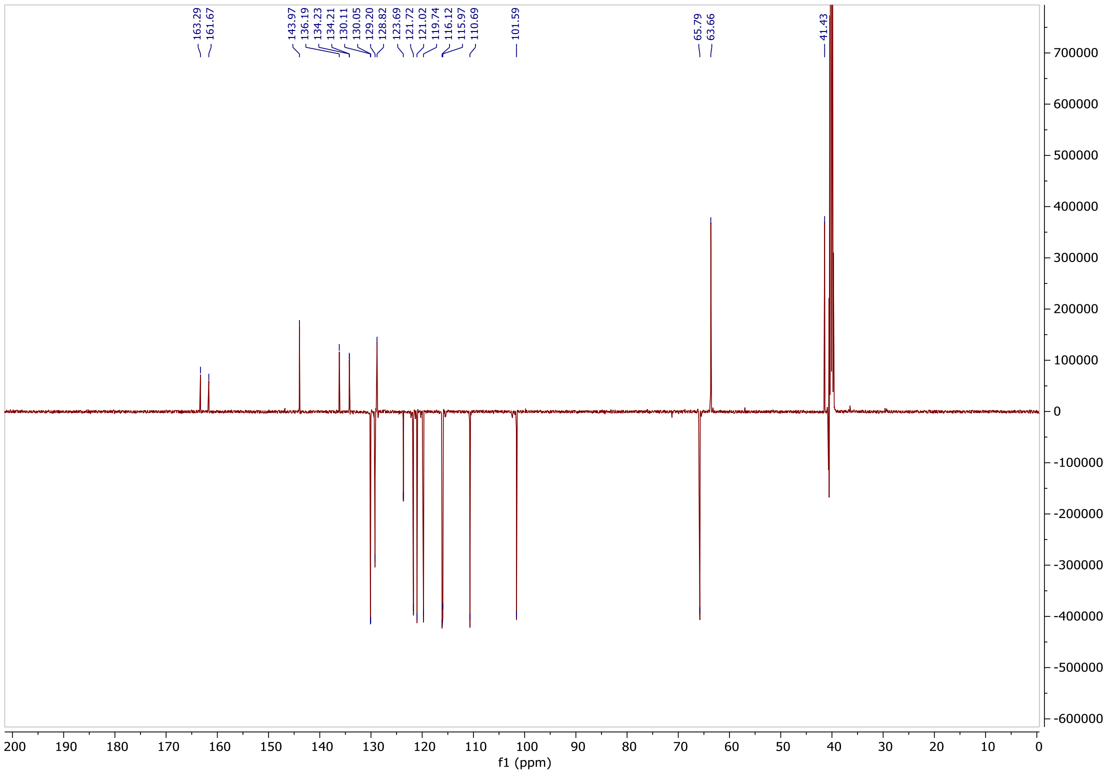

Fig. S3 a) ^{1}H NMR and b) ^{13}C NMR of compd. **28a**

a)

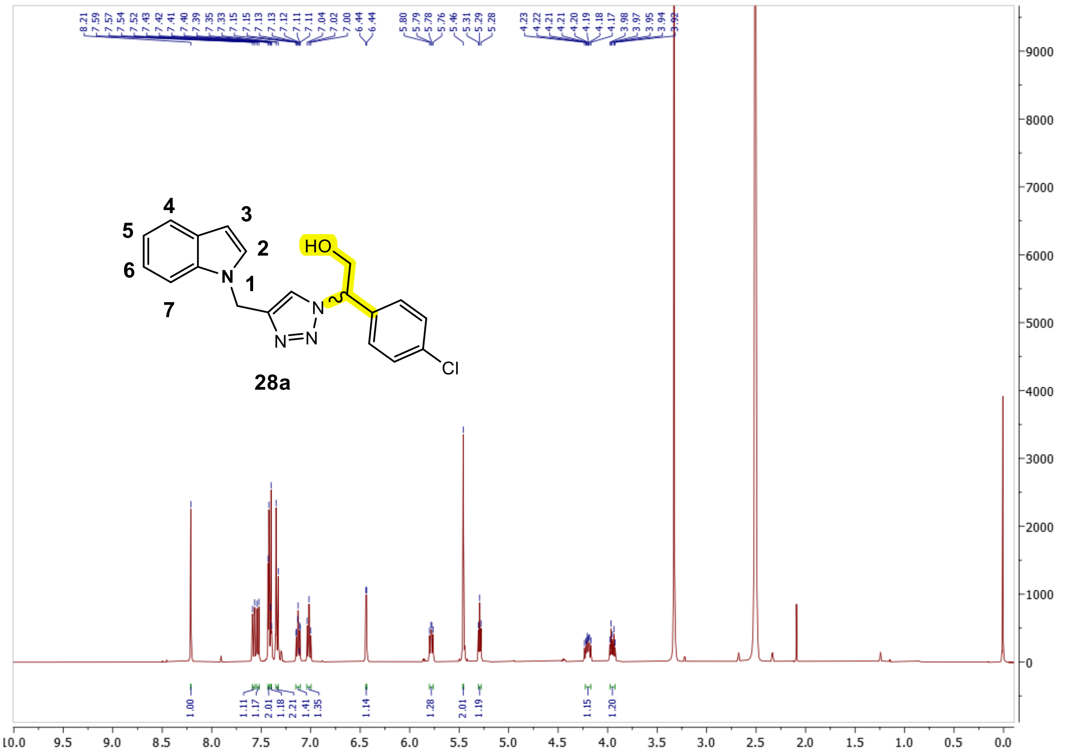

b)

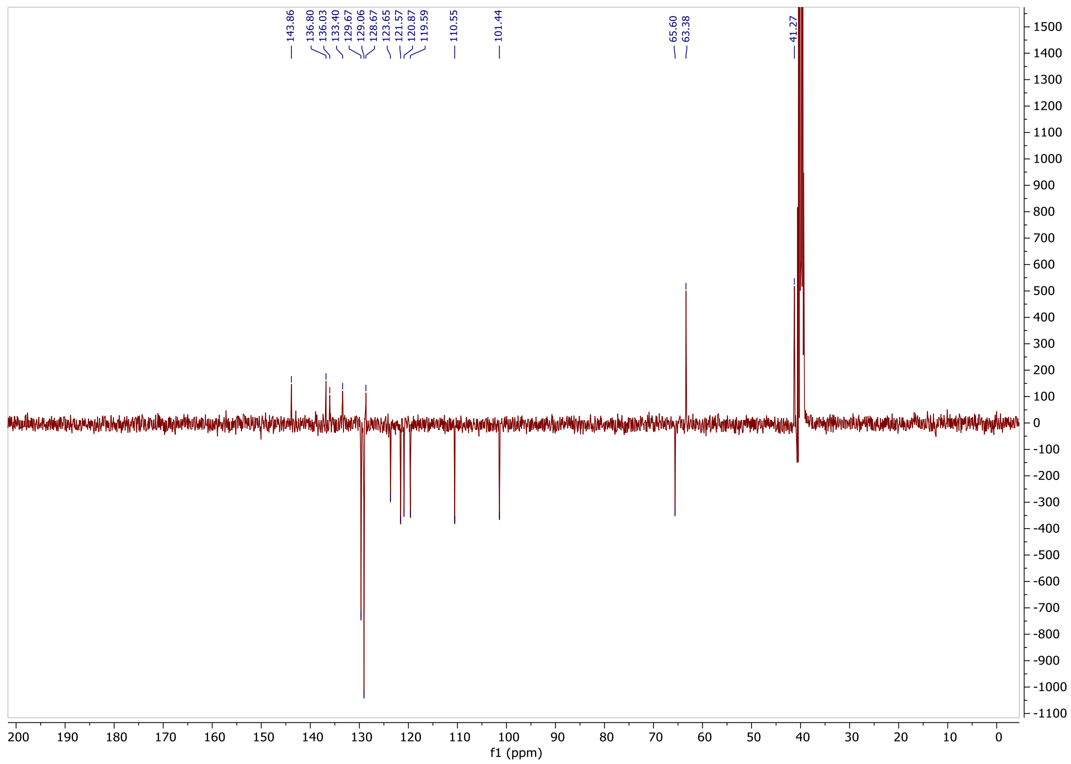

Fig. S4 a) ^{1}H NMR and b) ^{13}C NMR of compd. **29a**

a)

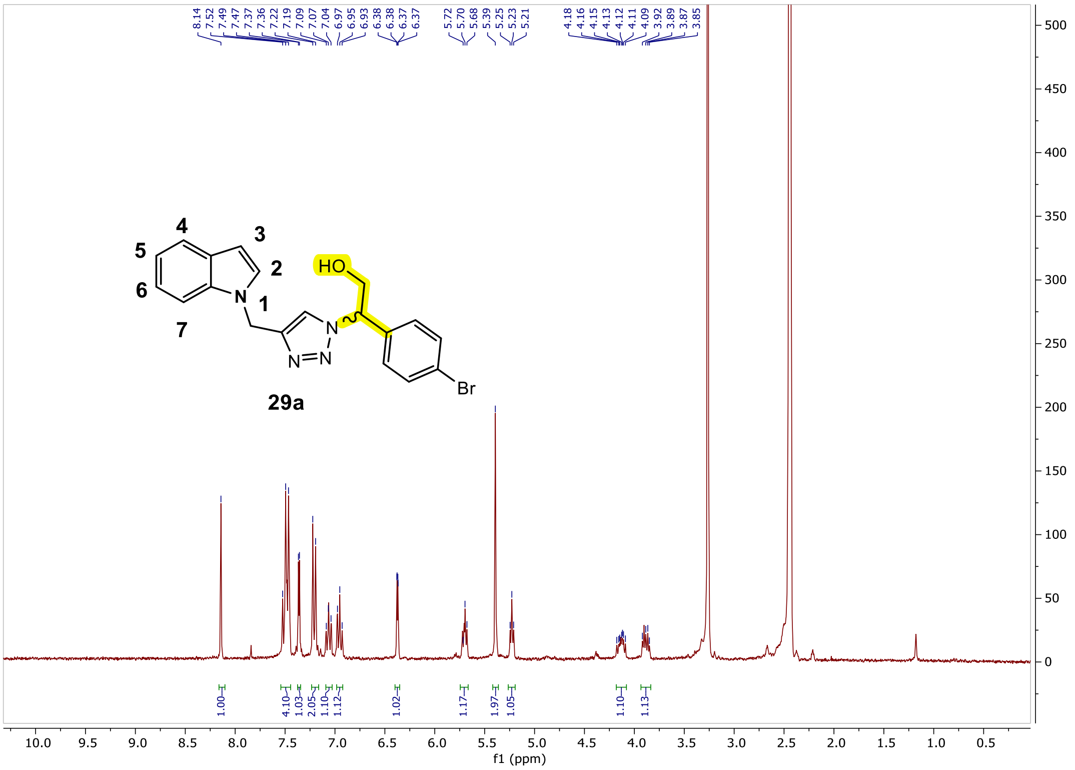

b)

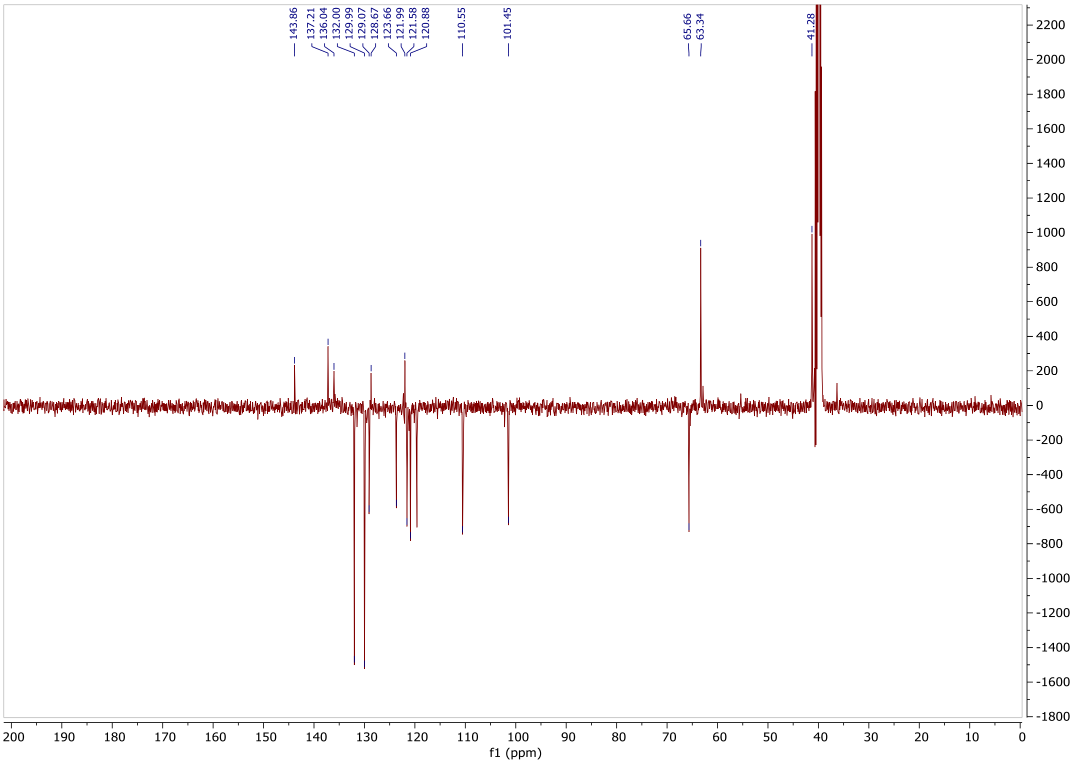

Fig. S5 a) ^{1}H NMR and b) ^{13}C NMR of compd. **30a**

a)

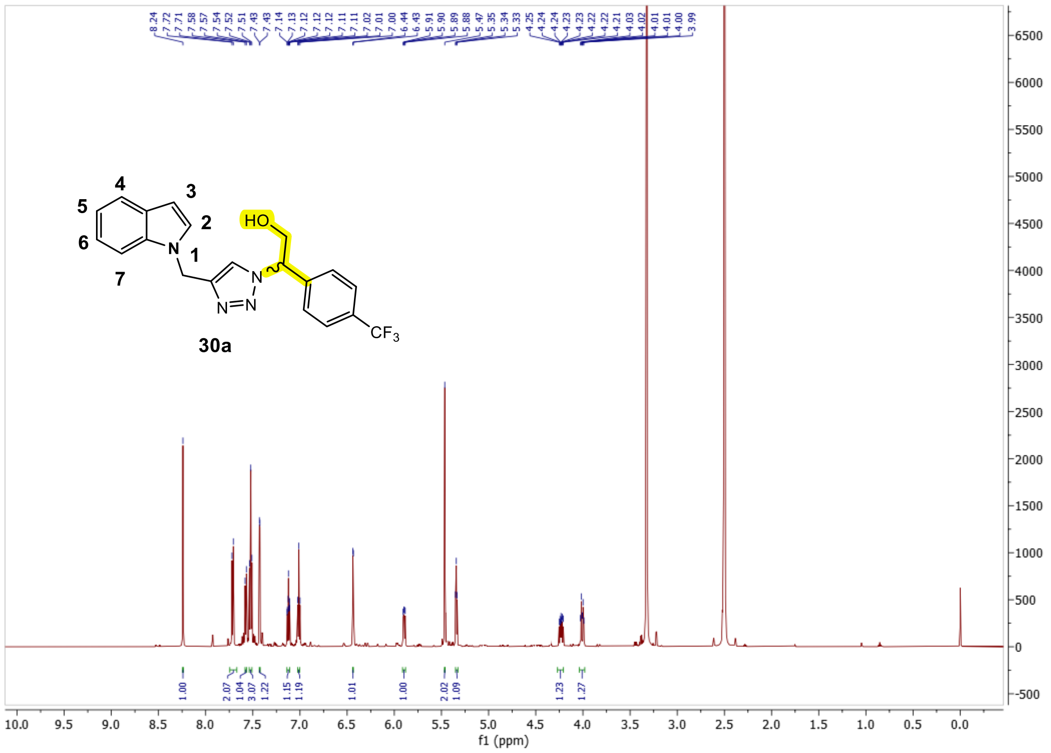

b)

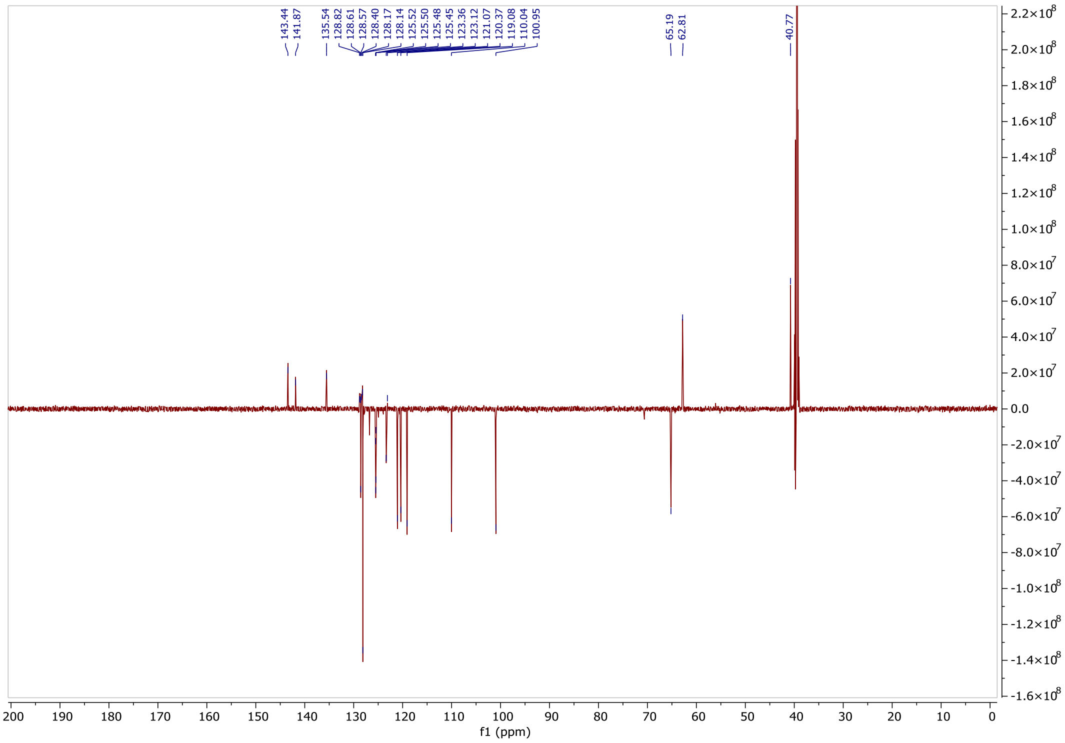

Fig. S6 a) ^{1}H NMR and b) ^{13}C NMR of compd. **31a**

a)

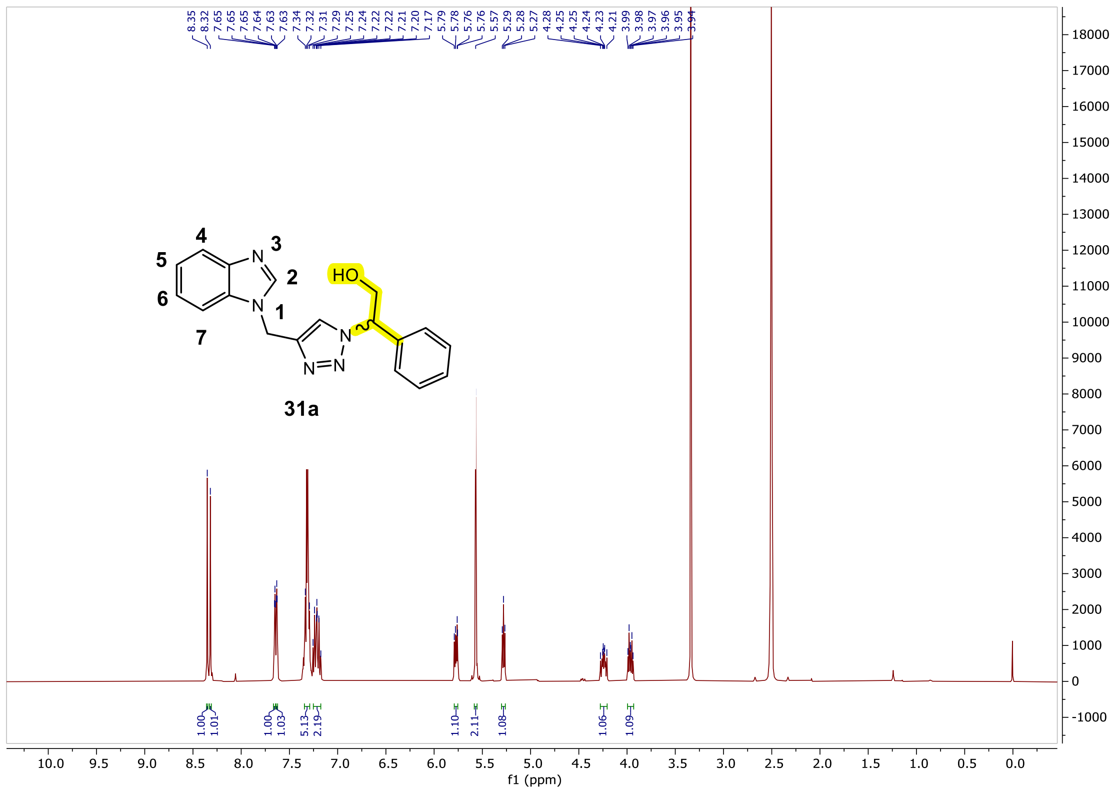
8.35
8.32
7.65
7.65
7.64
7.63
7.63
7.34
7.32
7.31
7.29
7.25
7.24
7.22
7.22
7.21
7.20
7.17
5.79
5.78
5.76
5.57
5.29
5.28
5.27
4.28
4.25
4.25
4.24
4.23
4.21
3.99
3.98
3.97
3.96
3.95
3.94
4
3
5
2
HO
6
1
7
31a
1.00
1.01
1.00
1.03
5.13
2.19
1.10
2.11
1.08
1.06
1.09
f1 (ppm)

b)

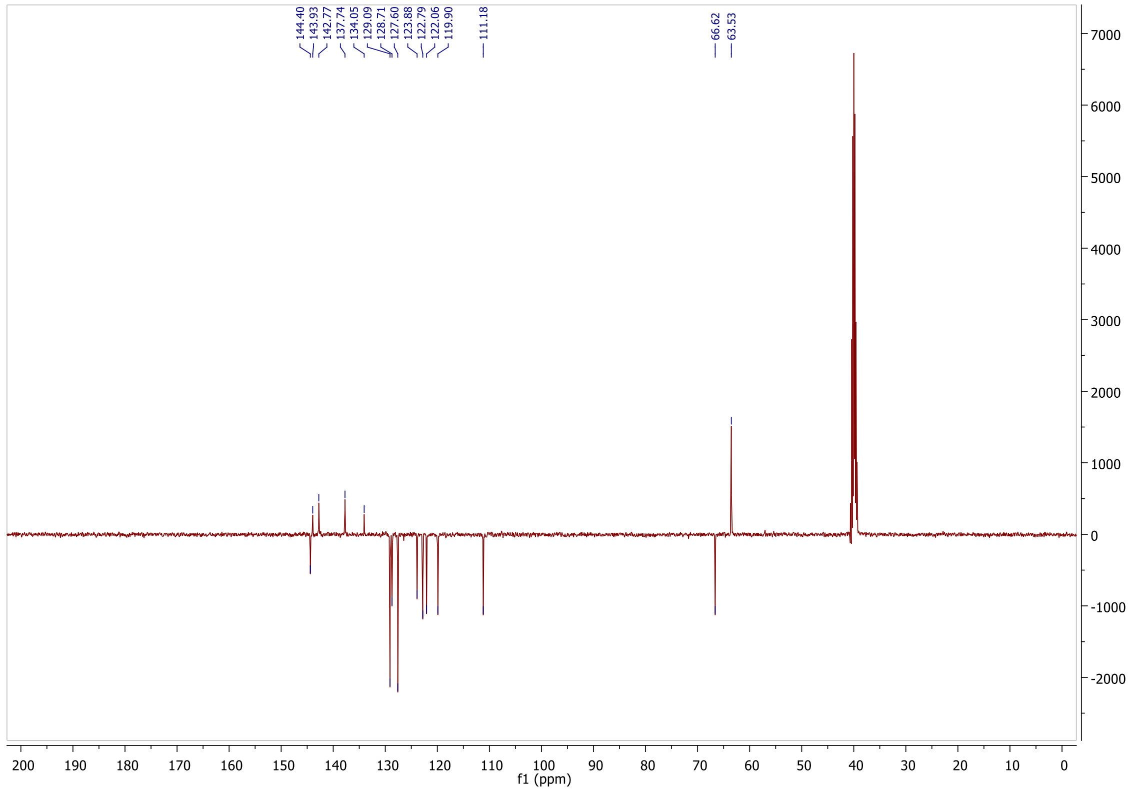
144.40
143.93
142.77
137.74
134.05
129.09
128.71
127.60
123.88
122.79
122.06
119.90
111.18
66.62
63.53
f1 (ppm)

Fig. S7 a) ^{1}H NMR and b) ^{13}C NMR of compd. **32a**

a)

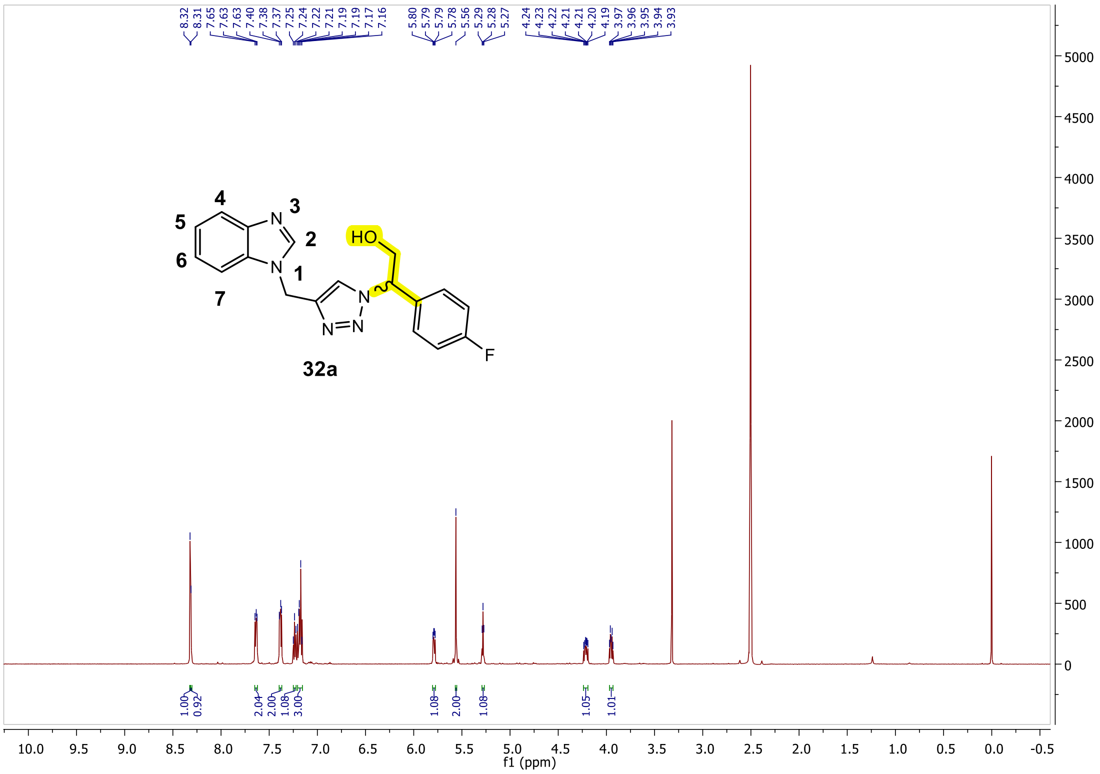

b)

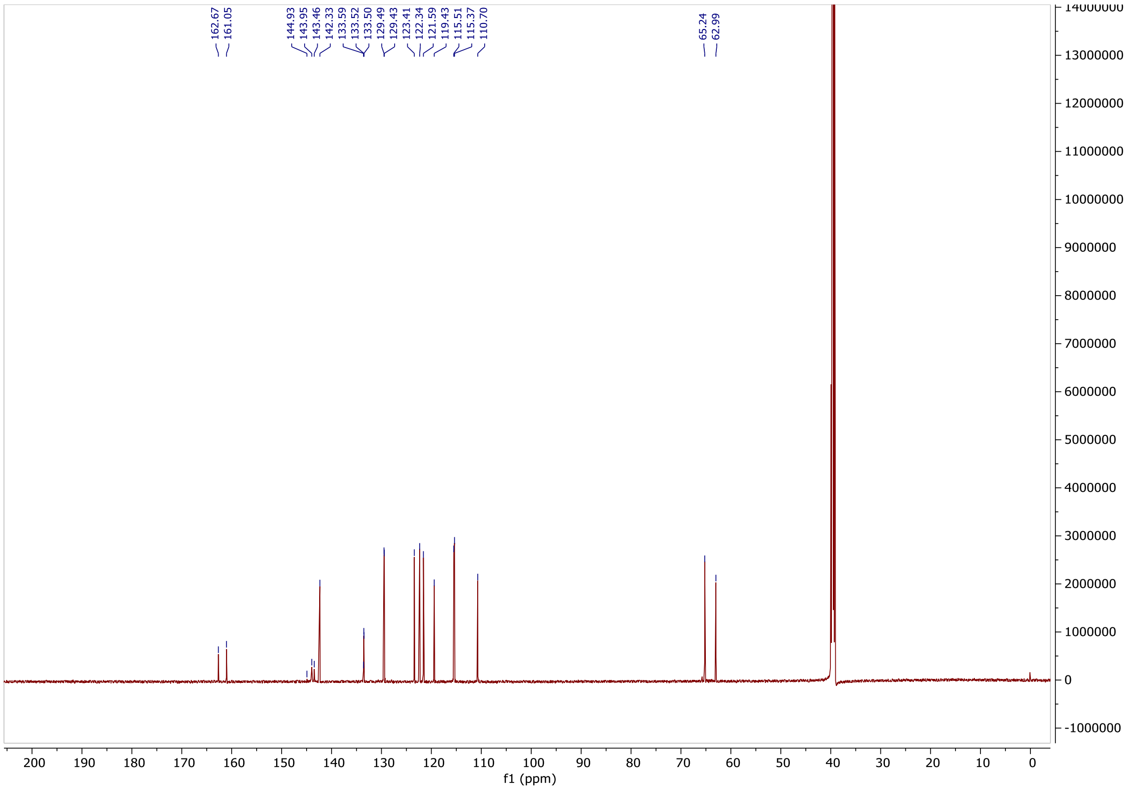

Fig. S8 a) ^{1}H NMR and b) ^{13}C NMR of compd. **33a**

a)

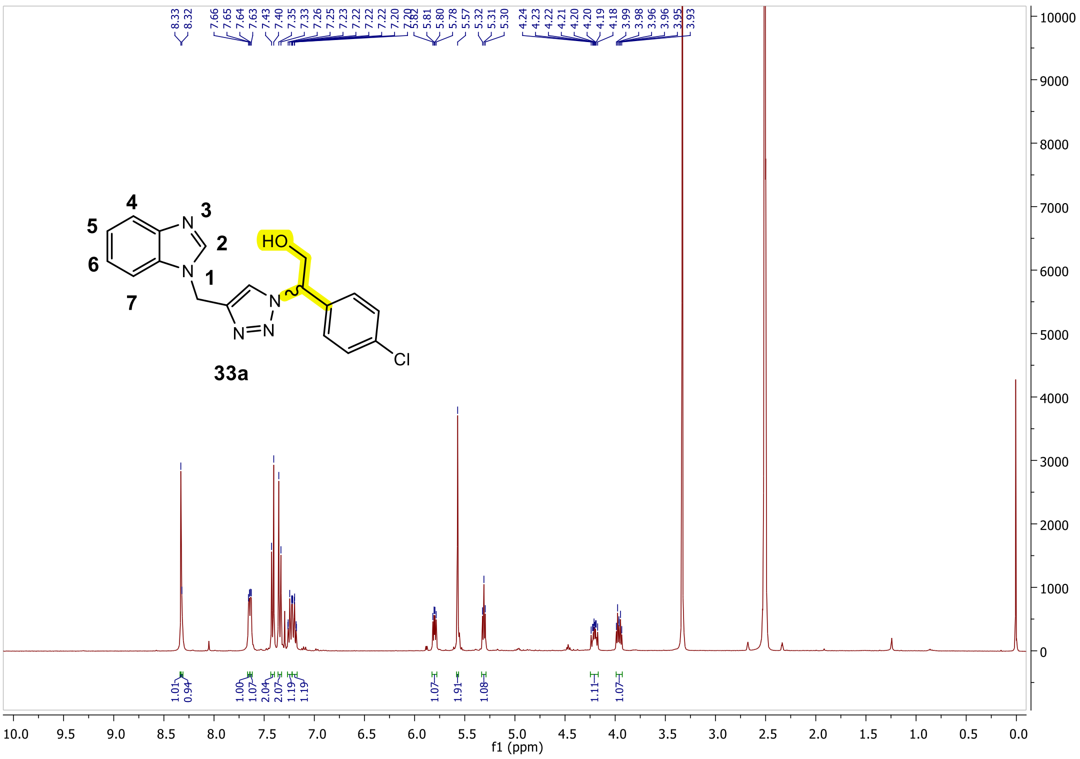

b)

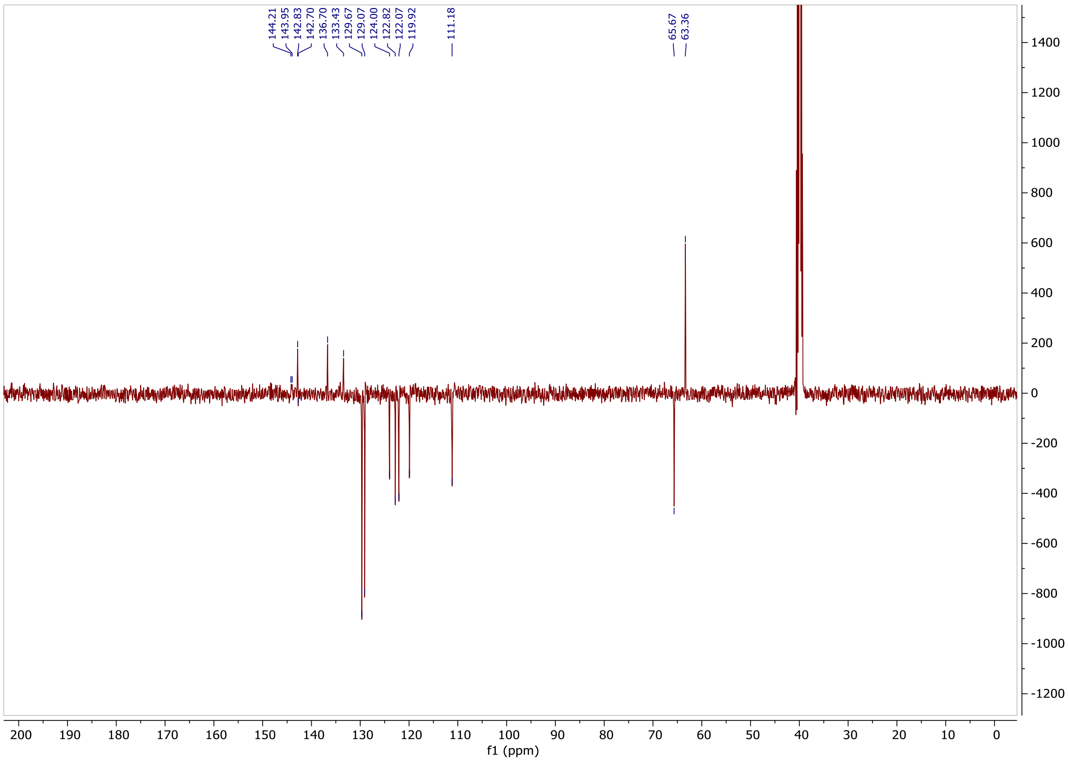

Fig. S9 a) ^{1}H NMR and b) ^{13}C NMR of compd. **34a**

a)

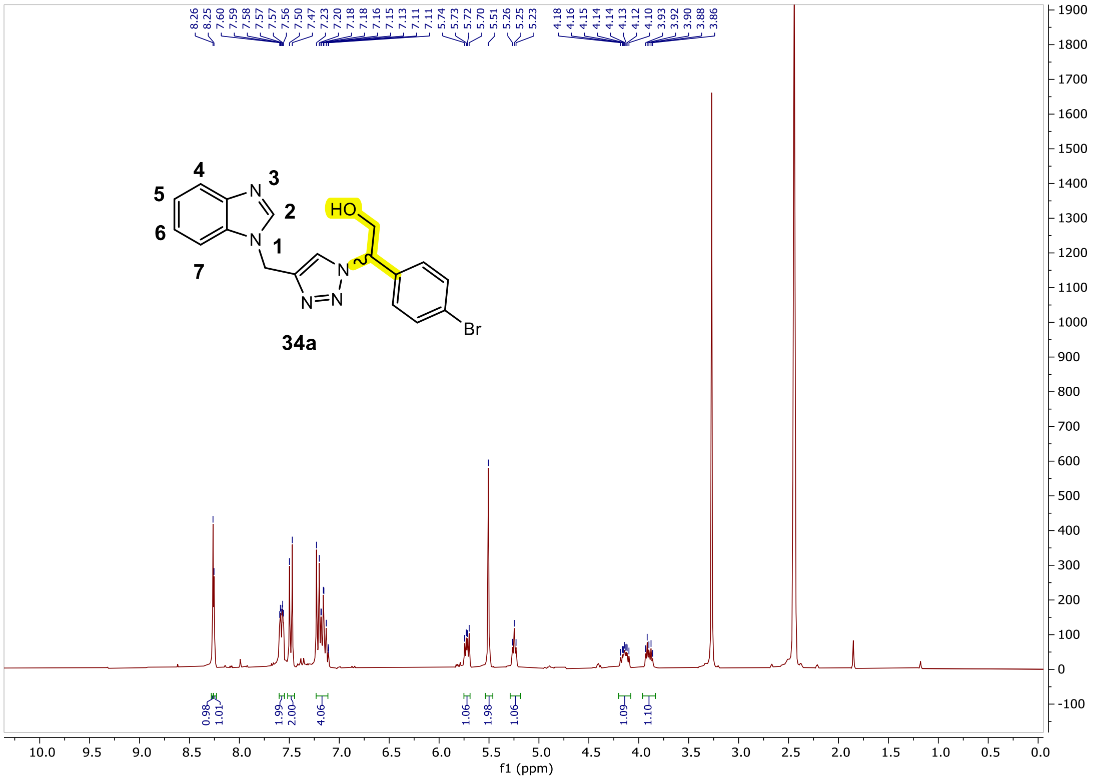

b)

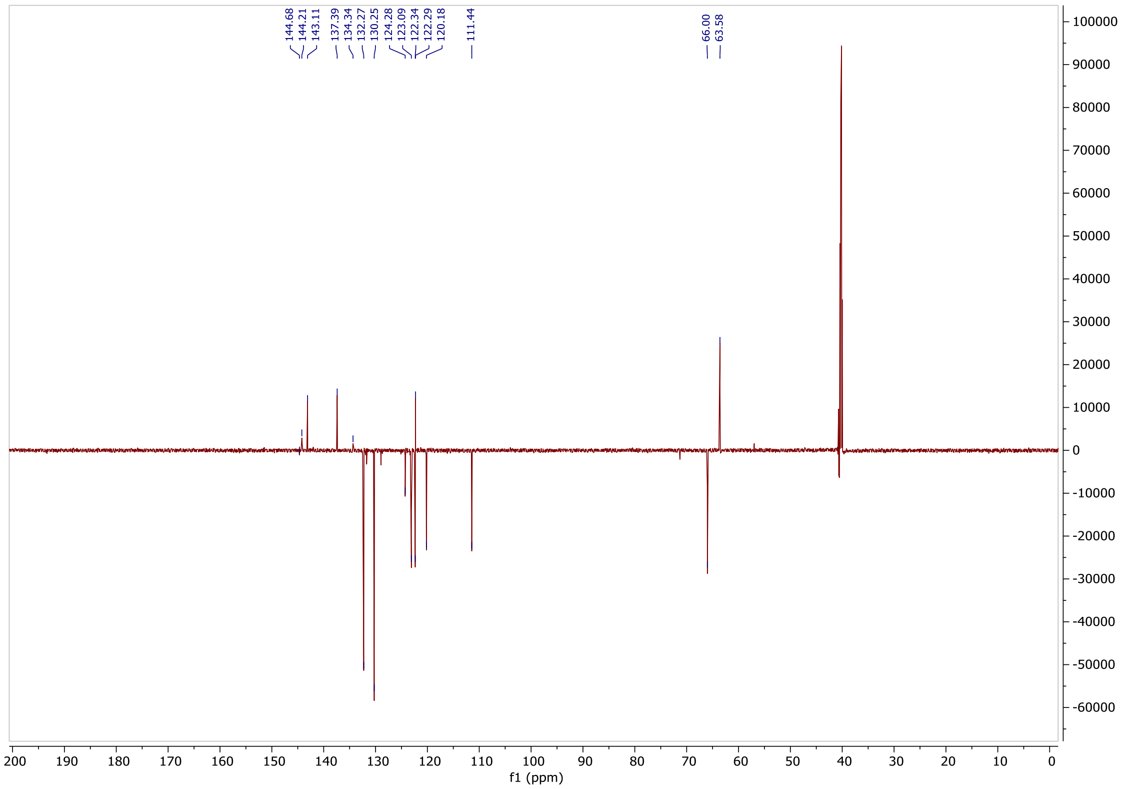

Fig. S10 a) ^{1}H NMR and b) ^{13}C NMR of compd. **35a**

a)

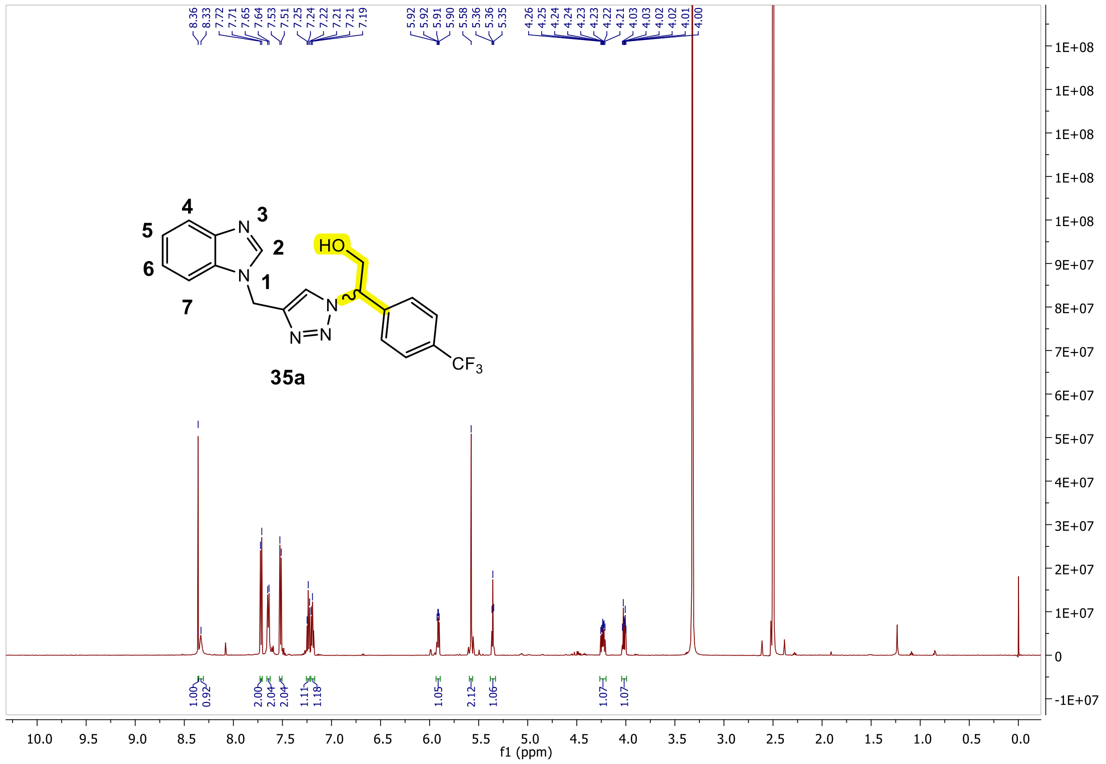

b)

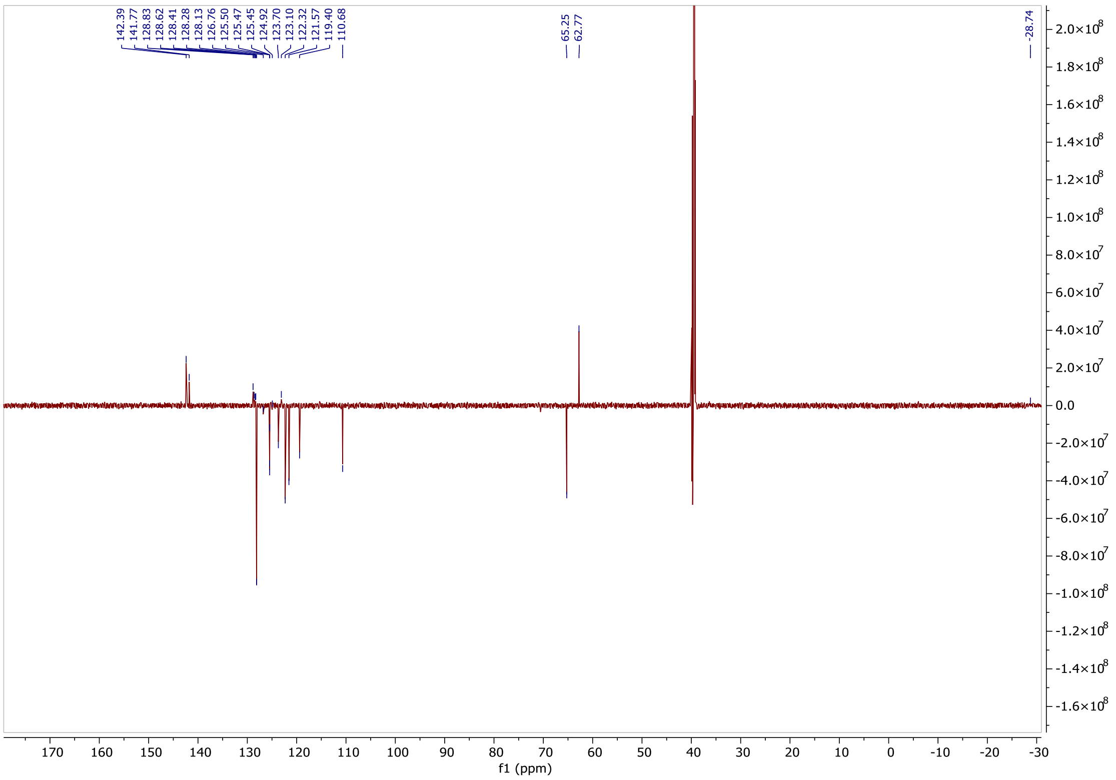

Fig. S11 a) ^{1}H NMR and b) ^{13}C NMR of compd. **36a**

a)

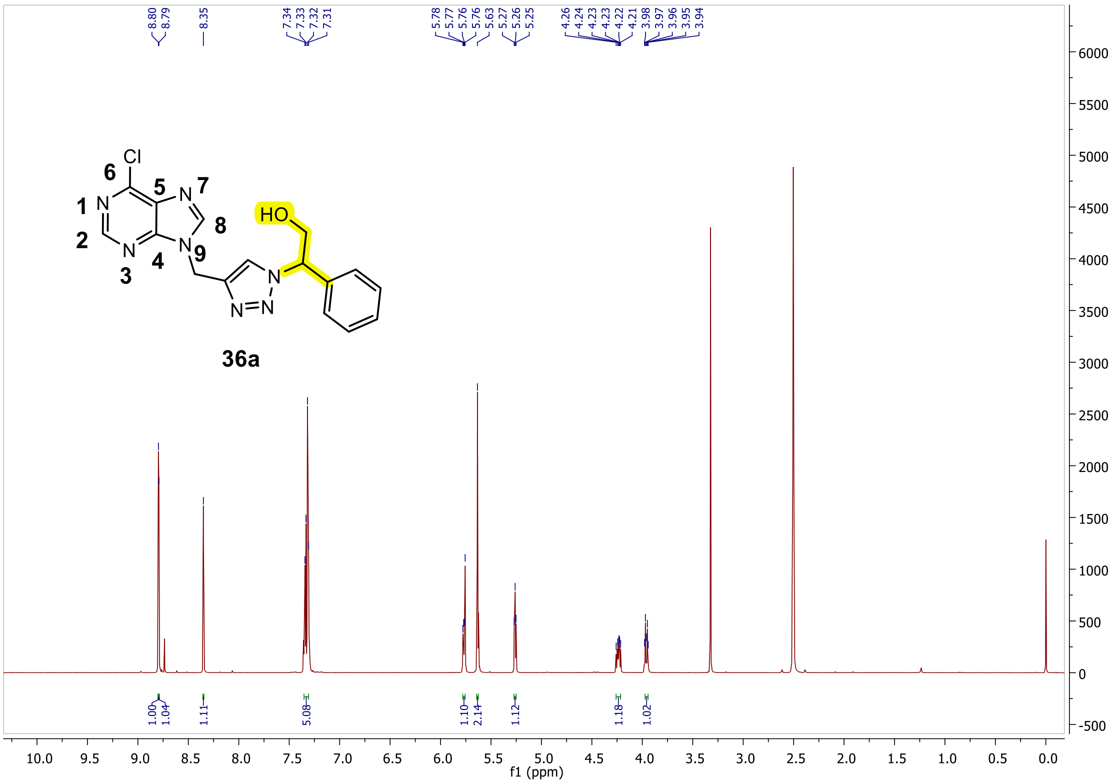

b)

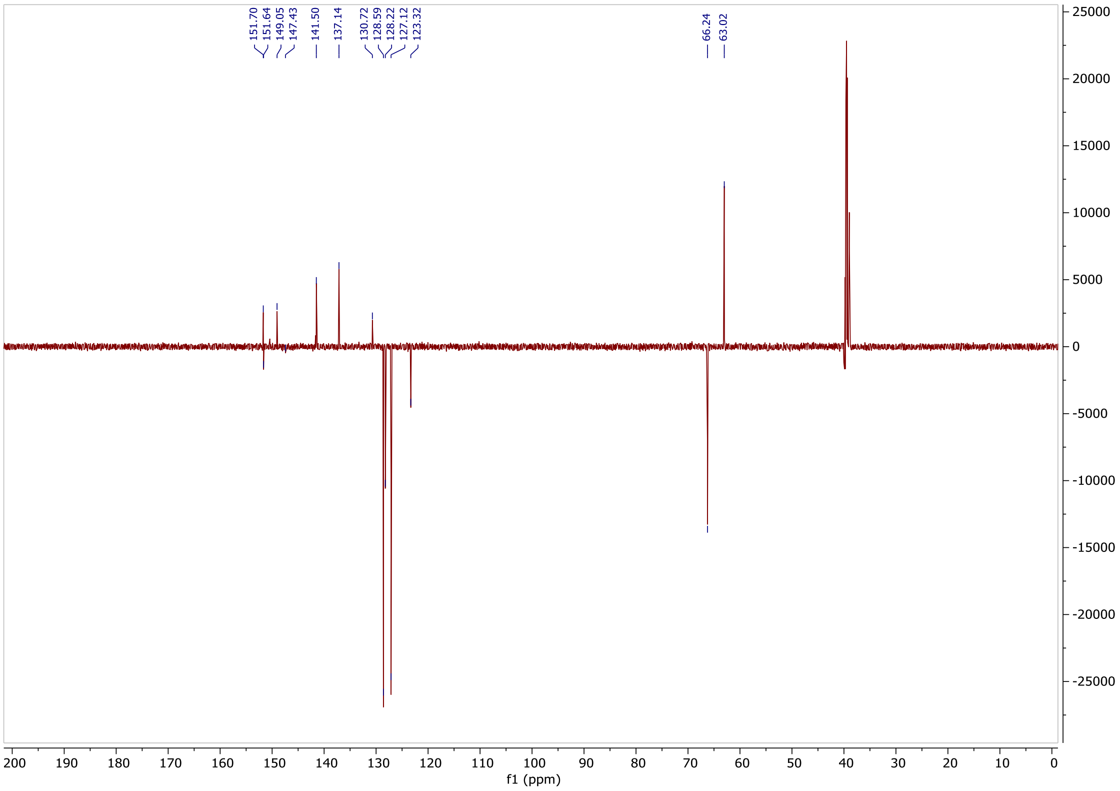

Fig. S12 a) ^{1}H NMR and b) ^{13}C NMR of compd. **37a**

a)

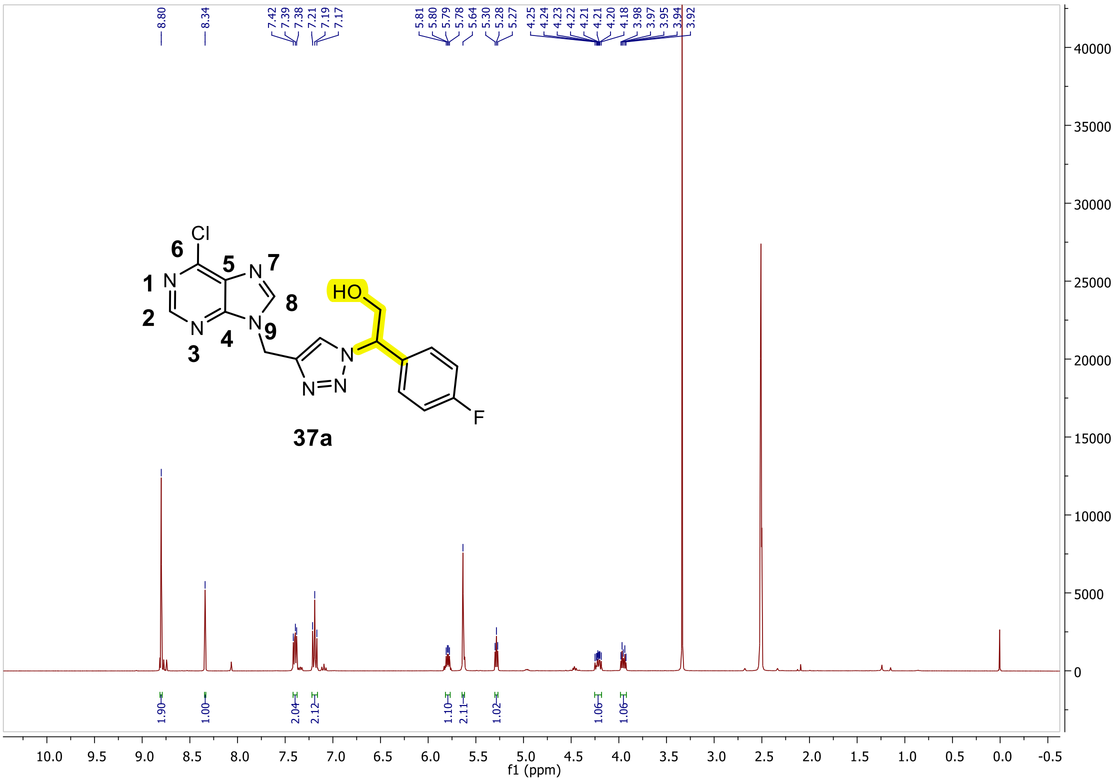

b)

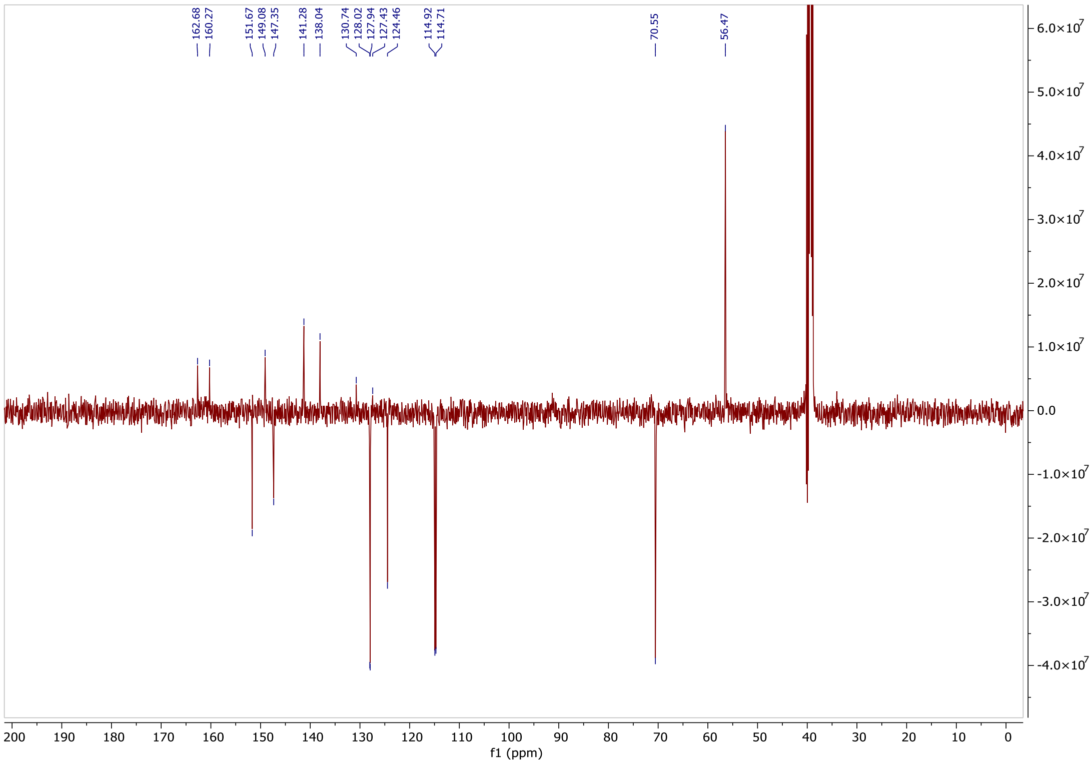

Fig. S13 a) ^{1}H NMR and b) ^{13}C NMR of compd. **38a**

a)

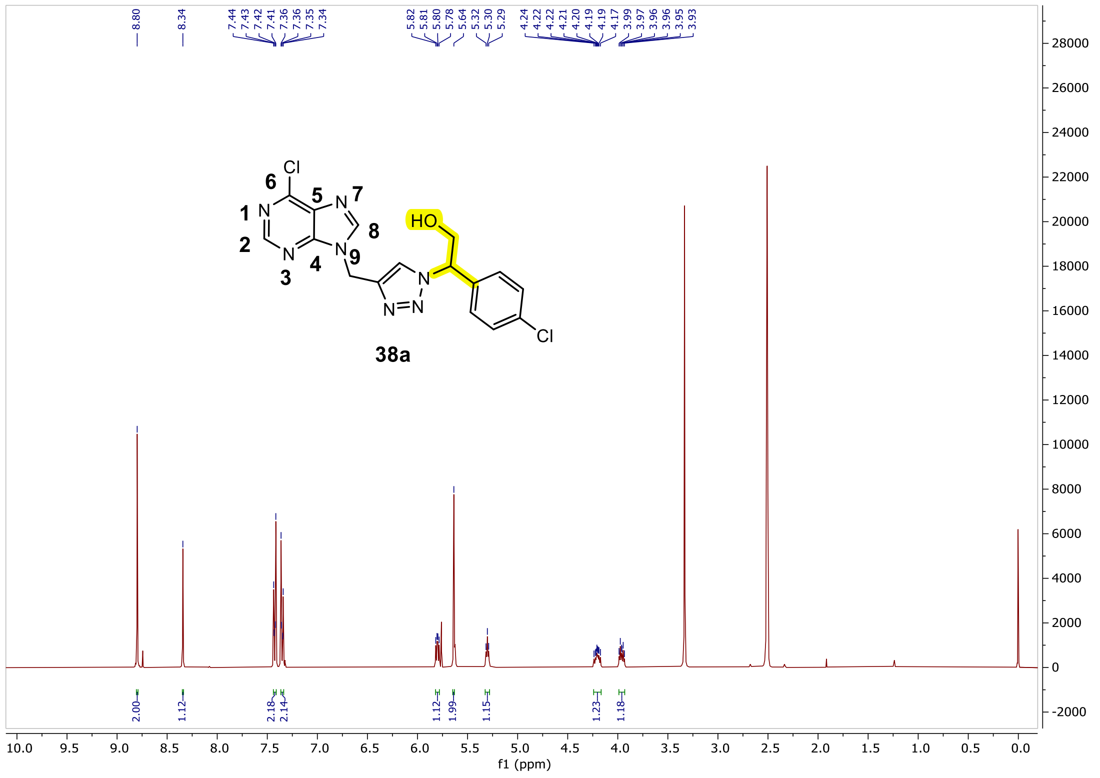

b)

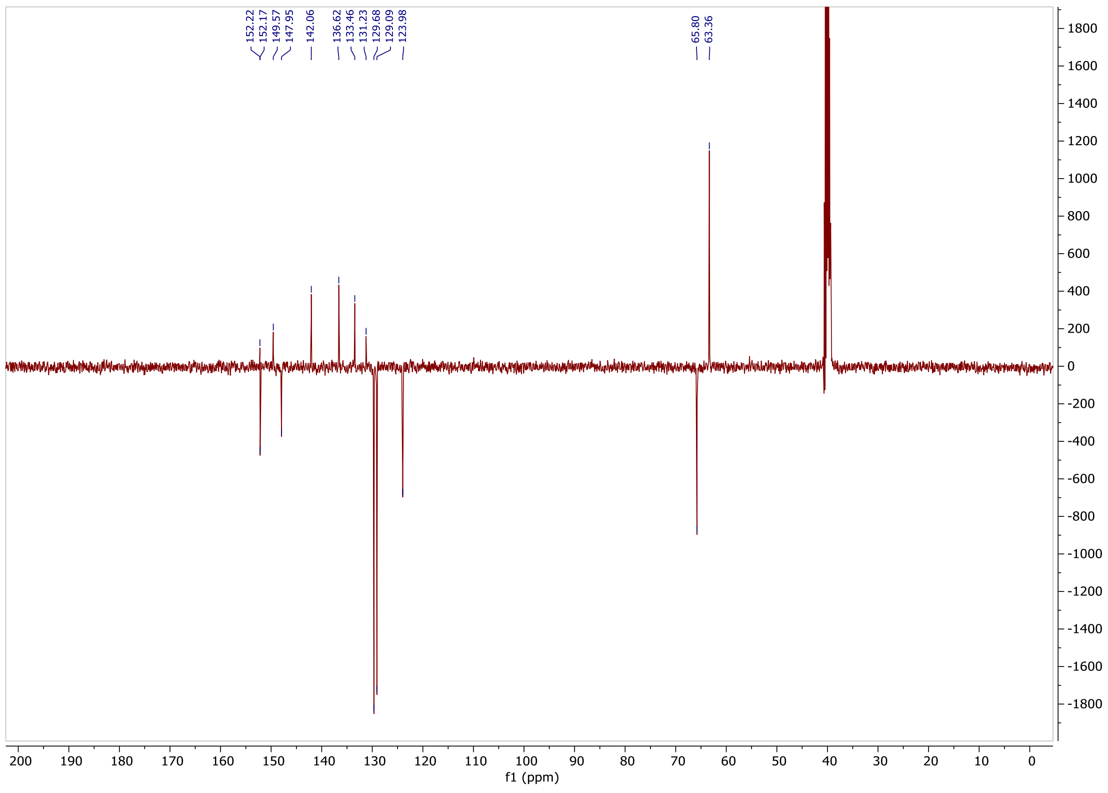

Fig. S14 a) ^1H NMR and b) ^{13}C NMR of compd. **39a**

a)

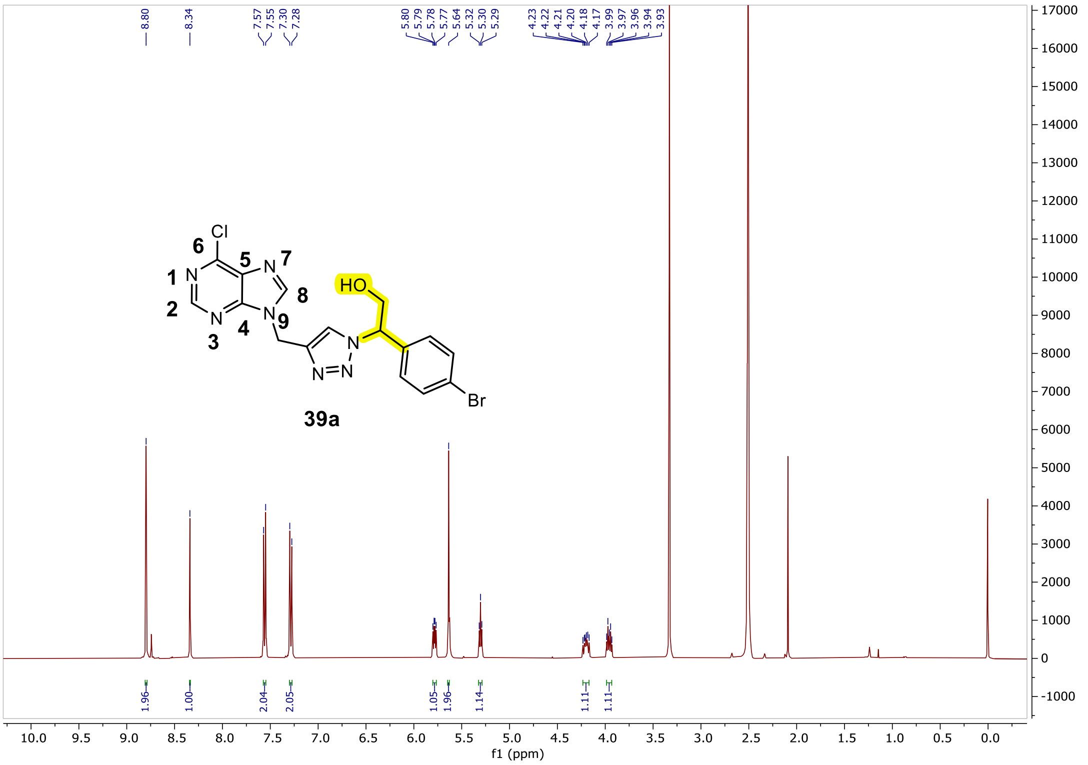

b)

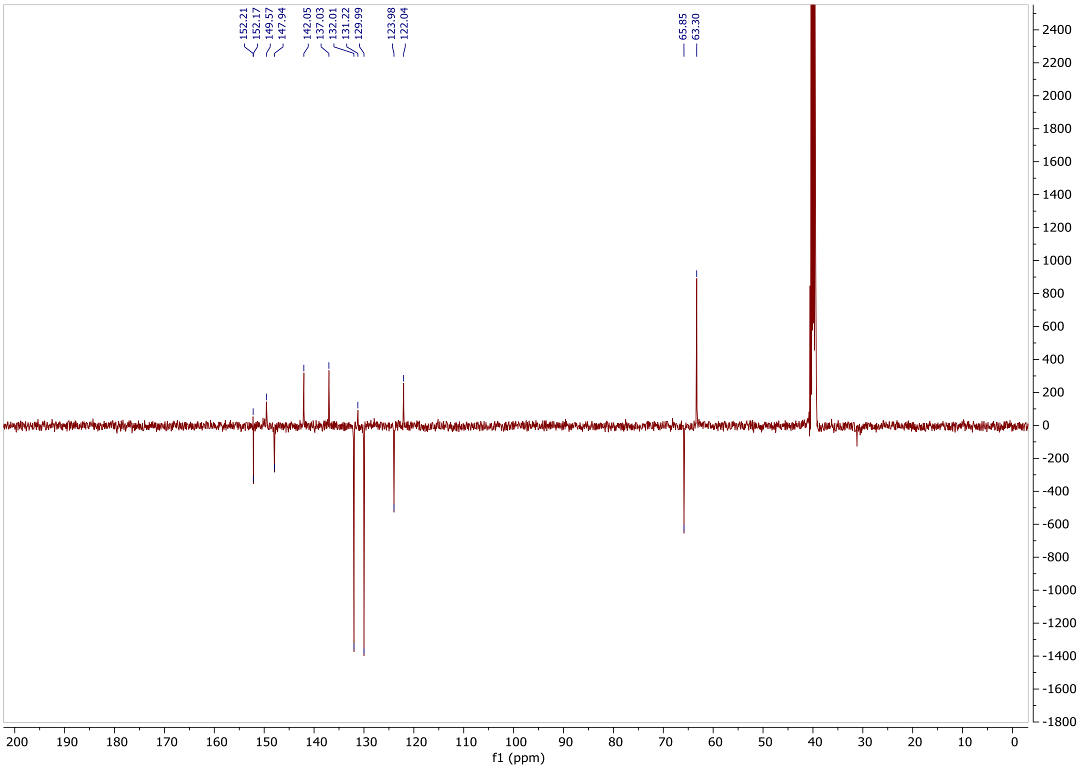

Fig. S15 a) ^{1}H NMR and b) ^{13}C NMR of compd. **40a**

a)

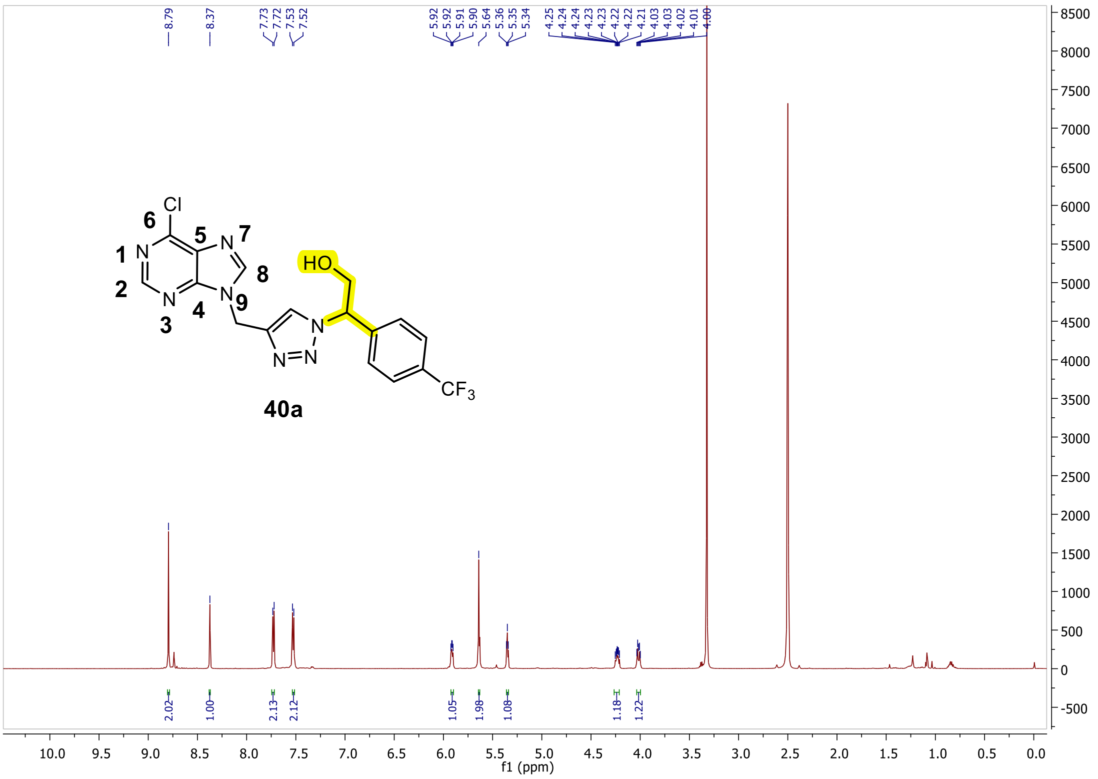

b)

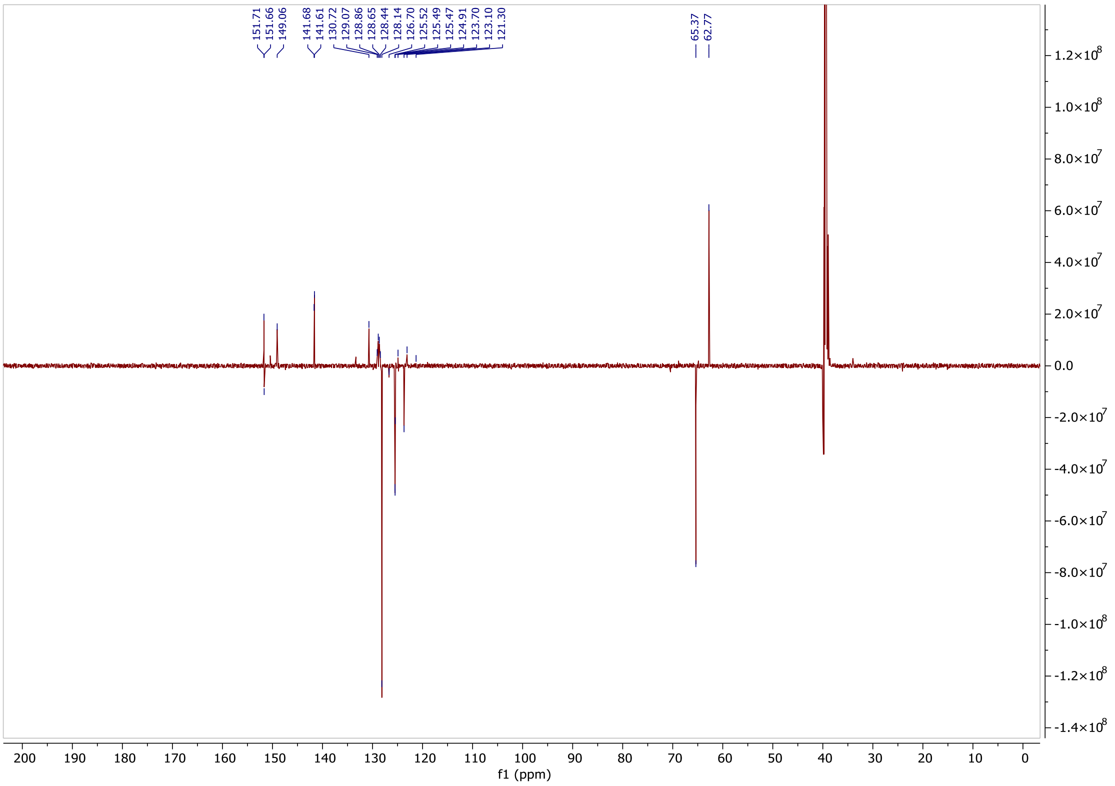

Fig. S16 a) ^{1}H NMR and b) ^{13}C NMR of compd. **41a**

a)

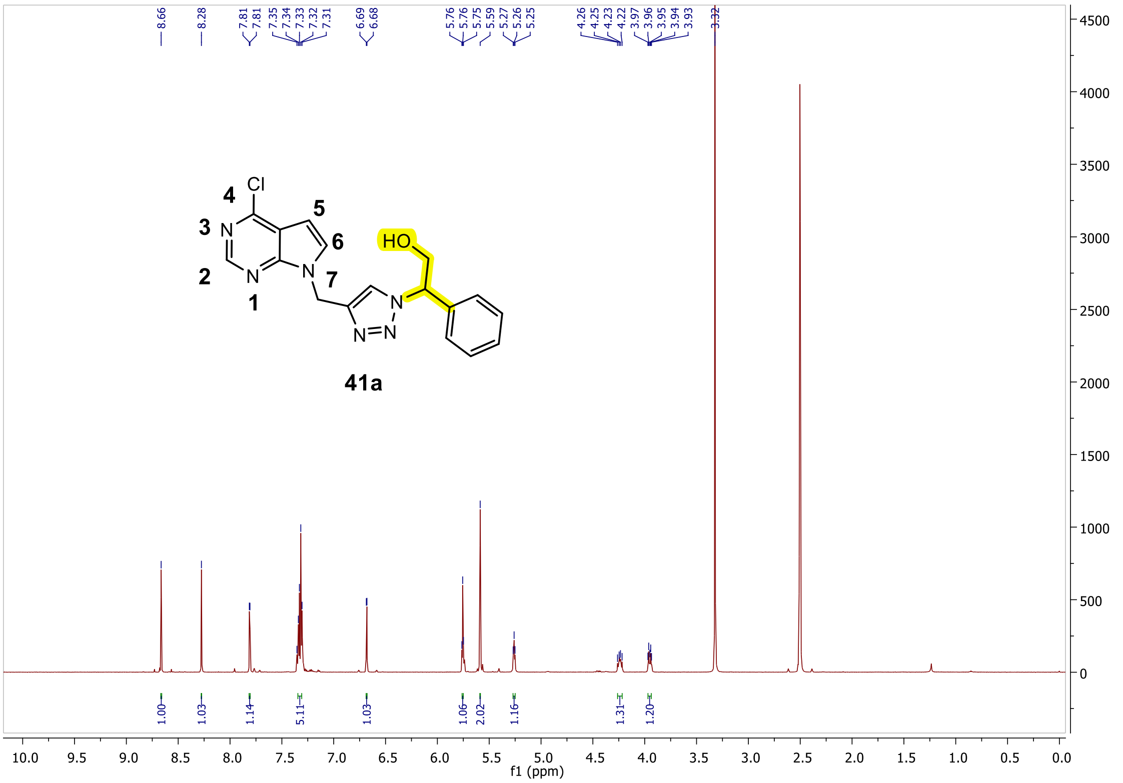

b)

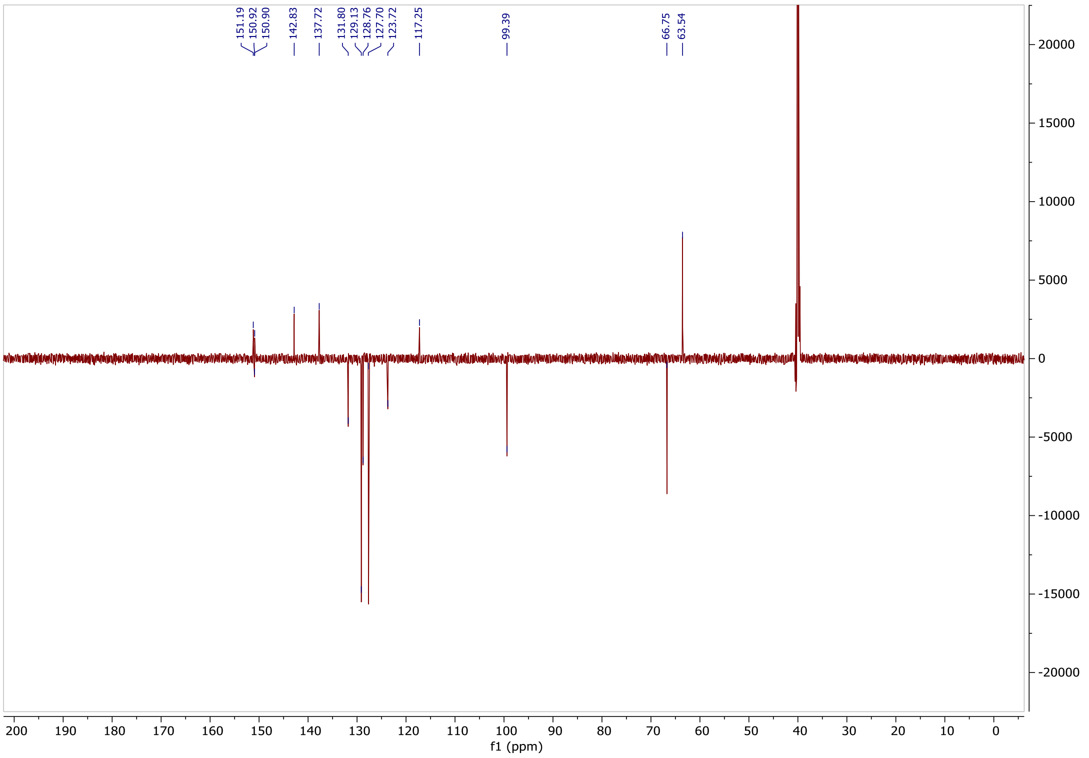

Fig. S17 a) ^{1}H NMR and b) ^{13}C NMR of compd. **42a**

a)

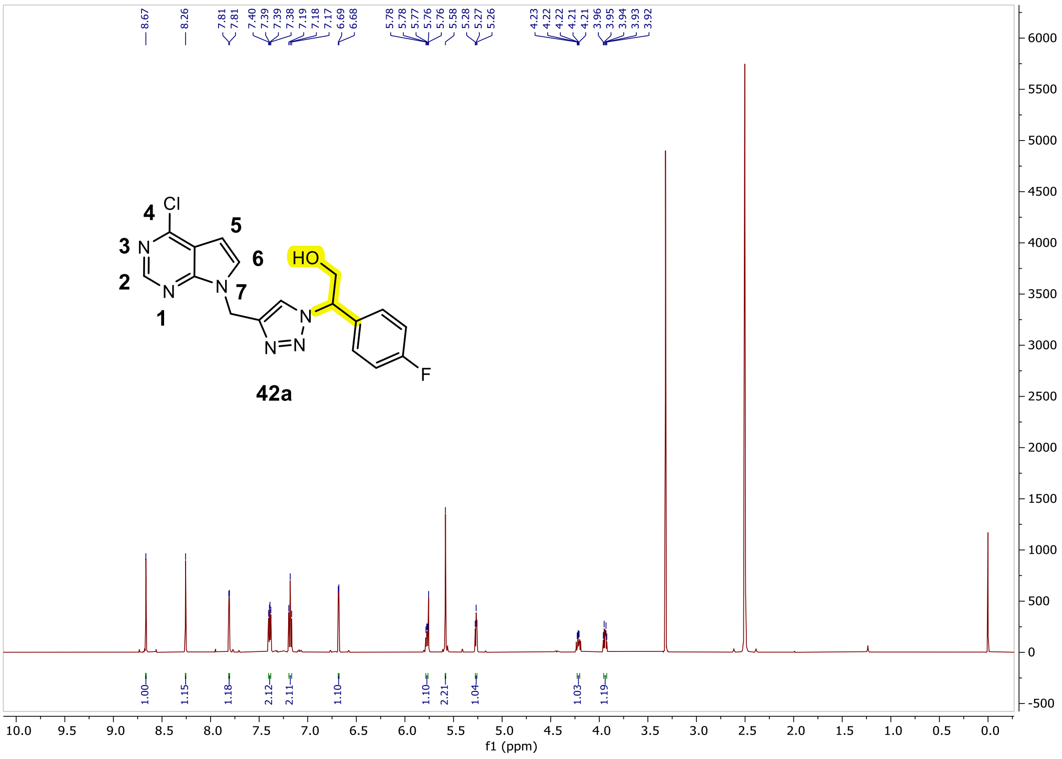

b)

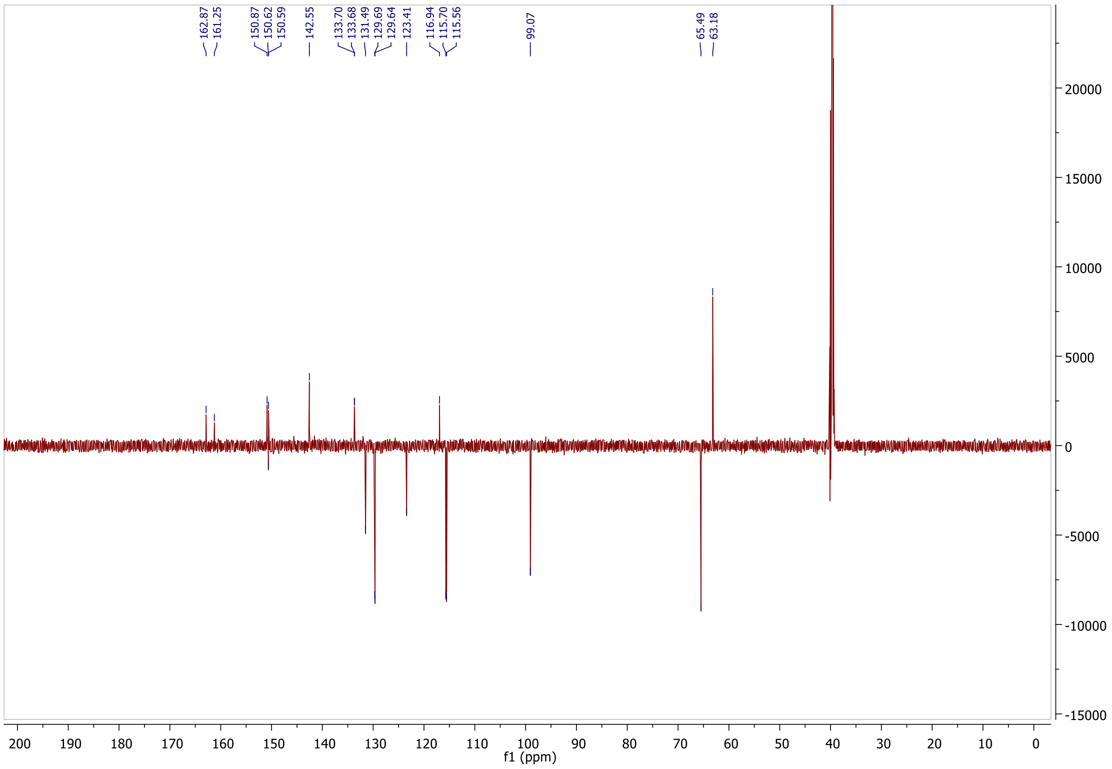

Fig. S18 a) ^{1}H NMR and b) ^{13}C NMR of compd. **43a**

a)

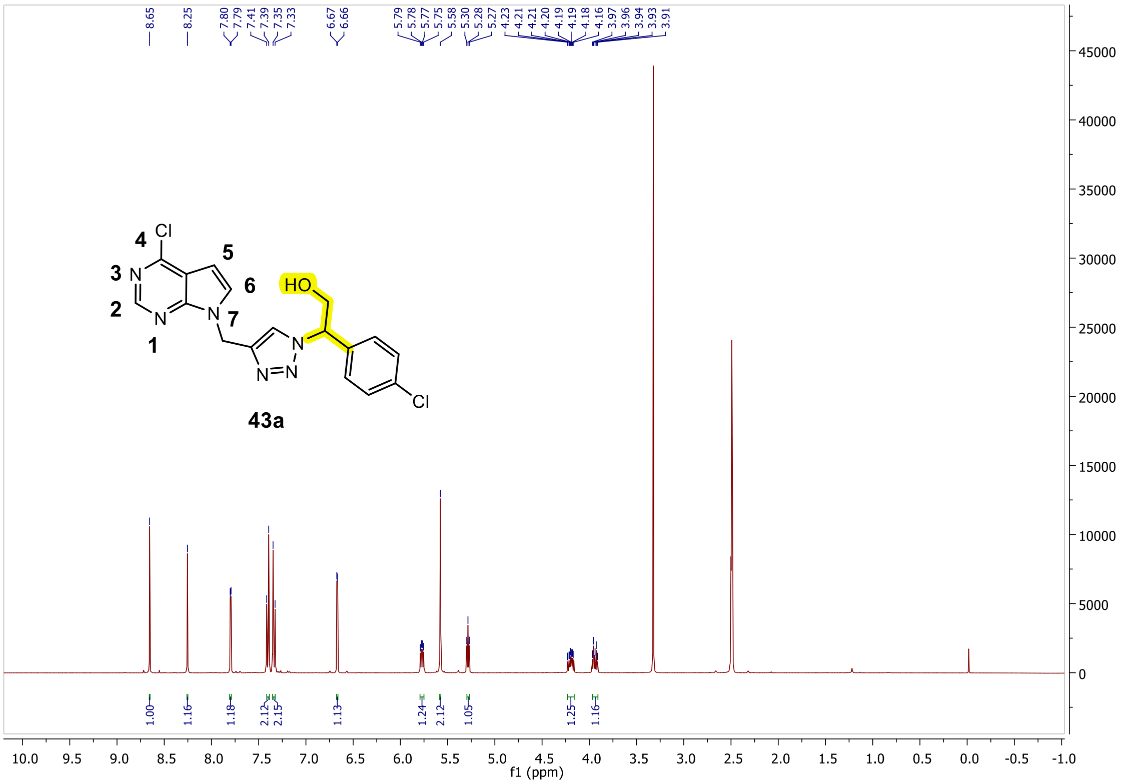

b)

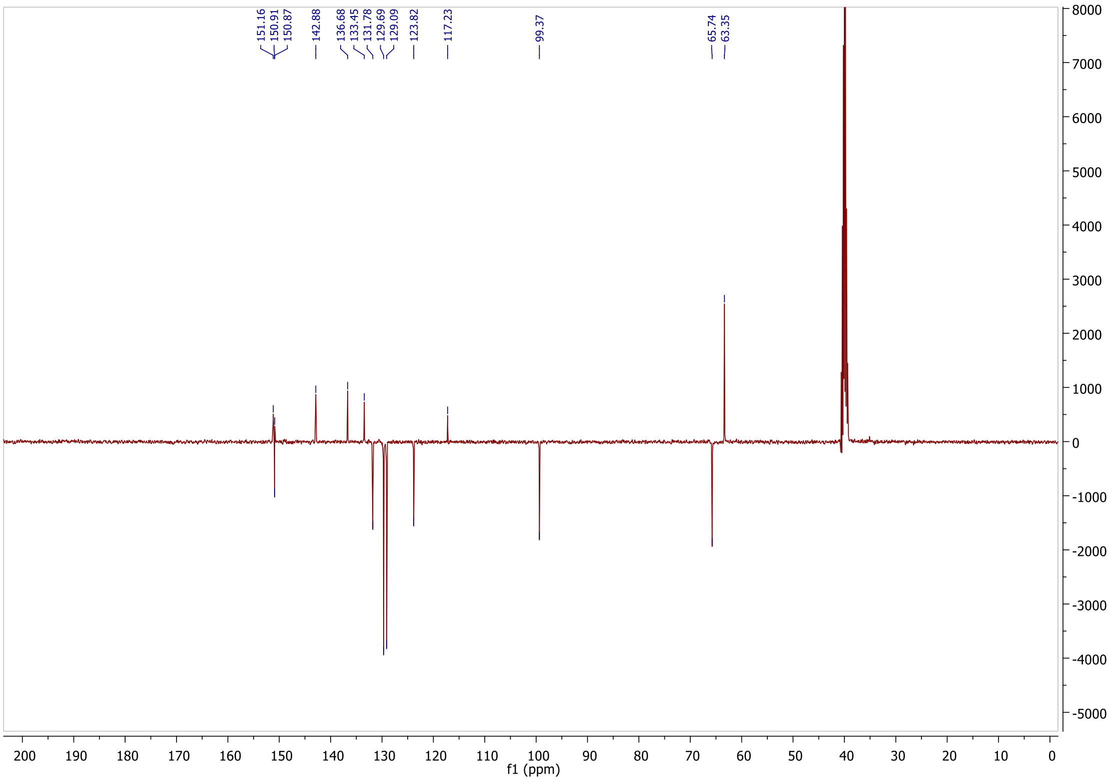

Fig. S19 a) ^{1}H NMR and b) ^{13}C NMR of compd. **44a**

a)

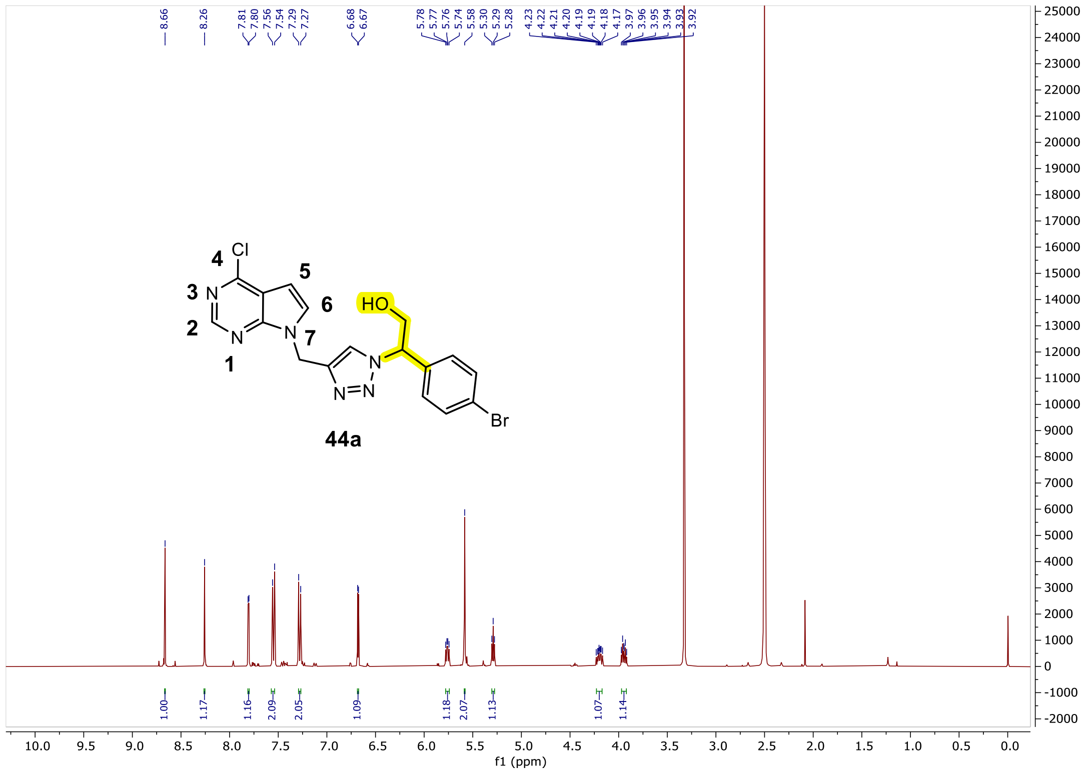

b)

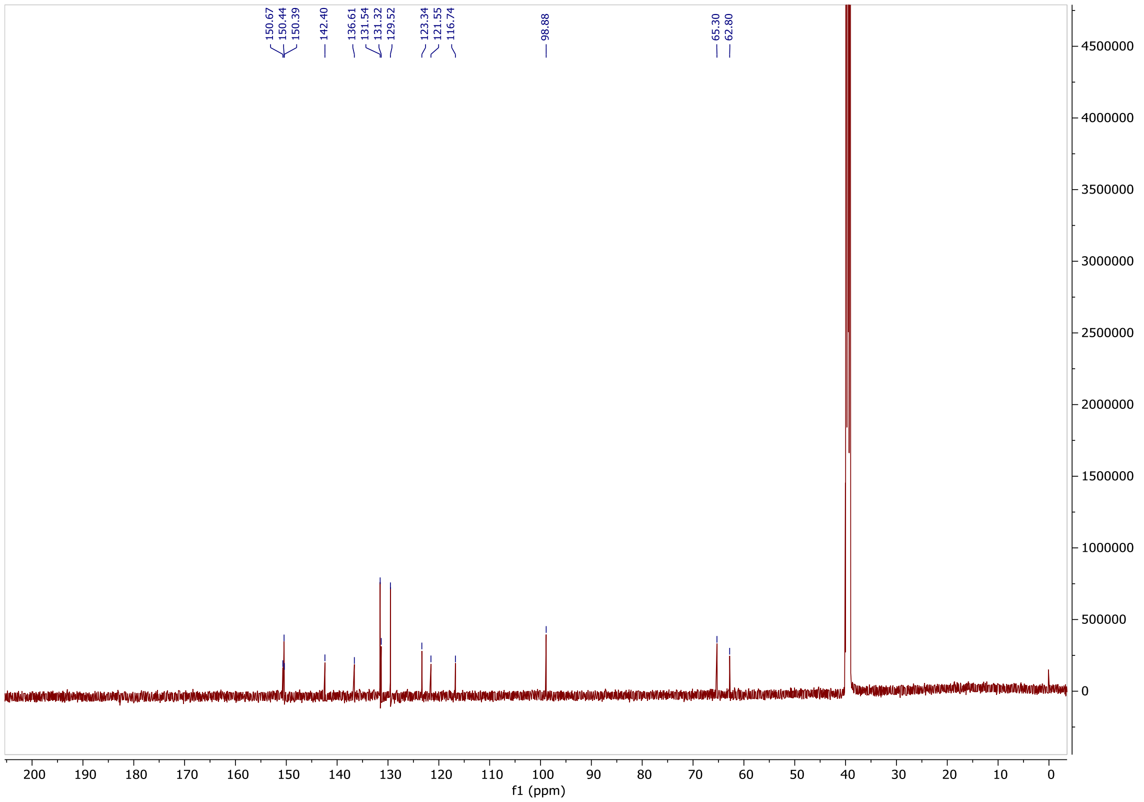

Fig. S20 a) ^{1}H NMR and b) ^{13}C NMR of compd. **45a**

a)

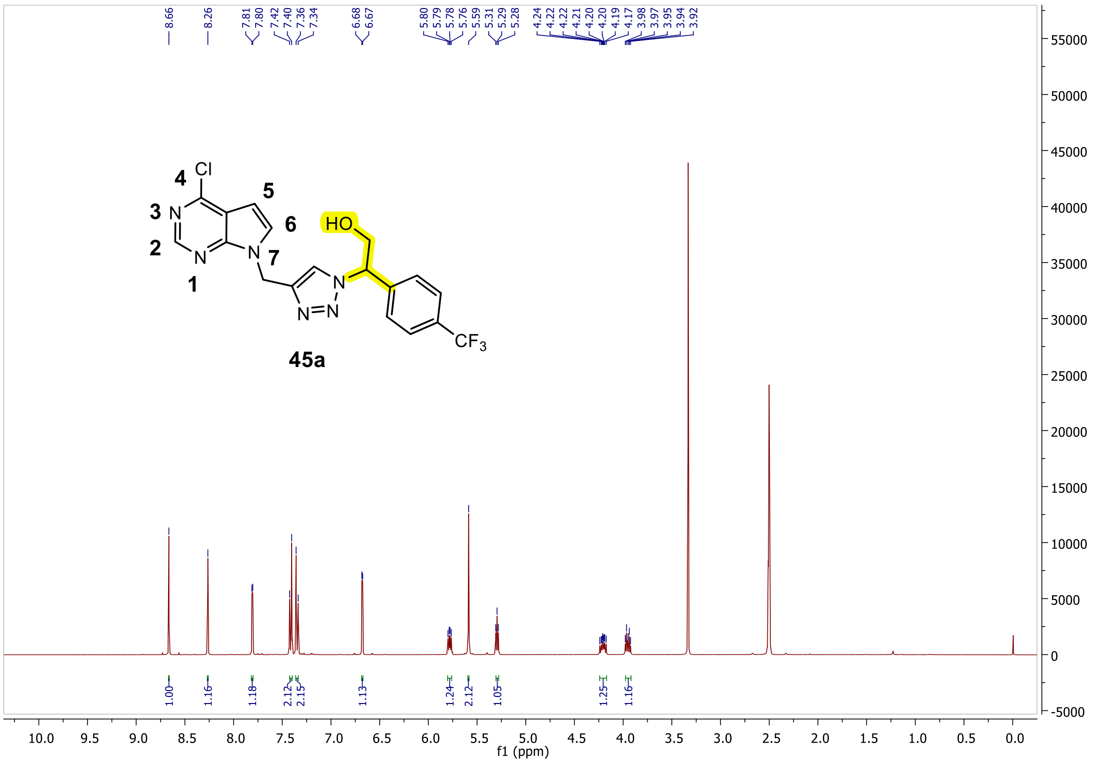

b)

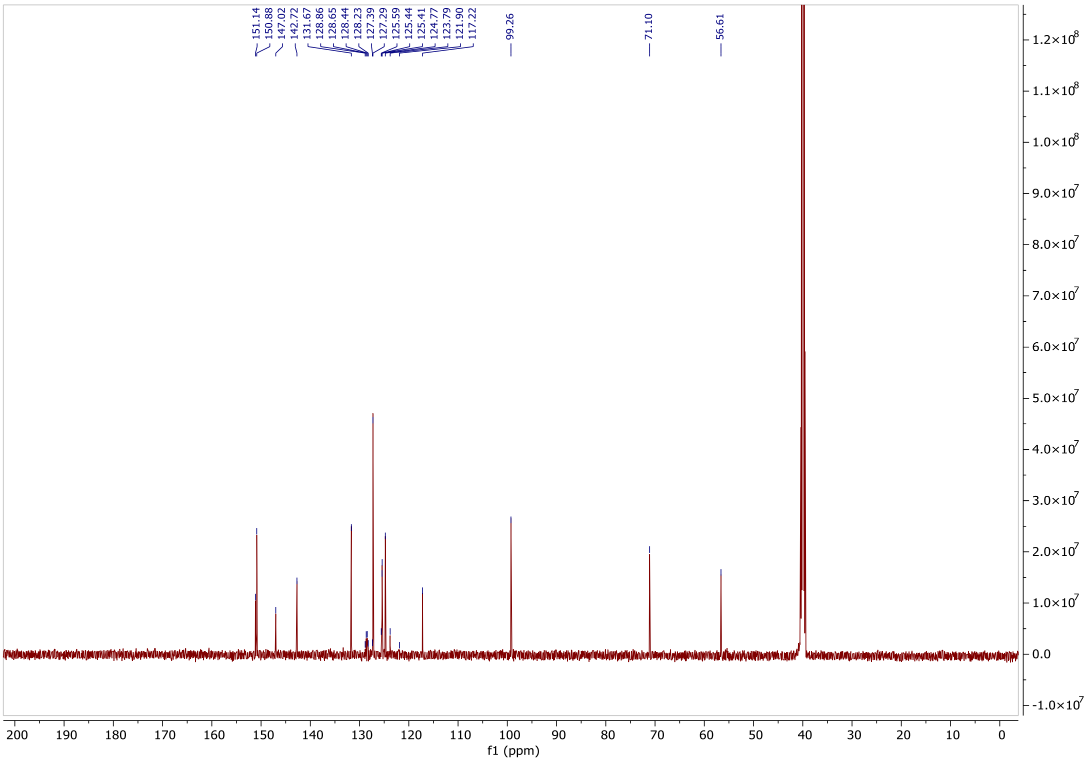

Fig. S21 a) ^{1}H NMR and b) ^{13}C NMR of compd. **46a**

a)

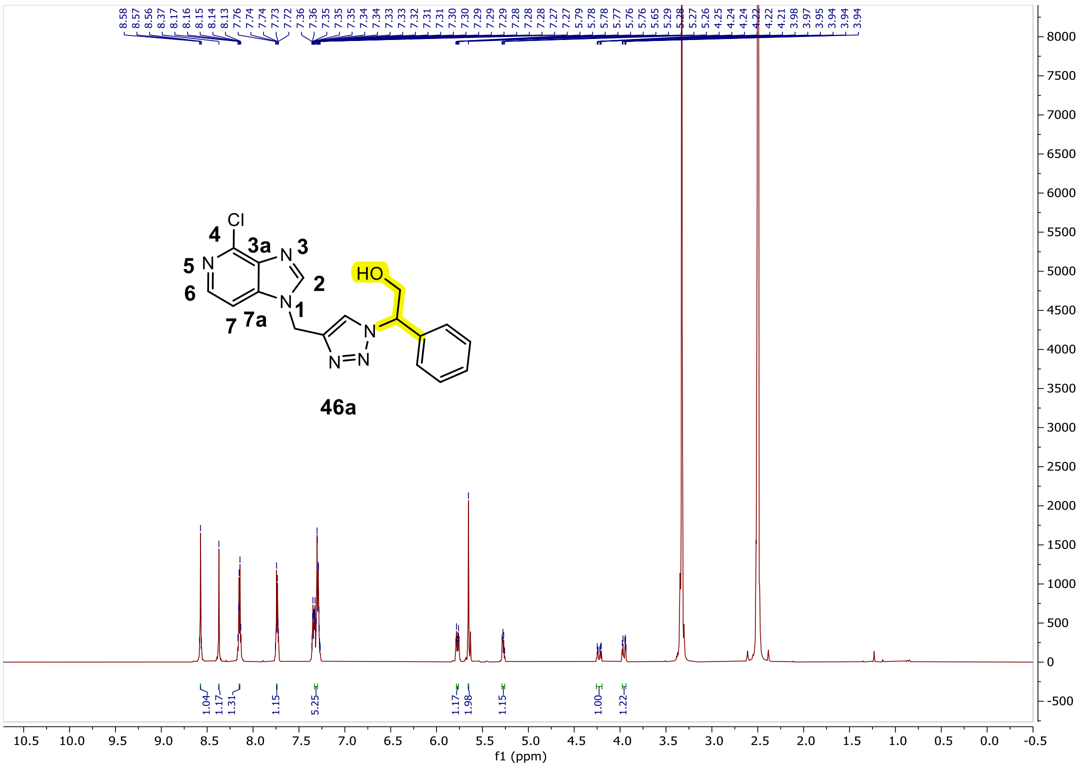

b)

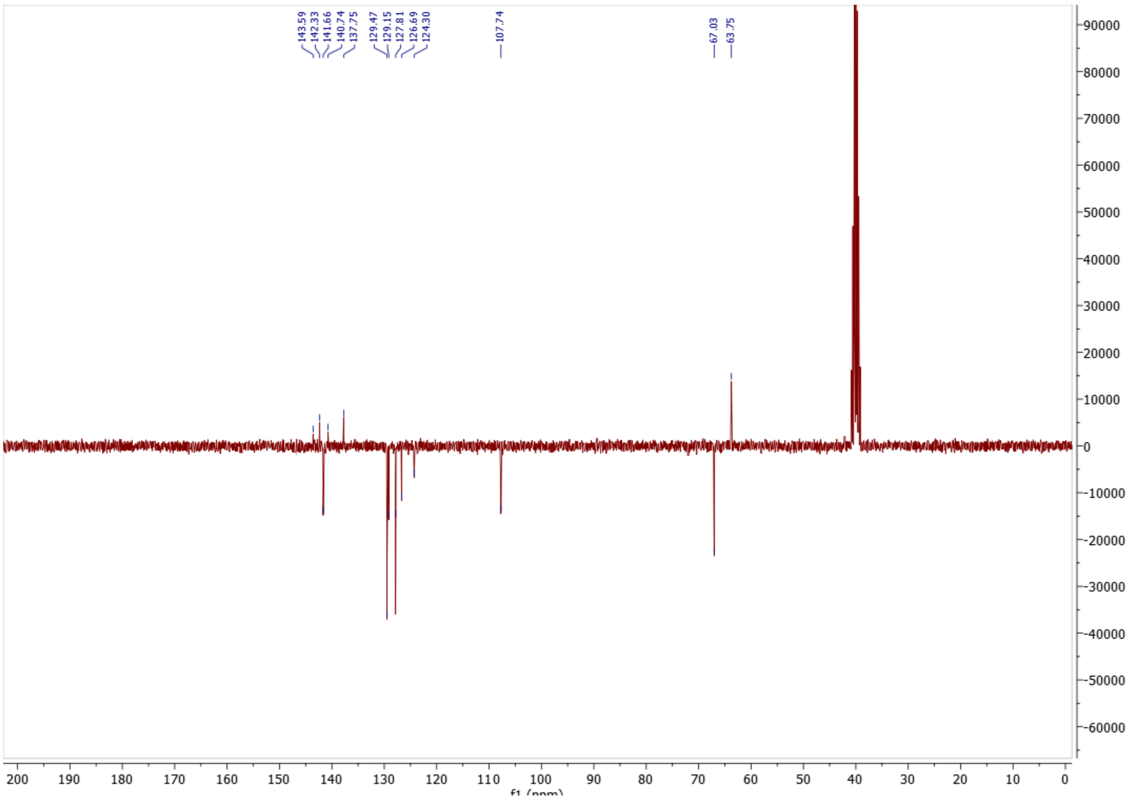

Fig. S22 a) ^{1}H NMR and b) ^{13}C NMR of compd. **47a**

a)

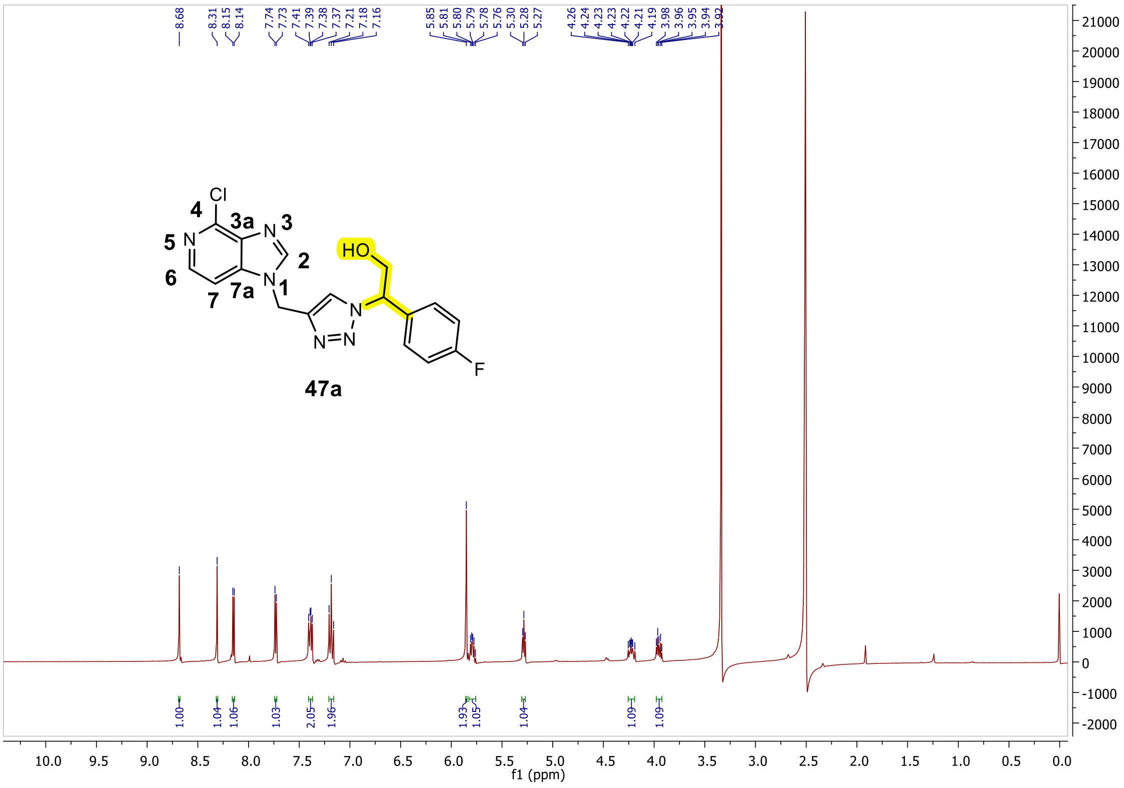

b)

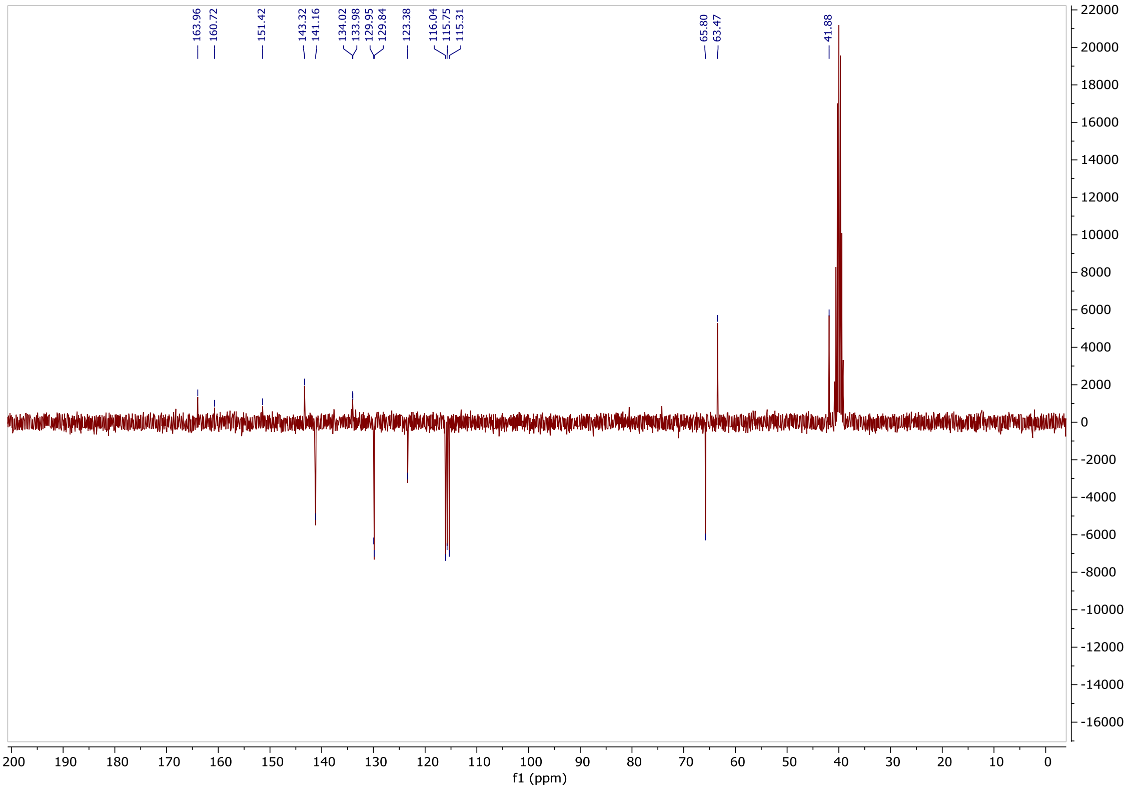

Fig. S23 a) ^{1}H NMR and b) ^{13}C NMR of compd. **48a**

a)

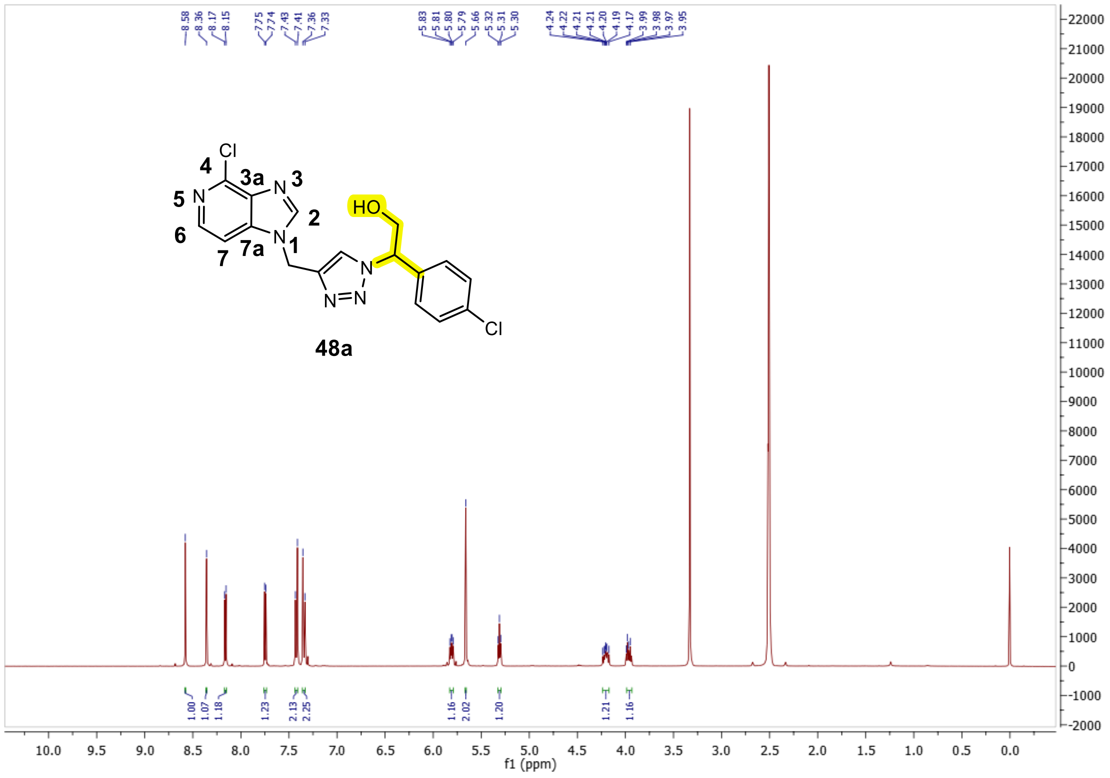

b)

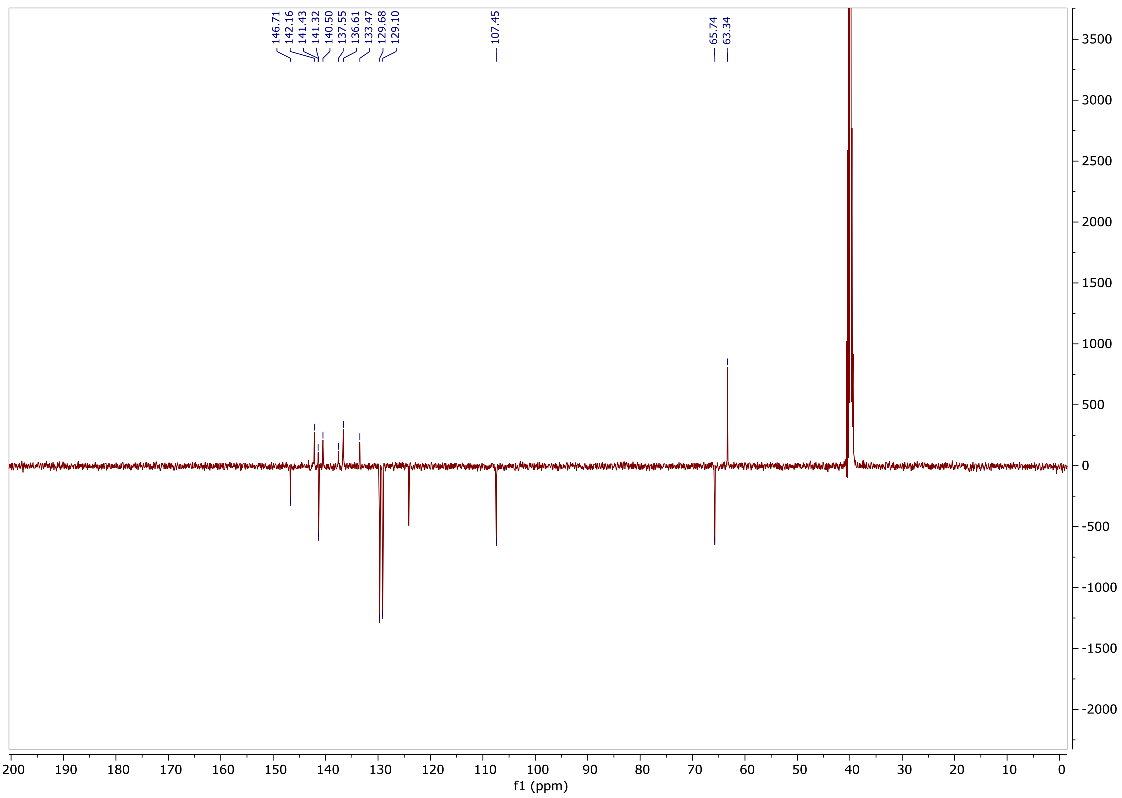

Fig. S24 a) ^{1}H NMR and b) ^{13}C NMR of compd. **49a**

a)

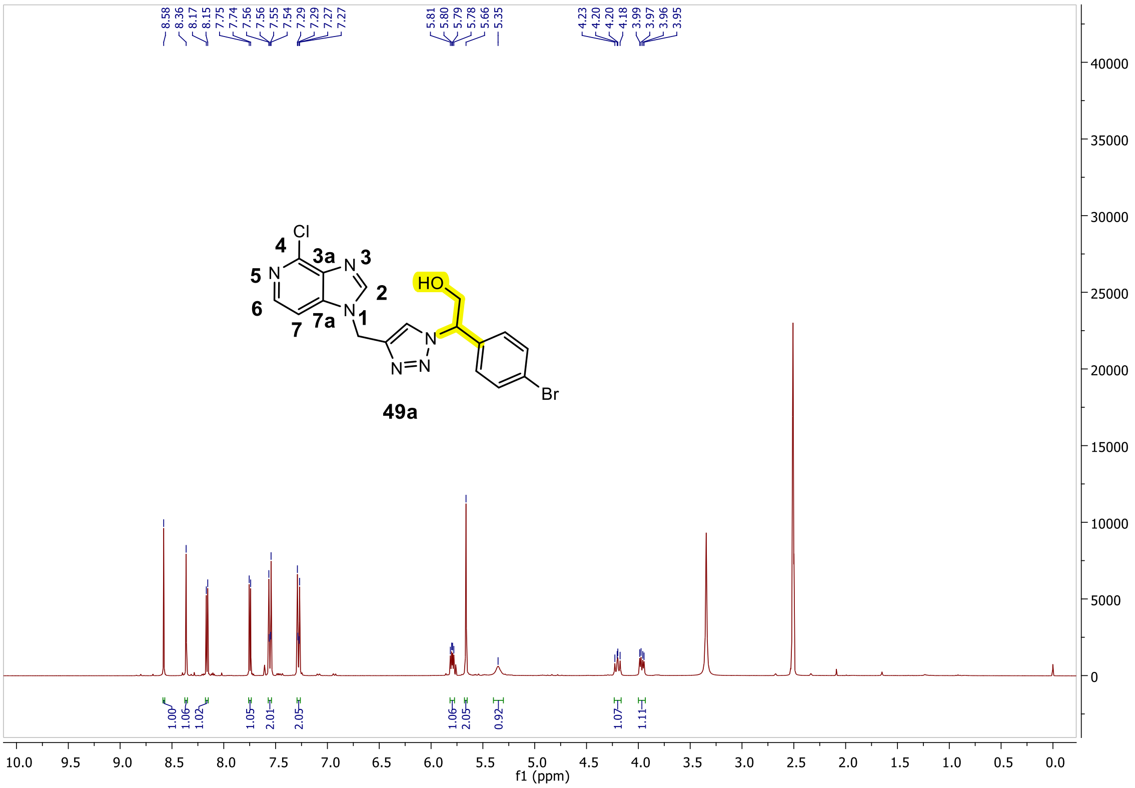

b)

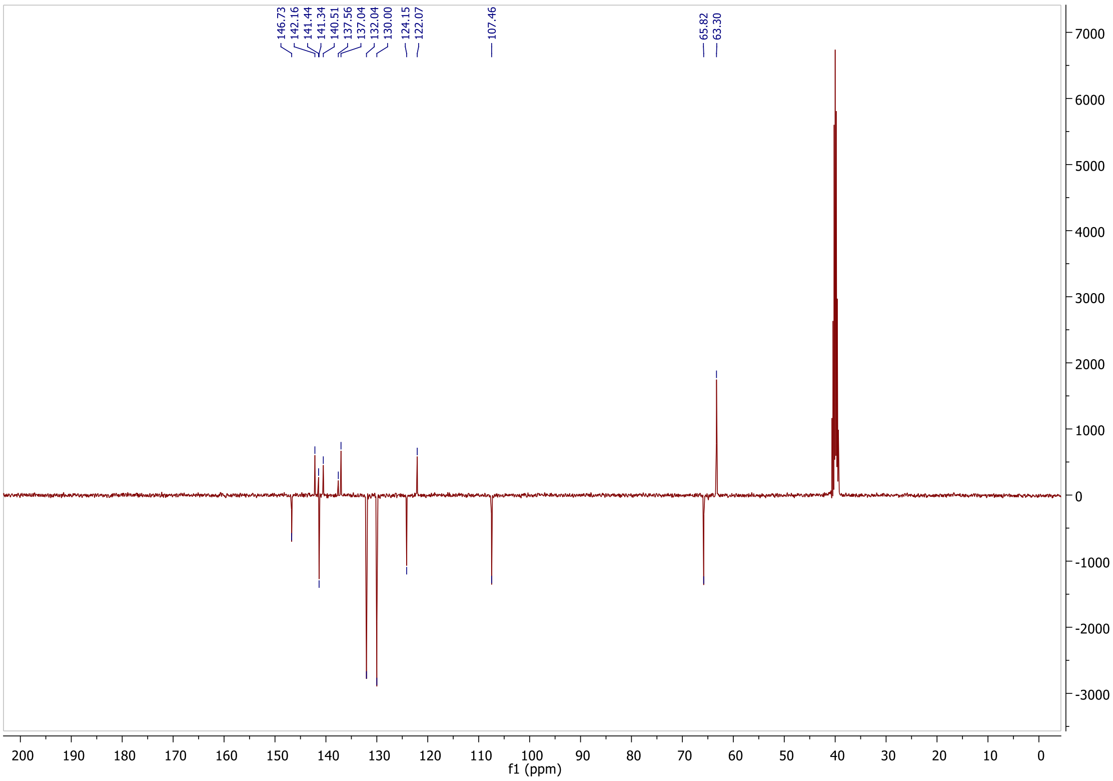

Fig. S25 a) ^{1}H NMR and b) ^{13}C NMR of compd. **26b**

a)

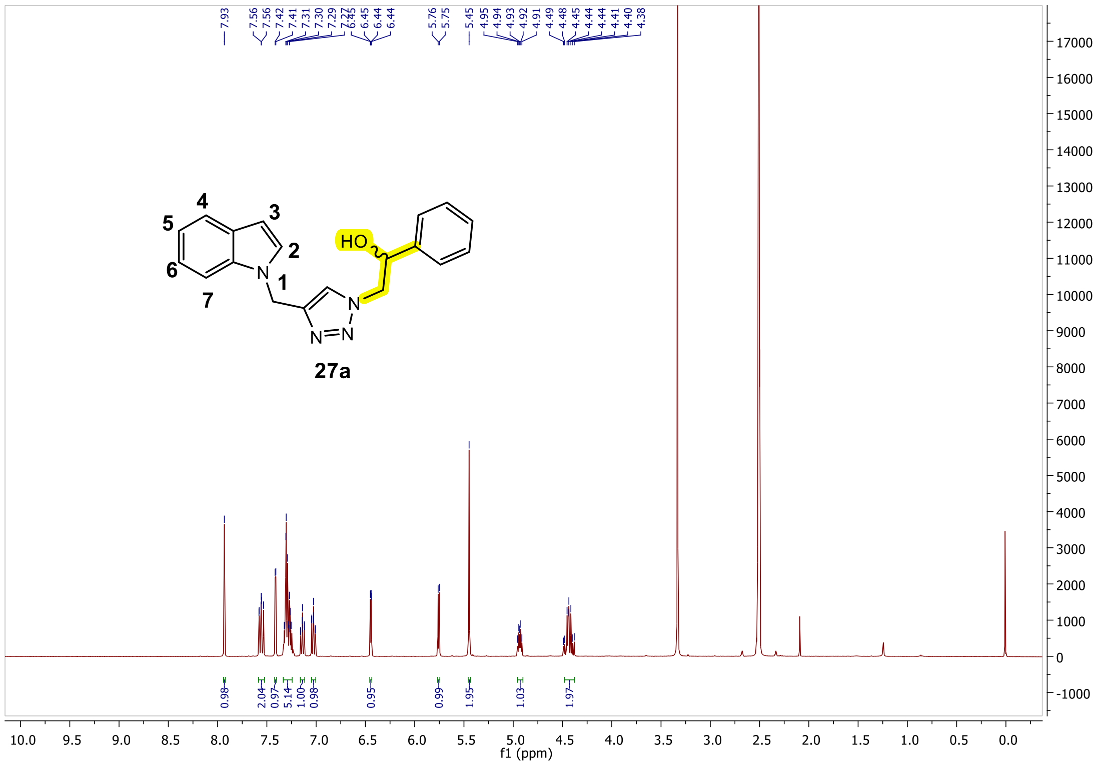

b)

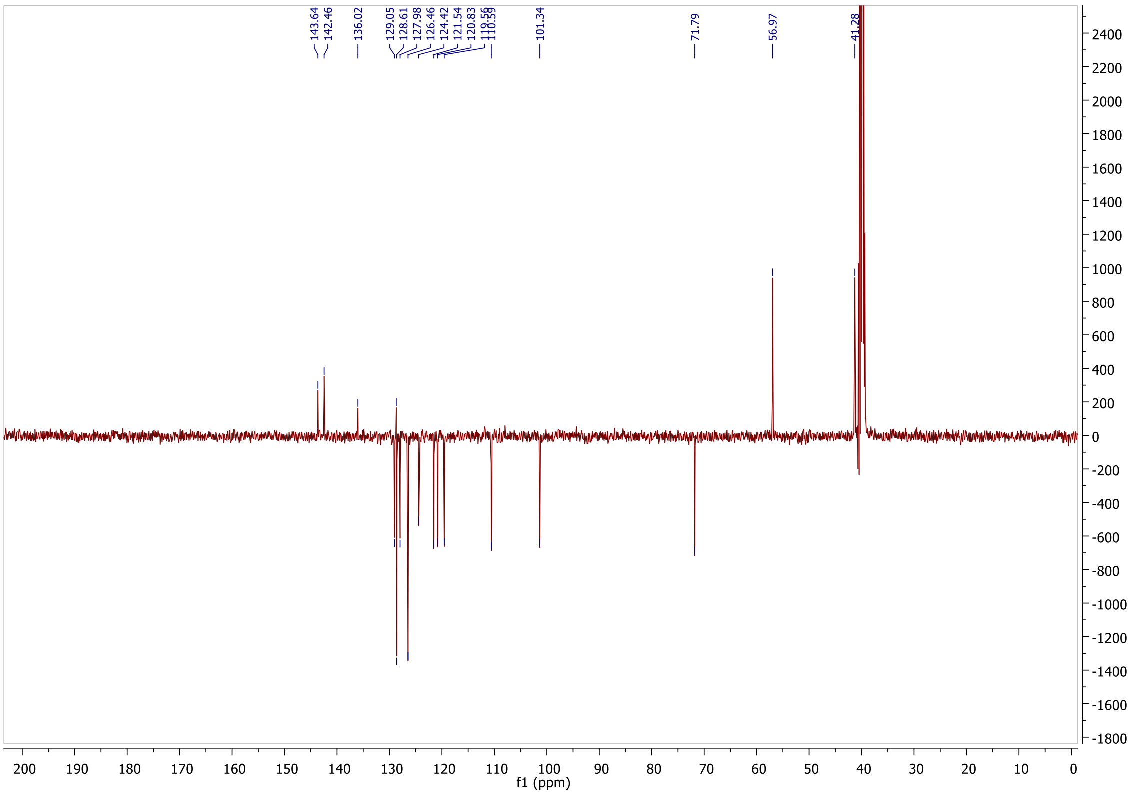

Fig. S26 a) ^{1}H NMR and b) ^{13}C NMR of compd. **27b**

a)

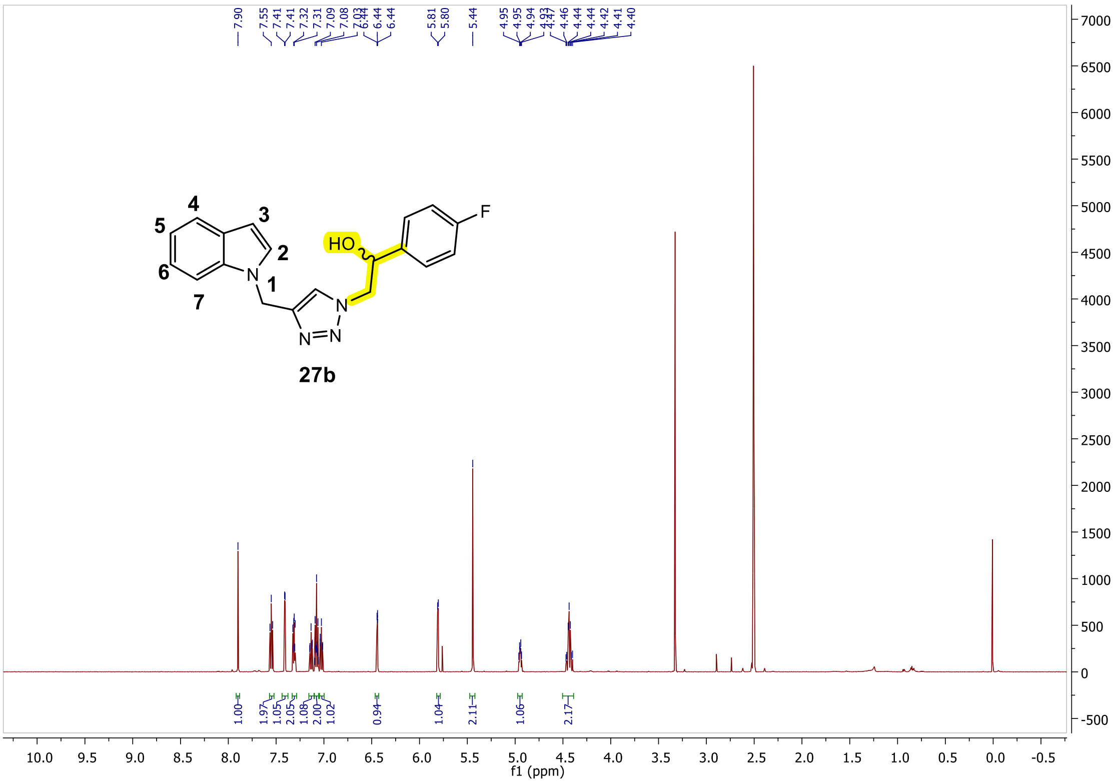

b)

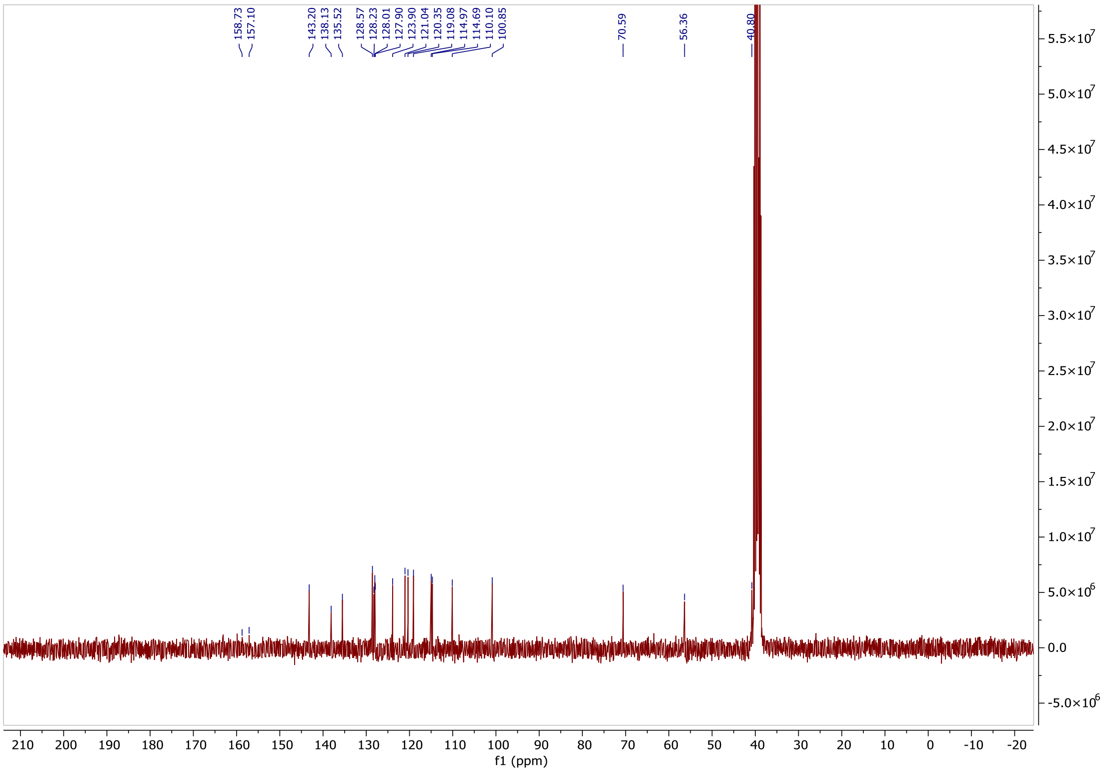

Fig. S27 a) ^{1}H NMR and b) ^{13}C NMR of compd. **28b**

a)

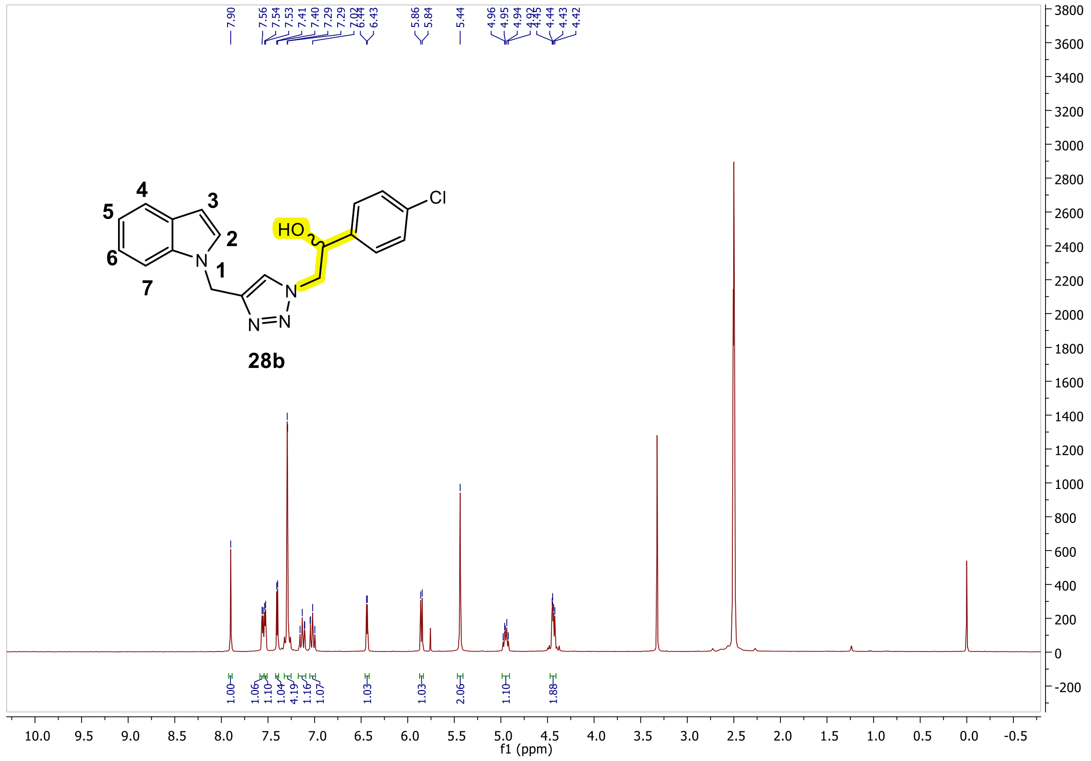

b)

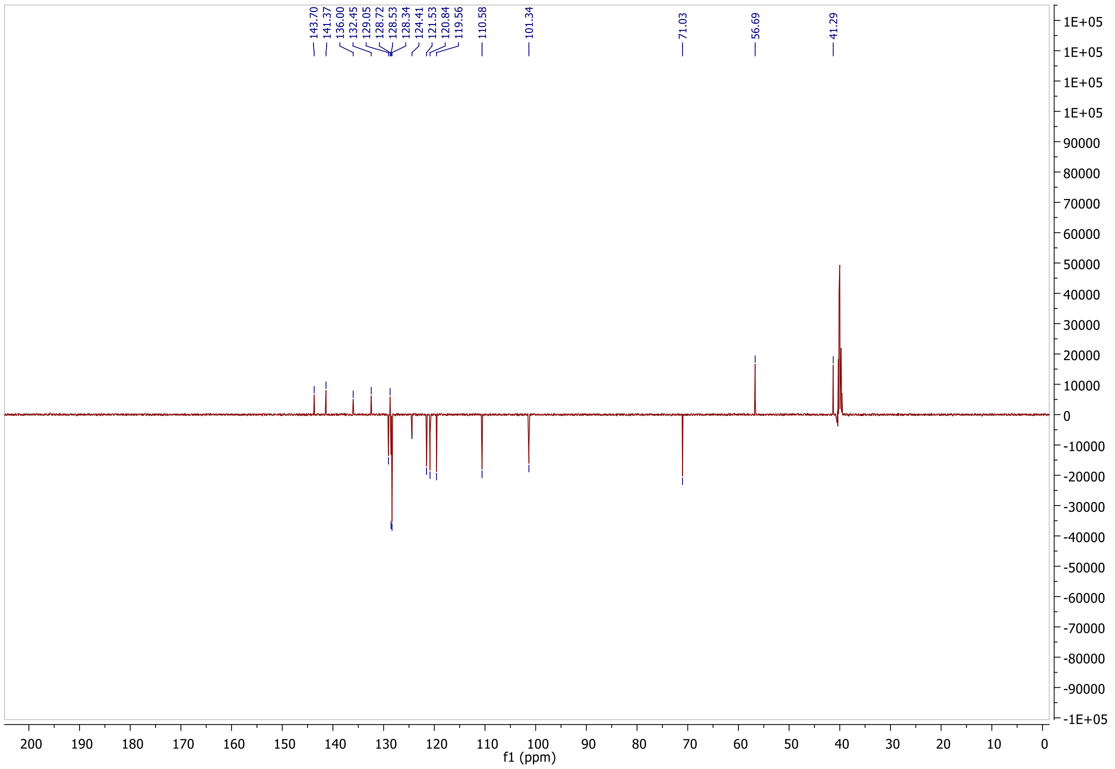

Fig. S28 a) ^{1}H NMR and b) ^{13}C NMR of compd. **29b**

a)

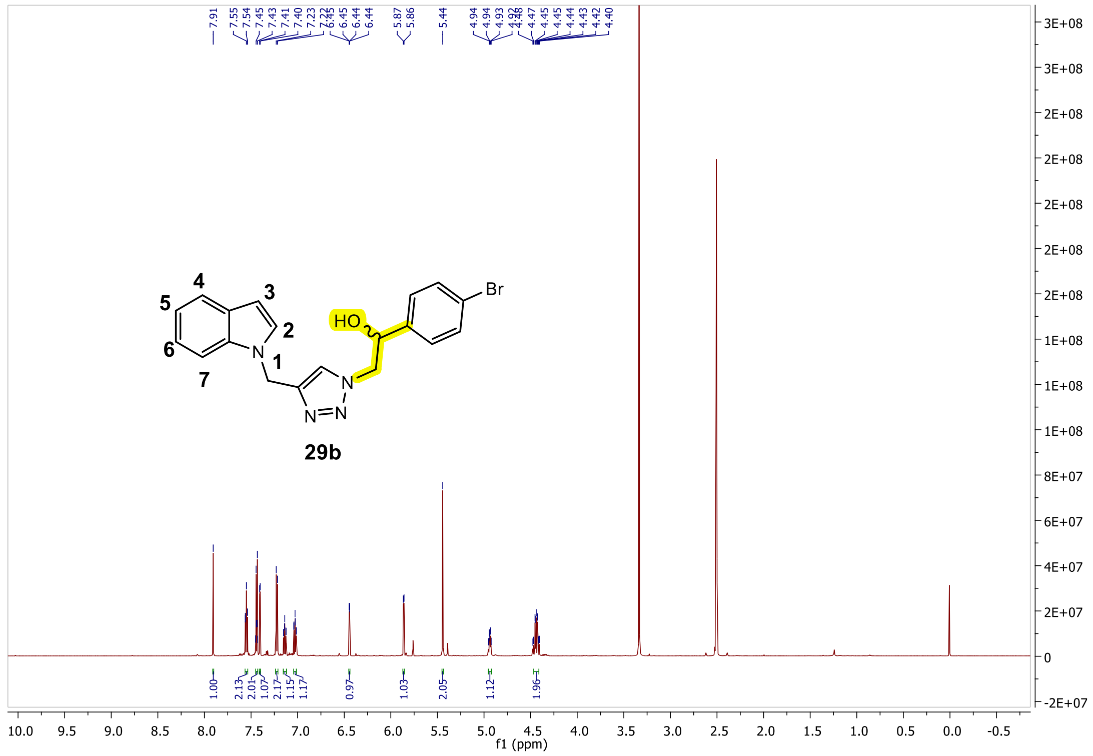

b)

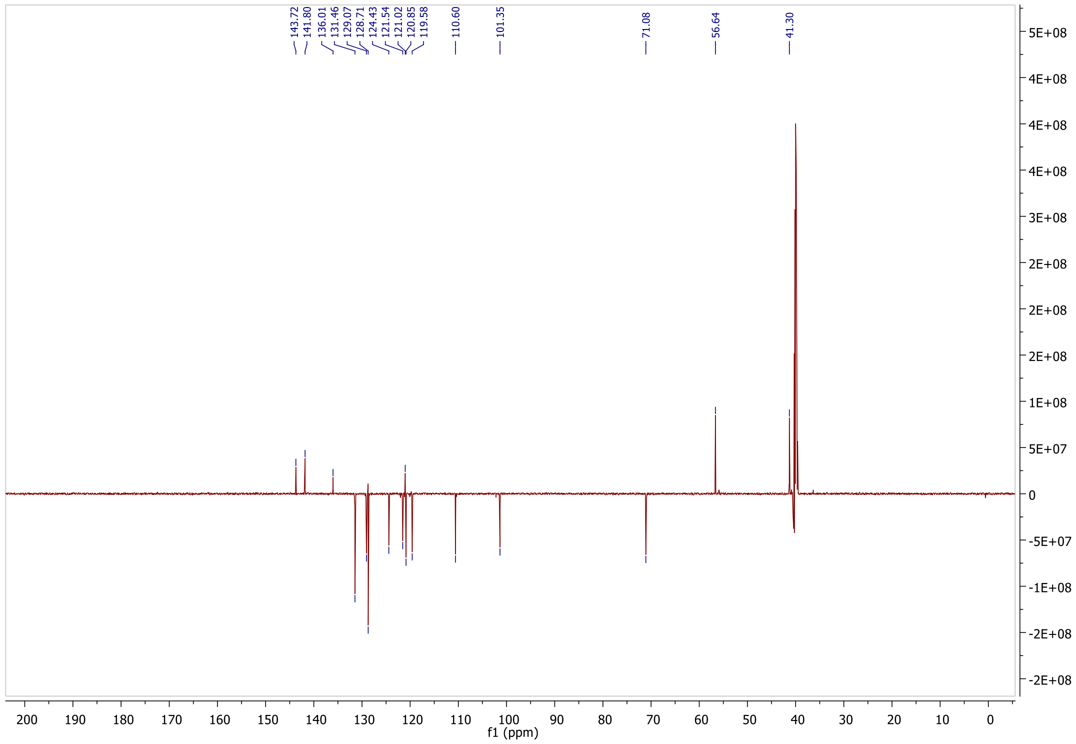

Fig. S29 a) ^{1}H NMR and b) ^{13}C NMR of compd. **30b**

a)

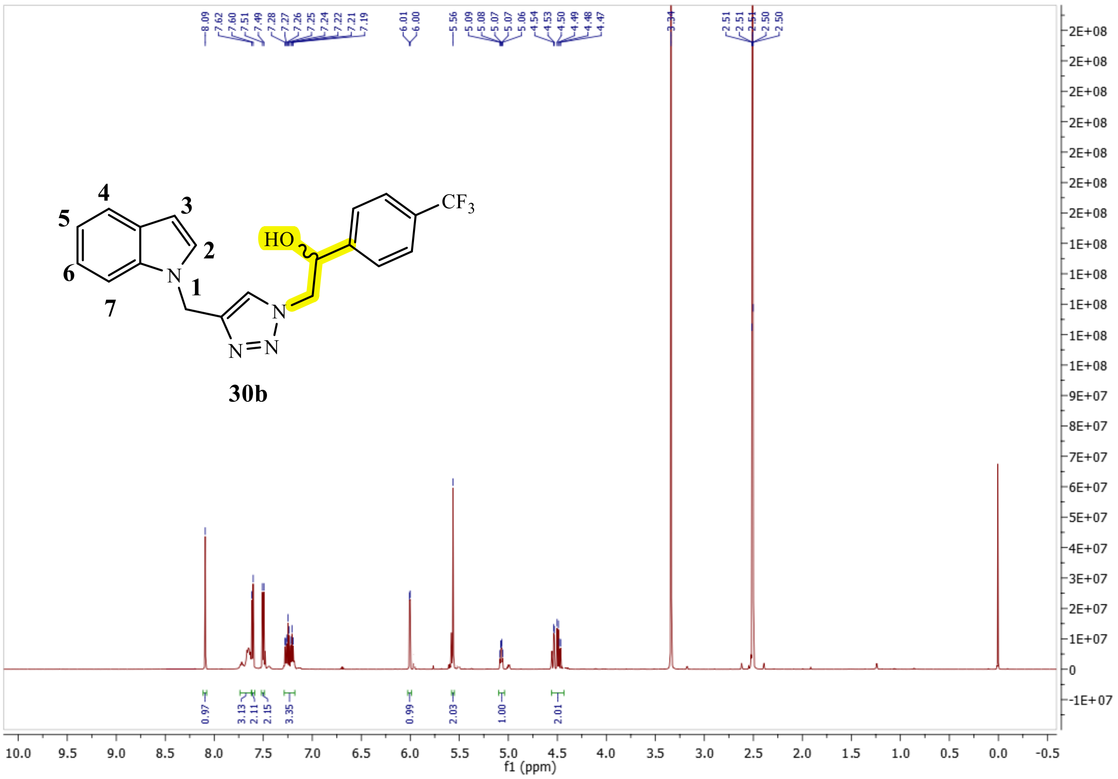

b)

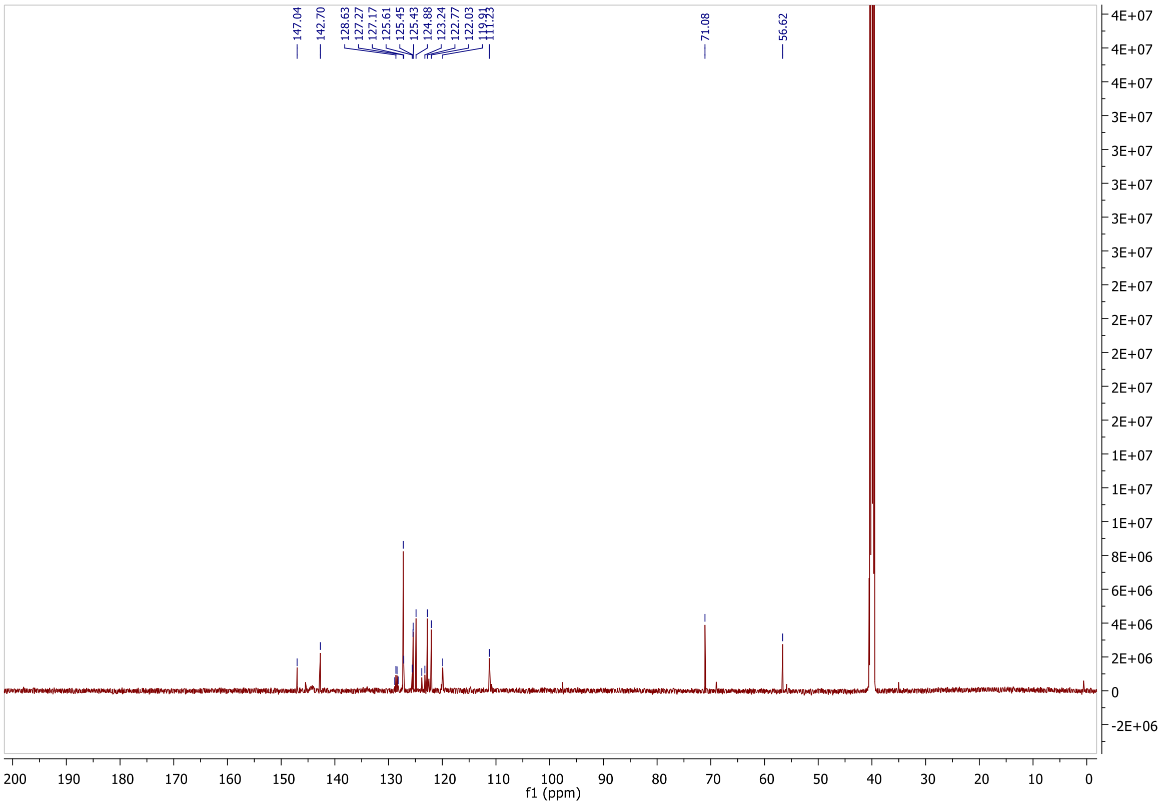

Fig. S30 a) ^{1}H NMR and b) ^{13}C NMR of compd. **31b**

a)

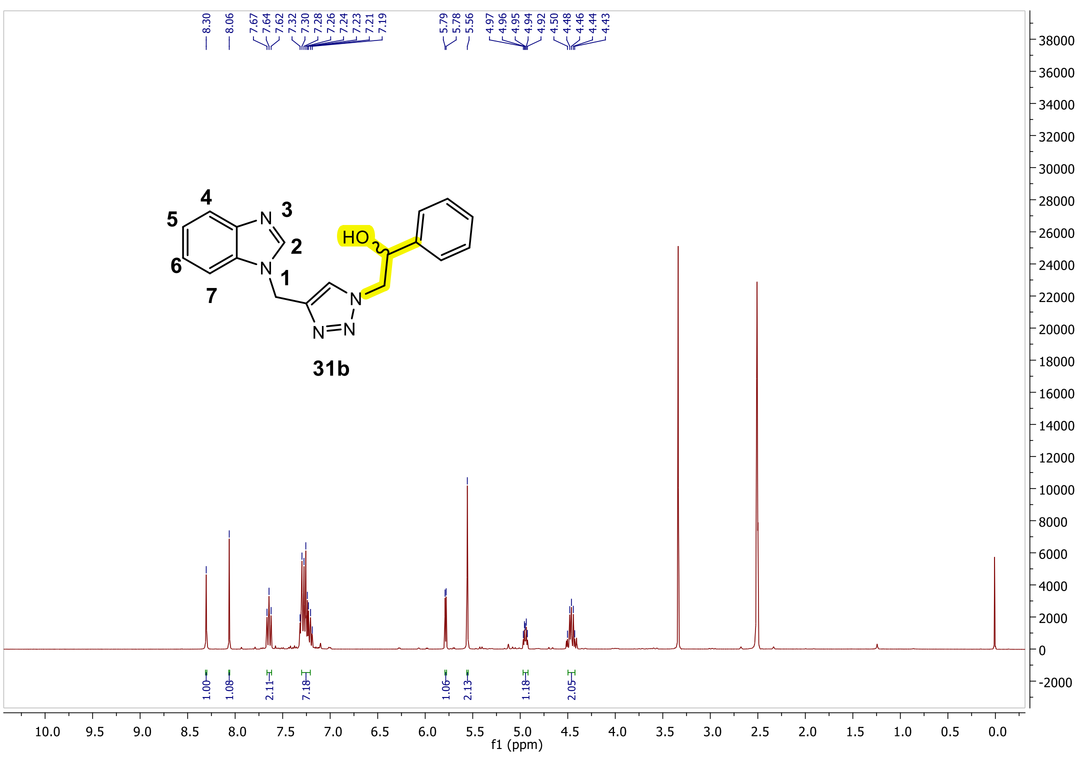

b)

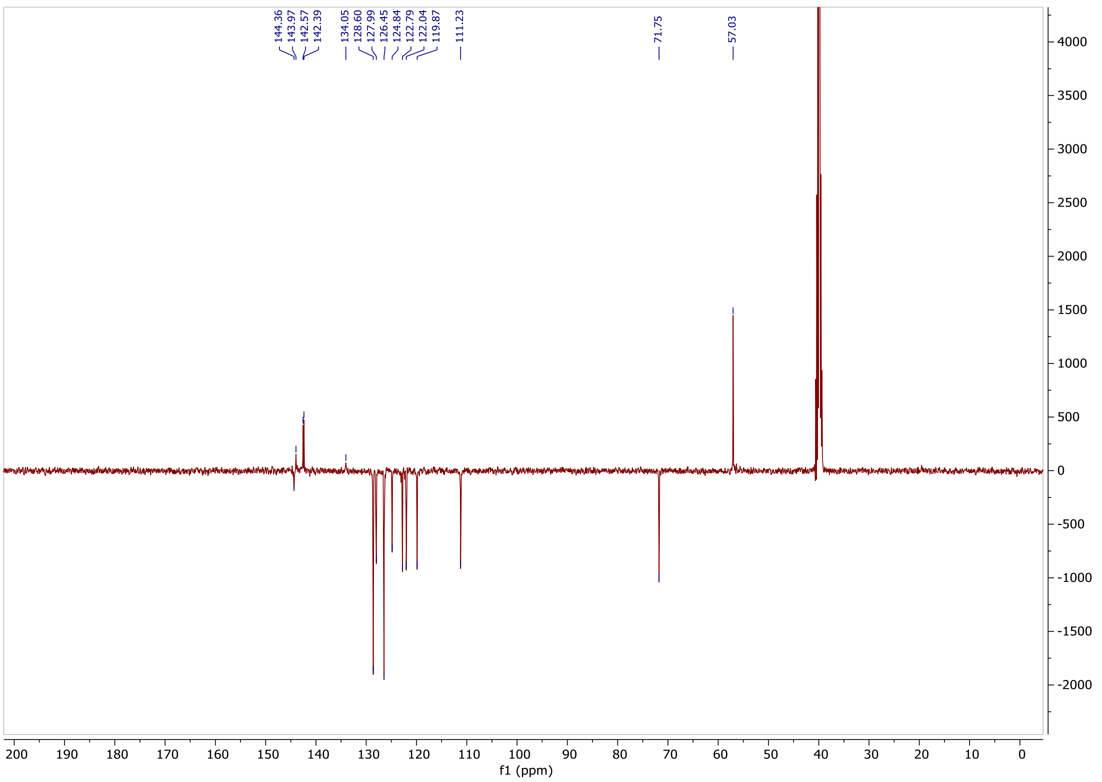

Fig. S31 a) ^{1}H NMR and b) ^{13}C NMR of compd. **32b**

a)

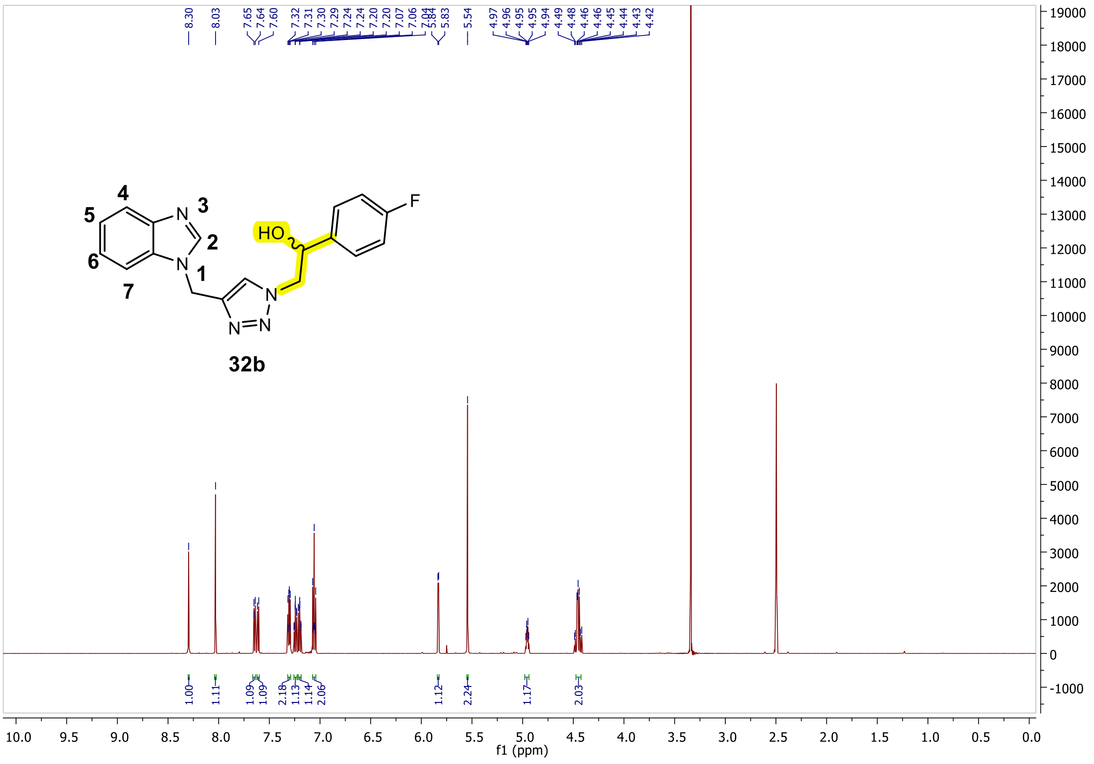

b)

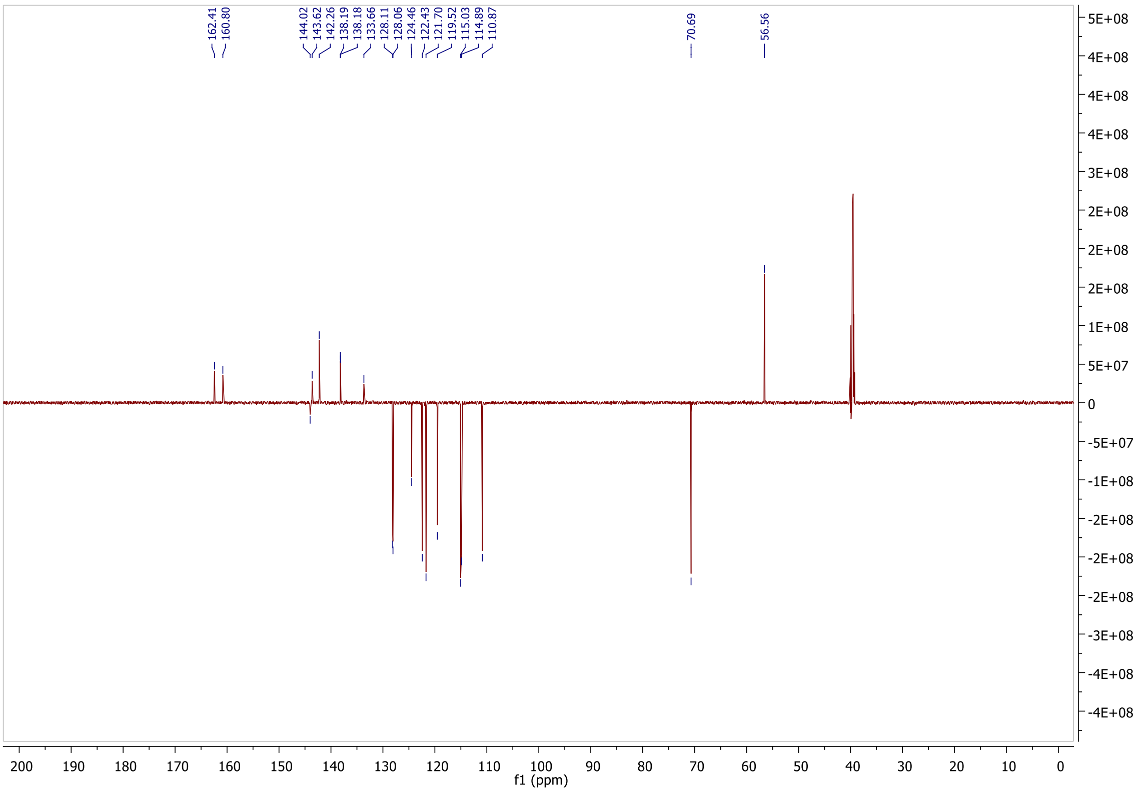

Fig. S32 a) ^{1}H NMR and b) ^{13}C NMR of compd. **33b**

a)

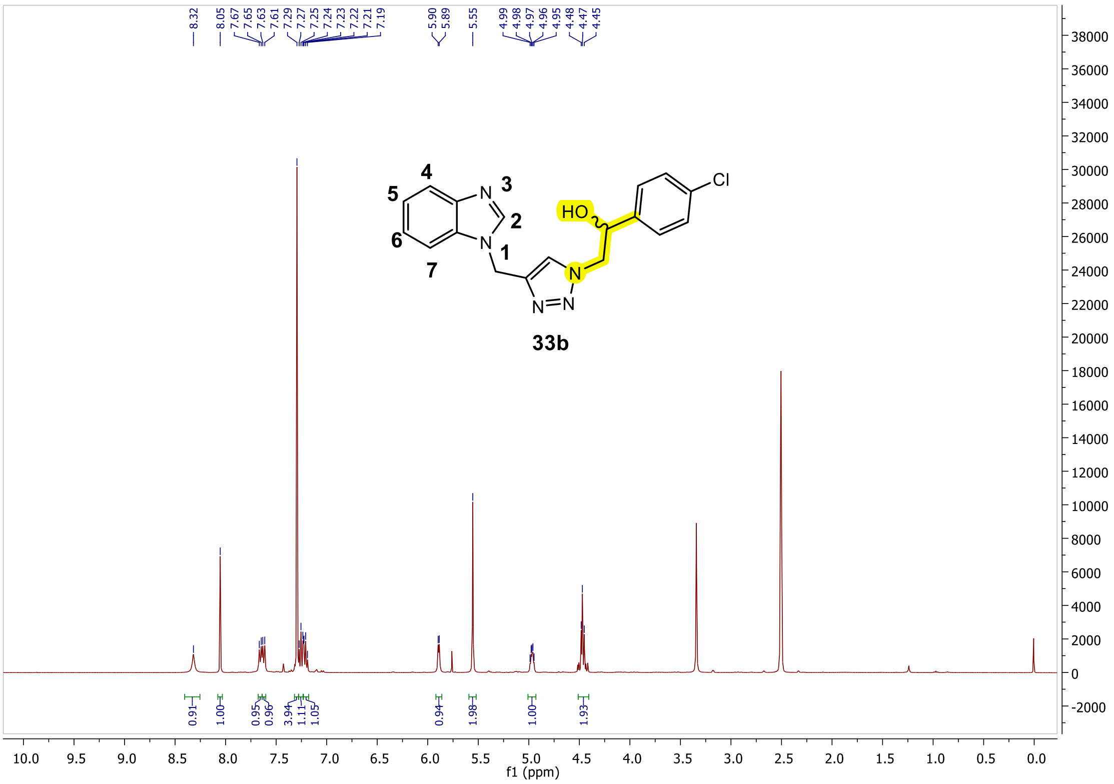

b)

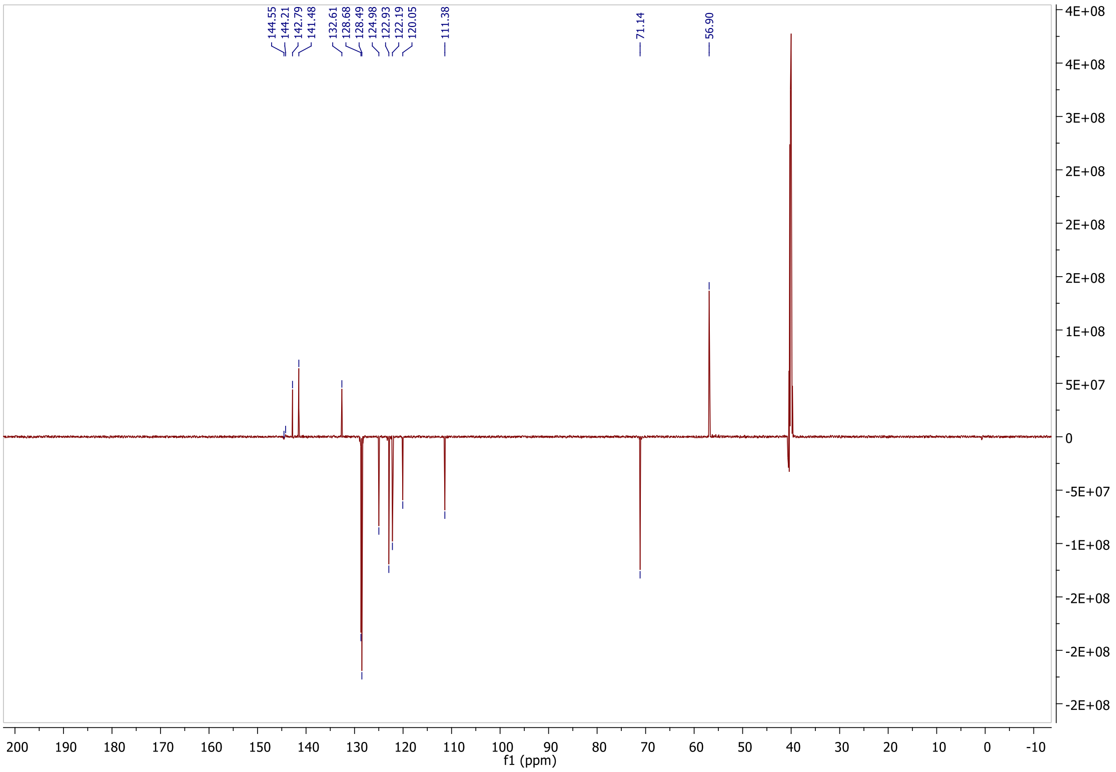

Fig. S33 a) ^{1}H NMR and b) ^{13}C NMR of compd. **34b**

a)

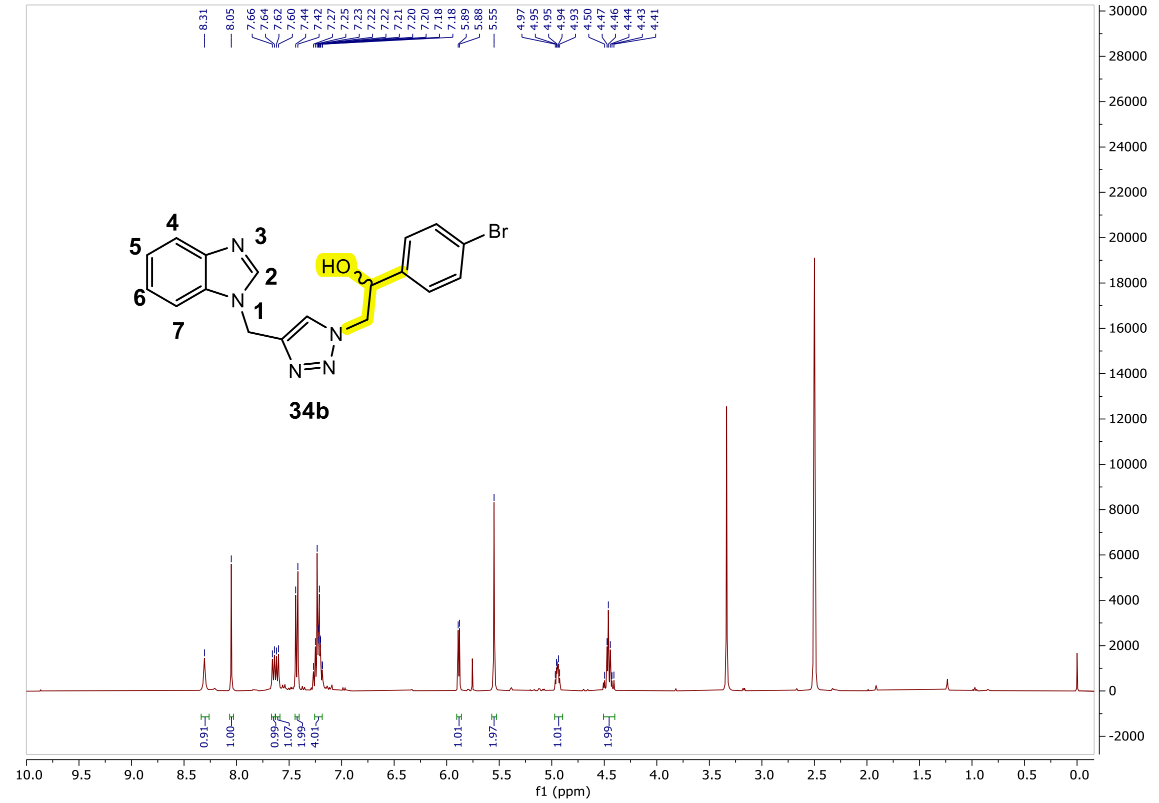

b)

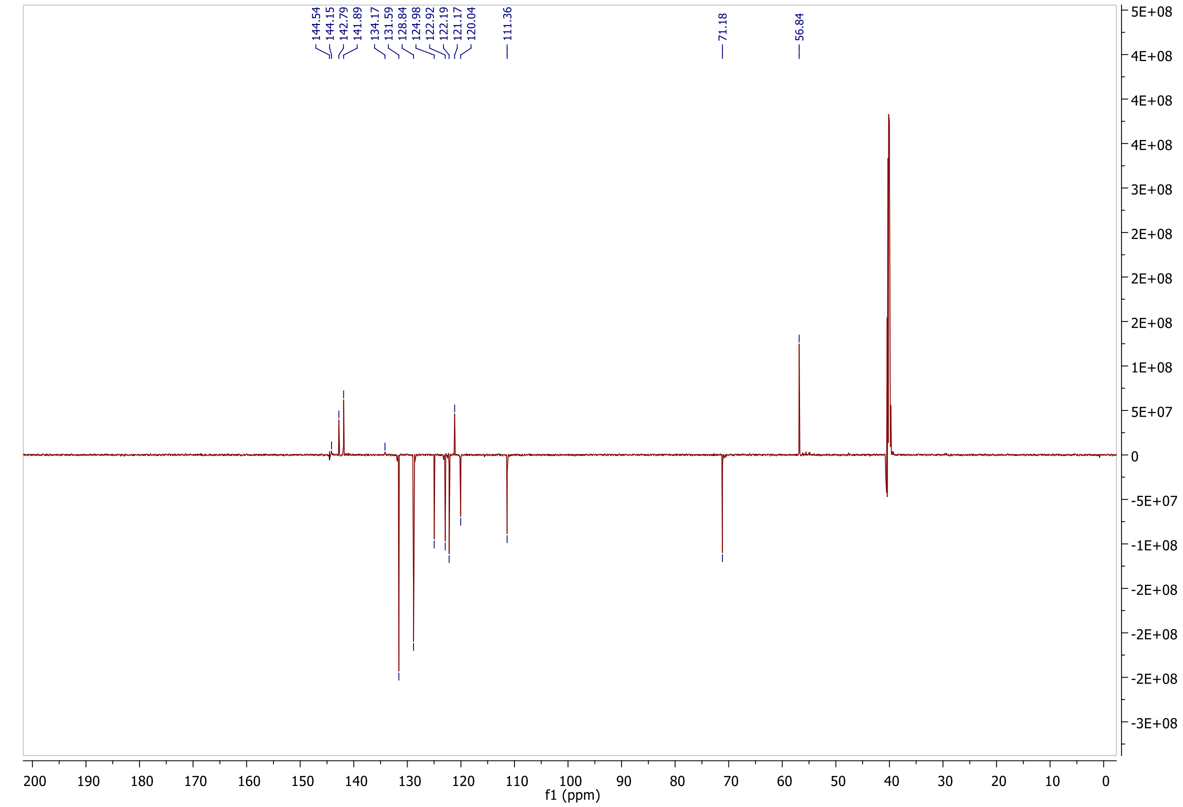

Fig. S34 a) ^{1}H NMR and b) ^{13}C NMR of compd. **35b**

a)

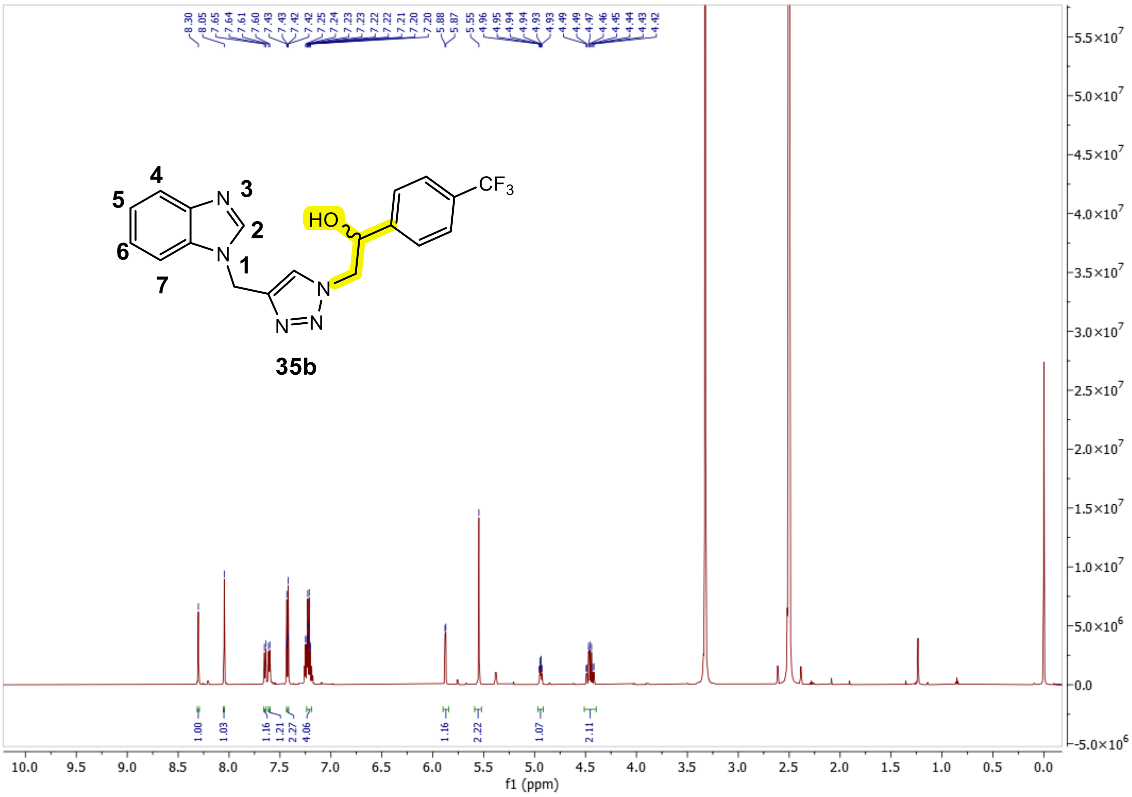

b)

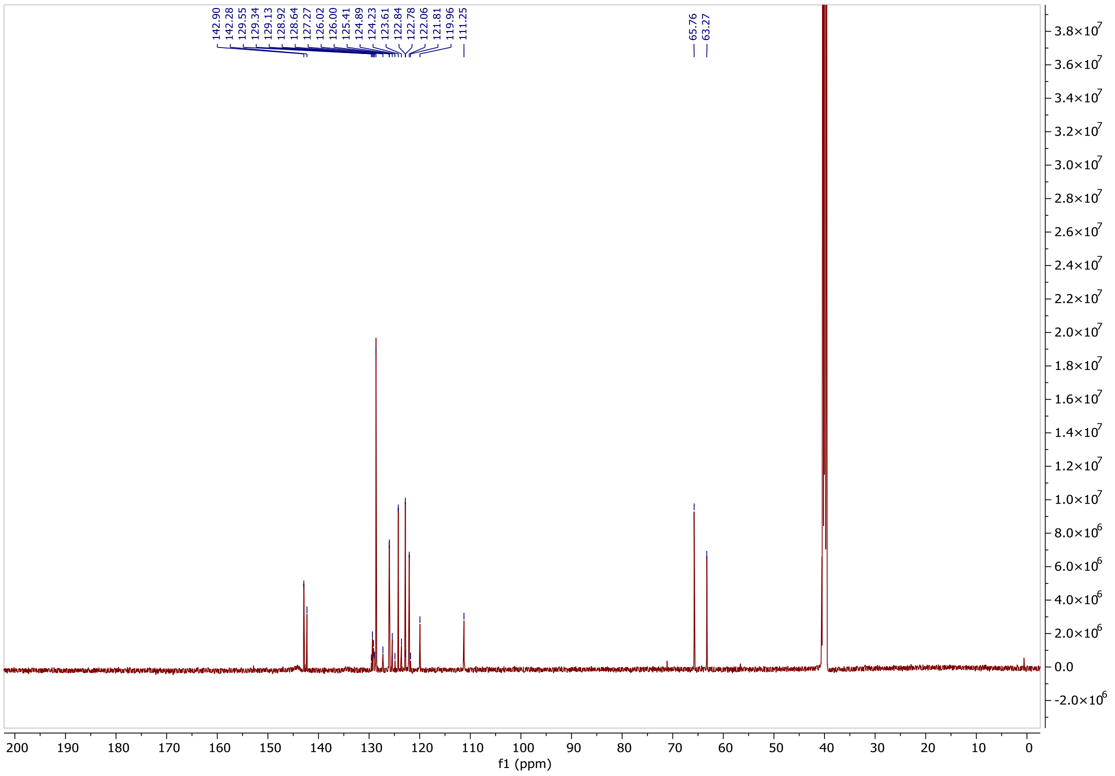

Fig. S35 a) ^{1}H NMR and b) ^{13}C NMR of compounds **36b,** (*R*)-**36b and** (*S*)-**36b**

a)

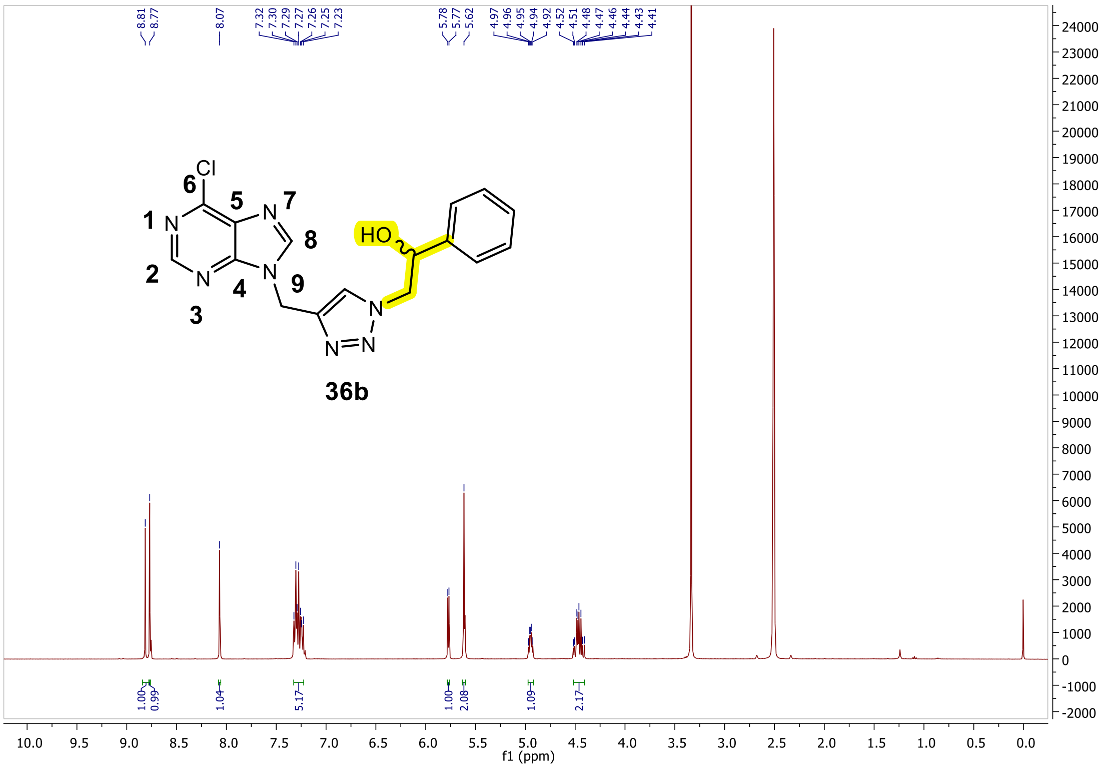

b)

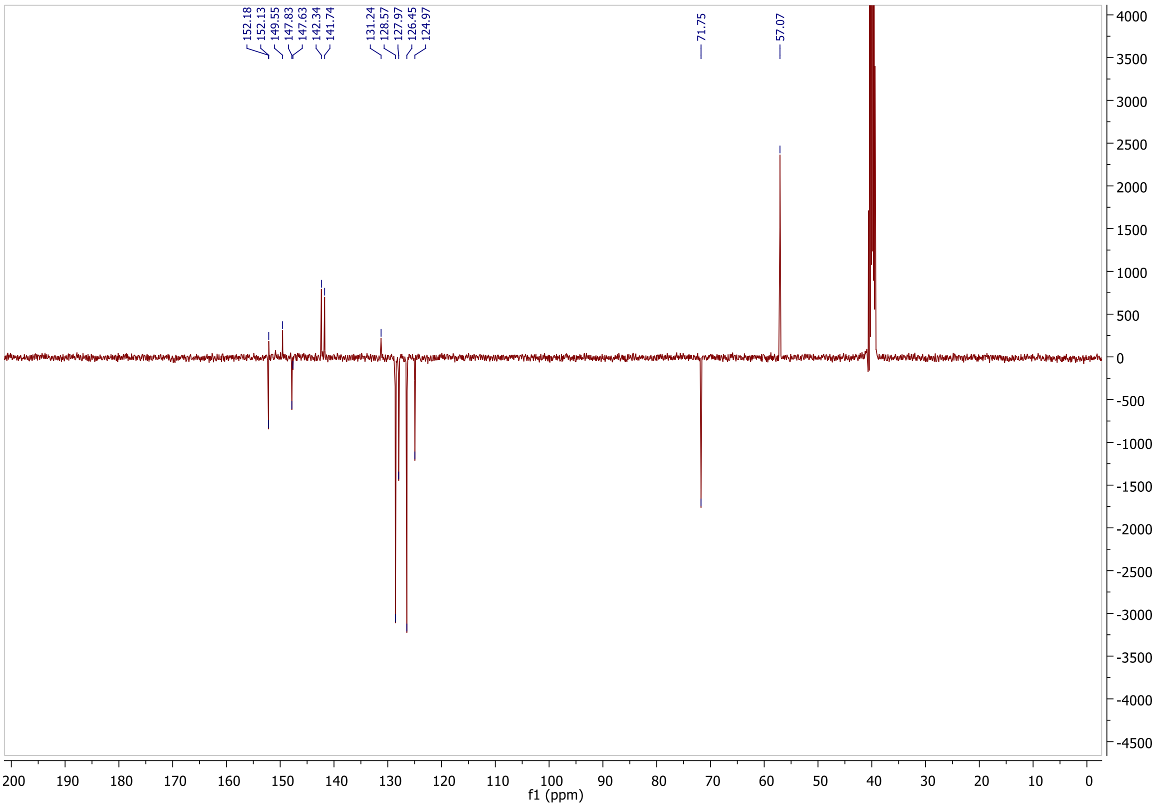

Fig. S36 a) ^{1}H NMR and b) ^{13}C NMR of compounds **37b,** (*R*)-**37b** and (*S*)-**37b**

a)

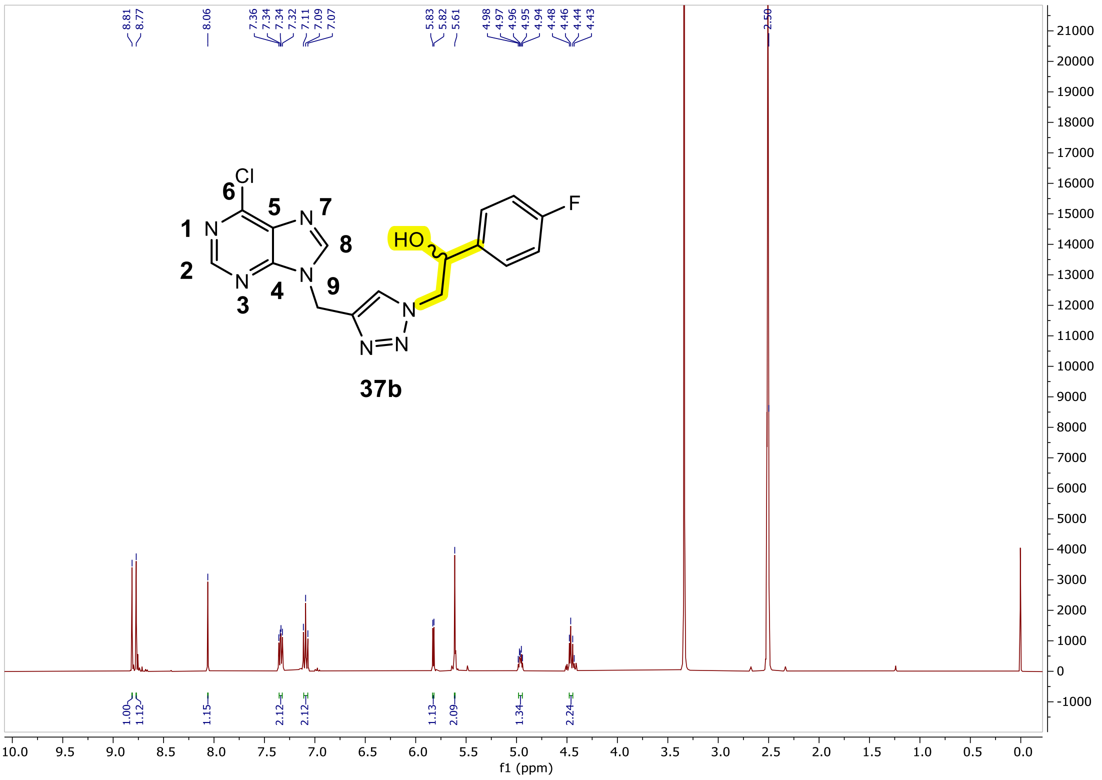

b)

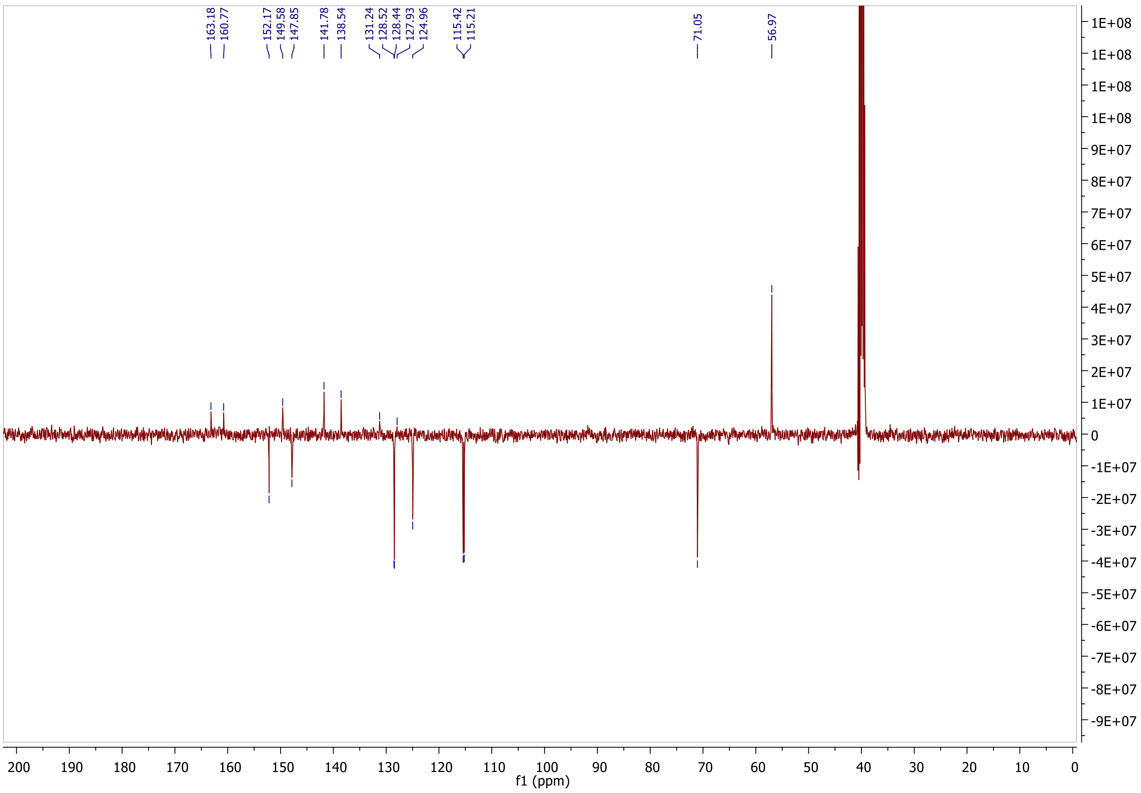

Fig. S37 a) ^{1}H NMR and b) ^{13}C NMR of compounds **38b,** (*R*)-**38b** and (*S*)-**38b**

a)

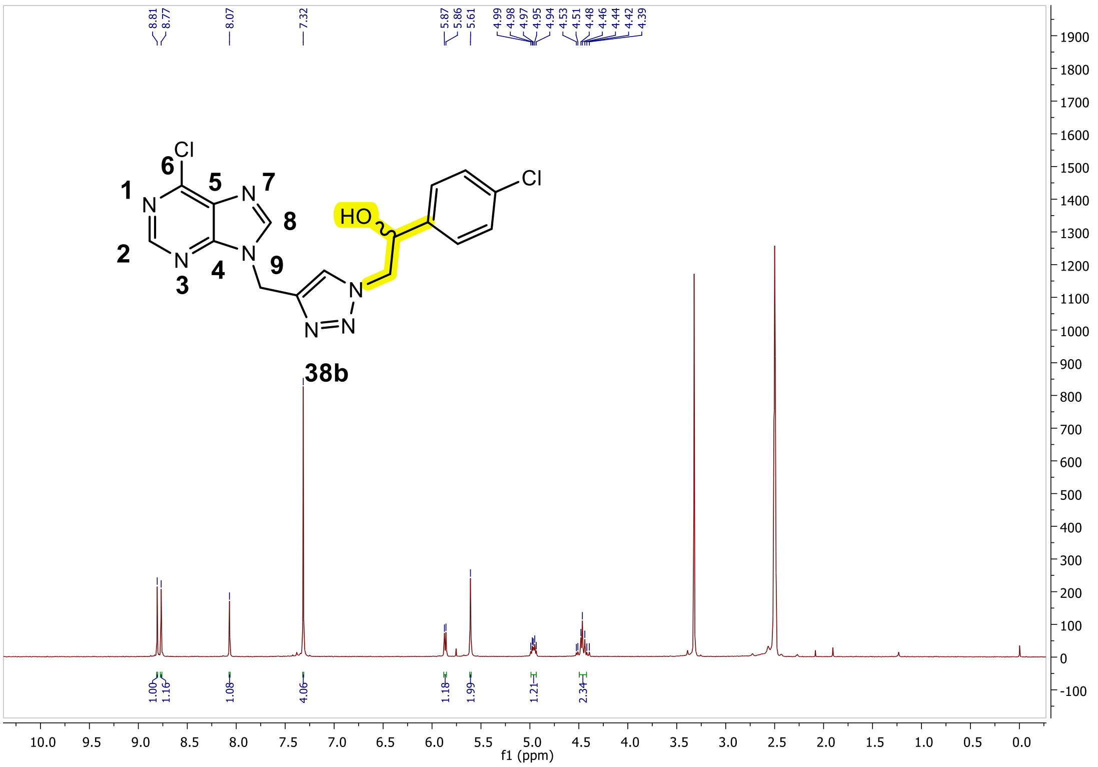

b)

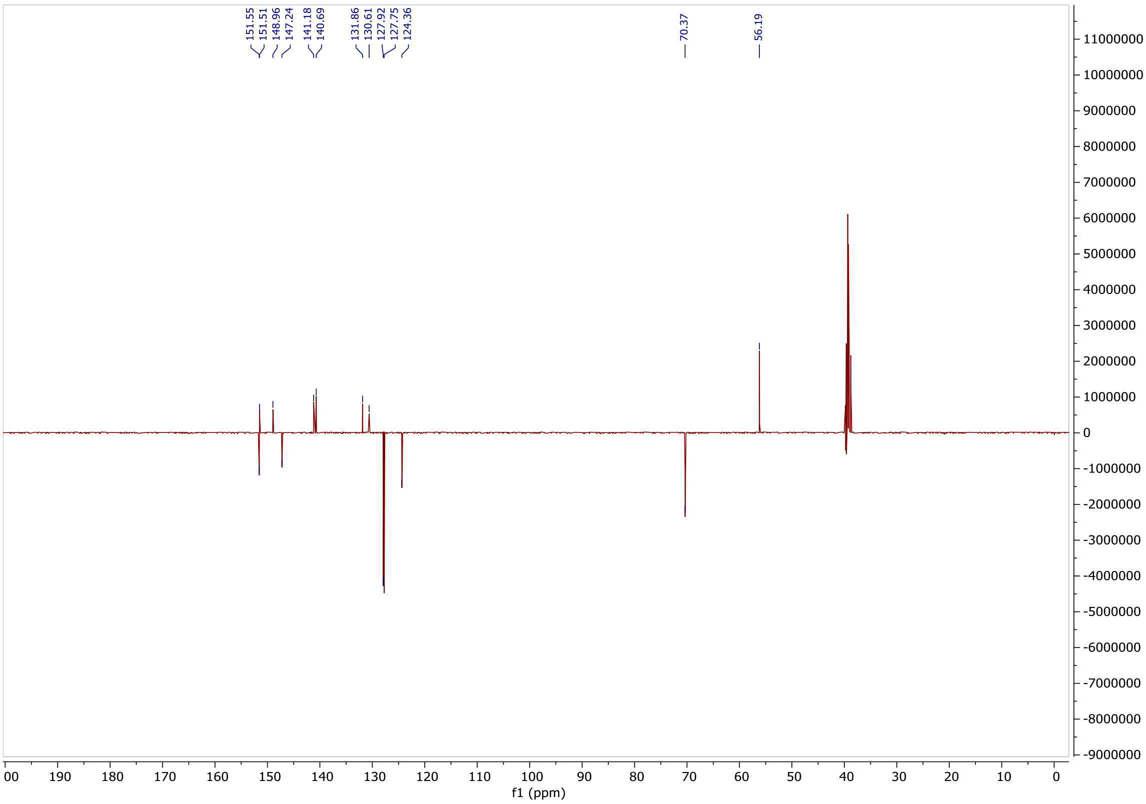

Fig. S38 a) ^{1}H NMR and b) ^{13}C NMR of compd. **39b**

a)

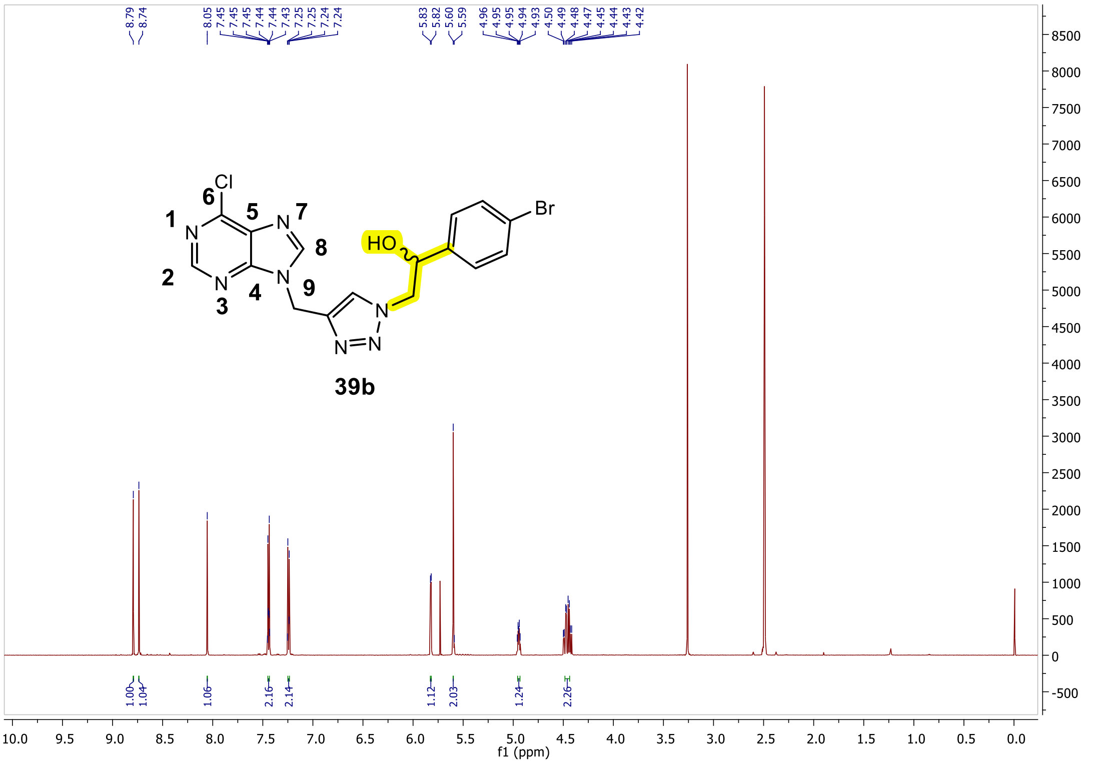

b)

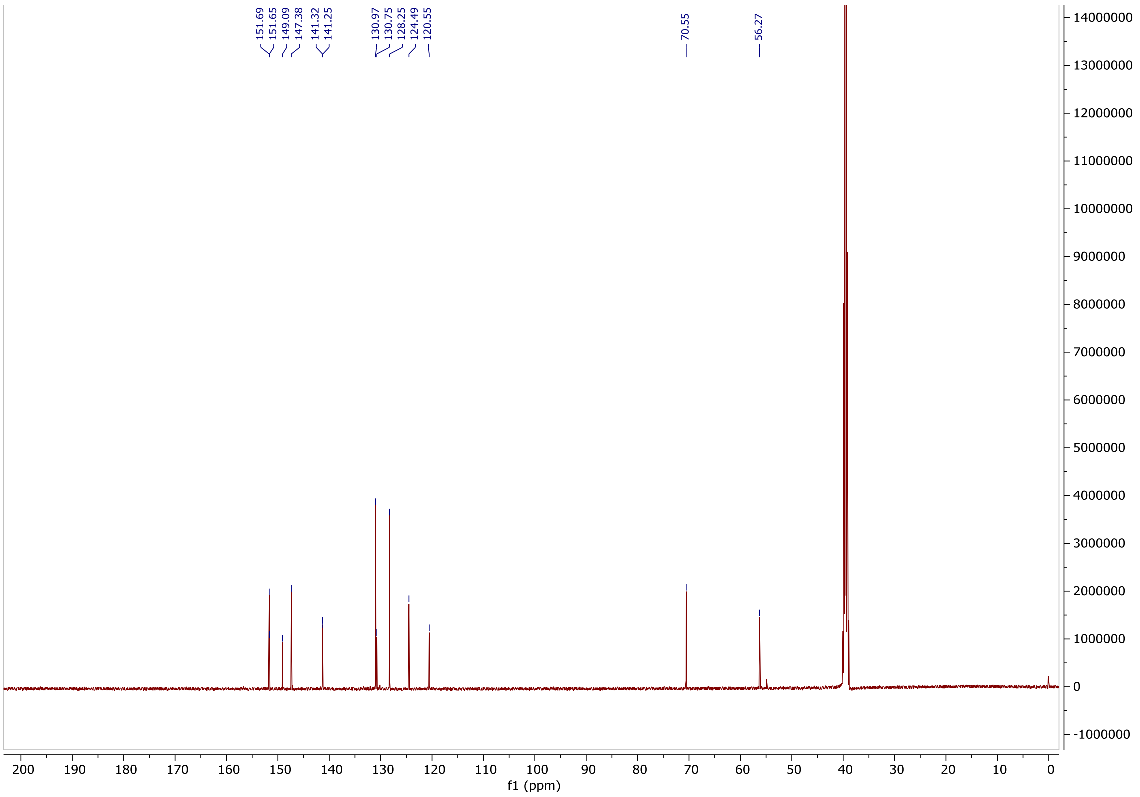

Fig. S39 a) ^{1}H NMR and b) ^{13}C NMR of compounds **40b,** (*R*)-**40b** and (*S*)-**40b**

a)

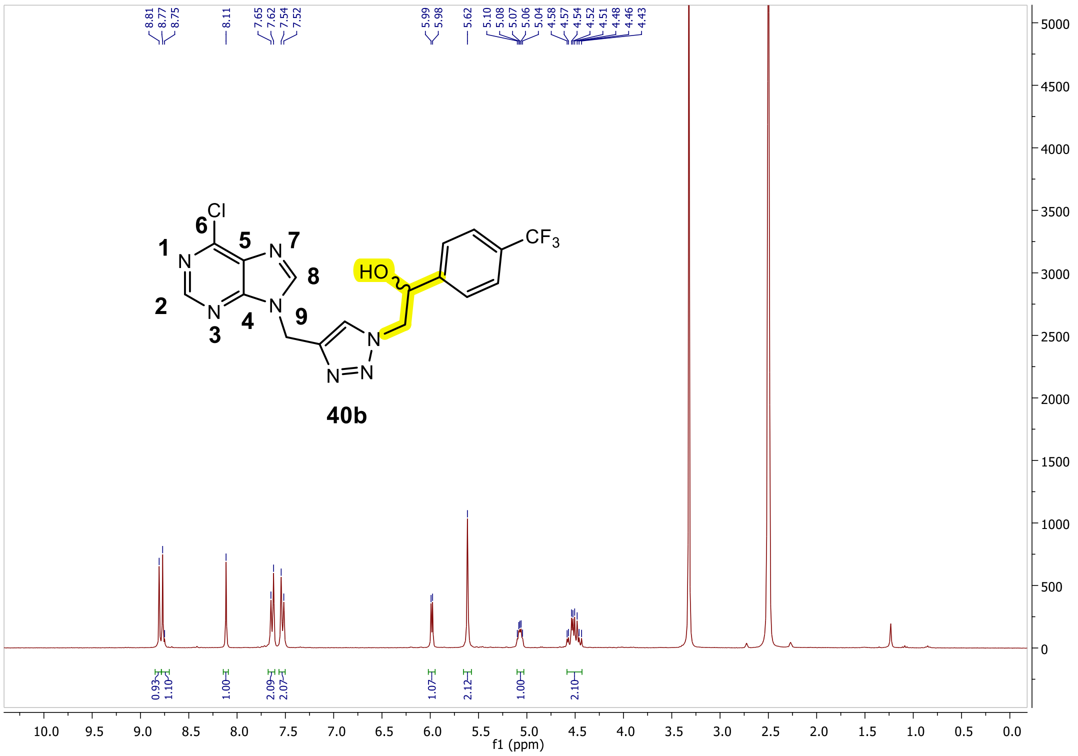

b)

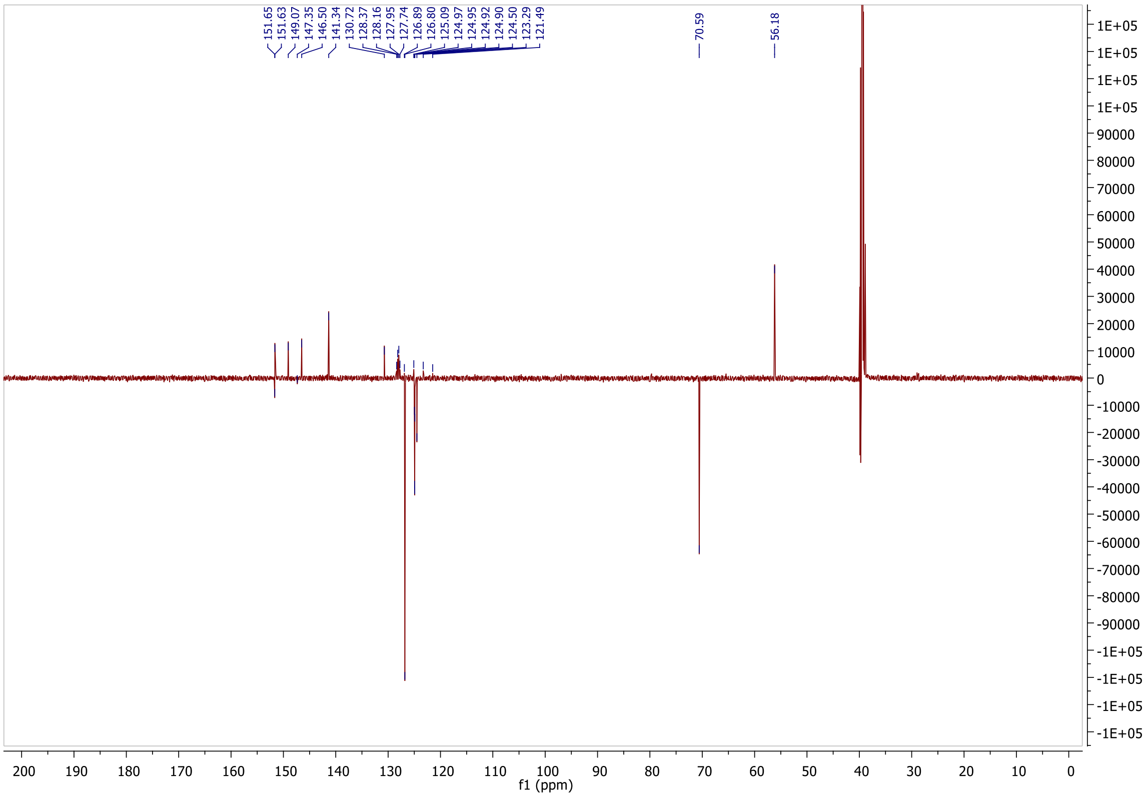

Fig. S40 a) ^{1}H NMR and b) ^{13}C NMR of compounds **41b,** (*R*)-**41b** and (*S*)-**41b**

a)

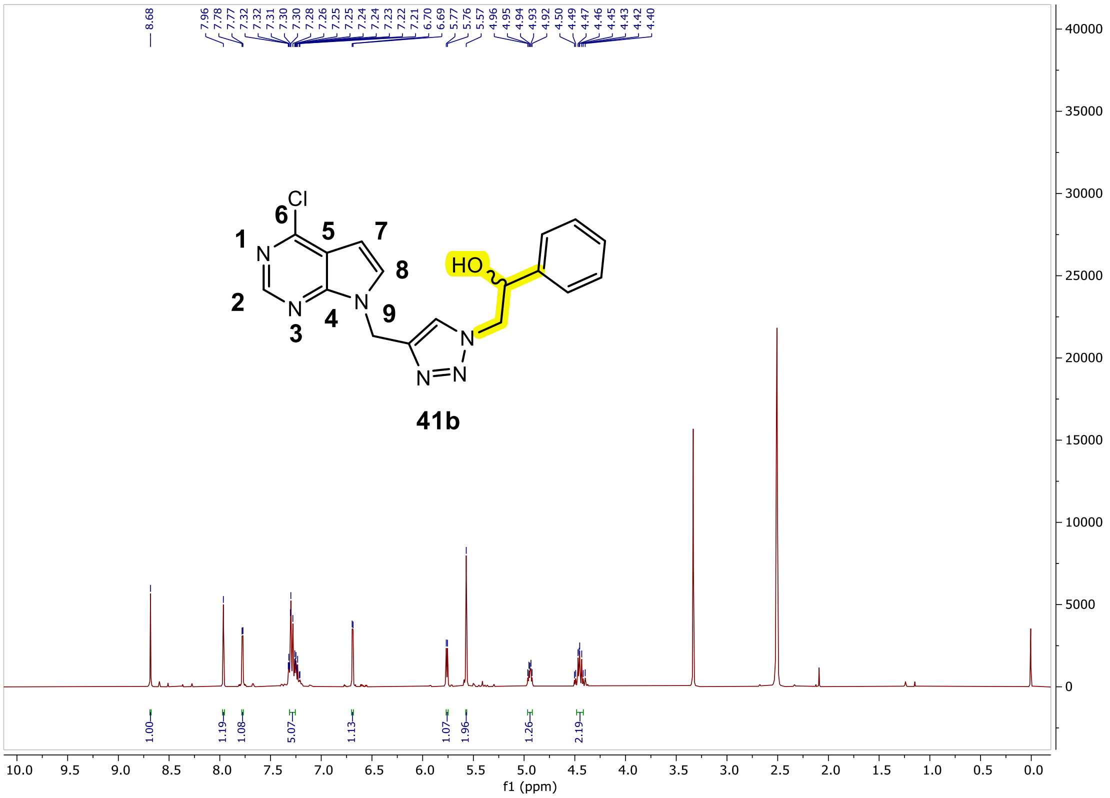

b)

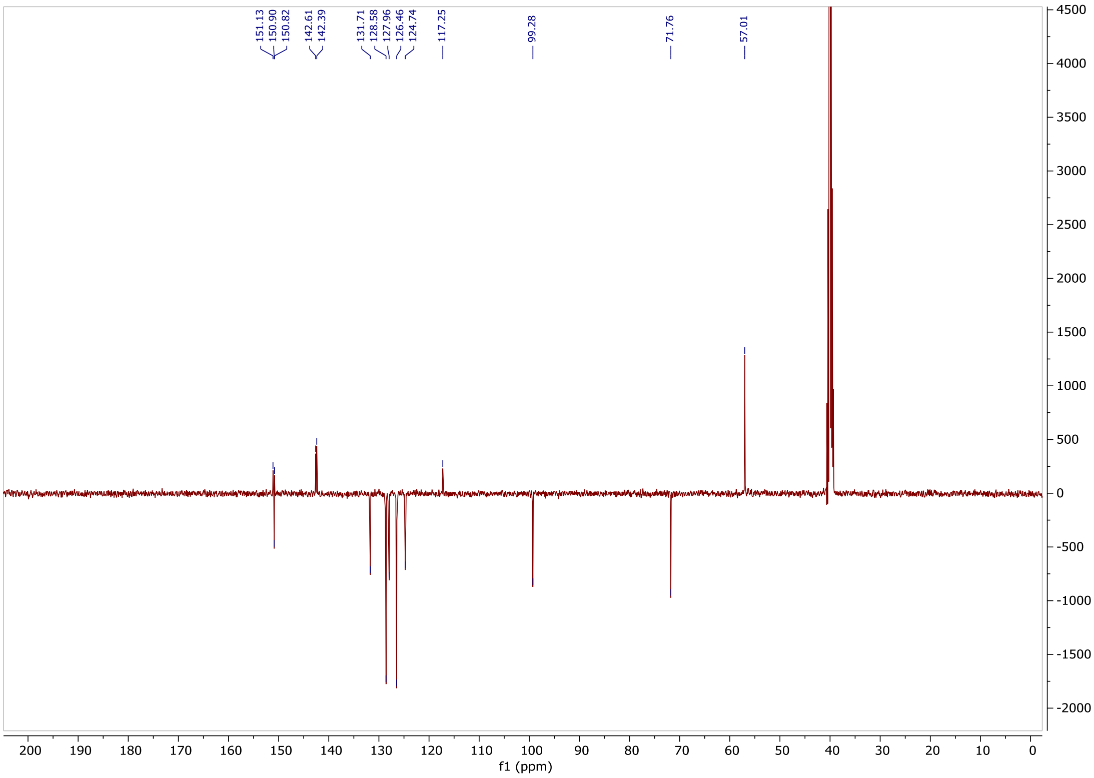

Fig. S41 a) ^{1}H NMR and b) ^{13}C NMR of compounds **42b,** (*R*)-**42b** and (*S*)-**42b**

a)

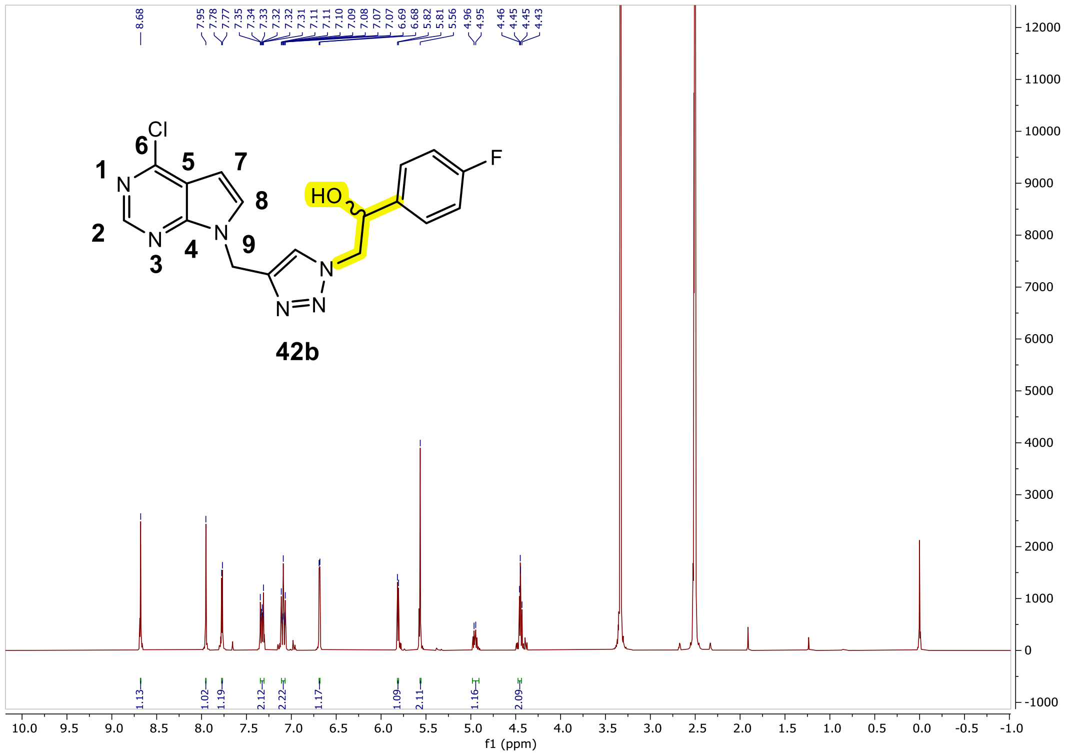

b)

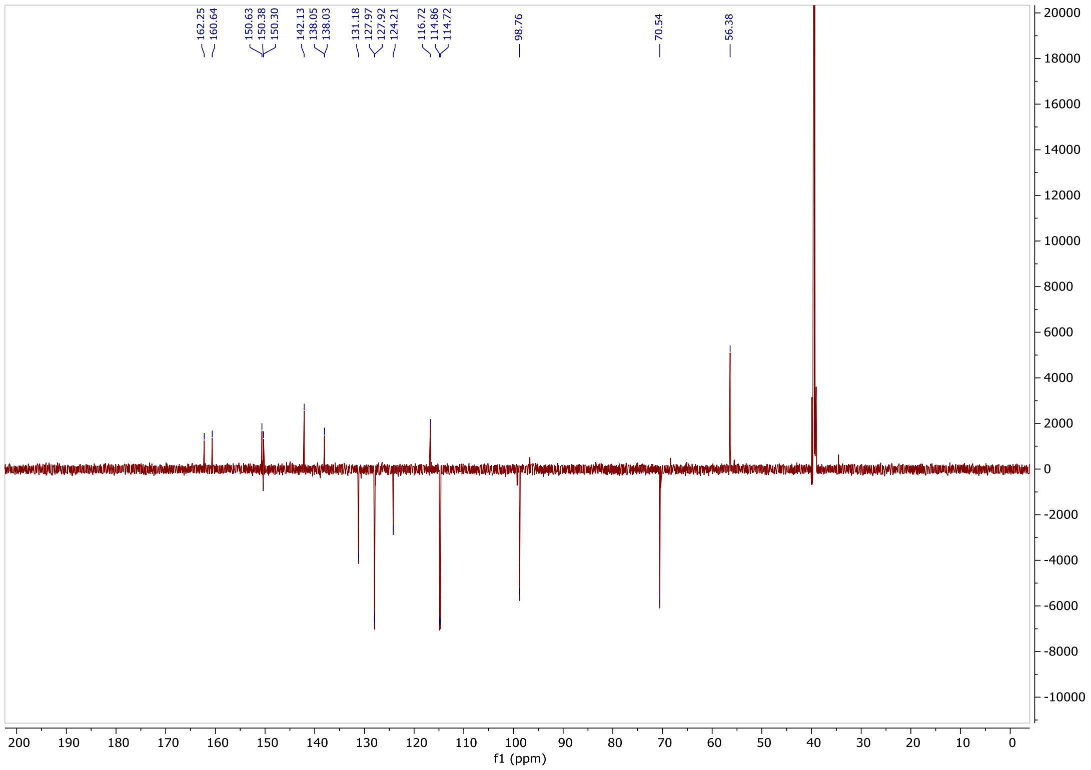

Fig. S42 a) ^{1}H NMR and b) ^{13}C NMR of compounds **43b,** (*R*)-**43b** and (*S*)-**43b**

a)

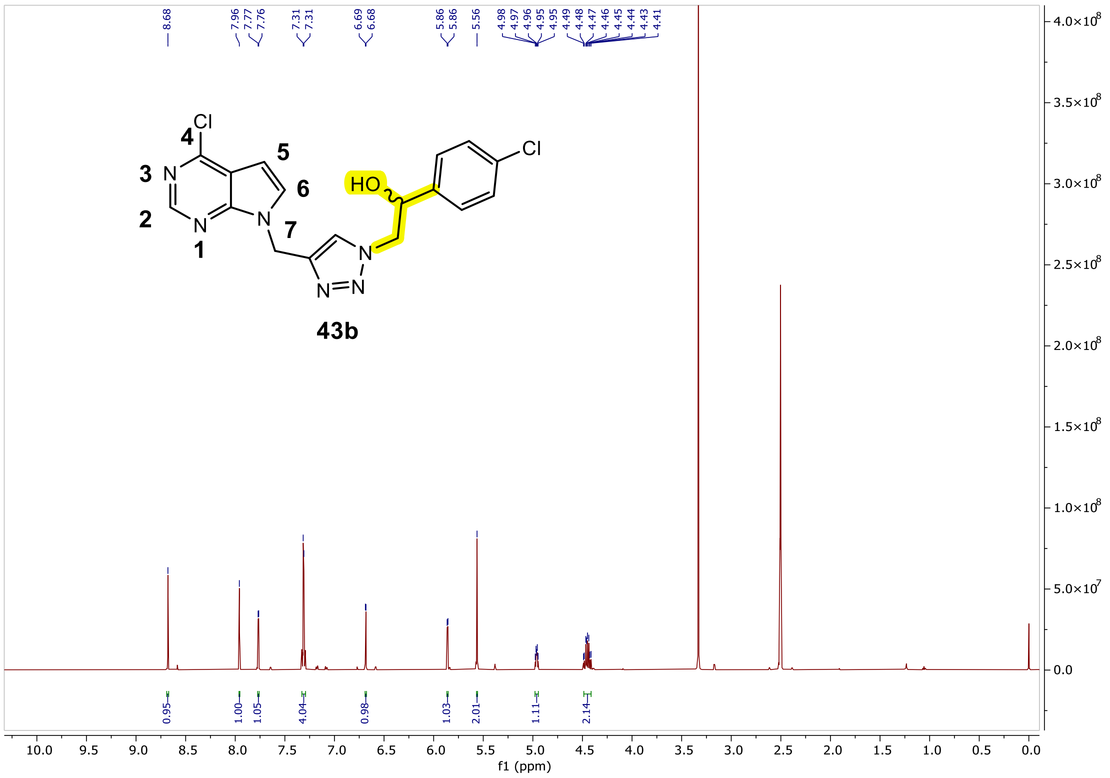

b)

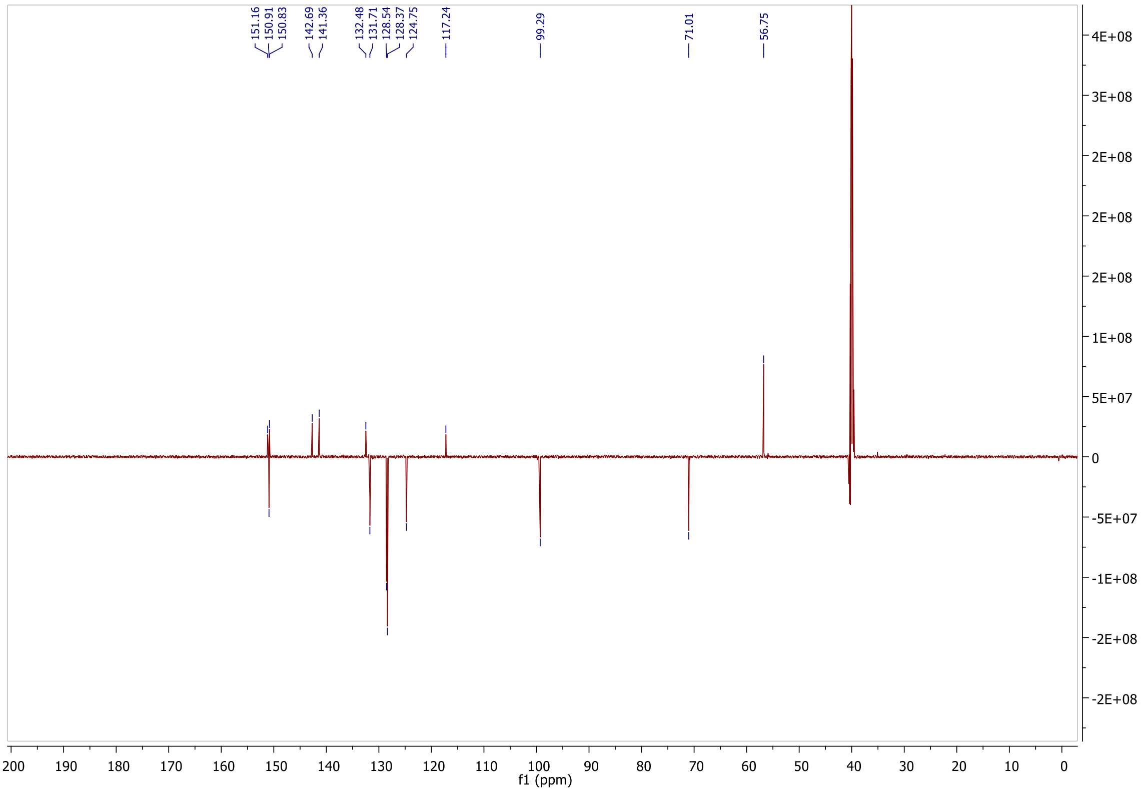

Fig. S43 a) ^{1}H NMR and b) ^{13}C NMR of compd. **44b**

a)

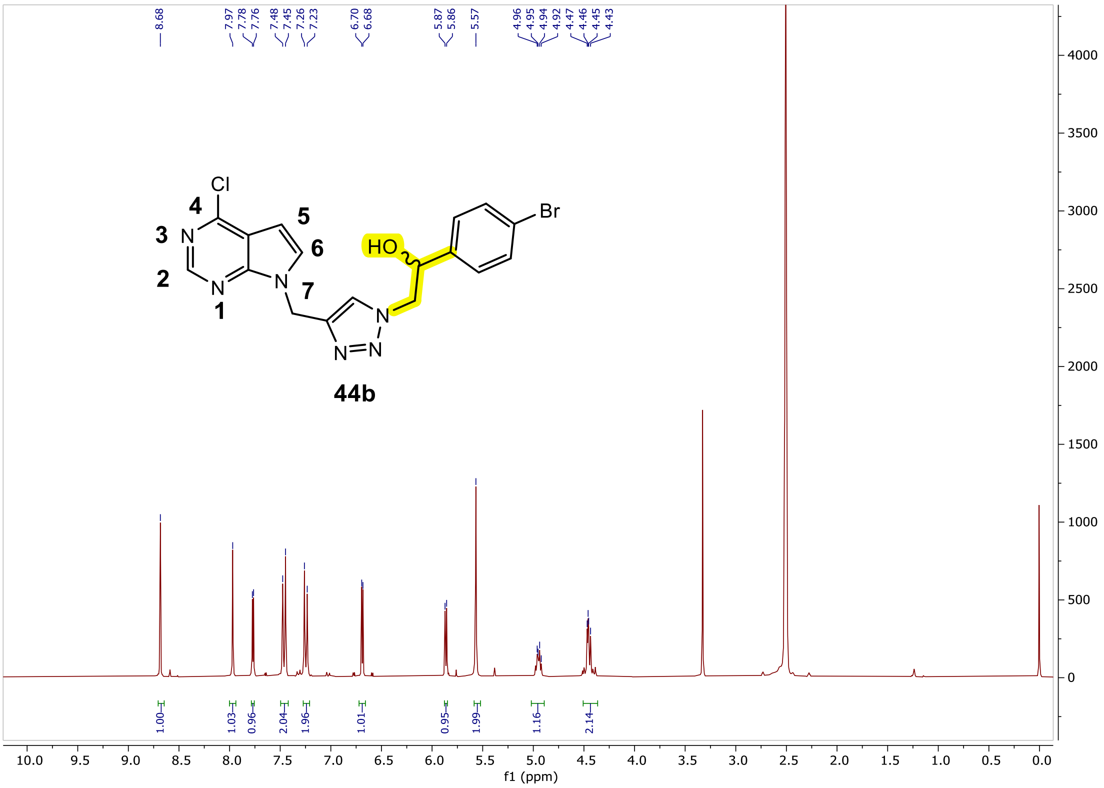

b)

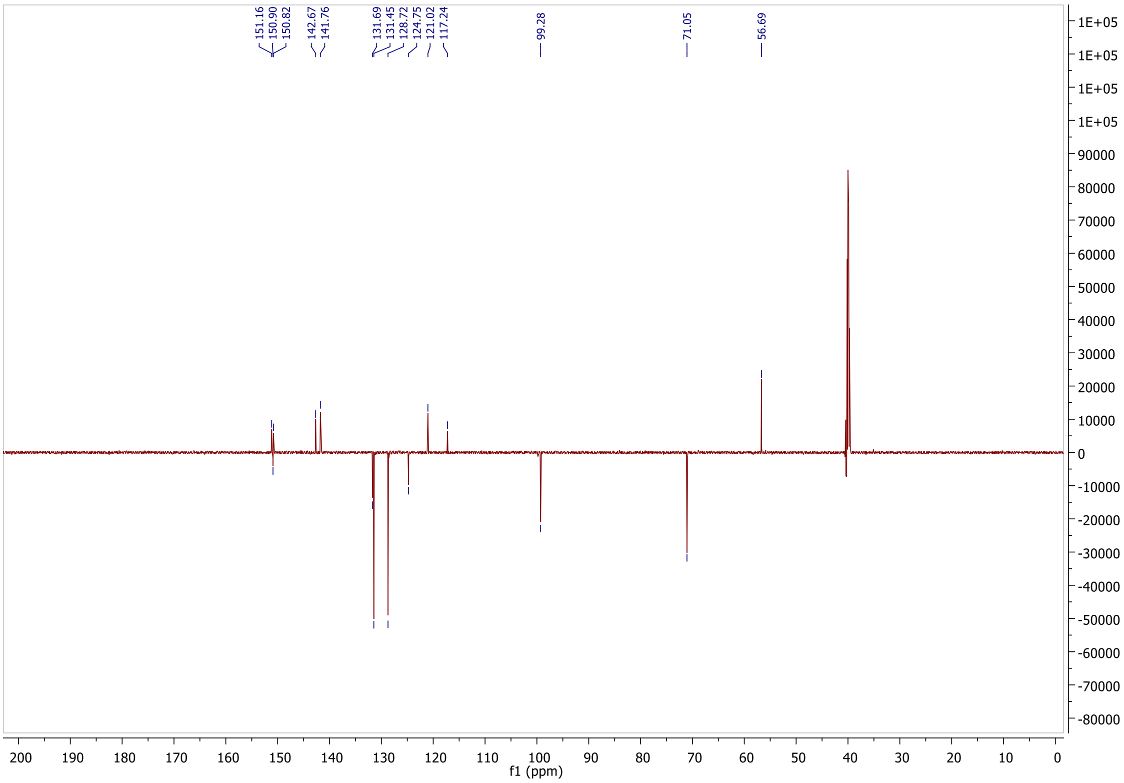

Fig. S44 a) ^{1}H NMR and b) ^{13}C NMR of compounds **45b,** (*R*)-**45b** and (*S*)-**45b**

a)

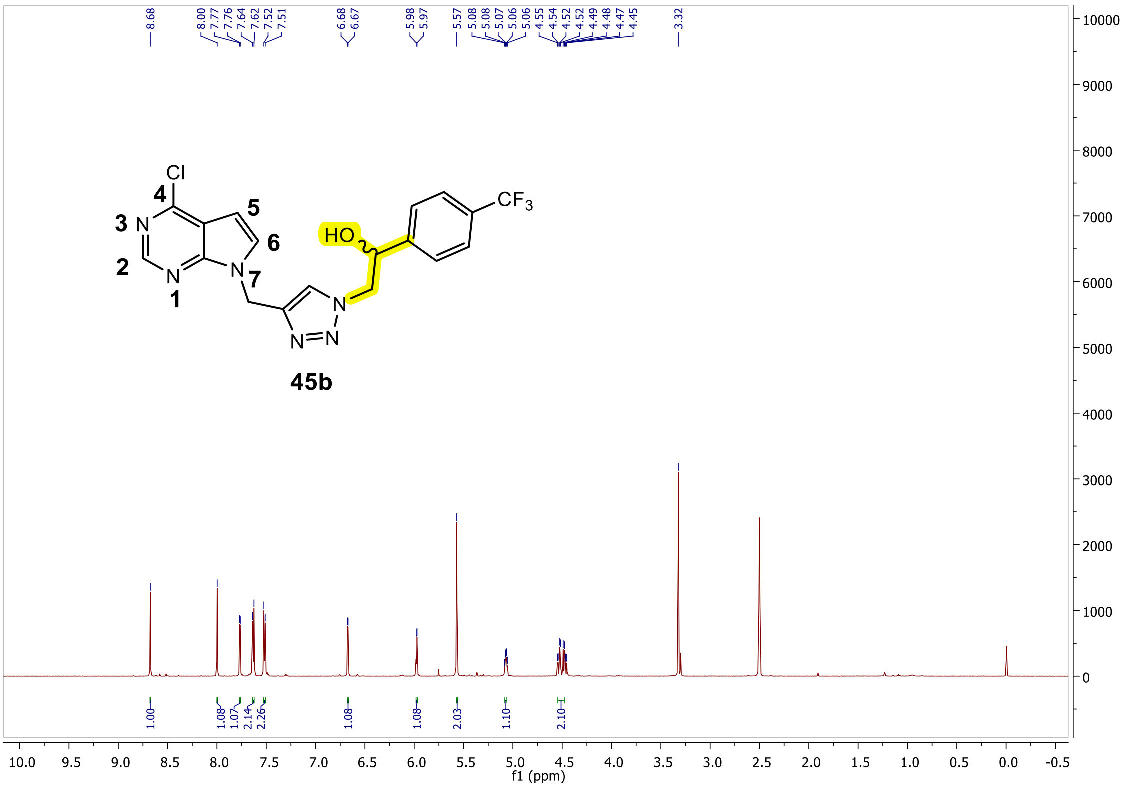

b)

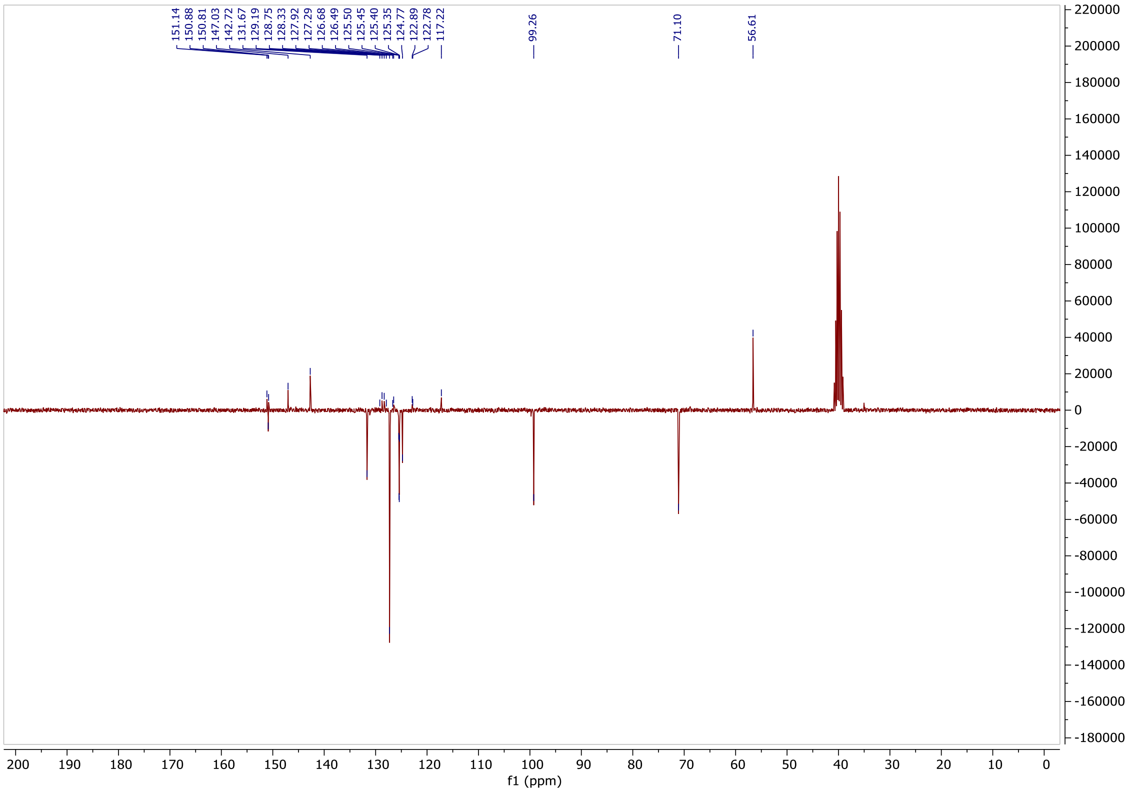

Fig. S45 a) ^{1}H NMR and b) ^{13}C NMR of compd. **46b**

a)

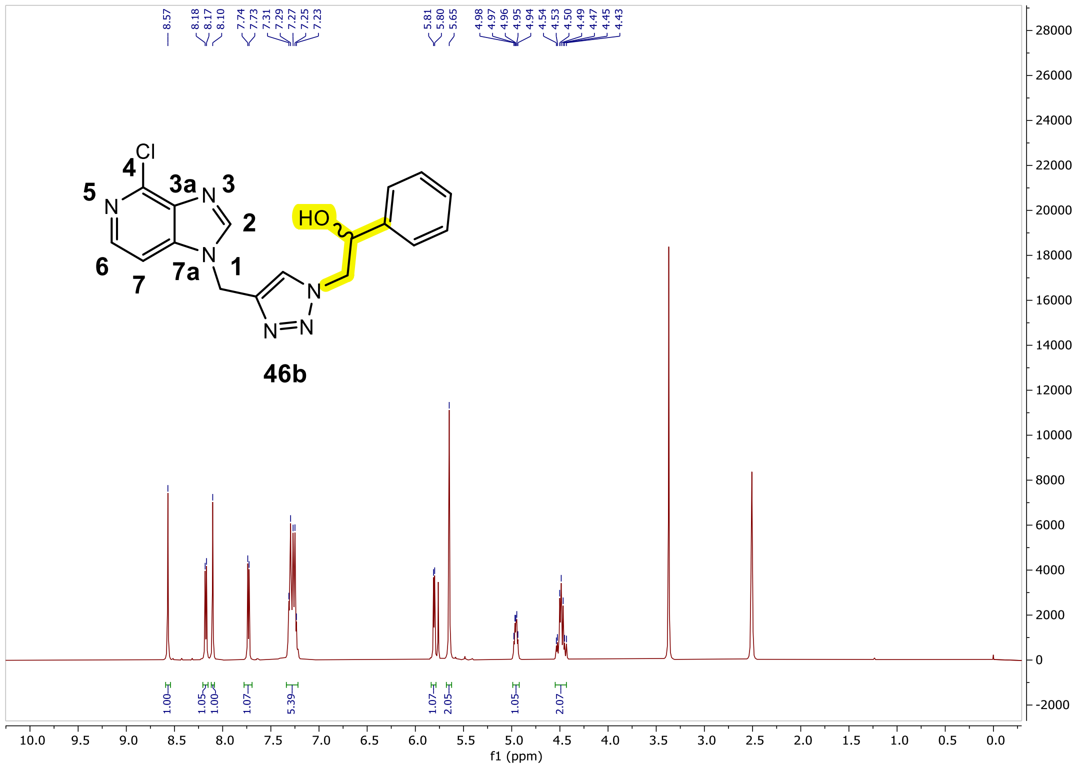

b)

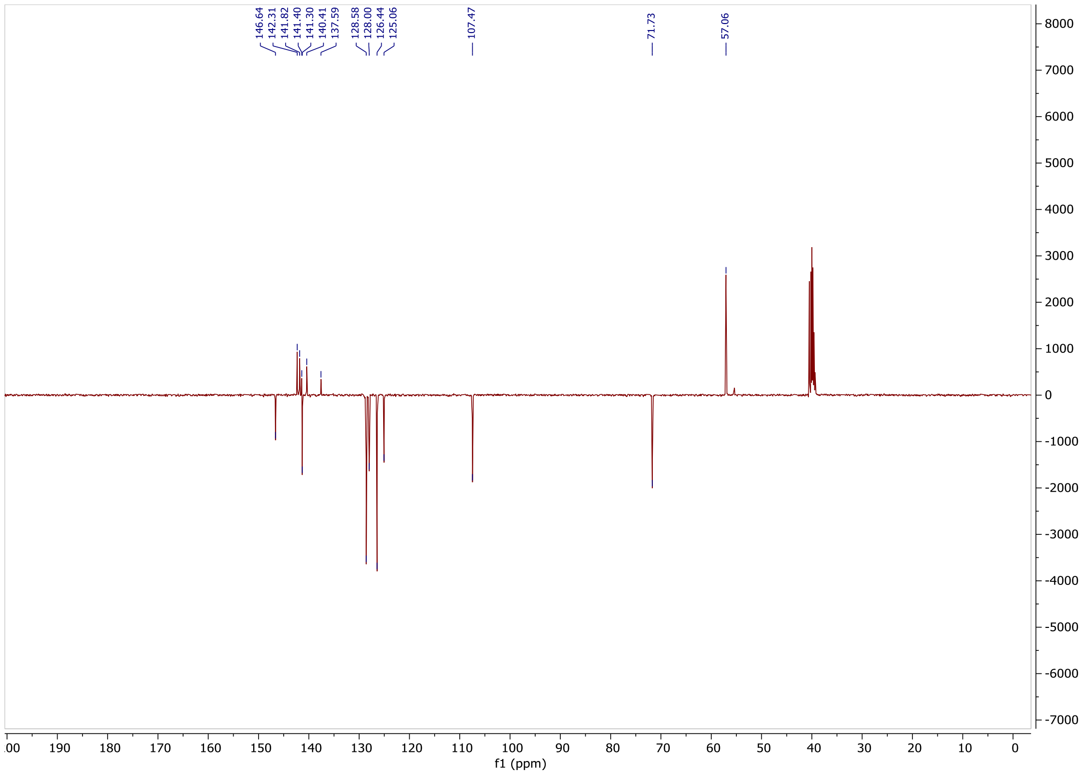

Fig. S46 a) ^{1}H NMR and b) ^{13}C NMR of compd. **47b**

a)

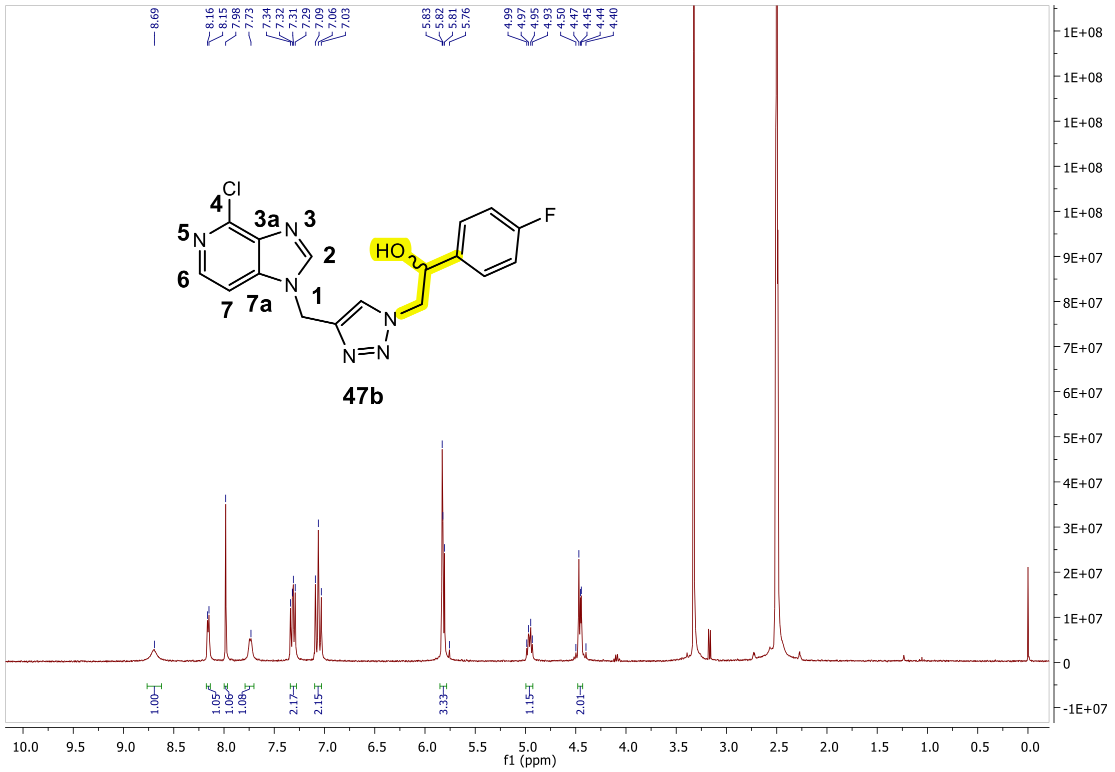

b)

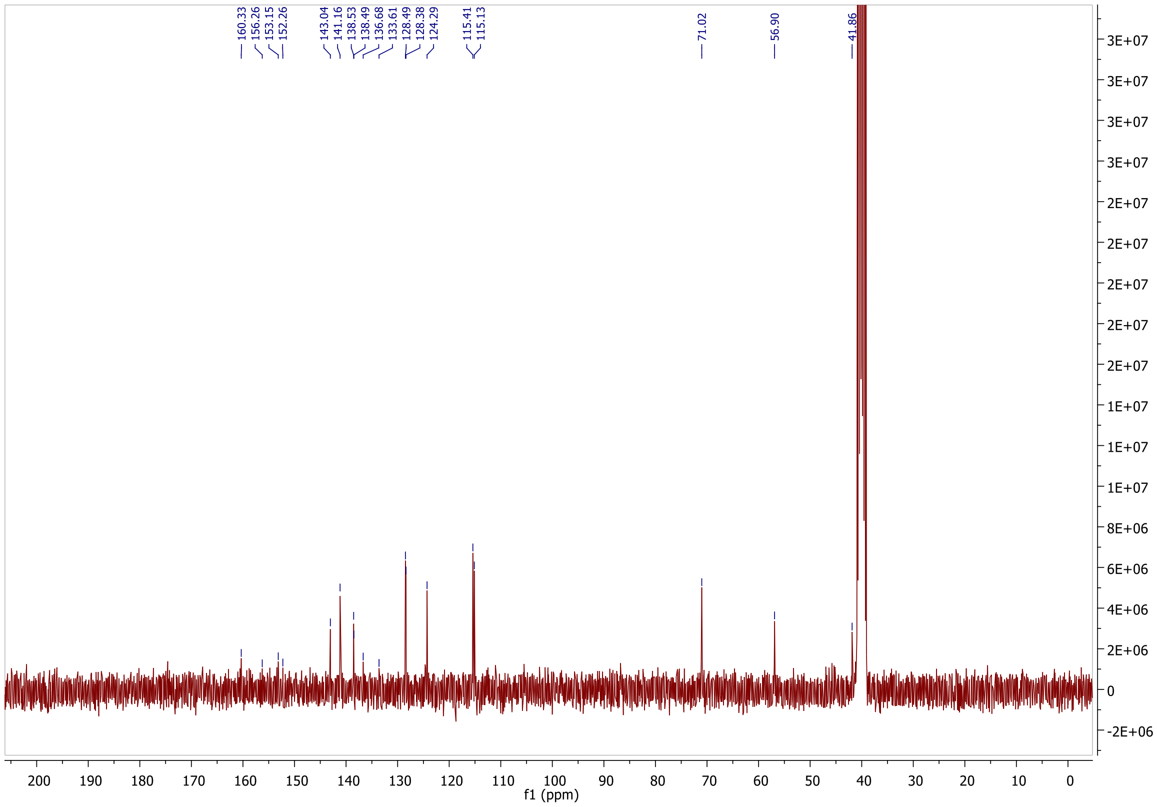

Fig. S47 a) ^{1}H NMR and b) ^{13}C NMR of compd. **48b**

a)

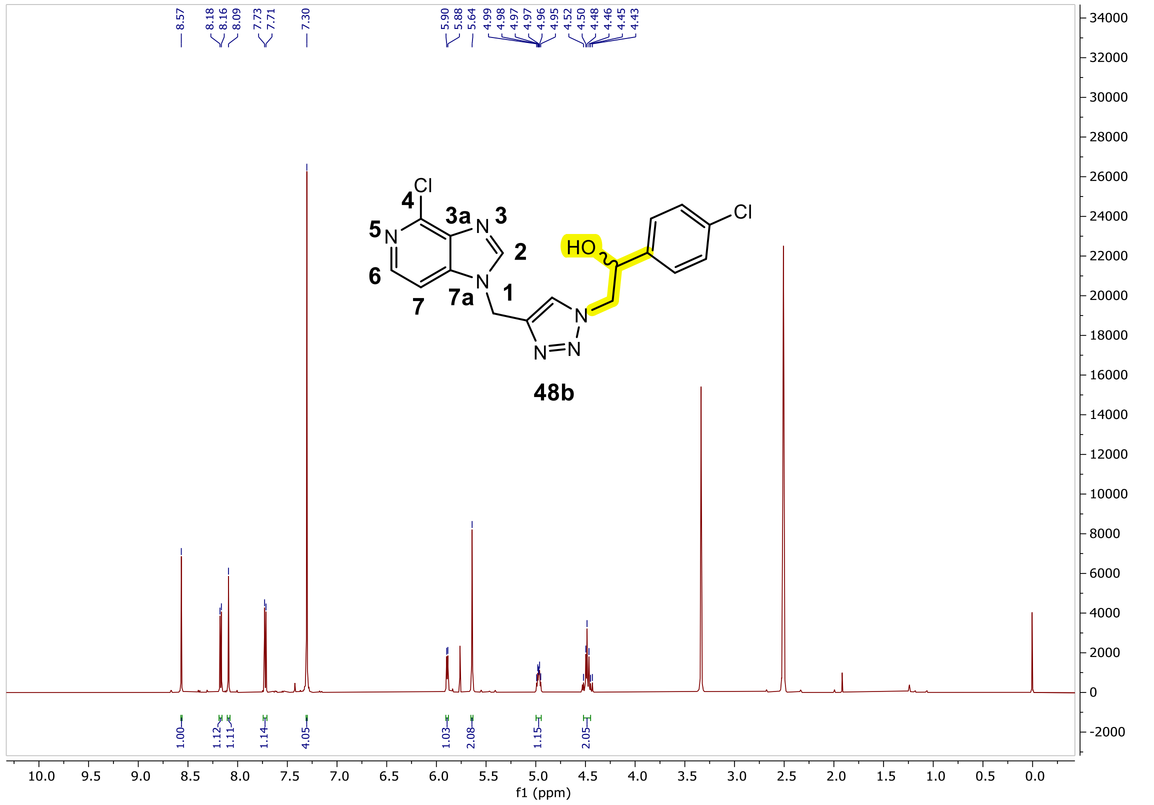

b)

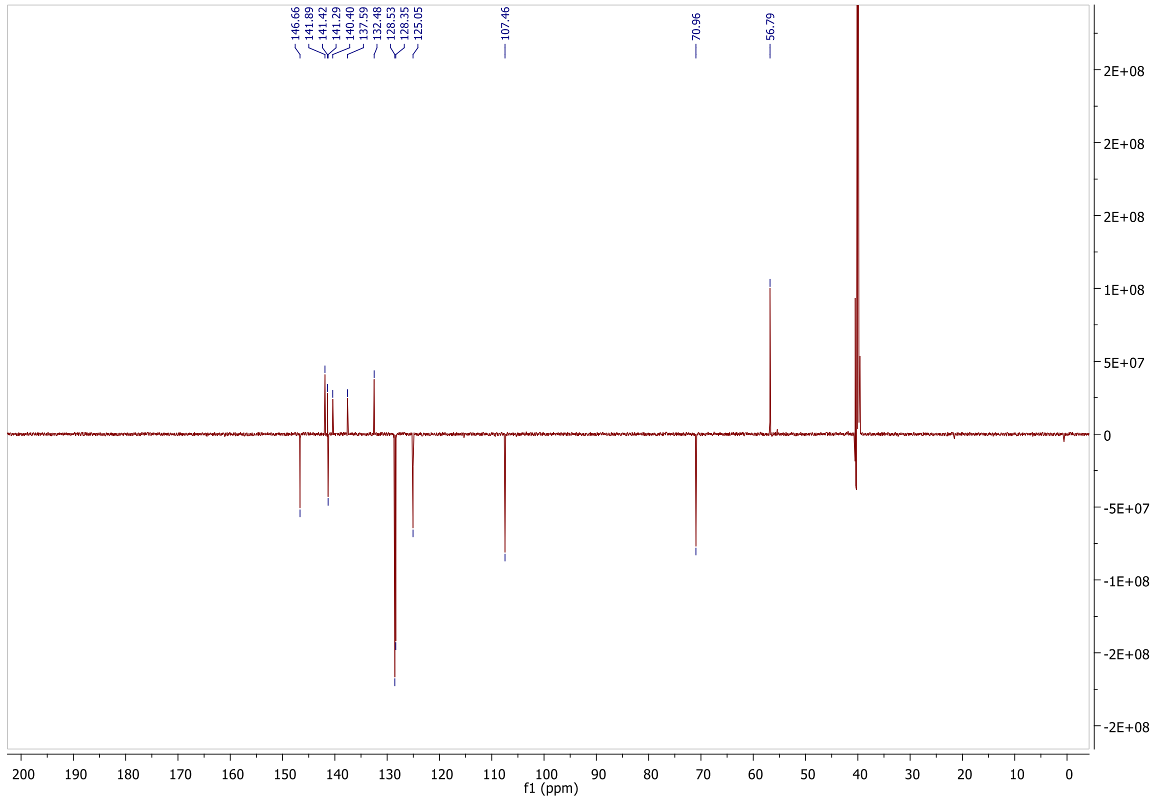

Fig. S48 a) ^{1}H NMR and b) ^{13}C NMR of compd. **49b**

a)

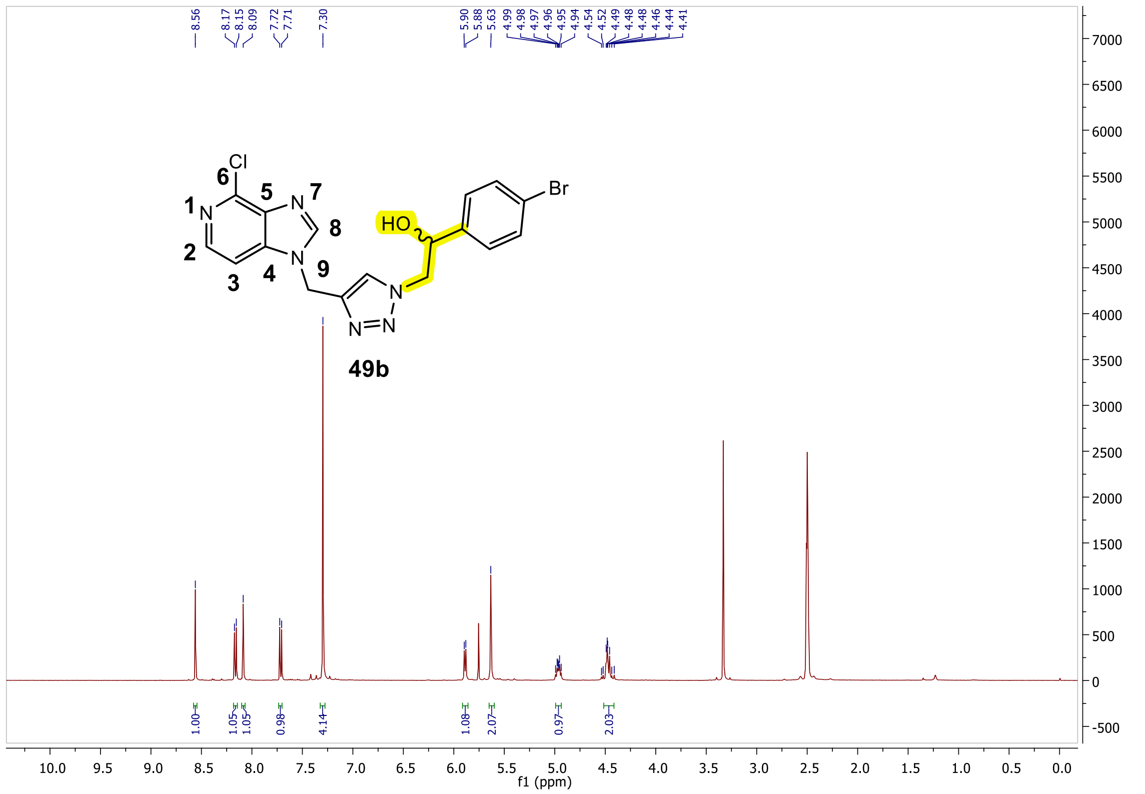

b)

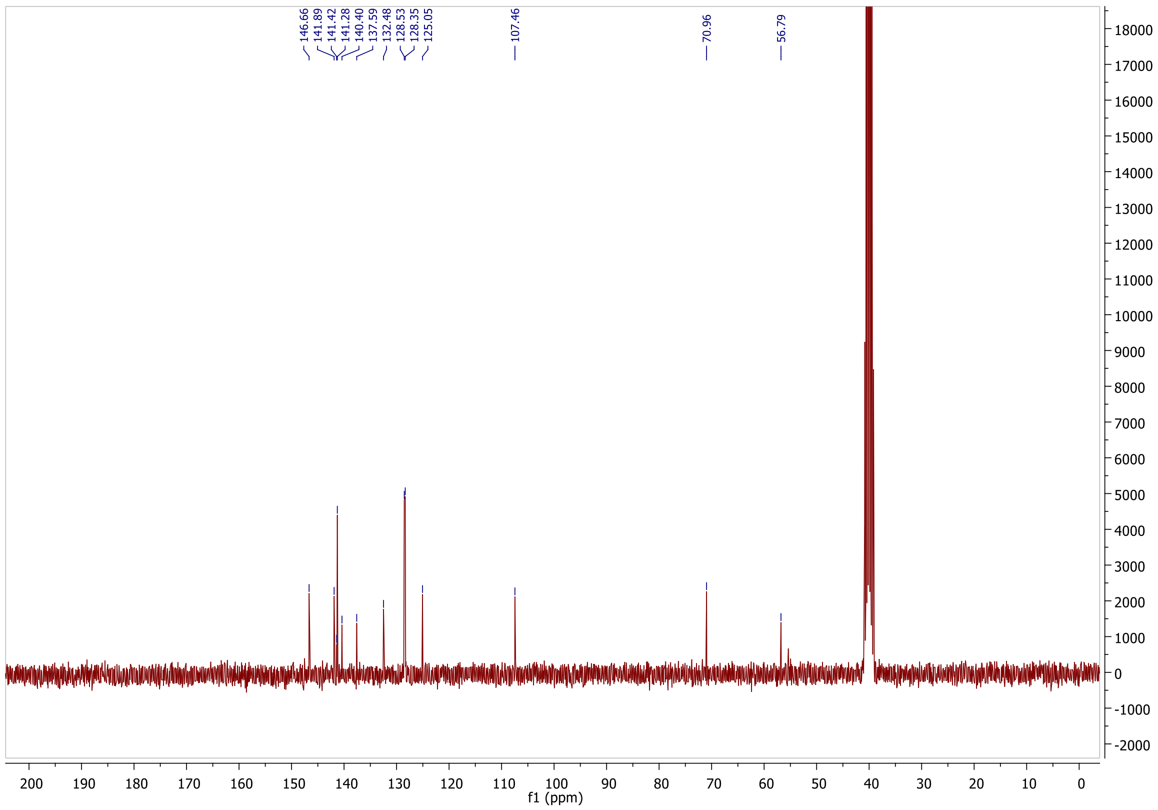

2. UV/Vis absorption spectra of selected compounds

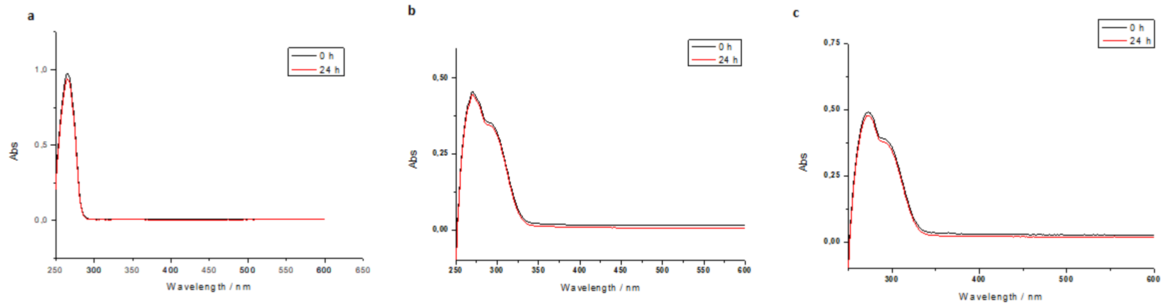

Figure S49. UV/Vis absorption spectra of compounds **40a** (a), **45a** (b), and **49b** (c) recorded in phosphate buffer with 1% DMSO (99:1, v/v) at pH 7.4 ($c = 1 \times 10^{-4}$ mol dm^{-3}). Spectra were measured immediately after sample preparation and after 24 hours to evaluate spectral stability over time.
